# Genetic and biological characterisation of Zika virus isolates from different Brazilian regions

**DOI:** 10.1590/0074-02760190150

**Published:** 2019-08-19

**Authors:** Daisy Maria Strottmann, Camila Zanluca, Ana Luiza Pamplona Mosimann, Andrea C Koishi, Nathalia Cavalheiro Auwerter, Helisson Faoro, Allan Henrique Depieri Cataneo, Diogo Kuczera, Pryscilla Fanini Wowk, Juliano Bordignon, Claudia Nunes Duarte dos Santos

**Affiliations:** 1 Fundação Oswaldo Cruz-Fiocruz, Instituto Carlos Chagas, Laboratório de Virologia Molecular, Curitiba, PR, Brasil; 2 Fundação Oswaldo Cruz-Fiocruz, Instituto Carlos Chagas, Laboratório de Regulação da Expressão Gênica, Curitiba, PR, Brasil

**Keywords:** Zika virus, Flavivirus, molecular markers, biological characterisation

## Abstract

**BACKGROUND:**

Zika virus (ZIKV) infections reported in recent epidemics have been linked to clinical complications that had never been associated with ZIKV before. Adaptive mutations could have contributed to the successful emergence of ZIKV as a global health threat to a nonimmune population. However, the causal relationships between the ZIKV genetic determinants, the pathogenesis and the rapid spread in Latin America and in the Caribbean remain widely unknown.

**OBJECTIVES:**

The aim of this study was to characterise three ZIKV isolates obtained from patient samples during the 2015/2016 Brazilian epidemics.

**METHODS:**

The ZIKV genomes of these strains were completely sequenced and *in vitro* infection kinetics experiments were carried out in cell lines and human primary cells.

**FINDINGS:**

Eight nonsynonymous substitutions throughout the viral genome of the three Brazilian isolates were identified. Infection kinetics experiments were carried out with mammalian cell lines A549, Huh7.5, Vero E6 and human monocyte-derived dendritic cells (mdDCs) and insect cells (Aag2, C6/36 and AP61) and suggest that some of these mutations might be associated with distinct viral fitness. The clinical isolates also presented differences in their infectivity rates when compared to the well-established ZIKV strains (MR766 and PE243), especially in their abilities to infect mammalian cells.

**MAIN CONCLUSIONS:**

Genomic analysis of three recent ZIKV isolates revealed some nonsynonymous substitutions, which could have an impact on the viral fitness in mammalian and insect cells.

ZIKV is a flavivirus (belonging to the family *Flaviviridae*, genus *Flavivirus*) that has recently emerged in many countries and has triggered an epidemic in the human population. For decades, human infections were only sporadic; however, outbreaks were noted in Yap Island in 2007 and in the Pacific Islands in 2013,^1^ and an explosive epidemic occurred in Brazil in 2015.^2^

Prior to 2007, ZIKV had silently circulated in many countries from African and Asian continents with a low impact on public health. However, during the recent emergence, different transmission routes have been reported, and the epidemic profile and self-limiting character of Zika disease were dramatically altered.^3^ After ZIKV introduction to Brazil, the virus raged on in South America and caused debilitating neurological and congenital complications, resulting in significant acute morbidity in adult patients and devastating neurological sequelae in newborns.^4^

As evidenced for other arthropod-borne viruses, a combination of factors ranging from environmental factors to viral genetic changes may have contributed to the selection and spread of new epidemic variants of ZIKV through a naïve population in tropical and subtropical areas. Currently, the occurrence of human ZIKV infections has dramatically decreased in Latin America. This decrease may be partially due to the population’s previous experience with natural ZIKV infection and the subsequent development of immunity.^5^ Additionally, other seasonal changes can influence the biology of vectors and can affect host-pathogen interactions; this generates a variation in the timing and severity of epidemic dynamics, as has been demonstrated by studies on other infectious agents.^6^ Although a decrease in ZIKV transmission has been observed in the last two years, human cases are sporadically detected, which highlights the possibility of a new epidemic event. Clarifying the dynamic of ZIKV infections between animals, human hosts and vectors should aid in preventing new epidemic outbreaks and in developing strategies for controlling ZIKV infections.

The ZIKV genome consists of single-stranded, positive-sense RNA of approximately 11 kb in length. The viral RNA architecture is composed of two noncoding regions at the 5’ and 3’ extremities flanking a single open reading frame (ORF). The viral ORF encodes a polyprotein that, after cleavage by viral and host cell proteases, generates three structural proteins [capsid (C), premembrane (prM) and envelope (E)] that constitute the viral particle and encode seven nonstructural proteins (NS1, NS2A, NS2B, NS3, NS4A, NS4B and NS5) that are mainly involved in viral replication and immune system evasion.^7^

Phylogenetic studies have demonstrated that after ZIKV emerged in Uganda, the virus spread independently and evolved into three lineages (west African, east African, and Asian) that appear to be related to distinct clinical complications in the human host.^8^ In accordance, ZIKV genomic characterisation suggests that during the geographical spread, the Asian lineage has accumulated several mutations that might have influenced the fitness and increased the virulence and the occurrence of new clinical entities associated with the ZIKV infections during the 2015/2016 outbreak.^9^

A better understanding of the contribution of viral genetic markers to human disease and the viral spread in nature might be of paramount importance to design prophylactic or therapeutic strategies to control ZIKV infections. Here, we describe the isolation and present a comparative genetic and biological characterisation of three Brazilian ZIKV clinical isolates selected from two different epidemic periods. Sequence alignment and phylogenetic analysis demonstrated that the isolates reported in this manuscript belong to the Asian lineage. We identified nonsynonymous mutations in the genomes of these Brazilian ZIKV isolates and the biological characterisations of the viruses showed that the mammalian cell lines were more permissive to ZIKV infections than the insect cell lines.

## MATERIALS AND METHODS

*Cell culture* - *Aedes albopictus* cells (C6/36) (ATCC CRL-1660; Manassas, VA, USA) were grown at 28ºC in Leibovitz L-15 medium (Gibco/Invitrogen, Grand Island, NY, USA) supplemented with 0.26% tryptose (Sigma-Aldrich, St. Louis, MO, USA), 25 μg/mL gentamicin (Gibco/Invitrogen) and 5% foetal bovine serum (FBS) (Gibco/Invitrogen). *Aedes pseudoscutellaris* cells (AP61) were grown at 28ºC in Leibovitz L-15 medium supplemented with 0.52% tryptose, 25 μg/mL gentamicin and 10% FBS. *Aedes aegypti* cells (Aag-2) (ATCC CCL-125) were grown at 28ºC in Schneider´s insect medium (Gibco/Invitrogen) supplemented with 25 μg/mL gentamicin, 100 IU/μg/mL penicillin/streptomycin (Gibco/Invitrogen) and 10% FBS. Human hepatoma cells (Huh7.5) (ATCC PTA-8561), human lung epithelial cells (A549) (ATCC CCL-185) and monkey kidney cells (Vero E6) (Sigma-Aldrich, 85020206) were grown at 37ºC under atmospheric conditions of 5% CO_2_ in Dulbecco’s Modified Eagle Medium: Nutrient Mixture F-12 (DMEM/F12) (Gibco/Invitrogen) supplemented with 100 IU/μg/mL penicillin/streptomycin and 10% FBS.

*ZIKV isolation and sequencing* -The serum samples were obtained from two patients living in Natal, Rio Grande do Norte state (RN)/Northern Brazil (latitude: 05º 47’ 51’’ S; longitude: 35º 13’ 34’’ W) in March (strain ZV BR 2015/15098) and in June (strain ZV BR 2015/15261) of 2015, during the beginning of the outbreak in Brazil. The other sample (strain ZV BR 2016/16288) was obtained from a patient living in Teutônia in Rio Grande do Sul state (RS)/South Brazil (latitude: 29º 28’ 18” S; longitude: 51º 49’ 00’’ W) in February of 2016. The use of these samples was approved by Fiocruz and the Brazilian National Ethics Committee of Human Experimentation (CAAE: 42481115.7.0000.5248). The laboratory diagnosis of acute ZIKV infection was confirmed by reverse transcription-quantitative polymerase chain reaction (RT-qPCR).^10^ Additionally, the sera were negative for anti-ZIKV IgM using an in-house enzyme-linked immunosorbent assay (ELISA) based on a previously described methodology.^11^

ZIKV was isolated from human serum samples by direct inoculation of C6/36 (5 x 10^5^ cells seeded in a 25 cm^2^ Tissue Culture Flask) and Vero E6 cells (10^5^ cells seeded in a 25 cm^2^ Tissue Culture Flask) or by intracerebral inoculation of 2-day-old BALB/c mice (CEUA Fiocruz: LW-2/17) as detailed below.

Attempts to isolate the ZV BR 2015/15098 in cell culture were not successful. Therefore, two-day-old BALB/c mice (n = 5) were inoculated intracranially with the serum sample (~ 20 µL). Ten days post-inoculation, the virus was recovered from two of them. The passage 0 (P0) corresponds to a 10% mouse brain suspension in PBS. ZV BR 2015/15261 viral isolation (P0) was performed on C6/36 cells for 22 days of culture, with medium exchange occurring at day 8. The isolation of ZV BR 2016/16288 was successful in both the C6/36 and Vero E6 cells, with the virus collection occurring on day 17 post-inoculation. The viral isolations were confirmed by indirect immunofluorescence^12^ using an anti-ZIKV E protein specific monoclonal antibody (produced by ICC/Fiocruz-PR) and/or by RT-PCR and sequencing.

To perform an *in vitro* biological characterisation of the three ZIKV isolates, we first amplified the ZIKV ZV BR 2015/15098, ZV BR 2015/15261 and ZV BR 2016/16288 isolates by three additional rounds of infection in *Ae. albopictus* C6/36 cells; a low multiplicity of infection (MOI of 0.01) was used to generate working virus stocks (passage 3 - P3). Virus titration was carried out on the same cell line by the focus-forming assay, adapted from a previously described protocol.^13^

The complete genomes of the ZIKV isolates were obtained by sequencing the overlapping PCR products. The viral RNA was extracted using the QIAamp viral RNA mini kit (Qiagen, Hilden, Germany) and was reverse transcribed with Improm-II Reverse Transcriptase (Promega, Madison, WI, USA) and 10 µM random primers. PCR was performed using the LongRange PCR kit (Qiagen) with 0.8 µM primers [Supplementary data **(Table I)**]. The amplicons were purified using High Pure PCR Product Purification Kit (Roche) or QIAquick Gel extraction Kit (Qiagen) and sequenced by Macrogen DNA sequencing service (Seoul, South Korea) in an Applied Biosystems 3730xl DNA Analyzer (Applied Biosystems) using specific ZIKV primers [Supplementary data **(Table II**)].

The ZIKV RNA genomes were assembled using Phred/Phrap/Consed software (http://www.phrap.org/phredphrapconsed.html), and the polyprotein amino acid sequences and the UTRs were annotated. ZV BR 2016/16288 P3 and ZV BR 2015/15261 P3 were shown to have identical sequences to the viruses that were previously sequenced from the P0 and patient sera, respectively; these sequences have been previously deposited (GenBank accession numbers MF073357 and MF073358). ZV BR 2015/15098 P3 was submitted to the GenBank database under the accession number MK566202.

*Sequence analyses* - Two different datasets were used for the phylogenetic analysis. For dataset 1, only the sequences with 100% query coverage in a Blast (https://blast.ncbi.nlm.nih.gov/Blast.cgi) search using ZV BR 2016/16288 as the query were selected. The sequences were aligned using ClustalW as implemented in BioEdit version 7.2.6.1 and were manually edited in the ORF region to avoid codon disruption by inserted gaps. Dataset 2 comprised sequences presenting 100% query coverage in a Blast search using ZV BR 2016/16288 ORF as the query. The sequences were codon aligned using Muscle as implemented in MEGA 7.0.26. Redundant sequences, sequences which presented no information regarding the country or collection date, and the sequences of synthetic constructs were excluded from both datasets. RDP4 Beta 4.97 was used to identify potential recombinants and to exclude them from the alignment as described in. ModelGenerator v0.85 was used for the selection of a nucleotide substitution model. Phylogenetic tree inference was performed in MrBayes v.3.2.6. using three hot chains and one cold chain and was run for 1 million (dataset 1) or 6 million (dataset 2) generations with a 25% burn-in. FigTree v.1.4.3 (http://tree.bio.ed.ac.uk/software/figtree/) and Inkscape 0.92 were used to edit the tree. The HMMTOP tool (http://www.enzim.hu/hmmtop/), which was run with the default settings, was used for the prediction of the transmembrane helices in the NS2A protein. The hydrophobicity plot of the NS4B protein was computed using the ProtScale tool (https://web.expasy.org/protscale/) based on a Kyte & Doolittle scale with the default settings. For the RNA secondary structure prediction in the 3’UTR, the mfold web server (http://unafold.rna.albany.edu/?q=mfold/RNA-Folding-Form) was used with the default settings. Only the highest revised free energy structure is presented.

*In vitro kinetics infection* - To perform the biological characterisation studies of recent ZIKV clinical isolates we used two well-characterised ZIKV strains, MR766 and PE243, as reference viruses. The African ZIKV MR766 strain (GenBank accession number NC_012532) was originally isolated in 1947 from a rhesus monkey. The Asian ZIKV PE243 (GenBank accession number KX197192) was isolated in Recife from a patient with classical symptoms of ZIKV infection in 2015. The ZIKV strains ZV BR 2015/15098, ZV BR 2015/15261, ZV BR 2016/16288, MR766 and PE243 were used to infect mammalian (Vero E6, Huh7.5 and A549 cell lines seeded into 96-well plates at densities of 1.5 x 10^4^ cells/well) and insect (Aag-2, AP61 and C6/36 cell lines seeded into 96-well plates at densities of 1.5 x 10^4^, 3 x 10^4^ and 8 x 10^3^ cells/well, respectively) cells at an MOI of 0.1 and 1 for one hour. After, the viral inoculum was removed followed by the addition of the appropriate media. The percentage of infected cells was evaluated 24, 48 and 72 hours post infection (h.p.i.) by indirect immunofluorescence using an anti-flavivirus monoclonal antibody (mAb 4G2; ATCC HB-112). The cell nuclei were stained with DAPI. The images were obtained with the Operetta CLS high-content imaging system (PerkinElmer) with a 20x objective and were quantified using Harmony software (PerkinElmer).

*Virus biological characterisation* - Initially, an infection kinetics assay was performed using the MOIs of 0.1 and 1 for 24, 48 and 72 h.p.i to select the best time point and MOI to carry the biological characterisation experiments in the different cell lines. Vero E6, Huh7.5 and A549 cells were infected with the ZIKV strains using an MOI of 0.1 and a period of incubation of 48 h. AP61 and C6/36 cells were infected with the ZIKV strains using an MOI of 1 and a period of incubation of 72 h. The cells were plated in 96 well plates, and after the period of incubation, the cells were fixed with methanol:acetone (v/v). The expression of viral E protein was analysed by immunofluorescence using an anti-flavivirus monoclonal antibody (mAb 4G2), as described above. In addition, the same infection was performed in 24 well plates. The cell supernatants were collected for virus titration by focus-forming assay, and the total RNA was extracted from cells for subsequent ZIKV genome RNA quantification. For all the experiments, the mock-infected cells were used as the negative control for the infection. The statistical analyses were performed using one-way ANOVA followed by Tukey’s multiple comparisons test. The differences were considered significant at p ≤ 0.05.

*Virus quantification* - The viral load from the culture supernatants of ZIKV-infected cells was determined by focus-forming assays in C6/36 cells using an anti-flavivirus monoclonal antibody (mAb 4G2).^13^

*ZIKV genomic RNA quantification* - RNA was extracted from mock- or ZIKV-infected cells using the RNeasy Mini Kit (Qiagen) according to manufacturer’s recommendation. ZIKV RT-qPCR was performed in a 20 μL reaction master mix (Promega) containing 10 ng of sample RNA, 500 nM of the ZIKV1086 and ZIKV1162c oligonucleotides and 200 nM of ZIKV1107-FAM probe according to a previously described protocol.^10^ The amplifications were performed with a LightCycler 96 instrument (Roche, Mannheim, Germany). For the analyses of mammalian cells, a similar RT-qPCR protocol was performed to amplify the RNase P housekeeping gene.^14^ For the analyses of mosquito cells, RT-qPCR using 250 nM of the primers 18SF (5’-CCCGTCGGCATGTATTAGCT-3’) and 18SR (5’-CACGGCCGGTACAGTGAAAC-3’) was performed for the amplification of the 18S rRNA gene, which was used as housekeeping gene for those cells. The housekeeping genes were included in all analyses for data normalisation. The relative ZIKV genomic RNA expression level was determined by calculating the 2^-ΔCT^.^15^ The statistical analyses were performed using one-way ANOVA followed by Tukey’s multiple comparisons test. The differences were considered significant at p ≤ 0.05.

*Human monocyte-derived Dendritic Cell culture and ZIKV infection* - The peripheral blood samples were obtained by venipuncture from six healthy donors after written consent was given (Committee of Research Ethics from Fiocruz- CAAE: 49931415.7.1001.5248/Fiocruz). Peripheral blood mononuclear cells (PBMC) were obtained by density gradient separation with Ficoll-Paque PLUS at a density of 1.077 g/mL (GE Healthcare, Chicago, IL, USA). CD14^+^ cells were purified by positive selection with human anti-CD14 Microbeads (Miltenyi Biotec, Auburn, CA) in accordance with the manufacturer’s recommendations. For dendritic cell differentiation, CD14^+^ cells were maintained in RPMI 1640 medium (Lonza) containing 100 IU/mL penicillin (Gibco), 100 µg/mL streptomycin (Gibco), 10% FBS supplemented with 25 ng/mL interleukin-4 (IL-4) and 12.5 ng/mL granulocyte-monocyte colony-stimulating factor (GM-CSF; PeproTech, Rocky Hill, NJ, USA) and were incubated at 37ºC in 5% CO_2._ Three days later, new media was added, and the cells were incubated for 4 more days. The monocyte-derived Dendritic Cell (mdDC) differentiation was confirmed by flow cytometry using anti-CD11c-PE-Cy5, anti-CD209-APC and anti-CD14-BV450 antibodies (all from BD Bioscience, CA, USA).

For ZIKV infection of mdDCs, 10^5^ cells/well were distributed into 96-well plates and were incubated for 1 h 30 min with ZV BR 2015/15098, ZV BR 2015/15261, ZV BR 2016/16288, ZIKV PE243 and ZIKV MR766 at an MOI of 10. After removal of the inoculum, the cells were washed with PBS and were incubated with fresh medium for 24 h at 37ºC in 5% CO_2_. For the negative control (mock), mdDCs were incubated with uninfected C6/36 cell culture supernatants. The cell-free supernatants were collected for virus titration by focus-forming assays and the total RNA was extracted from the cell pellets (RNeasy mini kit - Qiagen) to quantify the ZIKV genomic RNA. The ZIKV infections of mdDCs were evaluated by flow cytometry using anti-human monoclonal antibodies (mAb) as follows: anti-CD11c-PE-Cy5, CD14-BV450 and CD209-APC (BD Pharmingen, CA, USA) and their respective isotype controls. For intracellular ZIKV envelope protein detection, the mdDCs were permeabilised with Cytofix/Cytoperm (BD Biosciences) and were stained with 4G2-FITC at 1:200 (v/v) in Perm/Wash buffer. The cell suspensions were acquired in a FACSCanto II instrument (BD Biosciences) and were analysed by FlowJo software, version 10.0.7 (Tree Star, Ashland, OR, USA). The dendritic cells were first gated as CD11c^+^CD14^-^ cells and then were gated as CD11c^+^CD209^+^ cells; finally, 4G2^+^ cells show the frequency of infected cells. The statistical analyses were performed using PRISM (version 7.0; GraphPad, San Diego, USA). The significance was determined using a paired, nonparametric test (Wilcoxon) among different stimulations in the same group. The values of p ≤ 0.05 were considered significant.

## RESULTS

The ZIKV isolates reported in this study were obtained from the sera of three Brazilian patients who presented mild symptoms of ZIKV infection (malaise, exanthema, mild fever, headache, muscle pain, and periorbital pain). The ZIKV ZV BR 2015/15098 isolate was obtained from the serum sample of a female patient from Rio Grande do Norte state (RN), Brazil. The serum sample was collected on March 2015, in the beginning of the epidemic caused by ZIKV in Brazil. The ZIKV ZV BR 2015/15261 isolate was obtained from the serum sample of a male patient from RN State collected later in June 2015. The ZIKV ZV BR 2016/16288 was obtained from a serum sample with a high viral load (Cq value of 22 by RT-qPCR, Table I). The serum was collected from a female patient from Rio Grande do Sul state (RS) in January 2016, one year after the confirmation of the first cases of ZIKV in the country.

**TABLE I:** Zika virus (ZIKV) isolates described in this study

ZIKV isolate name	GenBank accession number	Patient sample information		
		Date of collection	Location	Cq value^*a*^
ZV BR 2015/15098	MK566202^*b*^	March 2015	Rio Grande do Norte	26
ZV BR 2015/15261	MF073358^*c*^	June 2015	Rio Grande do Norte	27
ZV BR 2016/16288	MF073357^*d*^	January 2016	Rio Grande do Sul	22

*a*: determined by reverse transcription-quantitative polymerase chain reaction (RT-qPCR) with the following primers/probe: ZIKV1086, ZIKV1162c and ZIKV1107-FAM.^(10)^ *b*: sequence generated from P3. *c*: sequence generated directly from patient sera which is identical to P3 sequence. *d*: sequence generated from P0 which is identical to P3 sequence.

A summary with information about the patient samples used for the ZIKV isolations and the GenBank accession numbers is presented in Table I.

The viruses that were recovered from the cell culture supernatant at passage three were fully sequenced. All ZIKV full-length genome sequencing resulted in a 10,807 nucleotide (nt) assembled sequence; this sequence contained an ORF of 10,269 nucleotides in length with 5ʹ (107 nt) and 3ʹ (431 nt) untranslated regions (UTRs). The numbers of nucleotide and amino acid differences among the isolates are shown in Table II.

**TABLE II:** Difference count matrix (nucleotide/amino acid)

	ZV BR 2016/16288	ZV BR 2015/15261	ZV BR 2015/15098
ZV BR 2016/16288	-	38/6	39/4
ZV BR 2015/15261	38/6	-	21/6
ZV BR 2015/15098	39/4	21/6	-

**Fig. 1:**
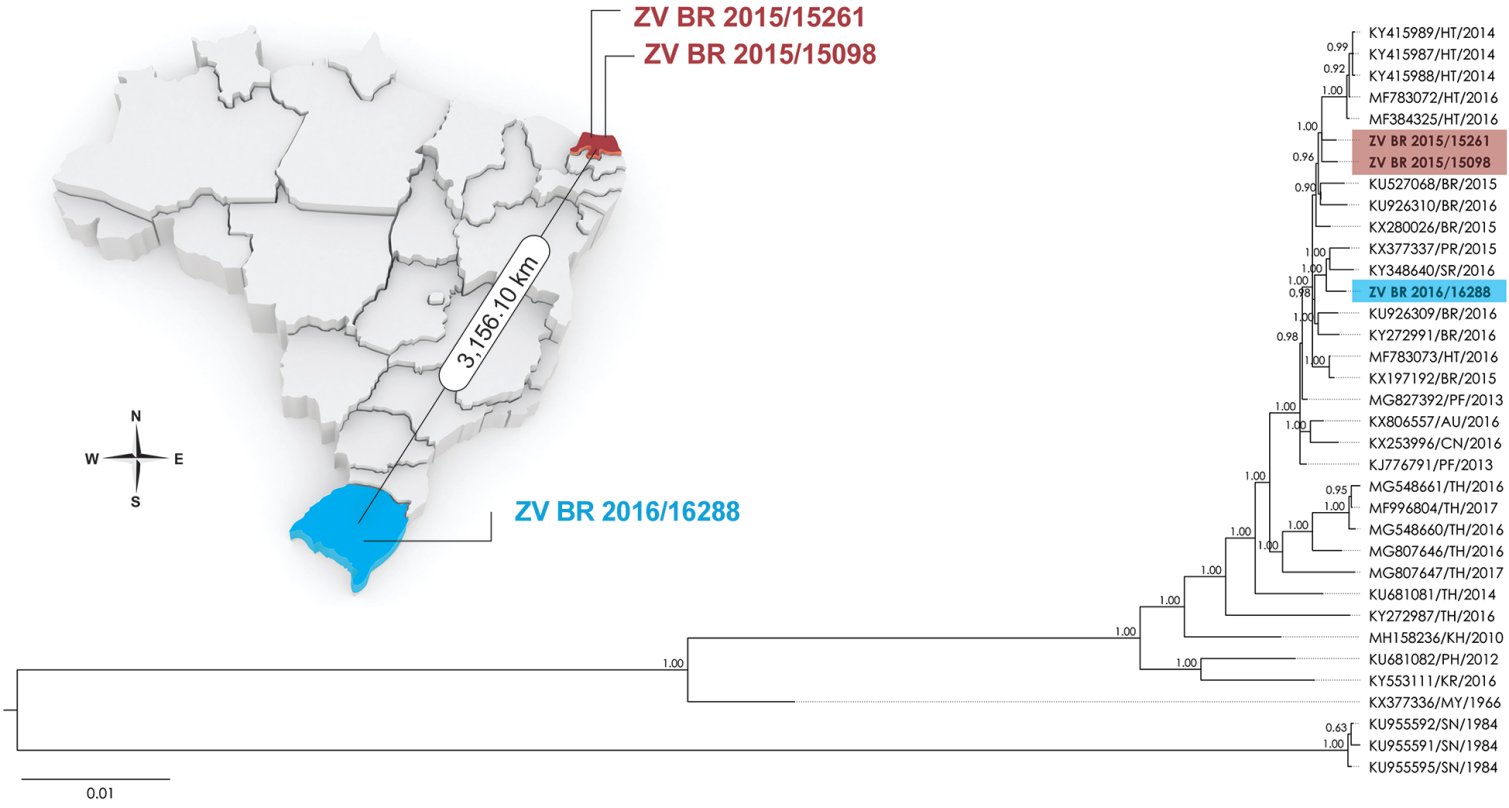
midpoint-rooted consensus tree of Zika virus (ZIKV) complete genome sequences inferred through Bayesian methodology using MrBayes v.3.2.6. based on the general, time-reversible model with gamma-distributed rate variation (GTR+G). The numbers shown to the left of the nodes represent posterior probabilities (ngen = 1000000). The sequences of the third passage of the Brazilian isolates reported in this manuscript are depicted in bold face. The map indicates the places of origin of the samples and the distance in kilometers between them. The following criteria was used for the identification of sequences included in this analysis: GenBank accession number/two letter country abbreviation/year of isolation. Information on country codes can be found in the Supplementary data.

Phylogenetic analyses of either the complete sequence (Fig. 1) or the ZIKV ORF [Supplementary data **(Fig. 1)**] grouped ZV BR 2015/15098 and ZV BR 2015/15261 [see green clade in Supplementary data **(Fig. 1)**] into a different subclade than ZV BR 2016/16288 [see blue clade in Supplementary data **(Fig. 1)**]. We have also analysed our datasets in search of any sign of potential recombination among these new isolates, but none was detected. An alignment of the Brazilian isolates included in the phylogenetic analysis can be found in the Supplementary data. The positions where mutations were identified among the newly reported isolates are evidenced by a light blue arrow. This alignment also includes sequences from the PE243 and MR766 strains (highlighted in green and yellow, respectively) that have been used in the biological characterisation experiments.

A genomic analysis comparing the P3 sequences of the two ZIKV isolates recovered from the patients’ serum from the Brazilian northeast region (RN state) obtained during the same epidemic event three months apart (ZV BR 2015/15098 versus ZV BR 2015/15261) was performed; this analysis showed six amino acid substitutions, more specifically in the E, NS2A and NS3 coding regions (Tables II-III). The genome of the isolate ZV BR 2016/16288 showed two exclusive amino acid substitutions when compared with the others two isolates studied, mapping at NS1 and NS4B proteins. Additionally, only the ZIKV ZV BR 2015/15098 showed a nucleotide substitution at 3’ UTR. As shown in the RNA fold analysis (Supplementary data), this change does not seem to have any impact in secondary structure.

**TABLE III:** Nonsynonymous mutations among the 2015/2016 Brazilian Zika virus (ZIKV) isolates

Polyprotein position	Protein position	ZV BR 2015/15098	ZV BR 2015/15261	ZV BR 2016/16288
		P3-C6/36	P3-C6/36	P3-C6/36
456	E 166	R	K	K
1143	NS1 349	V	V	M
1176	NS2A 30	T	I	T
1180	NS2A 34	I	T	I
1263	NS2A 117	V	A	A
1327	NS2A 181	M	V	M
1594	NS3 92	H	Y	H
2295	NS4B 26	I	I	T

P3: passage 3.

**Fig. 2:**
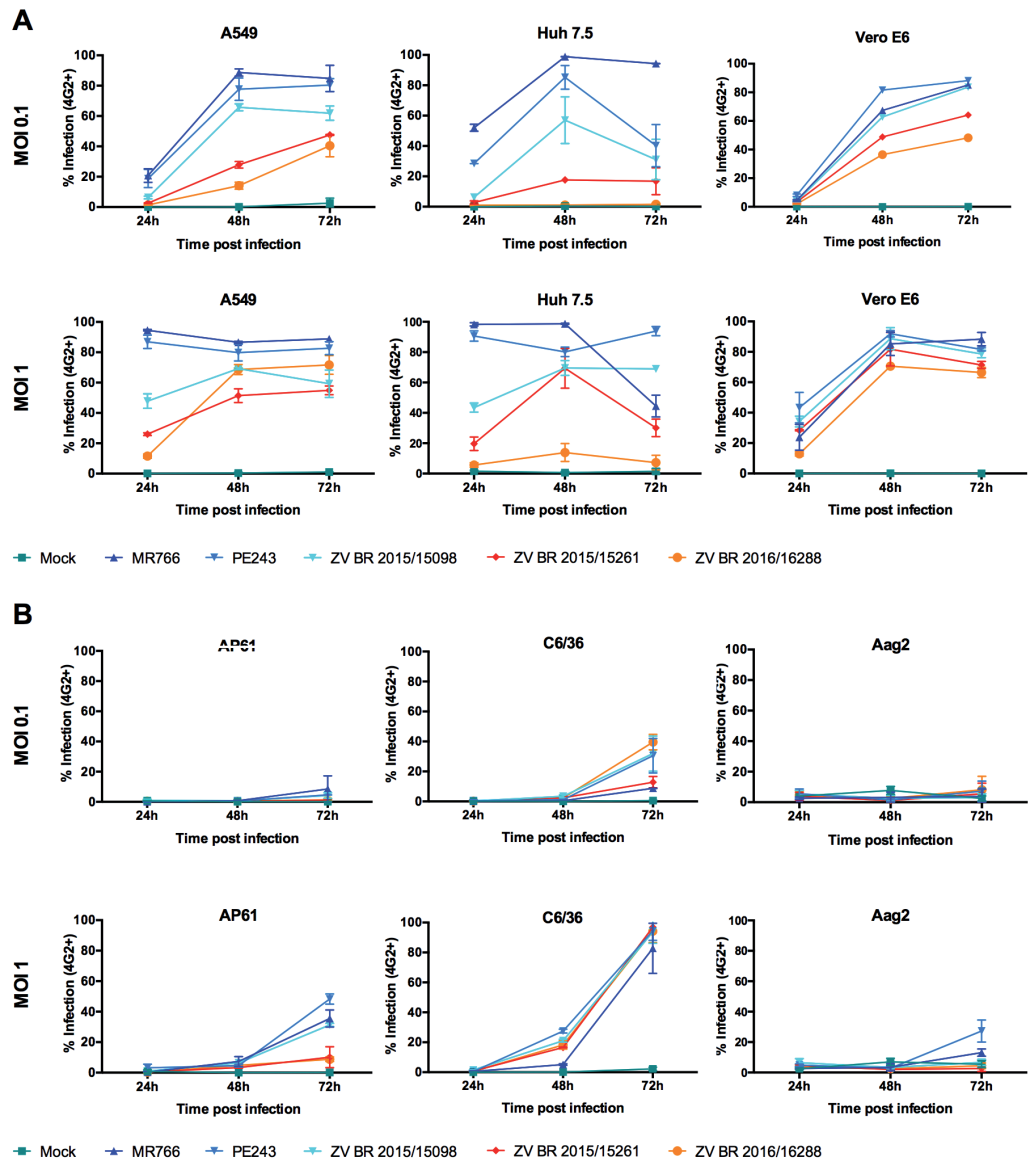
*in vitro* growth kinetics of Zika virus (ZIKV) isolates in (A) mammalian cell lines (A549, Huh7.5 and Vero E6) and (B) mosquito cell lines (AP61, C6/36 and Aag-2). The cells were infected at an MOI = 0.1 or 1 for 24, 48 and 72 h.p.i. The percentage of infected cells was determined by immunofluorescence using an anti-E protein monoclonal antibody (4G2). The bars represent the means ± SD. The results are the means from triplicate experiments.

To study the biological characteristics of the ZIKV Brazilian isolates ZV BR 2015/15098, ZV BR 2015/15261 and ZV BR 2016/16288 and to compare them to the extensively studied and well-characterised ZIKV strains such as MR766 and PE243, different mammalian and insect cell lines were used (Fig. 2). This kinetics analysis was also used to choose the best time and MOI to observe the differences between the virus isolates (Fig. 2). A period of incubation of 48 h and a MOI of 0.1 for the mammalian cell lines (A549, Huh7.5 and Vero E6), and 72 h and an MOI of 1 for the insect cell lines (AP61 and C6/36) were selected. Further studies with Aag2 were not carried out after this cell line was demonstrated to have low susceptibility to the ZIKV isolates tested (Fig. 2B).

**Fig. 3:**
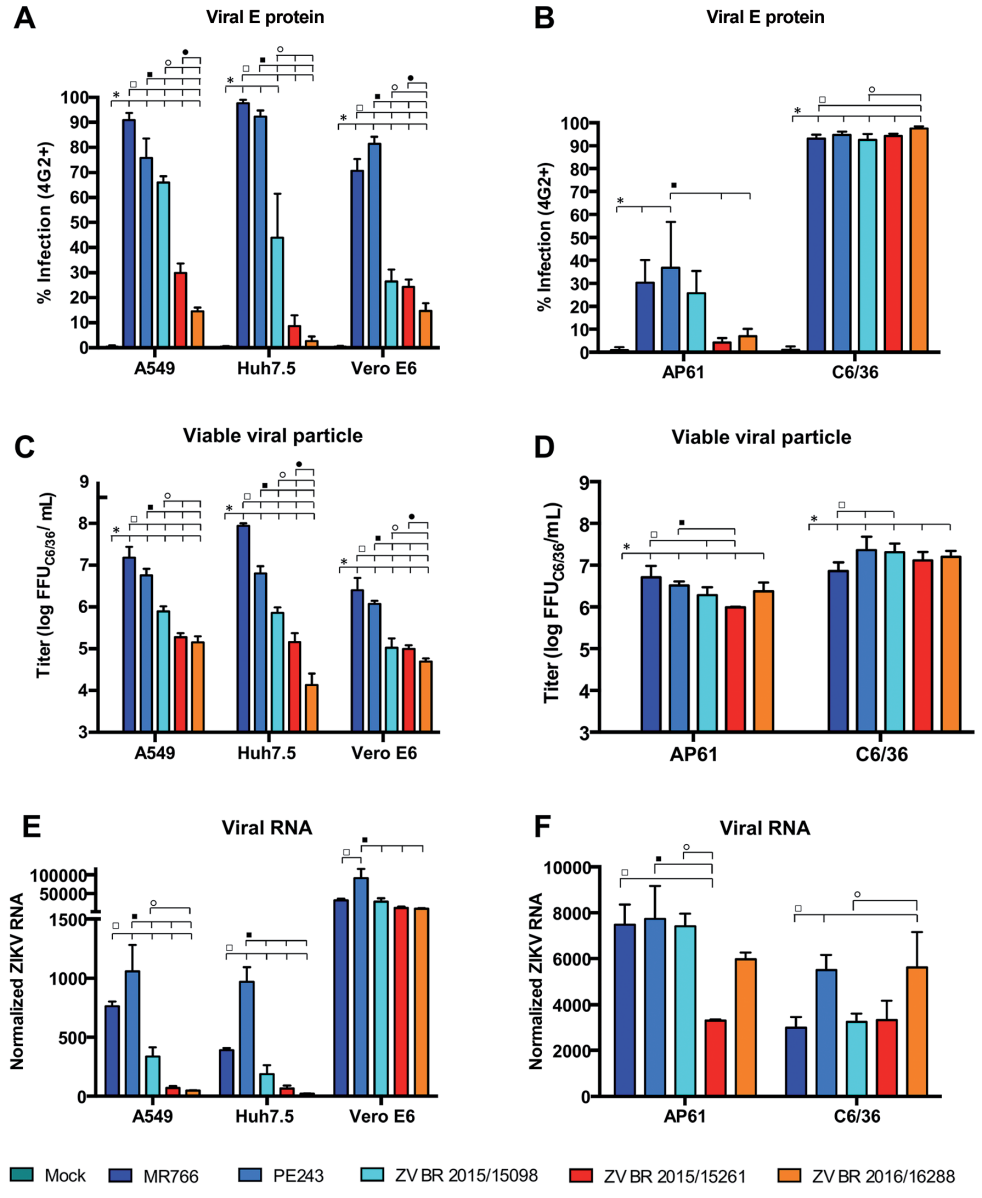
biological characterisation of different Zika virus (ZIKV) strains in mammalian (A, C, E) and mosquito (B, D, F) cell lines. The mammalian cells were infected at an MOI of 0.1 for 48 h; the insect cells were infected at an MOI of 1 for 72 h. (A-B) The frequency of ZIKV-infected cells (4G2^+^) was determined by indirect immunofluorescence. (C-D) The ZIKV progeny in the cell culture supernatant from the ZIKV-infected cells was quantified using a focus-forming assay in C6/36 cells. (E-F) The ZIKV replication was determined by reverse transcription-quantitative polymerase chain reaction (RT-qPCR) on the ZIKV-infected cells. The ZIKV E RNA was quantified using the housekeeping genes RNase P (E) or 18S rRNA (F) for normalisation (2^-ΔCT^). The data were analysed using one-way ANOVA followed by Tukey’s multiple comparisons test. The bars show the mean values ± SD from three independent experiments. * p ≤ 0.05 compared with mock; □ p ≤ 0.05 compared with MR766; ■ p ≤ 0.05 compared with PE243; ○ p ≤ 0.05 compared with ZV BR 2015/15098; ● p ≤ 0.05 p ≤ 0.05 compared with ZV BR 2015/15261.

The abilities of the ZIKV isolates to infect the different cell lines were evaluated based on the percentage of infected cells; this was determined based on the presence of viral E protein, the quantification of viral RNA in infected cells by RT-qPCR, as well as the titration of the cell culture supernatants to access the amount of released infectious viral particles [Fig. 3, Supplementary data **(Fig. 3)**]. However, when compared to the MR766 and PE243 strains, the three Brazilian ZIKV isolates (ZV BR 2015/15098, ZV BR 2015/15261 and ZV BR 2016/16288) had lower percentages of infected cells, RNA accumulation and viral progeny production in A549 and Huh7.5 cells. In Vero E6 cells, this difference was confirmed by analysing the percentage of infected cells and the titers of viral particles released; however, this was not the case for RNA accumulation. Our results show that the genomic RNA accumulation is higher during MR766 infection compared to the three recent Brazilian ZIKV strains in A549 and Huh7.5 cells at 48 h.p.i. However, in Vero E6 cells, the viral RNA levels were similar among the strains studied. The differences between the Brazilian ZIKV isolates and the MR766 and PE243 strains were less evident in the infected insect cells.

Comparisons between the three newly described isolates showed that ZV BR 2015/15098 had the highest infection rate in A549 cells and the highest viral progeny production rates in A549 and Huh7.5 cells. On the other hand, viral RNA accumulation was not significantly different in those cells.

**Fig. 4:**
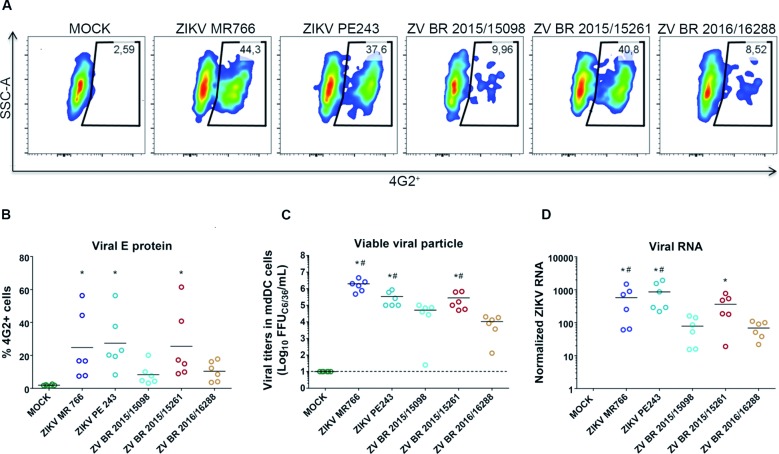
infection of human mdDCs with different Zika virus (ZIKV) strains. (A) A representative density plot containing data that show the mock- and ZIKV-infection (4G2^+^) of mdDCs (CD11c^+^CD14^-^CD209^+^) with five different strains of ZIKV (MOI of 10) after 24 h. (B) The frequency of ZIKV-infected mdDCs (CD11c^+^CD14^-^CD209^+^4G2^+^) was determined by flow cytometry. (C) The ZIKV progeny in cell culture supernatants from mdDCs infected with different strains of ZIKV were quantified using a focus-forming assay in C6/36 cells. (D) ZIKV replication was determined by reverse transcription-quantitative polymerase chain reaction (RT-qPCR) on mdDCs infected with ZIKV. The quantification of ZIKV E RNA using the housekeeping gene RNase P for data normalisation (2^-ΔCT^). The bars show the mean values from the mdDCs of six healthy donors. * p ≤ 0.05 versus ZV BR 2015/15098; ^#^ p ≤ 0.05 versus ZV BR 2016/16288.

Aiming to evaluate the ability of different ZIKV isolates from Brazil to infect a primary human cell target, monocyte-derived dendritic cells (mdDC) were used. After a 24 h infection period, it was possible to demonstrate the presence of envelope protein (4G2^+^) of ZIKV in mdDCs infected with the three clinical isolates of ZIKV (Fig. 4A-B). Flow cytometry analysis showed that the frequency of mdDCs (CD11c^+^/CD14^-^/CD209^+^) infected (4G2^+^) with ZV BR 2015/15261 (25.54% ± 21.12) was similar to the frequency of infection with ZIKV PE243 (27.47% ± 16.98) and ZIKV MR766 (24.84% ± 20.49). Conversely, the ZIKV strains ZV BR 2015/15098 (8.31% ± 6.29), that presented a better viral fitness on A459, Huh7.5 and Vero E6 cells, as well as the ZV BR 2016/16288 (10.40% ± 6.03), were shown to be less efficient in infecting mdDCs compared to the infection efficiencies of the ZV BR 2015/15261, PE243 and MR766 strains (Fig. 4A-B). Unlike the high infection rates observed after 48 h in mammalian cells or 72 h in insect cells, the mdDC infection rates with all ZIKV strains tested decreased over time (data not shown). The virus titration of cell culture supernatants confirmed the results of the abilities of viruses to replicate and release infective particles in cell supernatants (Fig. 4C). Corroborating the flow cytometry data, ZV BR 2015/15261, MR766 and PE243 showed higher titers compared to the titers of ZV BR 2015/15098 and ZV BR 2016/16288 (Fig. 4C).

Finally, quantitative real time PCR confirmed the previous data obtained by FACS and cell culture titration (Fig. 4D). Curiously, in part the results with primary human mdDCs cells were divergent from the results observed in the mammalian cell lines. Despite lower viral fitness in A549 and Huh7.5 cells, ZV BR 2015/15261 strain displayed the highest infectivity rates in the mdDC compared to others ZV BR 2015/15098 and ZV BR 2016/16288 strains. In contrast, ZV BR 2015/15098 that displayed the highest infection rates in A549 and Huh7.5 cells, it has low productive infection in mdDC. As a whole, however, the reference ZIKV strains, MR766 and PE243, and ZV BR 2016/16288 showed the same pattern of infection in both mammalian and mdDC cells.

## DISCUSSION

Phylogenetic studies support the idea that throughout its evolution, the single-stranded positive-sense RNA ZIKV has accumulated genetic changes that may have ensured some selective advantage for virus adaptation and recent epidemic spread of ZIKV Asian lineage. Several studies have experimentally investigated the role of some of these amino acid substitutions, and a correlation between genetic changes and the increased pathogenicity of ZIKV in recent outbreaks was suggested.^16^ ^,^ ^17^ All three ZIKV clinical isolates characterised in this study present the N_139_V_982_V_2634_ (numbering from the start codon of the ZIKV genome) signature characteristic of the American isolates. The substitutions S139N at prM and A982V at NS1 arose during the course of ZIKV spread from Asia to the Americas and were associated with severe microcephalic phenotype in neonatal mouse model and increased virus transmission in mosquitoes, respectively (reviewed by Liu et al.^18^ The M/T2634V substitution at NS5 is believed to be more recent and to have a neutral effect on viral fitness, although it has not been thoroughly studied.^18^ Apart from these genetic determinants of virulence, we identified a unique mutation in the envelope protein (K166R) in the isolate ZV BR 2015/15098 (Table III). This substitution is located in domain I of the envelope protein and does not implicate a change in the biochemical characteristics of the amino acid. The only other sequence in our datasets that presented a nonsynonymous substitution (K166E) in this position was an isolate from the Central African Republic (KF383115).

It is noteworthy that four of the eight substitutions identified here were observed in the NS2A protein, indicating that this small transmembrane protein had the higher number of positions under selective pressure. ZIKV NS2A protein has been previously reported to play a role in the disruption of adherens junction formation, which has been associated with reduced proliferation, premature differentiation and the depletion of radial glial cells in the mouse cortex.^19^ A single mutation at ZIKV NS2A (A175V), which has been shown to decrease virus production and RNA synthesis *in vitro*, was associated with an attenuated phenotype in a ZIKV mouse model.^20^ Based on a study by Xie et al.^21^ and on the transmembrane helices and protein topology prediction program HMMTOP (http://www.enzim.hu/hmmtop/), this mutation (A175V) maps to a transmembrane domain. The NS2A mutation M181V (between ZV BR 2015/15261 and ZV BR 2015/15098 or ZV BR 2016/16288 - Table III) maps to this same domain. The nature of the mutation is also similar, resulting in just a small increase in the amino acid side chain. The amino acid polymorphism identified in ZV BR 2015/15098 NS2A_117_ presents the same character as minor variants previously identified at this same position through next-generation sequencing.^22^ There are five other isolates (KX520666/BR/2015, KY648934/MX/2016, KX446951/MX/2016, MH900227/MX/2016 and KX377336/MY/1966) in our datasets that contain this same substitution (A117V). Based on the homology and the *in silico* prediction, this mutation also maps to a transmembrane domain, and the only amino acids identified at this position are either alanine or valine. Additionally, when the UTRs of the three new ZIKV isolates were compared, one single nucleotide change downstream of the stop codon of the NS5 gene was detected in the ZIKV ZV BR 2015/15098 strain. This mutation was mapped to the highly variable proximal region 1 of the 3ʹUTR sequence (3ʹHVR), a region of the Flavivirus genome known for displaying large heterogeneity in both sequence and length.^23^ The flavivirus 3’UTR contains essential elements that function as important mediators to promote viral genome replication and translation. Despite this, the nucleotide change at 3’HVR of the ZV BR 2015/15098 does not seem to have any impact on the secondary structure, as shown in the RNA fold analysis (Supplementary data). Nevertheless, previous studies have demonstrated that sequence variability at 3’HVR of DENV-1 and DENV-2 are associated with DENV fitness gain or loss in mammalian and insect cells.^23^ ^,^ ^24^

To evaluate the synergistic effect of the mutations identified in each Brazilian ZIKV isolate, we first compared the ability of recent ZIKV isolates and a representative of the African ZIKV lineage to infect mammalian and insect cells. We observed that the ZIKV prototypes (MR766 and PE243) exhibit more protein expression and viral particle production than the recent ZIKV isolates in all mammalian cells tested. However, among the recent isolates, the ZV BR 2015/15098 showed higher infective ability, especially in A549 and Huh7.5 cells. Nonetheless, in insect cells these differences were not so evident. Interestingly, ZV BR 2015/15098 has the NS2A (A117V) polymorphism. Another Brazilian isolate with this same polymorphism (KX520666/BR/2015) has been reported to have higher cytopathogenicity and a higher viral progeny production in different mammalian cell lines (Vero, HEK293T and SH-SY5Y) compared to a virus representative of the early Asian lineage (KF993678). Viral RNA accumulation was also higher in the Vero and HEK293T cells, but no difference was observed in SH-SY5Y cells.^25^

Additionally, we observed that ZV BR 2016/16288, the isolate circulating in southern Brazil one year after the detection of the first case of ZIKV in Brazil, is less infective when compared with the ZIKV prototypes and the 2015 isolates in all cell lineages tested in this study, except in C6/36 cells. In this cell line, the low-passage strain ZV BR 2016/16288 showed very similar viral growth when compared with the other two recent ZIKV isolates. The ZIKV ZV BR 2016/16288 showed one nonconservative point mutation at residue 26 of the N-terminal region of the NS4B protein (Table III) and one point mutation at residue 349 of NS1 protein that should play an important role in ZIKV infectivity, live virus release and viral RNA replication. NS1 protein is a secreted glycoprotein involved in viral replication and immune evasion during flaviviral infection. Further, one non-synonymous mutation at NS1 associated with a single mutation at E protein in a ZIKV strain was found to alter viral fitness in cell culture and pathogenesis in mice.^26^ On the other hand, although the exact topology of flavivirus integral membrane-NS4B on the host membrane compartment is not totally clear, previous studies have shown that the 30 N-terminal residues of NS4B form a dynamic structure that is not associated with the endoplasmic reticulum (ER).^27^ Elazar et al.^28^ demonstrated that in HCV, the modifications of the amphipathic characteristics of NS4B’s N-terminal helix have critical implication for RNA replication. The presence of the threonine residue at NS4B_26_ could disrupt the amphipathic nature of the NS4B helix and impact on NS4B functions in distinct cell lines [Supplementary data **(Fig. 2)**].

By analysing the percentage of cells expressing the E viral protein and production of progeny virus in infected cell lineages we can observe that the mammalian cells are, in general, more susceptible than the *Aedes* cells to the tested ZIKV strains. It is important to emphasize that all three recent ZIKV isolates presented similar profiles of patterns of focus formation in mosquito C6/36 cells; however, in Vero E6 cells, the ZV BR 2015/15098 strain exhibited larger plaque morphology when compared with the smaller plaque phenotype of ZV BR 2015/15261 and ZV BR 2016/16288 (data not shown). These differences can be attributed to the patterns of virus-host interactions in distinct susceptible cell types. Given the observed genotypic and phenotypic differences among the ZIKV isolates, the variations in the amino acid composition of ZIKV proteins could influence the manner by which the viral components interact with host cell, and thus, can modulate their infectivity in different cell substrates.

Human dendritic cells play an important role in antigen presentation to T cells and are also the first target cells of dengue virus after virus infection by a mosquito bite in the skin. It was recently shown that human epidermal keratinocytes, dermal fibroblasts and mdDCs^29^ are also susceptible to ZIKV infection *in vitro*. Several receptors are involved in ZIKV infection, such as AXL, Tyro3, TIM-1, and DC-SIGN, which is mainly expressed on dendritic cells and is also known as CD209.^29^ An analysis of the patients’ blood cells infected by ZIKV revealed that monocytes and myeloid dendritic cells were the main infected cell types; these cell types supported virus replication, and as circulating cells, they could transport the virus to different tissues and fluids.^30^ To characterise the infective profile of the different ZIKV strains in a more relevant cell type in ZIKV pathogenesis, we infected the human mdDCs primary cells. In this cellular model, ZV BR 2015/15261 has showed the better viral fitness when compared to the other recent isolates in contrast to the results observed in the experiments carried using mammalian cell lineages. The higher E protein synthesis, live virus release, and viral RNA level production in human mdDcs for the ZV BR 2015/15261 compared to ZV BR 2015/15098 and ZV BR 2015/16288 isolates suggests that T30I, I34T and M181V polymorphisms in NS2A and H92Y polymorphism in protease domain of NS3 protein (Table III) may contribute to both increasing viral fitness and controlling host antiviral response. The effect of the amino acid substitutions (isolated or in combination) identified among the ZV BR 2015/15261 and ZV BR 2015/15098 and ZV BR 2016/16288 isolates on the modulation of the infection rates in different cell substrates deserves further investigation. It is interesting to note that ZV BR 2015/15261, which presented the lowest infectivity in mammalian cell lines, was the isolate that presented the highest infection rates in the mdDCs from healthy donors, which is a more “physiological” infection model. We speculate that the four amino acid mutations identified in the NS2A and NS3 proteins that distinguish ZV BR 2015/15261 from the other two isolates (isolated or synergistically) could result in an increase in viral fitness during the infection of human dendritic cells.

In conclusion, we successfully isolated ZIKV from serum samples of patients from two different periods of the Brazilian epidemic. We observed significant differences between the clinical isolates and the well-established ZIKV strains (MR766 and PE243) with an emphasis on the abilities to infect mammalian cells. However, our comparison is limited due to the long passage history of these prototype strains in cell culture and/or mice. This reinforces the importance of using isolates with low numbers of passages to study viral pathogenesis. The mutations on the flavivirus genome are known to influence the virulence, replication efficiency and antigen-antibody interaction with the host. In our study, the viral genomes were fully sequenced and revealed some nonsynonymous substitutions, which could have an impact on the viral fitness in mammalian and insect cells. Although there is an increasing amount of data from ZIKV genomic analyses, especially after the 2015/2016 outbreak, there is still a lack of information linking specific amino acid substitution(s) to viral biological characteristics and fitness. Further studies using reverse genetics are essential to define the role of the observed mutations in the differential infection phenotypes observed, especially in human mdDCs. Those future studies could help the understanding of ZIKV replication efficiency and could contribute to the studies of viral attenuation strategies that will ideally lead to vaccine development.
