# Supplementary material for: Genetic and biological characterisation of Zika virus isolates from different Brazilian regions
Source: Mem Inst Oswaldo Cruz. 2019 Aug 19;114:e190150. doi: 10.1590/0074-02760190150 (PMC6701881; doi:10.1590/0074-02760190150)
Supplement: Supplementary file 1 [file 1678-8060-mioc-114-e190150-s.pdf]

Supplementary Material

Table S1: Primers used for the PCR amplification of the complete ZIKV genome

PCR	Primer name	Sequence (5'-3')	DNA Fragment (bp)
1	CNZik 35 - sense<br>CNZik 4 - antisense	AGTTGTTGATCTGTGTGAAATC<br>CCAAGTCCCACCTGACATACC	1037
2	CNZik 5 - sense<br>CNZik 8 - antisense	CGCGTTAGCAGCAGCTGCCATC<br>GGGGTTAGCGGTTATCAACC	1189
3	CNZik 7 - sense<br>CNZik 12 - antisense	GGCTCCCAGCACAGTGGG<br>CAGATGGGCCCTCTTCAGCC	1732
4	CNZik 11 - sense<br>CNZik 16 - antisense	GCCGTCTCTGCTGATGTGGGG<br>CGGCCATGGGCCCAGCCATCTC	1846
5	CNZik 15 - sense<br>CNZik 18 - antisense	GTGCAGGAAGGGCTGAAG<br>GTTGGGGACTCTGATTGGCTG	1845
6	CNZik 19 - sense<br>CNZik 20 - antisense	GAGGAGGGAGGAAGAGAC<br>CAGGGCCGCATGATCTGAAC	1313
7	CNZik 23 - sense<br>CNZik 24 - antisense	GCCGGAATAACCTACACAG<br>GCGGATGGTGGCGGCGTAG	1686
8	CNZik 27 - sense<br>CNZik 28 - antisense	GACGGTGTGGCAACGGGAGGC<br>GGCCAATGCCAAGGCCCTGTGCCC	1569
9	CNZik 31 - sense<br>CNZIK 36 - antisense	CCAGGAGGAAGGATGTATGCAG<br>AGACCCATGGATTTCCC	1562

Table S2: Primers used for Sanger sequencing

Primer name	Sequence (5'-3')	Position (MF073358)	Polarity
CNZik 35	AGTTGTTGATCTGTGTGAATC	1 to 21	Forward
CNZik 34	GCTCACACGGGCTACTCCGCG	162 to 182	Reverse
CNZik 3	GCAGCGGAGGTCACTAGACG	471 to 490	Forward
CNZik 2	CACCCCCTCATCCAGCATAGGGC	631 to 653	Reverse
CNZik 5	CGCGTTAGCAGCAGCTGCCATC	878 to 899	Forward
CNZik 4	CCAAGTCCCACCTGACATACC	1017 to 1037	Reverse
CNZik 7	GGCTCCCAGCACAGTGGG	1410 to 1427	Forward
CNZik 6	GGTGGCTTCGGCTCTTGGTG	1495 to 1514	Reverse
CNZik 9	CCAGCTCAGATGGCGGTGGAC	2001 to 2021	Forward
CNZik 8	GGGGTTAGCGGTTATCAACC	2047 to 2066	Reverse
CNZik 11	GCCGTCTCTGCTGATGTGGGG	2478 to 2498	Forward
CNZik 10	GACTGCTGCTGCCAATCTACG	2607 to 2627	Reverse
CNZik 13	GGGAAAGGAGGCTGTACACAG	3056 to 3076	Forward
CNZik 12	CAGATGGGCCCTCTTCAGCC	3121 to 3140	Reverse
CNZik 15	GTGCAGGAAGGGCTGAAG	3603 to 3620	Forward
CNZik 14	CGCGAAGGTGGCACCCATC	3725 to 3743	Reverse
CNZik 17	GGGAAGCGGAGCTGGCCCCC	4215 to 4234	Forward
CNZik 16	CGGCCATGGGCCCAGCCATCTC	4302 to 4323	Reverse
CNZik 19	GAGGAGGGAGGAAGAGAC	5117 to 5134	Forward
CNZik 21	GCAGTCAATGTCACCCACTC	5352 to 5371	Forward
CNZik 18	GTTGGGGACTCTGATTGGCTG	5427 to 5447	Reverse
CNZik 23	GCCGGAATAACCTACACAG	6267 to 6285	Forward
CNZik 20	CAGGGCCGCATGATCTGAAC	6412 to 6431	Reverse
CNZik 25	CCCCTGACCCTAATAGTGGCC	7230 to 7250	Forward
CNZik 22	GCTGCTGCCTGCAGCCCTGG	7287 to 7306	Reverse
CNZik 27	GACGGTGTGGCAACGGGAGGC	7803 to 7823	Forward
CNZik 24	GCGGATGGTGGCGGCGTAG	7934 to 7952	Reverse
CNZik 29	GCGGCTGAGCCGTGTGACACG	8073 to 8093	Forward
CNZik 26	GTCTGGCACCCTAGTGTCCAC	8745 to 8765	Reverse
CNZik 31	CCAGGAGGAAGGATGTATGCAG	9246 to 9267	Forward
CNZik 28	GGCCAATGCCAAGGCCCTGTGCCC	9348 to 9371	Reverse
CNZik CS	CCAGGGGCGGGATGGAGCATCCGGGAG	9897 to 9923	Forward
CNZik 30	GGCATTGGCCATCAGTCGGAGGTC	9984 to 10007	Reverse
CNZik3'UTR	GGGTCTACACCTGGAGTGCTGTAA	10356 to 10379	Forward
CNZik 32	GCCATGGCGTTCTCGGCCTG	10483 to 10502	Reverse
CNZik 33	GCTGCCTGTGAGCCCCTCAG	10517 to 10536	Forward
CNZik fim	TCTCCTCTAACCACTAGTCCC	10658 to 10678	Reverse
CNZIK36	AGACCCATGGATTTCCC	10791 to 10807	Reverse
CNZIK37	CCCGTCCCAGGCGGCATCTAGC	4865 to 4886	Reverse
CNZIK38	GGAGTGGGAGTTATGCAAGAGGGG	4722 to 4745	Forward
CNZIK39	CGGCAATGAGATCGCAGCTTG	5717 to 5737	Forward
CNZIK40	CCCCAGGACAACCAAATGGCA	6849 to 6869	Forward

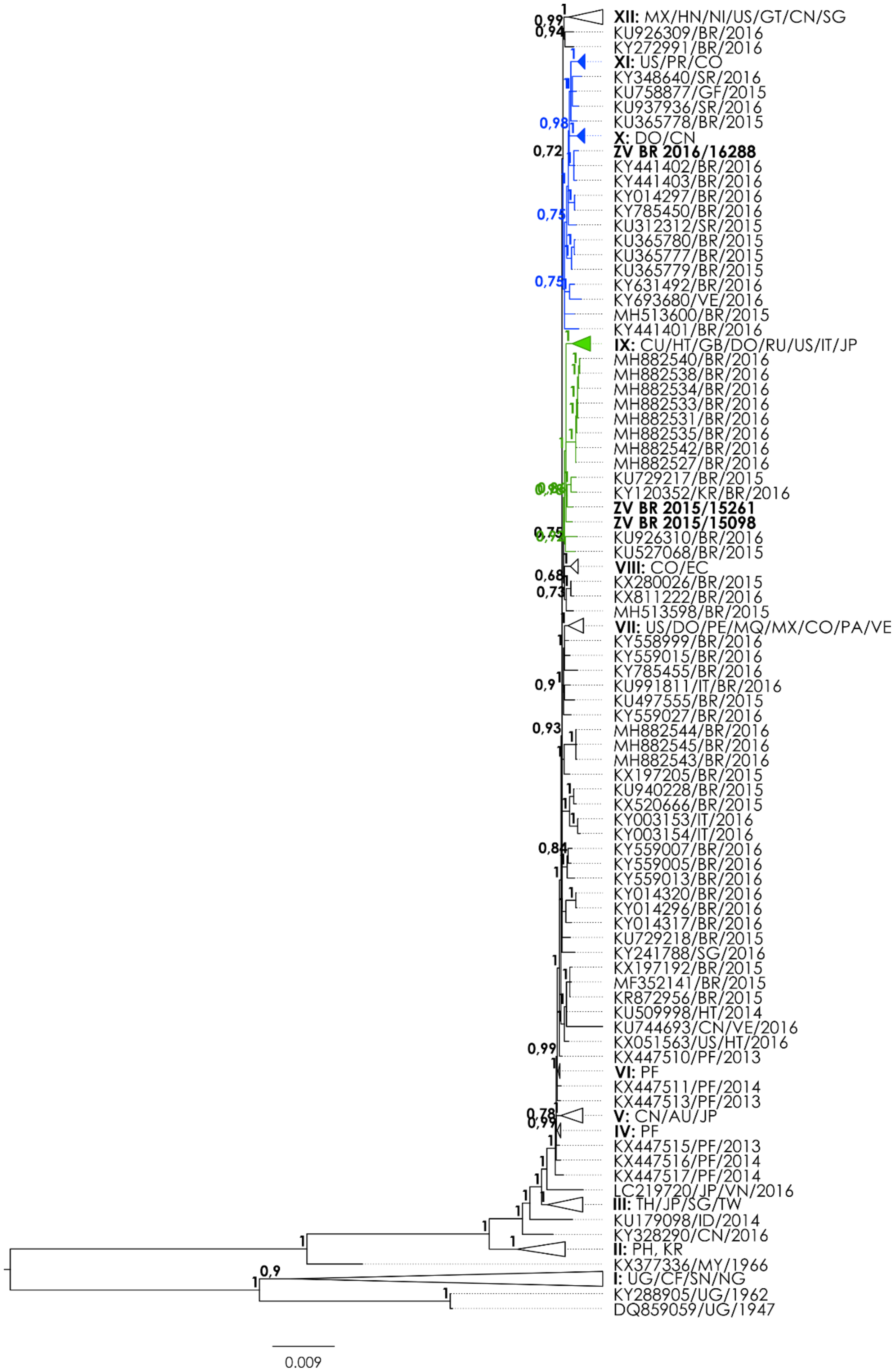

Figure S1: Midpoint rooted consensus tree of ZIKV complete ORF sequences inferred through Bayesian methodology using MrBayes v.3.2.6. based on the general time reversible model with gamma-distributed rate variation and a proportion of invariable sites (GTR+I+G). The numbers shown to the left of the nodes represent posterior probabilities (ngen = 6000000). The sequences of the third passage of the Brazilian isolates reported in this manuscript are depicted in bold face. The following criteria was used for the identification of sequences included in the analysis: GenBank accession number/two letter country abbreviation/year of isolation. The informed country code refers to the country of isolation. Imported cases with clear information on the country of infection present a second country code. Information on the collapsed clades and country codes can be found in the Supplementary material.

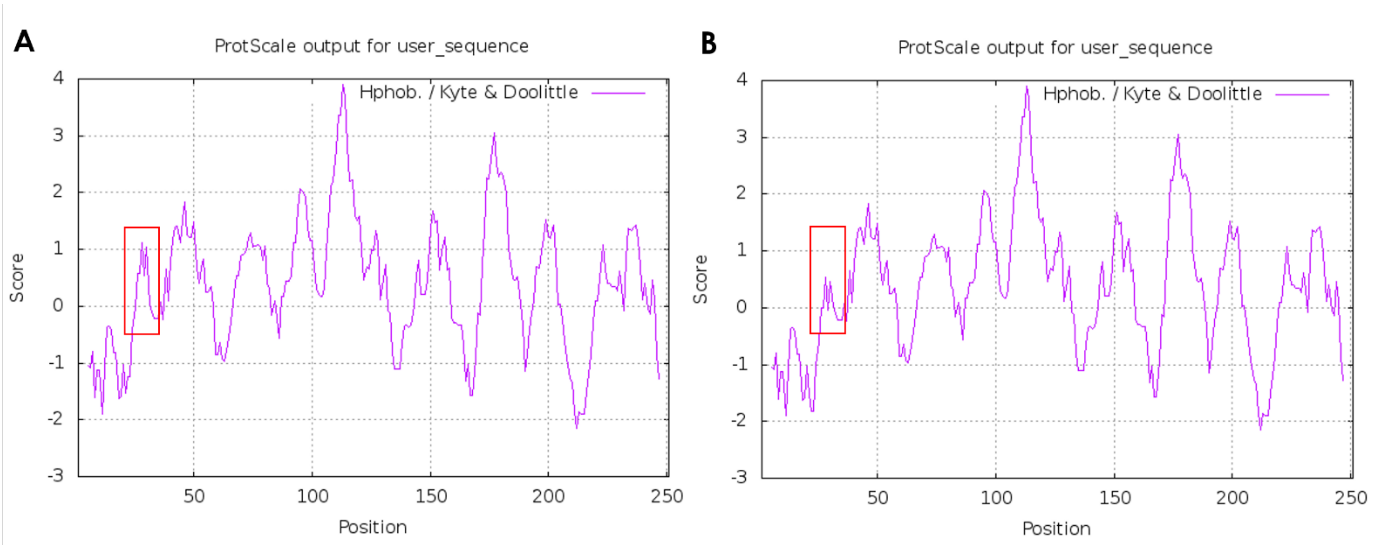

Figure S2: NS4B hydrophobicity plot. (A) represents ZV BR 2015/15098 or ZV BR 2015/15261 and (B) represents ZV BR 2016/16288. The region affected by the NS4B26 mutation is highlighted by the red box. The Kyte & Doolittle hydrophobicity plot was drawn using the default settings, as available at the ProtScale website (http://web.expasy.org/protscale/).

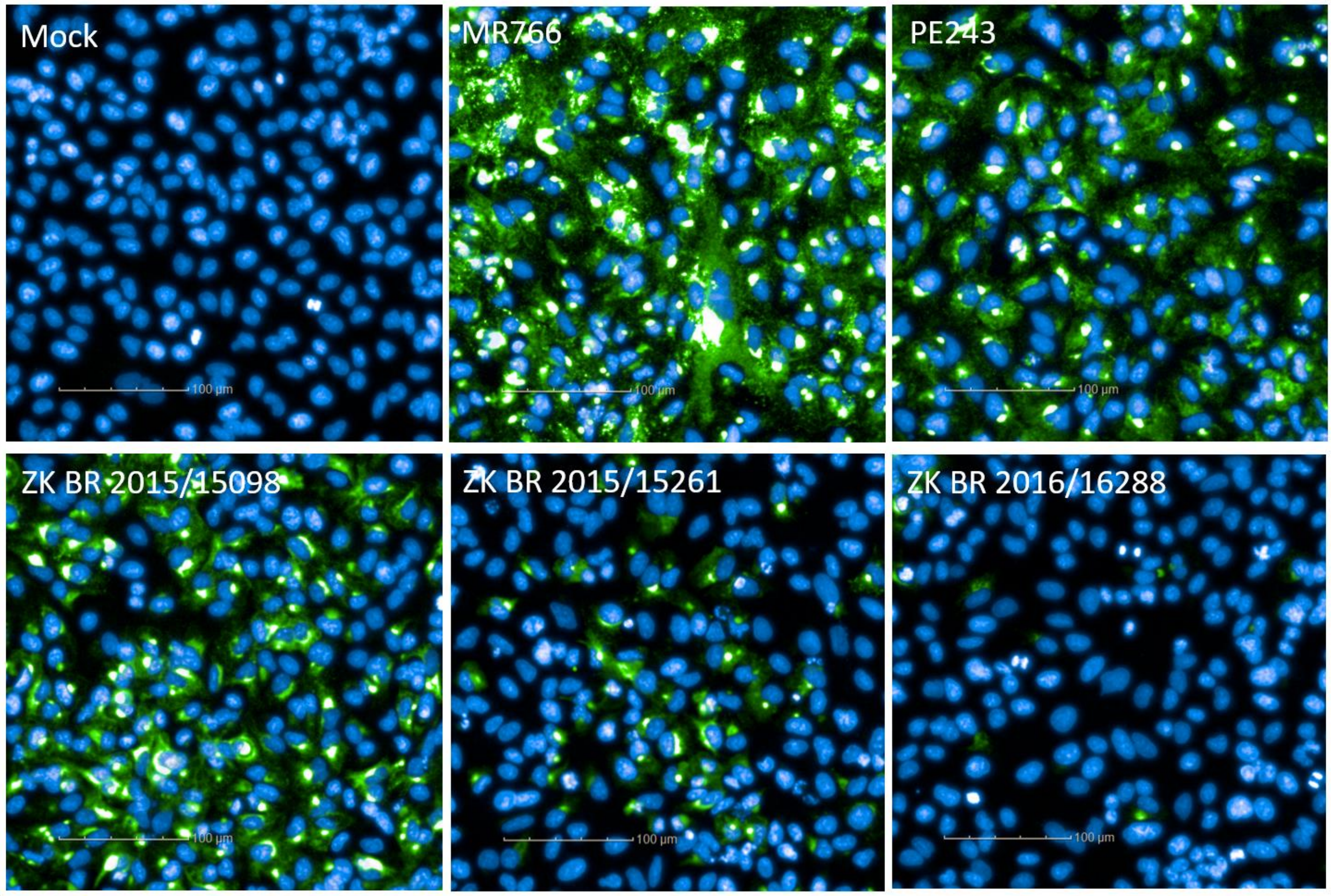

Figure S3: ZIKV strains infect A549 cell line at different rates. Immunofluorescence images show higher number of infected cells for ZV BR 2015/15098, PE243 and MR766 and lower infection rates for ZV BR 2015/15261 and ZV BR 2016/16288, after 48 h of infection.

Information on the collapsed clades in Figure S1:

I (Uganda, Central African Republic, Senegal, Nigeria): KU963574, HQ234500, MF510857, HQ234501, KX198134, KX601166, MG758786, KU955591, KU955592, KU955595, KF383115, KF268949, KF268950, KF268948, KX601169, LC002520, KX377335, KX830960, KX421193, KY989511, MK105975, KU955594, KU963573

II (Philippines, South Korea): KY120353, KY553111, KU681082
III (Thailand, Japan, Singapore, Taiwan): MG807647, KY126351, KY241691, MH013290, MF692778, LC369584, MG548660, MF996804, MG548661, MG807646, MH255601, KX827309, KY241683, KY241696, KY241704, KY241720, KY241726, KY241754, KY241758, KY241679, KY241733, KY241722, KY241725, KY241687, KY241781, KY241779, KX813683, KY241713, KY241689, KY241736, KY241692, KY241690, KY241694, KY241730, KY241787, KY241766, KY241673, KY241678, KY241767, KY241675, KY241756, KY241727, KY241780, KY241682, KY241778, KY241776, KY241729, KY241714, KY241734, KY241777, KY241751, KY241782, KY241773, KY241738, KY241697, KY241671, KY241716, KY241783, KY241749, KY241706, KY241721, KY241740, KY241698.
IV (French Polynesia): KX447514, KX447509, KJ776791
V (China, Australia, Japan): LC191864, KX806557, KU761560, KU761561, KX117076, MH055376, KU963796, KU820899, KU997667, KU866423, KX013000, MF964216, KY967711, KX266255, KU955589, MG674719, MG674718
VI (French Polynesia): KY766069, KX369547, KX447512
VII (USA, Dominican Republic, Peru, Martinique, Mexico, Colombia, Panama, Venezuela): KY075932, KX548902, MH179341, KY317937, KY693679, KY693678, KU647676, KU922923, KU922960, KY785466, KX198135, KY014303, MK049247, KY785469, MH544701, KY317940, KY317938, KY317936, KY317939, KY989971, KX156775, KX156776, MF574585, MF574587, KX247646, KX702400, KX893855, KU820897, MF574561, MF574570, MF574566, MF574565, MF574555, MF574573, MF574556, MF574558, MF574557, MF574554, MF574559, MF574572, MF574575, MF574567, MF574571, MF574562, MF574560, MF574568, MF574569, MF574563, MF574564
VIII (Colombia, Ecuador): MK049248, MF794971, KX879603, KX879604
IX (Cuba, Haiti, United Kingdom, Dominican Republic, Russia, USA, Italy, Japan): MH063261, MF384325, MF783072, KY415986, KY415989, KX673530, KY415988, MF438286, KY785441, LC190723, KY014305, MF098766, KX269878, MH063264, KY014314, LC331561, KY785475, KY014318, KY785420, KY785415, KY014321, KY014302, KY785476, KY014304, KY785435, MF664436, MF098768, KX922707, KU853012, KU853013, KY014300, KY325476, KY325464, KY785422, KY075937, KY325473, KX922705, KX838906, MF988743, KY014299, KX922708, KY325467, KY014325, KX922706, KX832731, KX842449, KY075936, KY075935, KY075938, KY014324, KX838904, KX922703, KY325469, KY014323, KY325468, KY785468, KY075939
X (Dominican Republic, China): KX766028, KU761564, KU820898, KX056898, KY379148, MF167360, KU955590
XI (USA, Puerto Rico, Colombia): KY075933, KX377337, KU501215, KY075934, KY785464, KX087101, MH916802, MH916806, MF574578, MH916803
XII (Mexico, Honduras, Nicaragua, USA, Guatemala, China, Singapore): KY765323, KY765326, MF801406, MF801378, MF801412, MF801384, KY927808, MF593625, KY325465, MF801426, MF988734, MH063262, MF159531, MF801396, MF801402, MF801418, KX906952, KX694534, KX262887, MF801381, MF801403, KU501216, KU501217, MG494697, KY606273, KY014306, KY693677, KY765325, MF801410, KY014315, KY785418, KY765324, KY014312, KY328289, KY013310, KY785442, KY014319, MF434521, KY765318, KY765317, KU870645, MF801387, KY765320, KX421194, MF434517, MF434516, KY785448, MF434522, KY693676, MF098771, MF801398, MH157208, MH157213, MH157202, MF801395, MF801414, MF801417, KY648934, KX856011, KX766029, KY606272, MF801413, KY631494, KY631493, KY120349, MH900227, KY120348, KX446950, KX446951, KX24763

Country codes used in Figure 1 and S1:
AU: Australia, BR: Brazil, CF: Central African Republic, CN: China, CO: Colombia, CU: Cuba, DO: Dominican Republic, EC: Ecuador, GB: United Kingdom, GF: French Guiana, GT: Guatemala, HN: Honduras, HT: Haiti, ID: Indonesia, IT: Italia, JP: Japan, KH: Cambodia, KR: South Korea, MQ: Martinique, MX: Mexico, MY: Malaysia, NG: Nigeria, NI: Nicaragua, PA:

Panama, PE: Peru, PF: French Polynesia, PH: Philippines, PR: Puerto Rico, RU: Russia, SG: Singapore, SN: Senegal, SR: Suriname, TH: Thailand, TW: Taiwan, UG: Uganda, US: USA, VE: Venezuela, VN: Viet Nam.

3’UTR secondary RNA structures

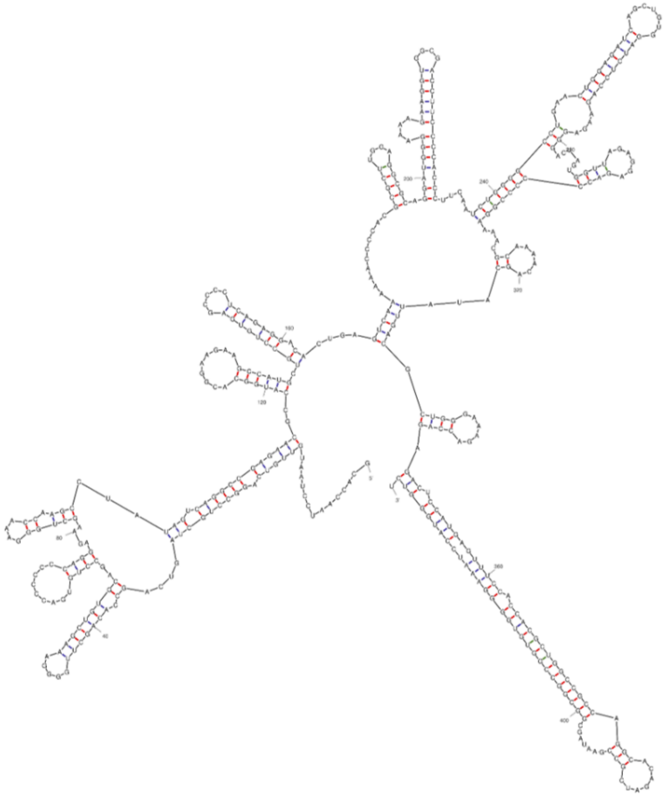

dG = -173.57 [Initially -176.40] ZV BR 2015/15098

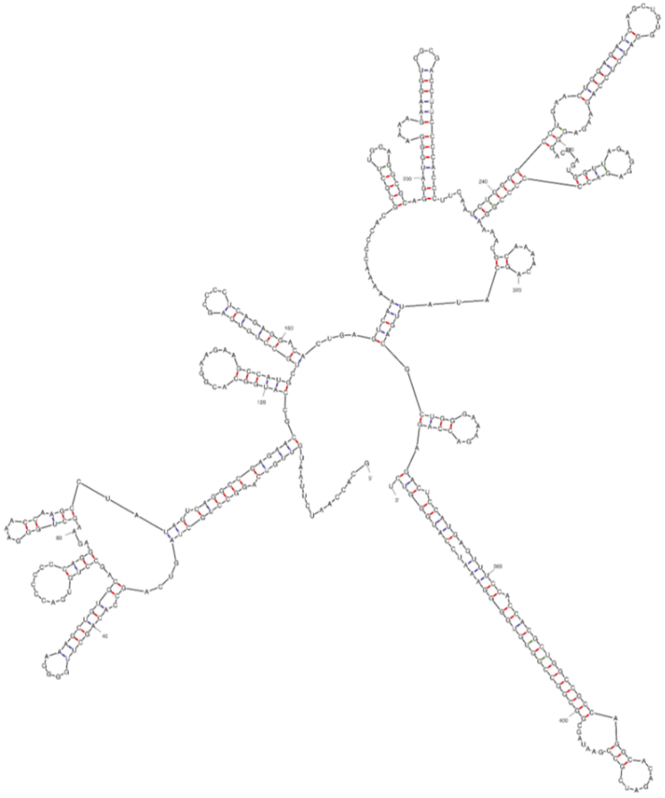

dG = -173.57 [Initially -176.40] ZV BR 2015/15261

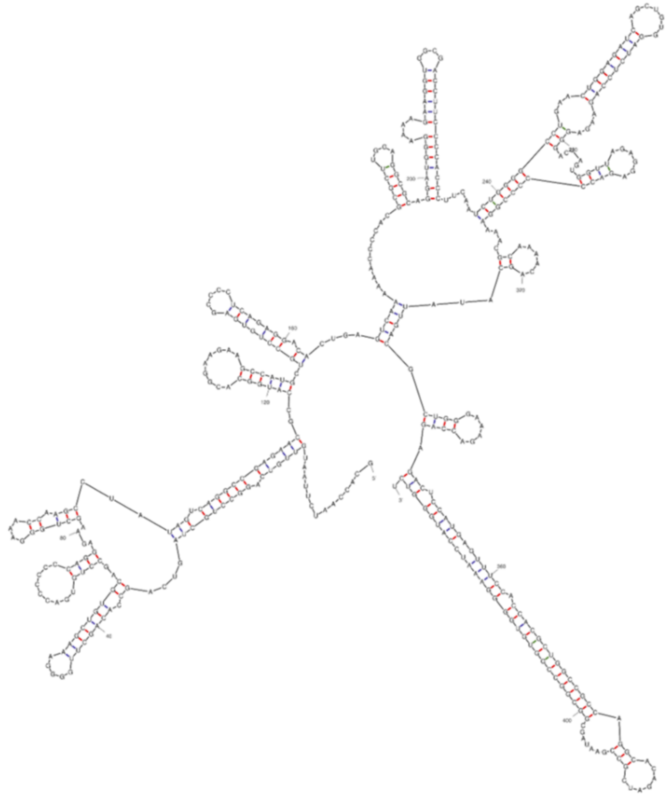

dG = -173.57 [Initially -176.40] ZV BR 2016/16288

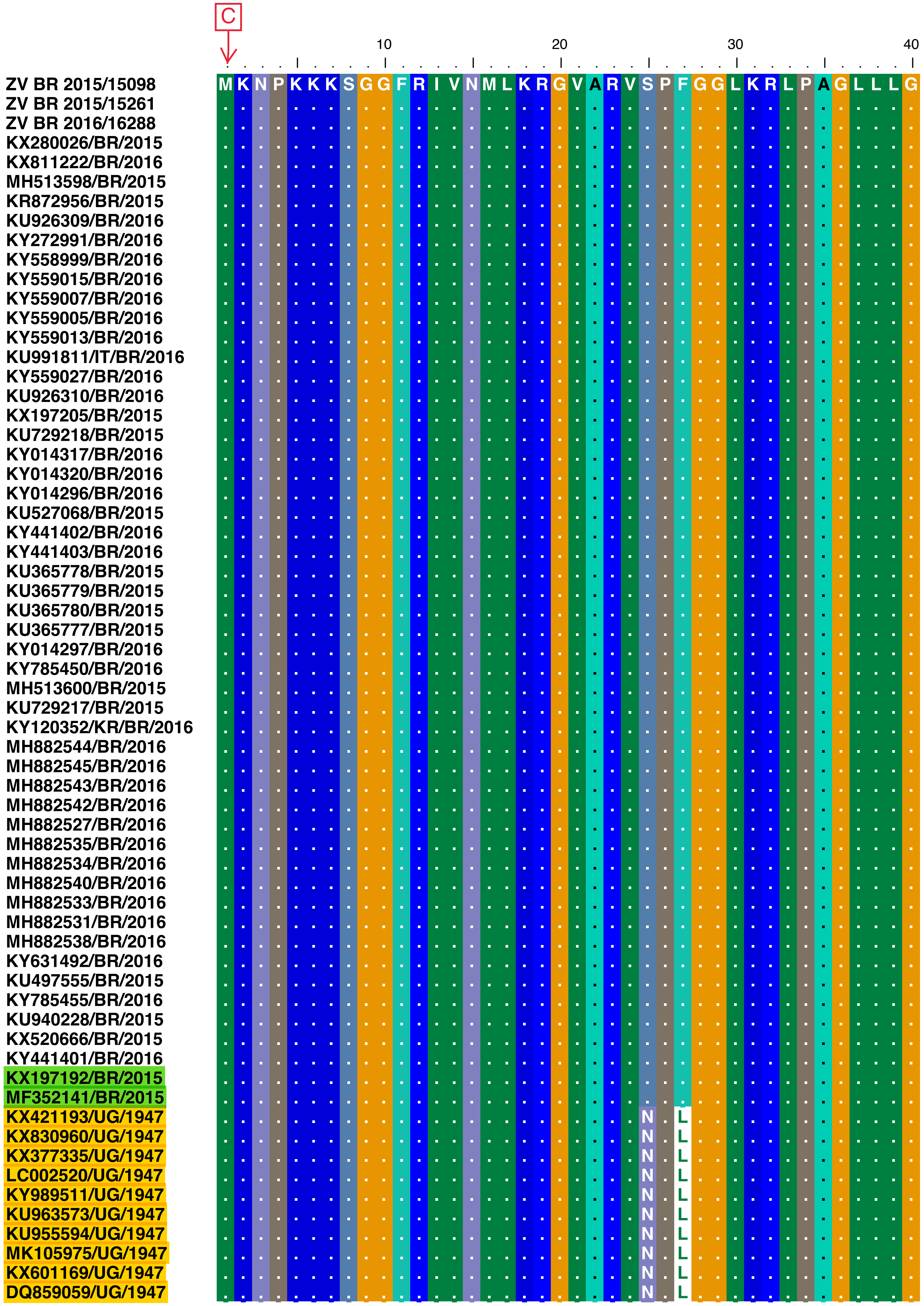
C
10
20
30
40
M K N P K K K S G G F R I V N M L K R G V A R V S P F G G L K R L P A G L L L G
ZV BR 2015/15098
ZV BR 2015/15261
ZV BR 2016/16288
KX280026/BR/2015
KX811222/BR/2016
MH513598/BR/2015
KR872956/BR/2015
KU926309/BR/2016
KY272991/BR/2016
KY558999/BR/2016
KY559015/BR/2016
KY559007/BR/2016
KY559005/BR/2016
KY559013/BR/2016
KU991811/IT/BR/2016
KY559027/BR/2016
KU926310/BR/2016
KX197205/BR/2015
KU729218/BR/2015
KY014317/BR/2016
KY014320/BR/2016
KY014296/BR/2016
KU527068/BR/2015
KY441402/BR/2016
KY441403/BR/2016
KU365778/BR/2015
KU365779/BR/2015
KU365780/BR/2015
KU365777/BR/2015
KY014297/BR/2016
KY785450/BR/2016
MH513600/BR/2015
KU729217/BR/2015
KY120352/KR/BR/2016
MH882544/BR/2016
MH882545/BR/2016
MH882543/BR/2016
MH882542/BR/2016
MH882527/BR/2016
MH882535/BR/2016
MH882534/BR/2016
MH882540/BR/2016
MH882533/BR/2016
MH882531/BR/2016
MH882538/BR/2016
KY631492/BR/2016
KU497555/BR/2015
KY785455/BR/2016
KU940228/BR/2015
KX520666/BR/2015
KY441401/BR/2016
KX197192/BR/2015
MF352141/BR/2015
KX421193/UG/1947
KX830960/UG/1947
KX377335/UG/1947
LC002520/UG/1947
KY989511/UG/1947
KU963573/UG/1947
KU955594/UG/1947
MK105975/UG/1947
KX601169/UG/1947
DQ859059/UG/1947
N L

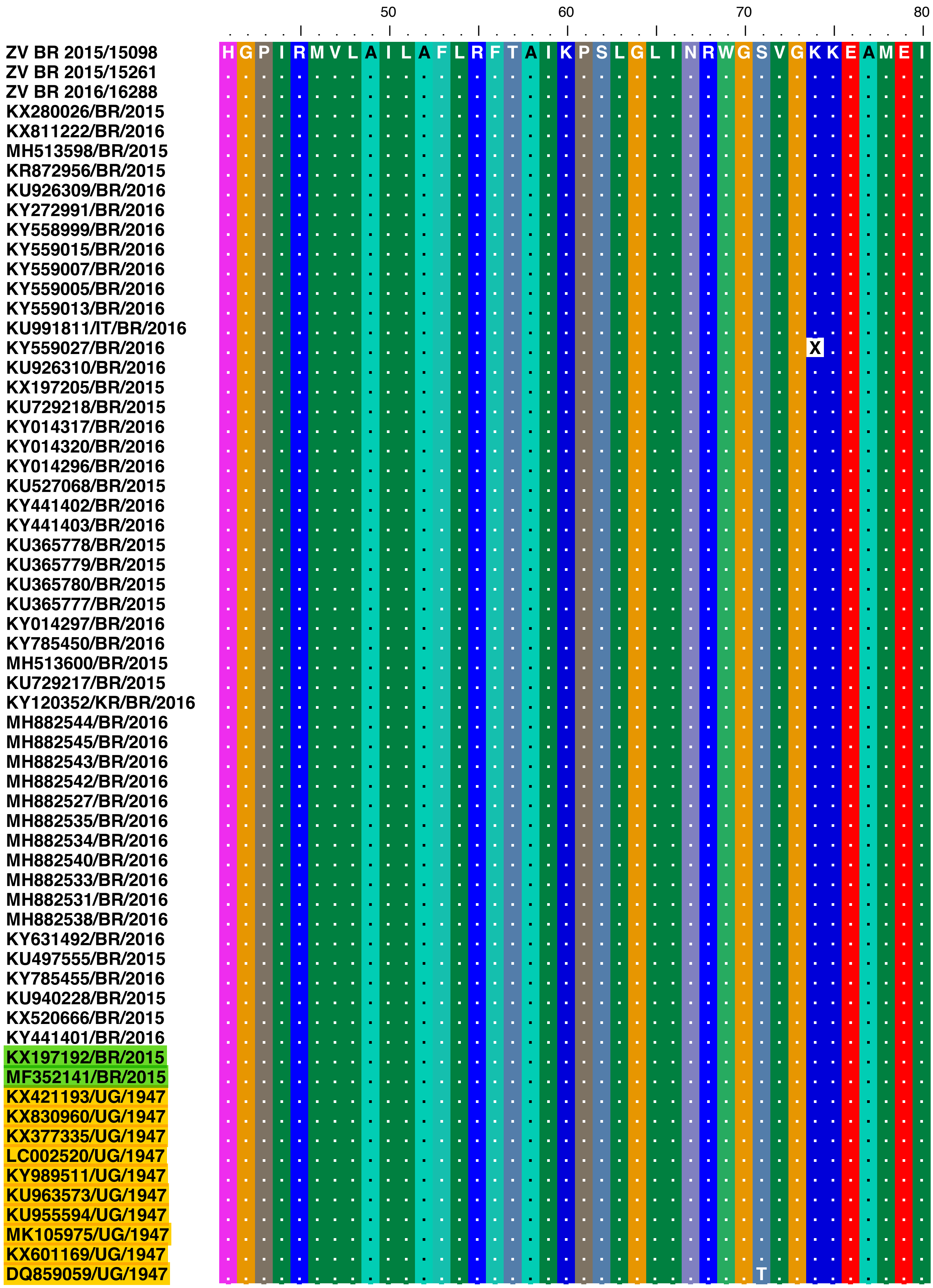
50
60
70
80
H G P I R M V L A I L A F L R F T A I K P S L G L I N R W G S V G K K E A M E I
ZV BR 2015/15098
ZV BR 2015/15261
ZV BR 2016/16288
KX280026/BR/2015
KX811222/BR/2016
MH513598/BR/2015
KR872956/BR/2015
KU926309/BR/2016
KY272991/BR/2016
KY558999/BR/2016
KY559015/BR/2016
KY559007/BR/2016
KY559005/BR/2016
KY559013/BR/2016
KU991811/IT/BR/2016
KY559027/BR/2016
X
KU926310/BR/2016
KX197205/BR/2015
KU729218/BR/2015
KY014317/BR/2016
KY014320/BR/2016
KY014296/BR/2016
KU527068/BR/2015
KY441402/BR/2016
KY441403/BR/2016
KU365778/BR/2015
KU365779/BR/2015
KU365780/BR/2015
KU365777/BR/2015
KY014297/BR/2016
KY785450/BR/2016
MH513600/BR/2015
KU729217/BR/2015
KY120352/KR/BR/2016
MH882544/BR/2016
MH882545/BR/2016
MH882543/BR/2016
MH882542/BR/2016
MH882527/BR/2016
MH882535/BR/2016
MH882534/BR/2016
MH882540/BR/2016
MH882533/BR/2016
MH882531/BR/2016
MH882538/BR/2016
KY631492/BR/2016
KU497555/BR/2015
KY785455/BR/2016
KU940228/BR/2015
KX520666/BR/2015
KY441401/BR/2016
KX197192/BR/2015
MF352141/BR/2015
KX421193/UG/1947
KX830960/UG/1947
KX377335/UG/1947
LC002520/UG/1947
KY989511/UG/1947
KU963573/UG/1947
KU955594/UG/1947
MK105975/UG/1947
KX601169/UG/1947
DQ859059/UG/1947
T

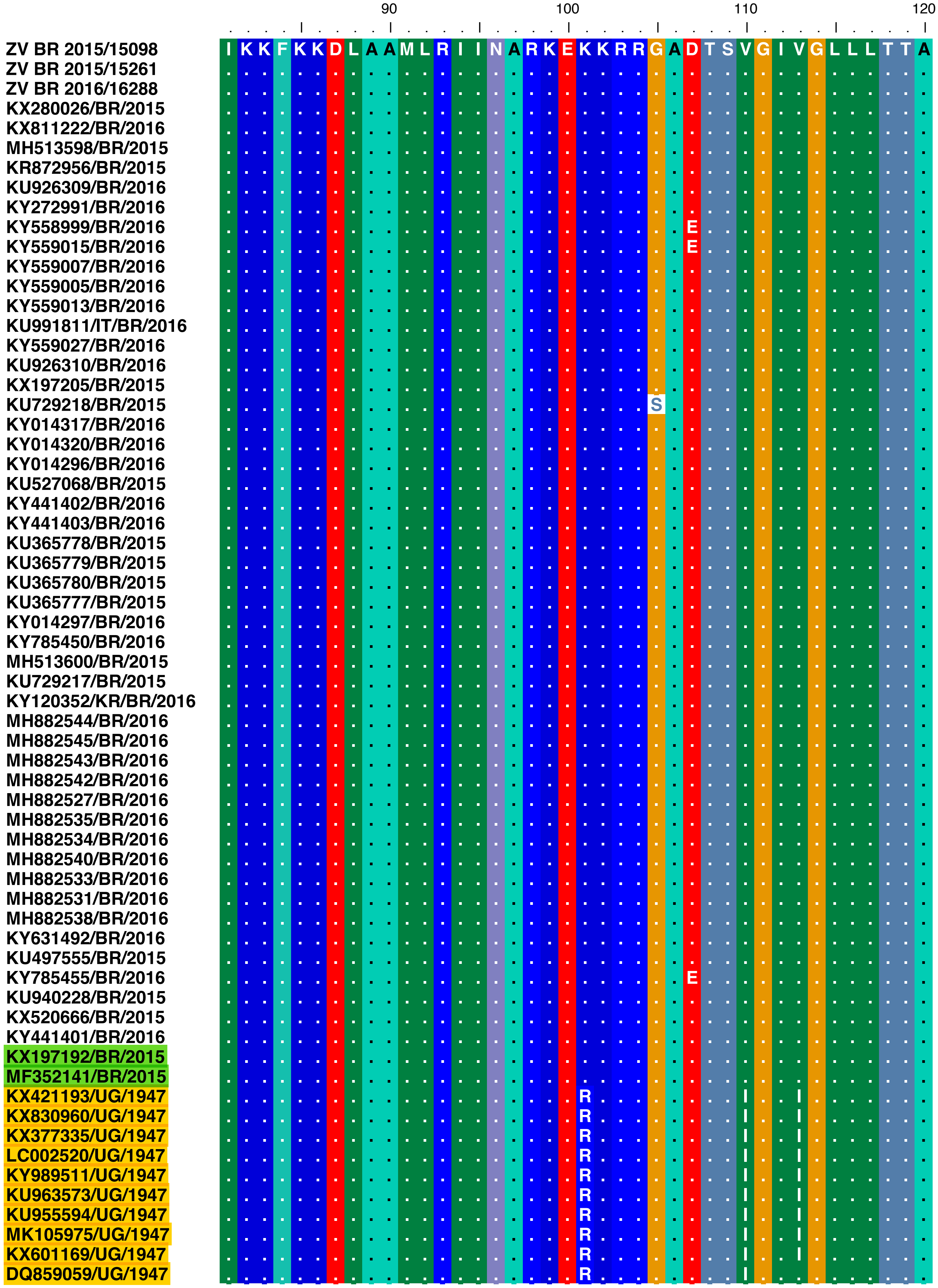
90
100
110
120
I K K F K K D L A A M L R I I N A R K E K K R R G A D T S V G I V G L L L T T A
ZV BR 2015/15098
ZV BR 2015/15261
ZV BR 2016/16288
KX280026/BR/2015
KX811222/BR/2016
MH513598/BR/2015
KR872956/BR/2015
KU926309/BR/2016
KY272991/BR/2016
KY558999/BR/2016
KY559015/BR/2016
KY559007/BR/2016
KY559005/BR/2016
KY559013/BR/2016
KU991811/IT/BR/2016
KY559027/BR/2016
KU926310/BR/2016
KX197205/BR/2015
KU729218/BR/2015
KY014317/BR/2016
KY014320/BR/2016
KY014296/BR/2016
KU527068/BR/2015
KY441402/BR/2016
KY441403/BR/2016
KU365778/BR/2015
KU365779/BR/2015
KU365780/BR/2015
KU365777/BR/2015
KY014297/BR/2016
KY785450/BR/2016
MH513600/BR/2015
KU729217/BR/2015
KY120352/KR/BR/2016
MH882544/BR/2016
MH882545/BR/2016
MH882543/BR/2016
MH882542/BR/2016
MH882527/BR/2016
MH882535/BR/2016
MH882534/BR/2016
MH882540/BR/2016
MH882533/BR/2016
MH882531/BR/2016
MH882538/BR/2016
KY631492/BR/2016
KU497555/BR/2015
KY785455/BR/2016
KU940228/BR/2015
KX520666/BR/2015
KY441401/BR/2016
KX197192/BR/2015
MF352141/BR/2015
KX421193/UG/1947
KX830960/UG/1947
KX377335/UG/1947
LC002520/UG/1947
KY989511/UG/1947
KU963573/UG/1947
KU955594/UG/1947
MK105975/UG/1947
KX601169/UG/1947
DQ859059/UG/1947
E
E
S
E
R
I
I

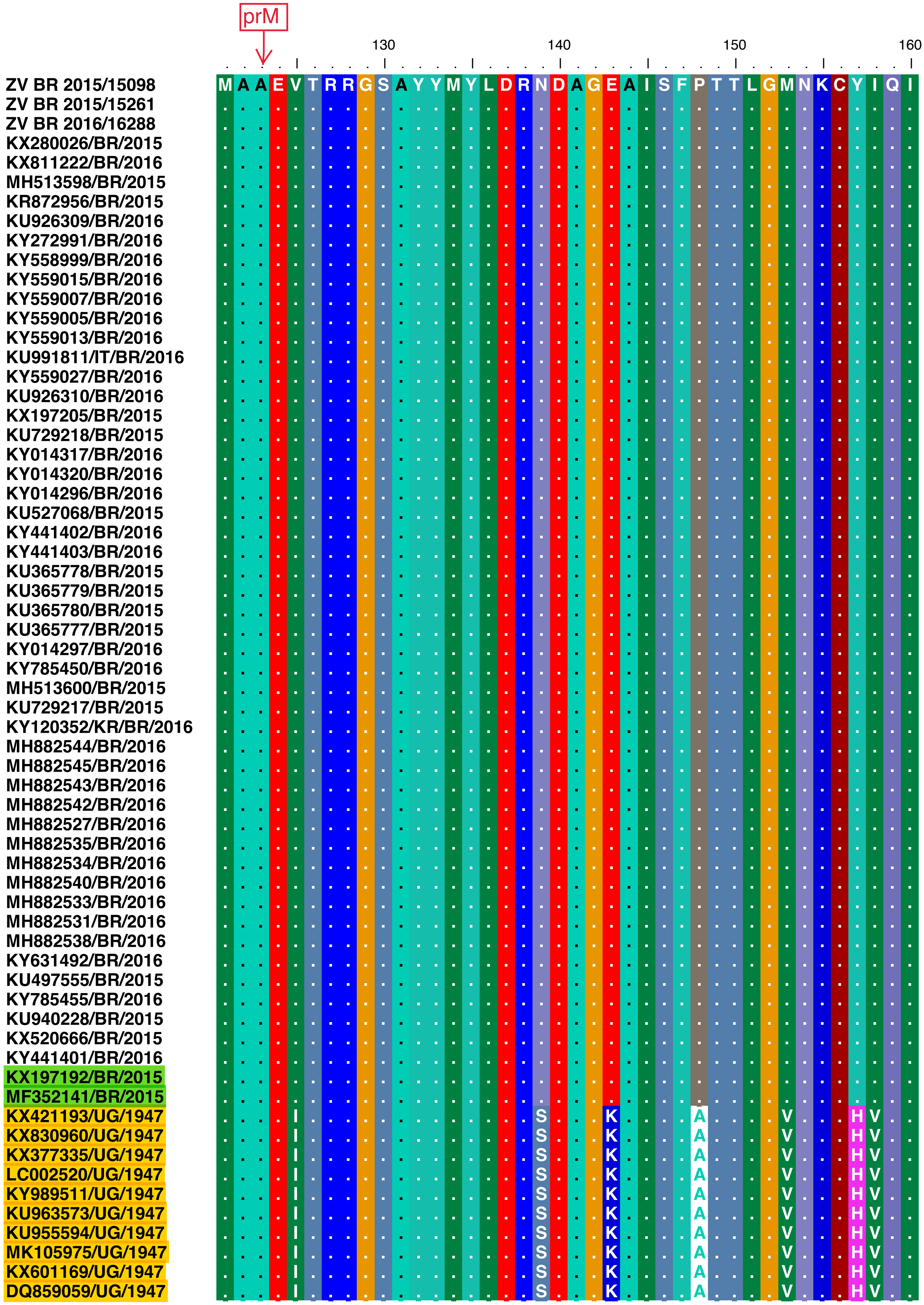

prM
130
140
150
160
MAAEVTRRGSAYYMYLDRNDAGEAISFPTTLGMNKCYIQI
ZV BR 2015/15098
ZV BR 2015/15261
ZV BR 2016/16288
KX280026/BR/2015
KX811222/BR/2016
MH513598/BR/2015
KR872956/BR/2015
KU926309/BR/2016
KY272991/BR/2016
KY558999/BR/2016
KY559015/BR/2016
KY559007/BR/2016
KY559005/BR/2016
KY559013/BR/2016
KU991811/IT/BR/2016
KY559027/BR/2016
KU926310/BR/2016
KX197205/BR/2015
KU729218/BR/2015
KY014317/BR/2016
KY014320/BR/2016
KY014296/BR/2016
KU527068/BR/2015
KY441402/BR/2016
KY441403/BR/2016
KU365778/BR/2015
KU365779/BR/2015
KU365780/BR/2015
KU365777/BR/2015
KY014297/BR/2016
KY785450/BR/2016
MH513600/BR/2015
KU729217/BR/2015
KY120352/KR/BR/2016
MH882544/BR/2016
MH882545/BR/2016
MH882543/BR/2016
MH882542/BR/2016
MH882527/BR/2016
MH882535/BR/2016
MH882534/BR/2016
MH882540/BR/2016
MH882533/BR/2016
MH882531/BR/2016
MH882538/BR/2016
KY631492/BR/2016
KU497555/BR/2015
KY785455/BR/2016
KU940228/BR/2015
KX520666/BR/2015
KY441401/BR/2016
KX197192/BR/2015
MF352141/BR/2015
KX421193/UG/1947
KX830960/UG/1947
KX377335/UG/1947
LC002520/UG/1947
KY989511/UG/1947
KU963573/UG/1947
KU955594/UG/1947
MK105975/UG/1947
KX601169/UG/1947
DQ859059/UG/1947
I S K A V H V

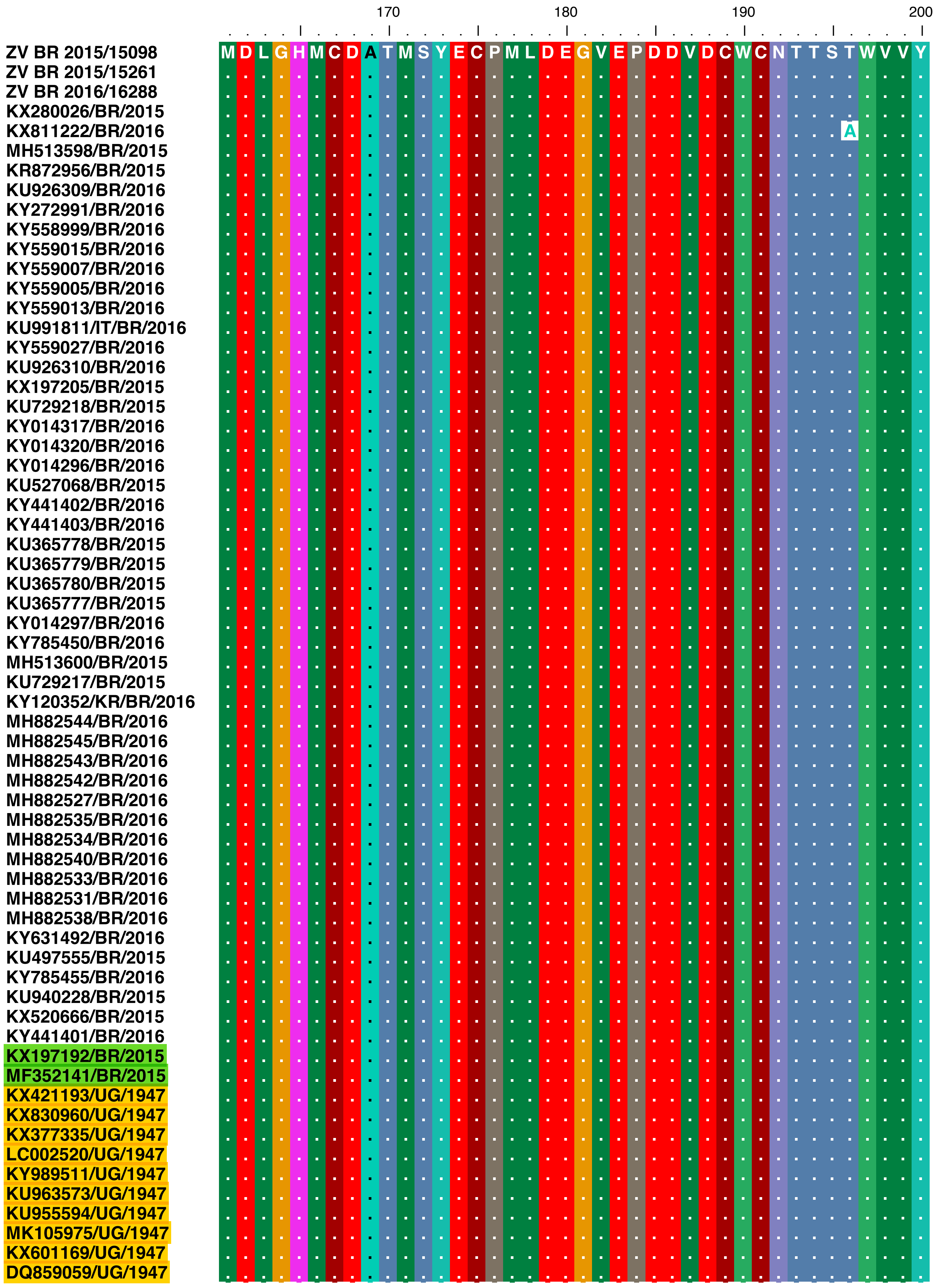

170
180
190
200
M D L G H M C D A T M S Y E C P M L D E G V E P D D V D C W C N T T S T W V V Y
A
ZV BR 2015/15098
ZV BR 2015/15261
ZV BR 2016/16288
KX280026/BR/2015
KX811222/BR/2016
MH513598/BR/2015
KR872956/BR/2015
KU926309/BR/2016
KY272991/BR/2016
KY558999/BR/2016
KY559015/BR/2016
KY559007/BR/2016
KY559005/BR/2016
KY559013/BR/2016
KU991811/IT/BR/2016
KY559027/BR/2016
KU926310/BR/2016
KX197205/BR/2015
KU729218/BR/2015
KY014317/BR/2016
KY014320/BR/2016
KY014296/BR/2016
KU527068/BR/2015
KY441402/BR/2016
KY441403/BR/2016
KU365778/BR/2015
KU365779/BR/2015
KU365780/BR/2015
KU365777/BR/2015
KY014297/BR/2016
KY785450/BR/2016
MH513600/BR/2015
KU729217/BR/2015
KY120352/KR/BR/2016
MH882544/BR/2016
MH882545/BR/2016
MH882543/BR/2016
MH882542/BR/2016
MH882527/BR/2016
MH882535/BR/2016
MH882534/BR/2016
MH882540/BR/2016
MH882533/BR/2016
MH882531/BR/2016
MH882538/BR/2016
KY631492/BR/2016
KU497555/BR/2015
KY785455/BR/2016
KU940228/BR/2015
KX520666/BR/2015
KY441401/BR/2016
KX197192/BR/2015
MF352141/BR/2015
KX421193/UG/1947
KX830960/UG/1947
KX377335/UG/1947
LC002520/UG/1947
KY989511/UG/1947
KU963573/UG/1947
KU955594/UG/1947
MK105975/UG/1947
KX601169/UG/1947
DQ859059/UG/1947

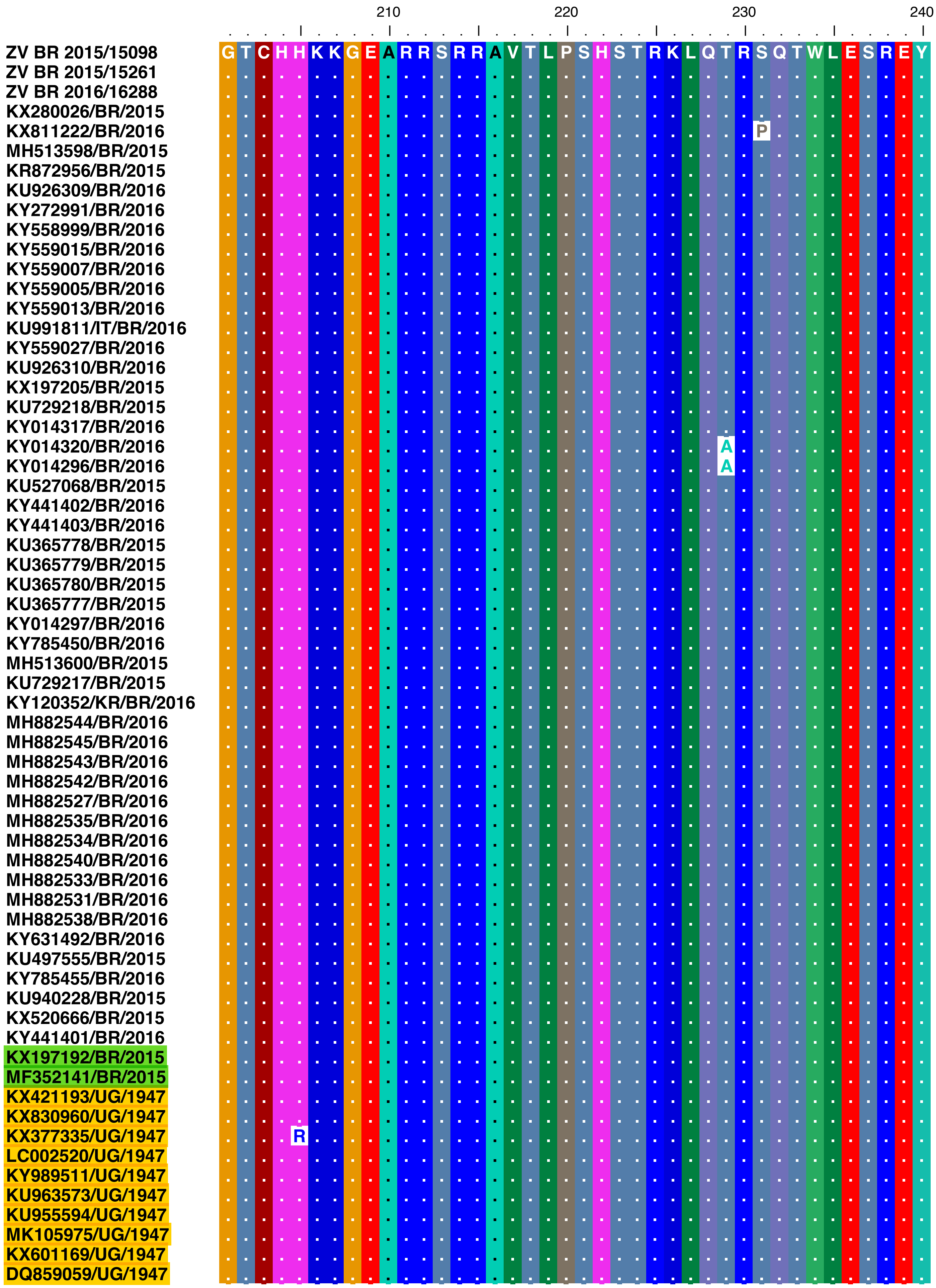

210
220
230
240
G T C H H K K G E A R R S R R A V T L P S H S T R K L Q T R S Q T W L E S R E Y
ZV BR 2015/15098
ZV BR 2015/15261
ZV BR 2016/16288
KX280026/BR/2015
KX811222/BR/2016
P
MH513598/BR/2015
KR872956/BR/2015
KU926309/BR/2016
KY272991/BR/2016
KY558999/BR/2016
KY559015/BR/2016
KY559007/BR/2016
KY559005/BR/2016
KY559013/BR/2016
KU991811/IT/BR/2016
KY559027/BR/2016
KU926310/BR/2016
KX197205/BR/2015
KU729218/BR/2015
KY014317/BR/2016
KY014320/BR/2016
A
KY014296/BR/2016
A
KU527068/BR/2015
KY441402/BR/2016
KY441403/BR/2016
KU365778/BR/2015
KU365779/BR/2015
KU365780/BR/2015
KU365777/BR/2015
KY014297/BR/2016
KY785450/BR/2016
MH513600/BR/2015
KU729217/BR/2015
KY120352/KR/BR/2016
MH882544/BR/2016
MH882545/BR/2016
MH882543/BR/2016
MH882542/BR/2016
MH882527/BR/2016
MH882535/BR/2016
MH882534/BR/2016
MH882540/BR/2016
MH882533/BR/2016
MH882531/BR/2016
MH882538/BR/2016
KY631492/BR/2016
KU497555/BR/2015
KY785455/BR/2016
KU940228/BR/2015
KX520666/BR/2015
KY441401/BR/2016
KX197192/BR/2015
MF352141/BR/2015
KX421193/UG/1947
KX830960/UG/1947
KX377335/UG/1947
R
LC002520/UG/1947
KY989511/UG/1947
KU963573/UG/1947
KU955594/UG/1947
MK105975/UG/1947
KX601169/UG/1947
DQ859059/UG/1947

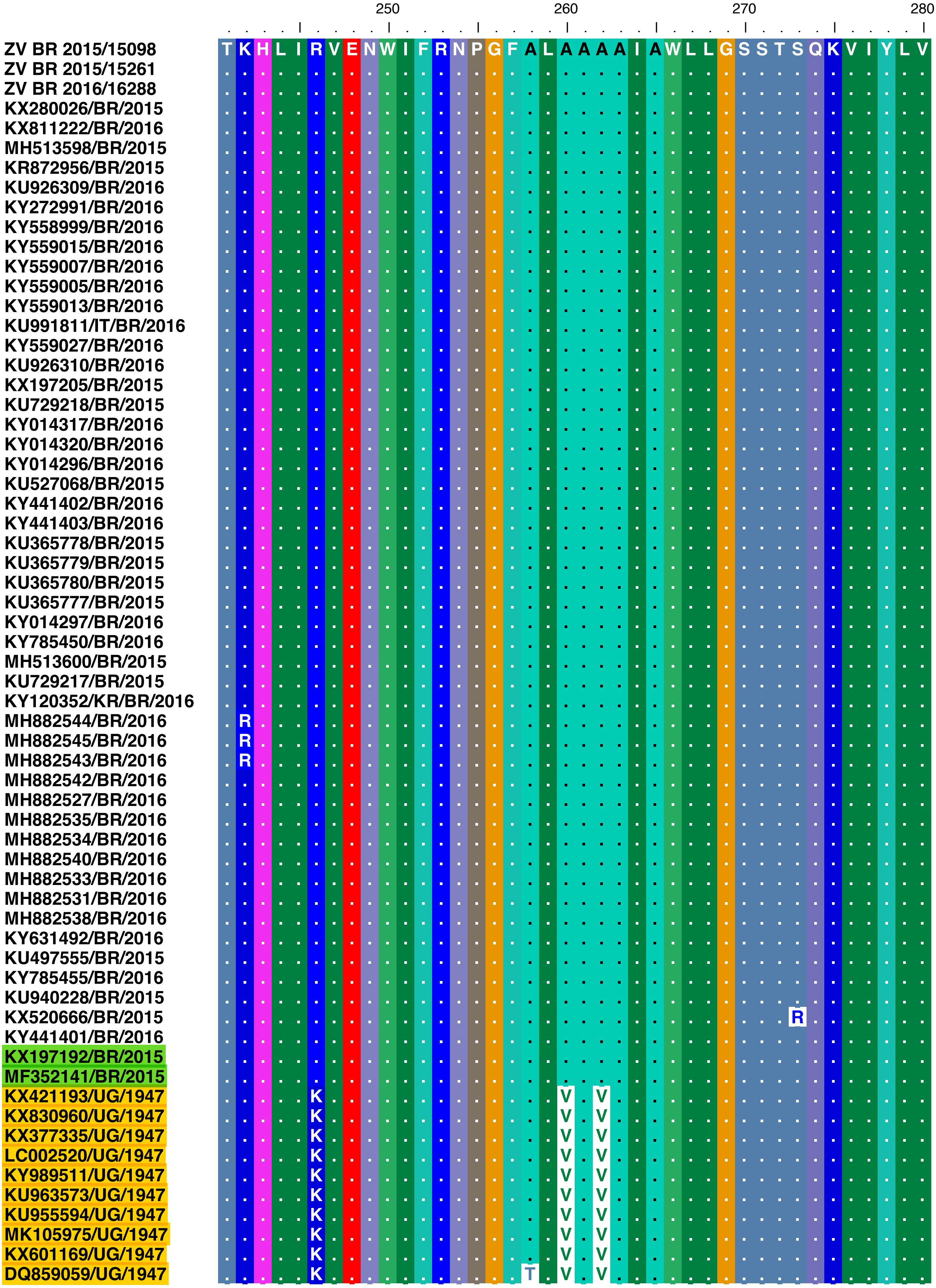
250
260
270
280
T K H L I R V E N W I F R N P G F A L A A A A I A W L L G S S T S Q K V I Y L V
ZV BR 2015/15098
ZV BR 2015/15261
ZV BR 2016/16288
KX280026/BR/2015
KX811222/BR/2016
MH513598/BR/2015
KR872956/BR/2015
KU926309/BR/2016
KY272991/BR/2016
KY558999/BR/2016
KY559015/BR/2016
KY559007/BR/2016
KY559005/BR/2016
KY559013/BR/2016
KU991811/IT/BR/2016
KY559027/BR/2016
KU926310/BR/2016
KX197205/BR/2015
KU729218/BR/2015
KY014317/BR/2016
KY014320/BR/2016
KY014296/BR/2016
KU527068/BR/2015
KY441402/BR/2016
KY441403/BR/2016
KU365778/BR/2015
KU365779/BR/2015
KU365780/BR/2015
KU365777/BR/2015
KY014297/BR/2016
KY785450/BR/2016
MH513600/BR/2015
KU729217/BR/2015
KY120352/KR/BR/2016
MH882544/BR/2016
MH882545/BR/2016
MH882543/BR/2016
MH882542/BR/2016
MH882527/BR/2016
MH882535/BR/2016
MH882534/BR/2016
MH882540/BR/2016
MH882533/BR/2016
MH882531/BR/2016
MH882538/BR/2016
KY631492/BR/2016
KU497555/BR/2015
KY785455/BR/2016
KU940228/BR/2015
KX520666/BR/2015
KY441401/BR/2016
KX197192/BR/2015
MF352141/BR/2015
KX421193/UG/1947
KX830960/UG/1947
KX377335/UG/1947
LC002520/UG/1947
KY989511/UG/1947
KU963573/UG/1947
KU955594/UG/1947
MK105975/UG/1947
KX601169/UG/1947
DQ859059/UG/1947

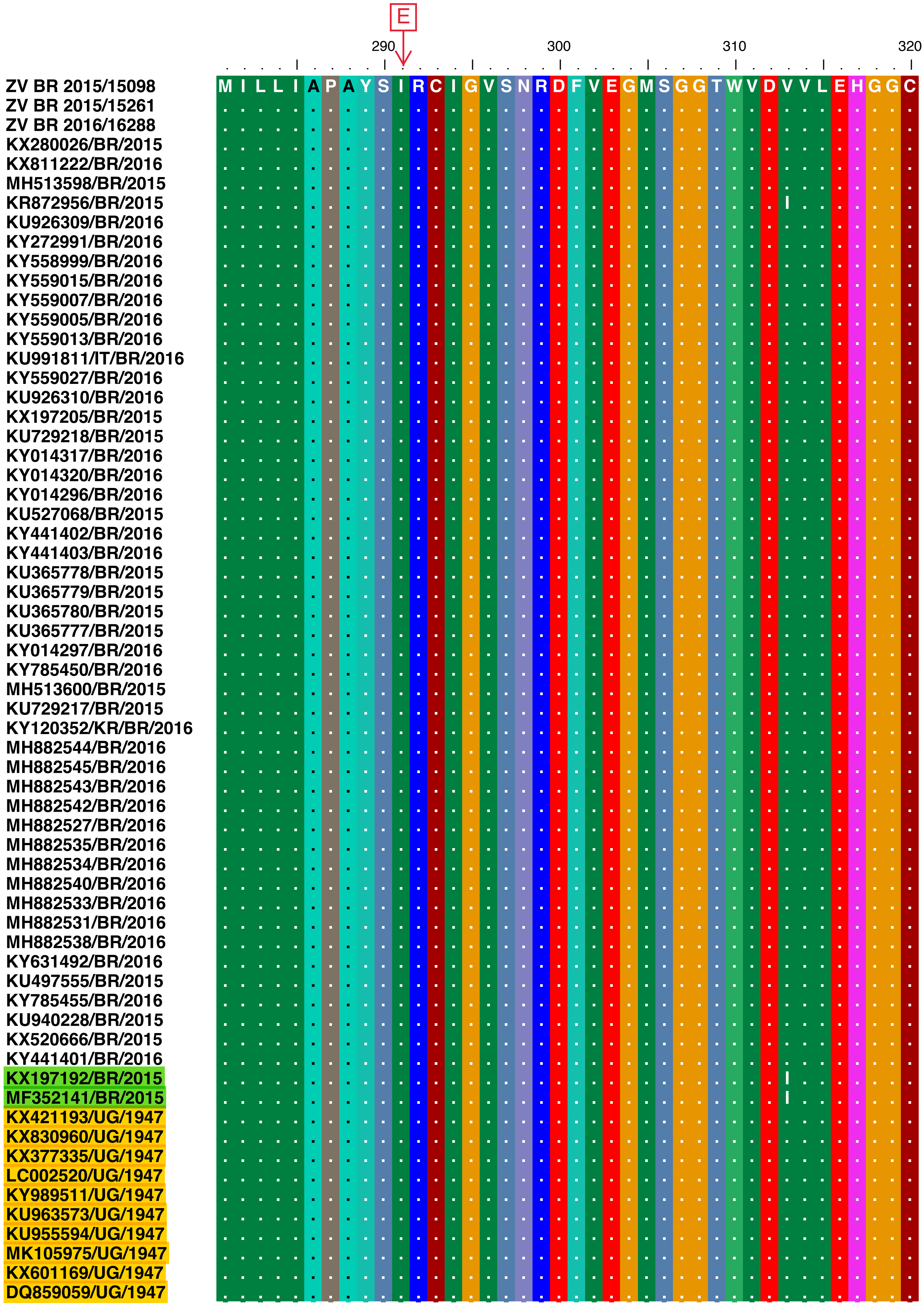
E
290
300
310
320
M I L L I A P A Y S I R C I G V S N R D F V E G M S G G T W V D V V L E H G G C
ZV BR 2015/15098
ZV BR 2015/15261
ZV BR 2016/16288
KX280026/BR/2015
KX811222/BR/2016
MH513598/BR/2015
KR872956/BR/2015
KU926309/BR/2016
KY272991/BR/2016
KY558999/BR/2016
KY559015/BR/2016
KY559007/BR/2016
KY559005/BR/2016
KY559013/BR/2016
KU991811/IT/BR/2016
KY559027/BR/2016
KU926310/BR/2016
KX197205/BR/2015
KU729218/BR/2015
KY014317/BR/2016
KY014320/BR/2016
KY014296/BR/2016
KU527068/BR/2015
KY441402/BR/2016
KY441403/BR/2016
KU365778/BR/2015
KU365779/BR/2015
KU365780/BR/2015
KU365777/BR/2015
KY014297/BR/2016
KY785450/BR/2016
MH513600/BR/2015
KU729217/BR/2015
KY120352/KR/BR/2016
MH882544/BR/2016
MH882545/BR/2016
MH882543/BR/2016
MH882542/BR/2016
MH882527/BR/2016
MH882535/BR/2016
MH882534/BR/2016
MH882540/BR/2016
MH882533/BR/2016
MH882531/BR/2016
MH882538/BR/2016
KY631492/BR/2016
KU497555/BR/2015
KY785455/BR/2016
KU940228/BR/2015
KX520666/BR/2015
KY441401/BR/2016
KX197192/BR/2015
MF352141/BR/2015
KX421193/UG/1947
KX830960/UG/1947
KX377335/UG/1947
LC002520/UG/1947
KY989511/UG/1947
KU963573/UG/1947
KU955594/UG/1947
MK105975/UG/1947
KX601169/UG/1947
DQ859059/UG/1947

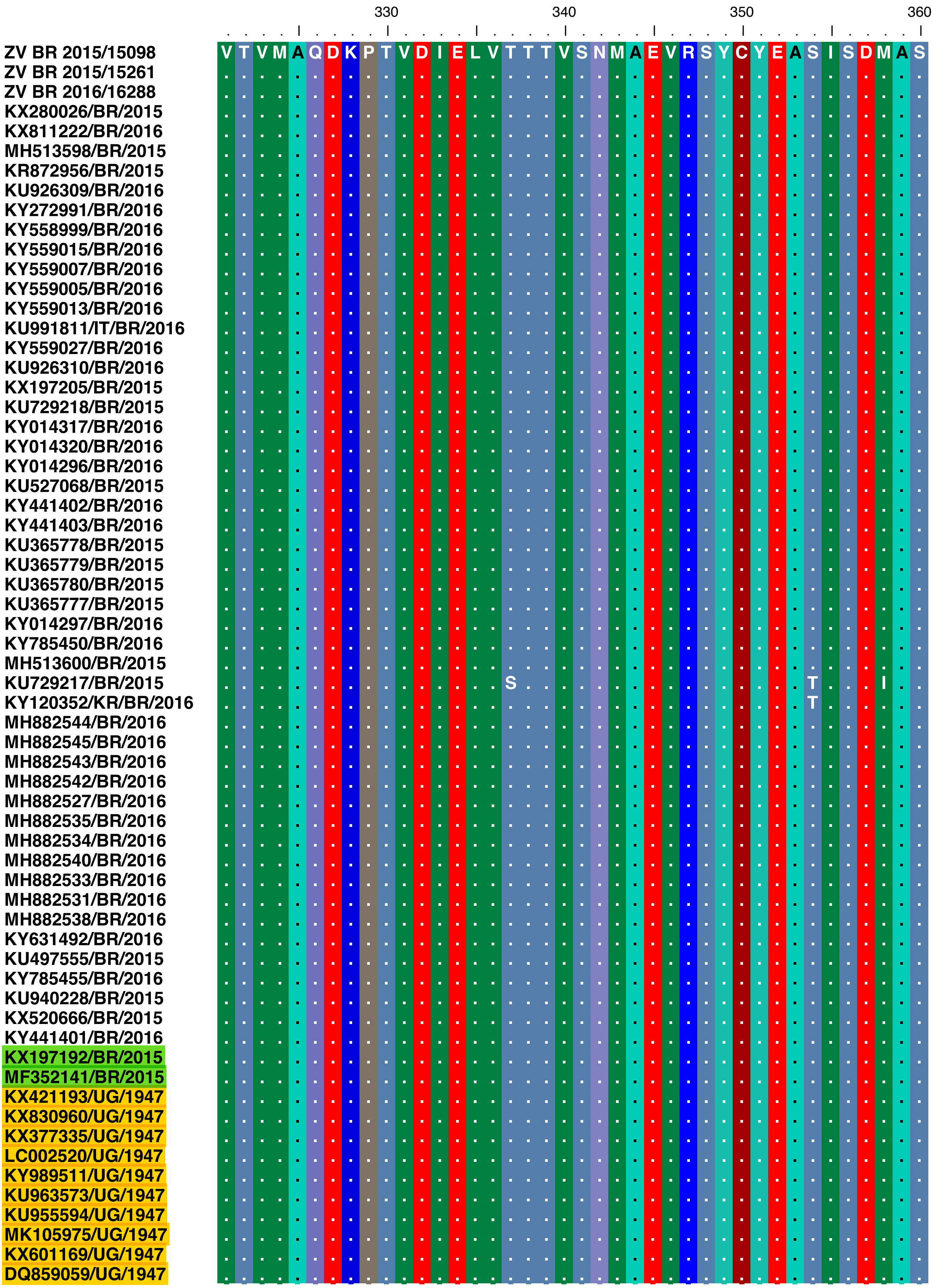

330
340
350
360
V T V M A Q D K P T V D I E L V T T T V S N M A E V R S Y C Y E A S I S D M A S
ZV BR 2015/15098
ZV BR 2015/15261
ZV BR 2016/16288
KX280026/BR/2015
KX811222/BR/2016
MH513598/BR/2015
KR872956/BR/2015
KU926309/BR/2016
KY272991/BR/2016
KY558999/BR/2016
KY559015/BR/2016
KY559007/BR/2016
KY559005/BR/2016
KY559013/BR/2016
KU991811/IT/BR/2016
KY559027/BR/2016
KU926310/BR/2016
KX197205/BR/2015
KU729218/BR/2015
KY014317/BR/2016
KY014320/BR/2016
KY014296/BR/2016
KU527068/BR/2015
KY441402/BR/2016
KY441403/BR/2016
KU365778/BR/2015
KU365779/BR/2015
KU365780/BR/2015
KU365777/BR/2015
KY014297/BR/2016
KY785450/BR/2016
MH513600/BR/2015
KU729217/BR/2015
S
T
I
KY120352/KR/BR/2016
T
MH882544/BR/2016
MH882545/BR/2016
MH882543/BR/2016
MH882542/BR/2016
MH882527/BR/2016
MH882535/BR/2016
MH882534/BR/2016
MH882540/BR/2016
MH882533/BR/2016
MH882531/BR/2016
MH882538/BR/2016
KY631492/BR/2016
KU497555/BR/2015
KY785455/BR/2016
KU940228/BR/2015
KX520666/BR/2015
KY441401/BR/2016
KX197192/BR/2015
MF352141/BR/2015
KX421193/UG/1947
KX830960/UG/1947
KX377335/UG/1947
LC002520/UG/1947
KY989511/UG/1947
KU963573/UG/1947
KU955594/UG/1947
MK105975/UG/1947
KX601169/UG/1947
DQ859059/UG/1947

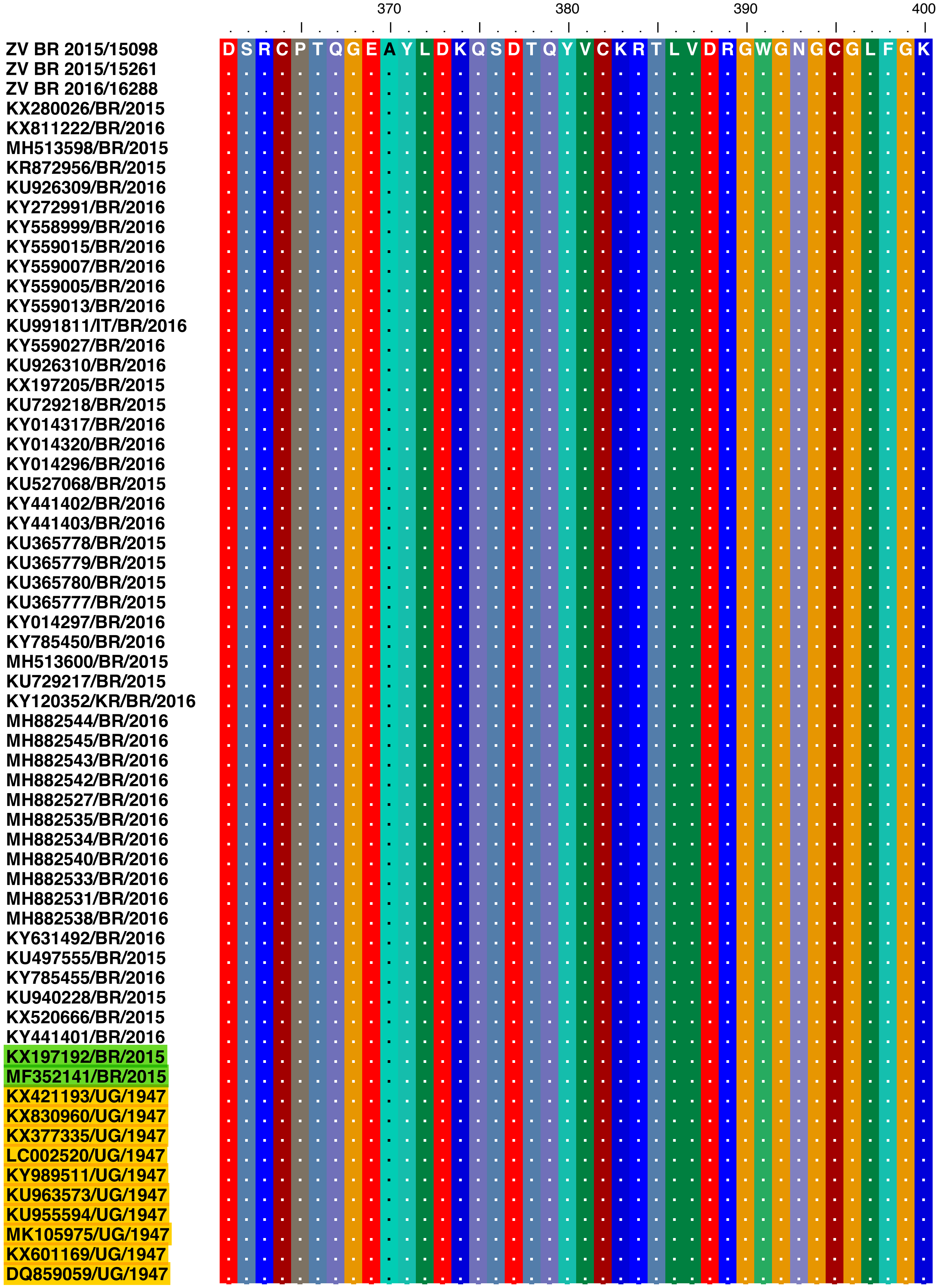

370
380
390
400
D S R C P T Q G E A Y L D K Q S D T Q Y V C K R T L V D R G W G N G C G L F G K
ZV BR 2015/15098
ZV BR 2015/15261
ZV BR 2016/16288
KX280026/BR/2015
KX811222/BR/2016
MH513598/BR/2015
KR872956/BR/2015
KU926309/BR/2016
KY272991/BR/2016
KY558999/BR/2016
KY559015/BR/2016
KY559007/BR/2016
KY559005/BR/2016
KY559013/BR/2016
KU991811/IT/BR/2016
KY559027/BR/2016
KU926310/BR/2016
KX197205/BR/2015
KU729218/BR/2015
KY014317/BR/2016
KY014320/BR/2016
KY014296/BR/2016
KU527068/BR/2015
KY441402/BR/2016
KY441403/BR/2016
KU365778/BR/2015
KU365779/BR/2015
KU365780/BR/2015
KU365777/BR/2015
KY014297/BR/2016
KY785450/BR/2016
MH513600/BR/2015
KU729217/BR/2015
KY120352/KR/BR/2016
MH882544/BR/2016
MH882545/BR/2016
MH882543/BR/2016
MH882542/BR/2016
MH882527/BR/2016
MH882535/BR/2016
MH882534/BR/2016
MH882540/BR/2016
MH882533/BR/2016
MH882531/BR/2016
MH882538/BR/2016
KY631492/BR/2016
KU497555/BR/2015
KY785455/BR/2016
KU940228/BR/2015
KX520666/BR/2015
KY441401/BR/2016
KX197192/BR/2015
MF352141/BR/2015
KX421193/UG/1947
KX830960/UG/1947
KX377335/UG/1947
LC002520/UG/1947
KY989511/UG/1947
KU963573/UG/1947
KU955594/UG/1947
MK105975/UG/1947
KX601169/UG/1947
DQ859059/UG/1947

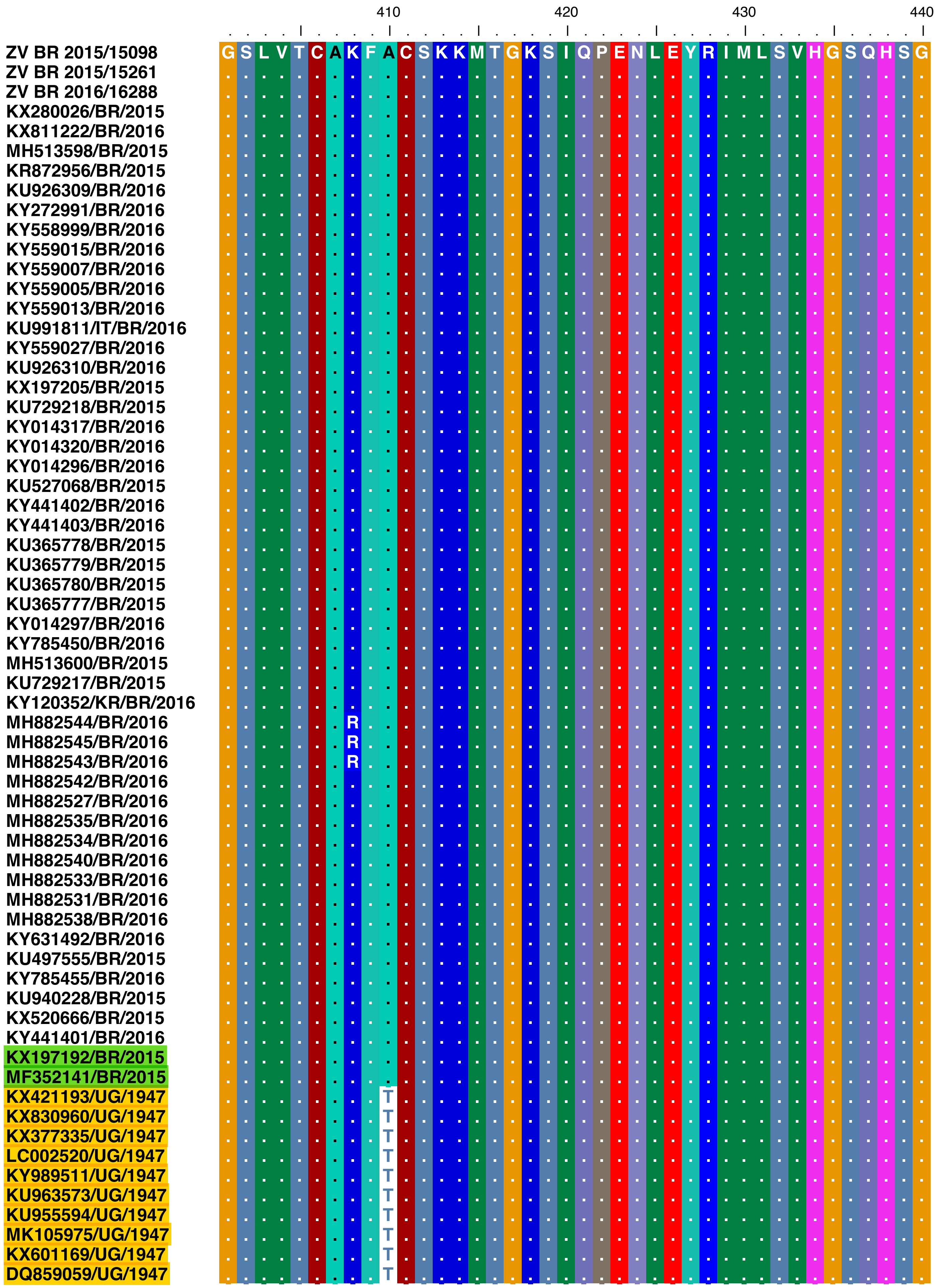
410
420
430
440
G S L V T C A K F A C S K K M T G K S I Q P E N L E Y R I M L S V H G S Q H S G
ZV BR 2015/15098
ZV BR 2015/15261
ZV BR 2016/16288
KX280026/BR/2015
KX811222/BR/2016
MH513598/BR/2015
KR872956/BR/2015
KU926309/BR/2016
KY272991/BR/2016
KY558999/BR/2016
KY559015/BR/2016
KY559007/BR/2016
KY559005/BR/2016
KY559013/BR/2016
KU991811/IT/BR/2016
KY559027/BR/2016
KU926310/BR/2016
KX197205/BR/2015
KU729218/BR/2015
KY014317/BR/2016
KY014320/BR/2016
KY014296/BR/2016
KU527068/BR/2015
KY441402/BR/2016
KY441403/BR/2016
KU365778/BR/2015
KU365779/BR/2015
KU365780/BR/2015
KU365777/BR/2015
KY014297/BR/2016
KY785450/BR/2016
MH513600/BR/2015
KU729217/BR/2015
KY120352/KR/BR/2016
MH882544/BR/2016 R
MH882545/BR/2016 R
MH882543/BR/2016 R
MH882542/BR/2016
MH882527/BR/2016
MH882535/BR/2016
MH882534/BR/2016
MH882540/BR/2016
MH882533/BR/2016
MH882531/BR/2016
MH882538/BR/2016
KY631492/BR/2016
KU497555/BR/2015
KY785455/BR/2016
KU940228/BR/2015
KX520666/BR/2015
KY441401/BR/2016
KX197192/BR/2015
MF352141/BR/2015
KX421193/UG/1947 T
KX830960/UG/1947 T
KX377335/UG/1947 T
LC002520/UG/1947 T
KY989511/UG/1947 T
KU963573/UG/1947 T
KU955594/UG/1947 T
MK105975/UG/1947 T
KX601169/UG/1947 T
DQ859059/UG/1947 T

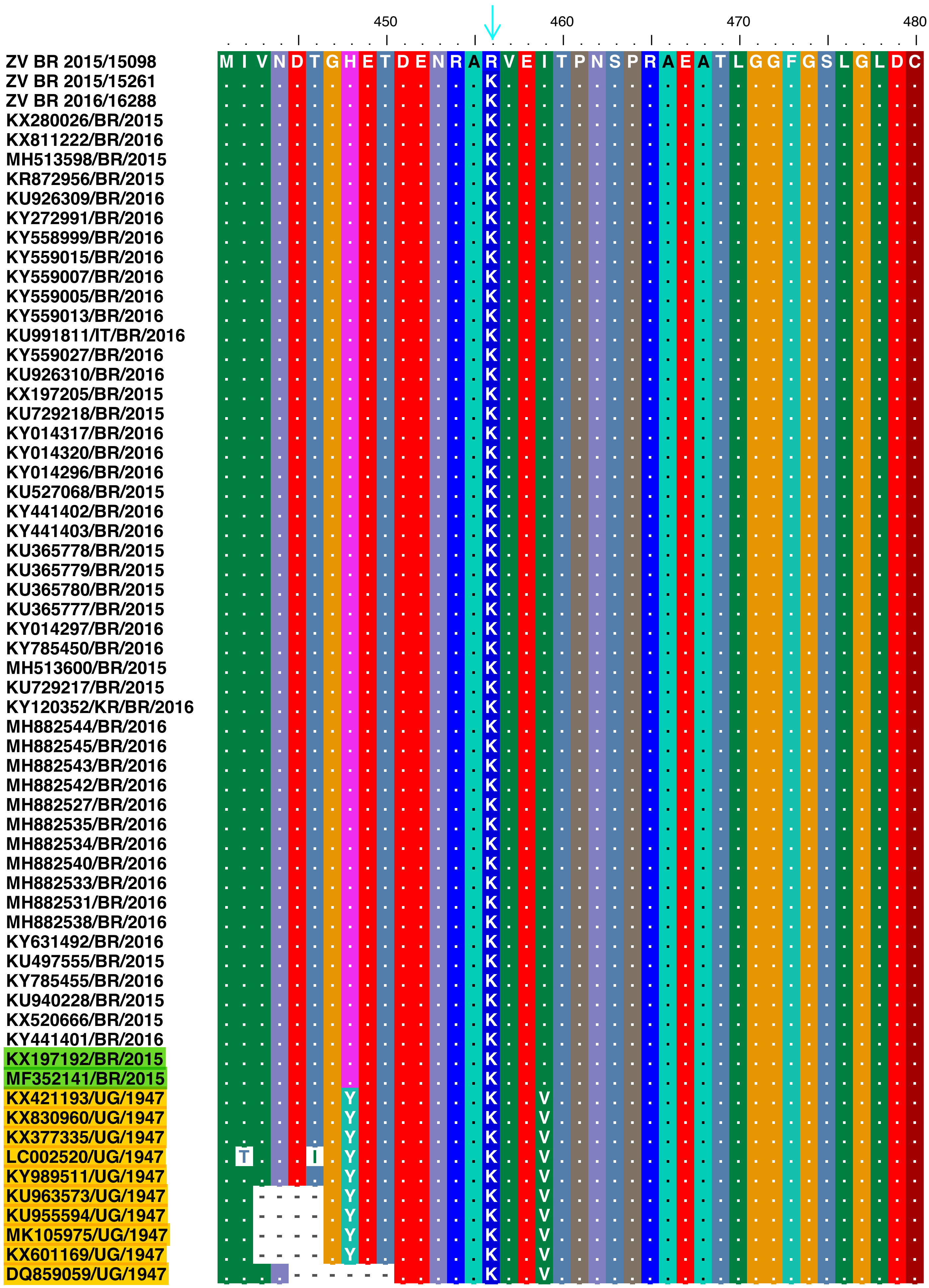
450
460
470
480
ZV BR 2015/15098
M I V N D T G H E T D E N R A R V E I T P N S P R A E A T L G G F G S L G L D C
ZV BR 2015/15261
ZV BR 2016/16288
KX280026/BR/2015
KX811222/BR/2016
MH513598/BR/2015
KR872956/BR/2015
KU926309/BR/2016
KY272991/BR/2016
KY558999/BR/2016
KY559015/BR/2016
KY559007/BR/2016
KY559005/BR/2016
KY559013/BR/2016
KU991811/IT/BR/2016
KY559027/BR/2016
KU926310/BR/2016
KX197205/BR/2015
KU729218/BR/2015
KY014317/BR/2016
KY014320/BR/2016
KY014296/BR/2016
KU527068/BR/2015
KY441402/BR/2016
KY441403/BR/2016
KU365778/BR/2015
KU365779/BR/2015
KU365780/BR/2015
KU365777/BR/2015
KY014297/BR/2016
KY785450/BR/2016
MH513600/BR/2015
KU729217/BR/2015
KY120352/KR/BR/2016
MH882544/BR/2016
MH882545/BR/2016
MH882543/BR/2016
MH882542/BR/2016
MH882527/BR/2016
MH882535/BR/2016
MH882534/BR/2016
MH882540/BR/2016
MH882533/BR/2016
MH882531/BR/2016
MH882538/BR/2016
KY631492/BR/2016
KU497555/BR/2015
KY785455/BR/2016
KU940228/BR/2015
KX520666/BR/2015
KY441401/BR/2016
KX197192/BR/2015
MF352141/BR/2015
KX421193/UG/1947
KX830960/UG/1947
KX377335/UG/1947
LC002520/UG/1947
KY989511/UG/1947
KU963573/UG/1947
KU955594/UG/1947
MK105975/UG/1947
KX601169/UG/1947
DQ859059/UG/1947

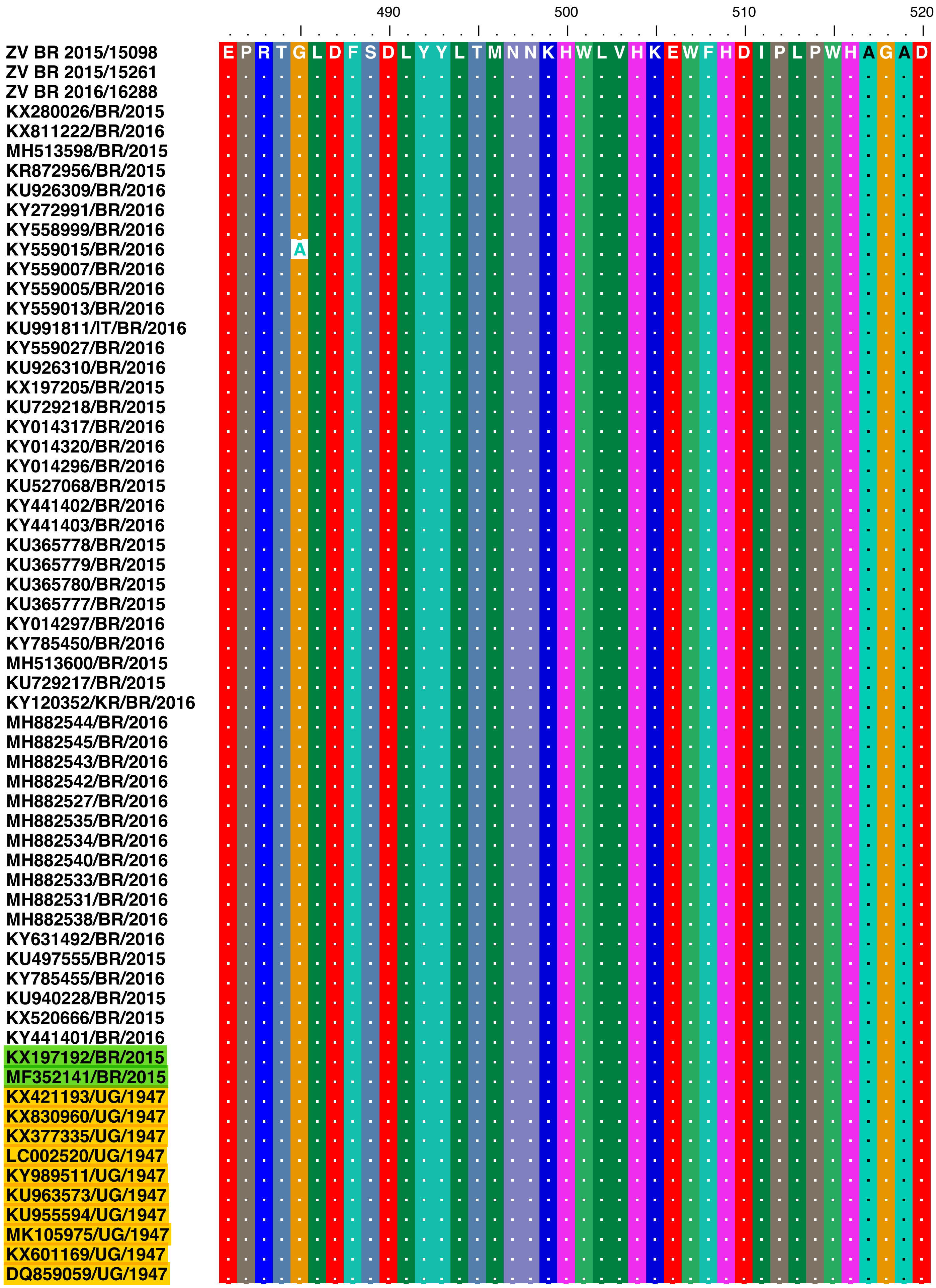
490
500
510
520
E P R T G L D F S D L Y Y L T M N N K H W L V H K E W F H D I P L P W H A G A D
A
ZV BR 2015/15098
ZV BR 2015/15261
ZV BR 2016/16288
KX280026/BR/2015
KX811222/BR/2016
MH513598/BR/2015
KR872956/BR/2015
KU926309/BR/2016
KY272991/BR/2016
KY558999/BR/2016
KY559015/BR/2016
KY559007/BR/2016
KY559005/BR/2016
KY559013/BR/2016
KU991811/IT/BR/2016
KY559027/BR/2016
KU926310/BR/2016
KX197205/BR/2015
KU729218/BR/2015
KY014317/BR/2016
KY014320/BR/2016
KY014296/BR/2016
KU527068/BR/2015
KY441402/BR/2016
KY441403/BR/2016
KU365778/BR/2015
KU365779/BR/2015
KU365780/BR/2015
KU365777/BR/2015
KY014297/BR/2016
KY785450/BR/2016
MH513600/BR/2015
KU729217/BR/2015
KY120352/KR/BR/2016
MH882544/BR/2016
MH882545/BR/2016
MH882543/BR/2016
MH882542/BR/2016
MH882527/BR/2016
MH882535/BR/2016
MH882534/BR/2016
MH882540/BR/2016
MH882533/BR/2016
MH882531/BR/2016
MH882538/BR/2016
KY631492/BR/2016
KU497555/BR/2015
KY785455/BR/2016
KU940228/BR/2015
KX520666/BR/2015
KY441401/BR/2016
KX197192/BR/2015
MF352141/BR/2015
KX421193/UG/1947
KX830960/UG/1947
KX377335/UG/1947
LC002520/UG/1947
KY989511/UG/1947
KU963573/UG/1947
KU955594/UG/1947
MK105975/UG/1947
KX601169/UG/1947
DQ859059/UG/1947

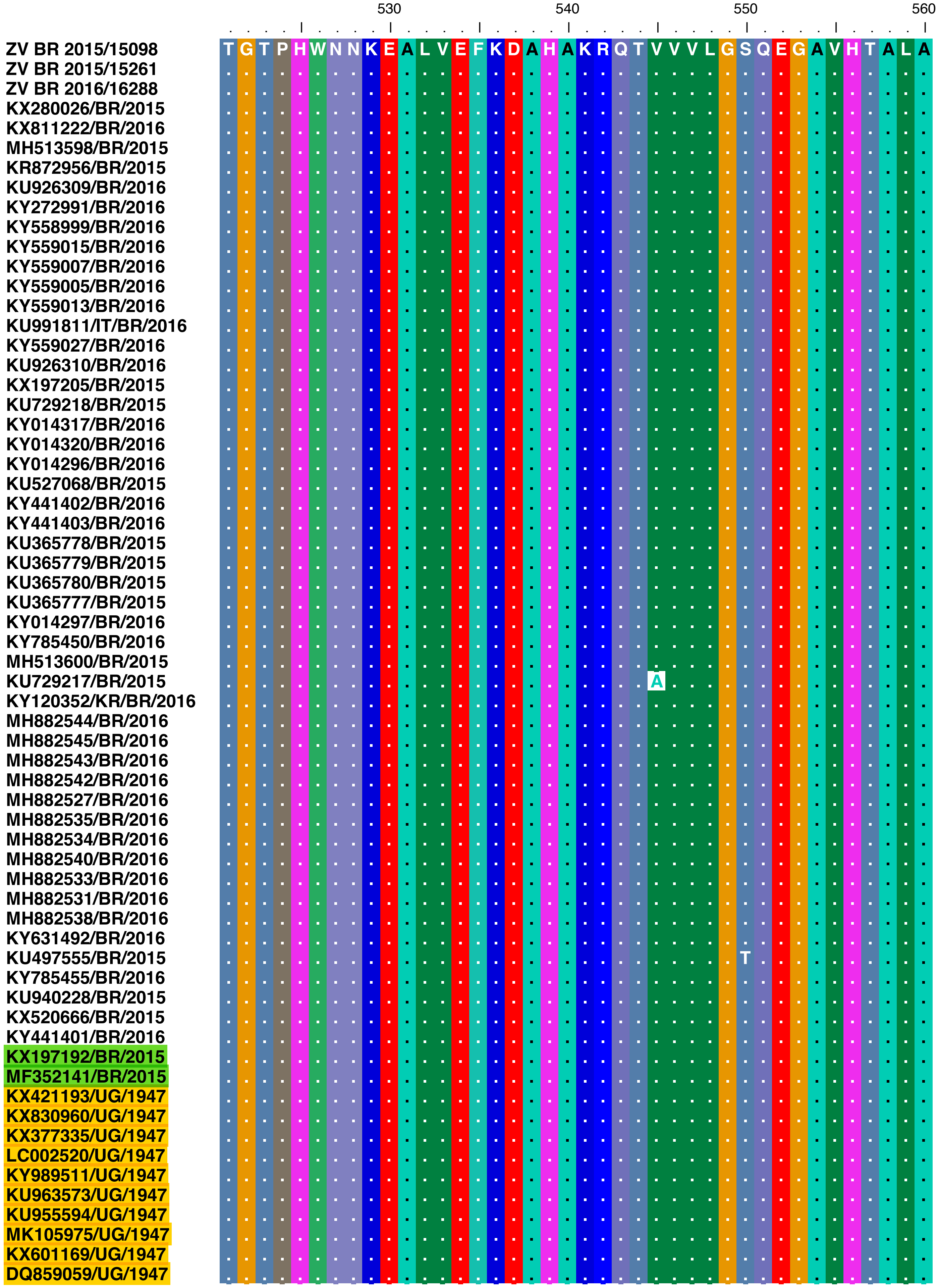

530
540
550
560
T G T P H W N N K E A L V E F K D A H A K R Q T V V V L G S Q E G A V H T A L A
ZV BR 2015/15098
ZV BR 2015/15261
ZV BR 2016/16288
KX280026/BR/2015
KX811222/BR/2016
MH513598/BR/2015
KR872956/BR/2015
KU926309/BR/2016
KY272991/BR/2016
KY558999/BR/2016
KY559015/BR/2016
KY559007/BR/2016
KY559005/BR/2016
KY559013/BR/2016
KU991811/IT/BR/2016
KY559027/BR/2016
KU926310/BR/2016
KX197205/BR/2015
KU729218/BR/2015
KY014317/BR/2016
KY014320/BR/2016
KY014296/BR/2016
KU527068/BR/2015
KY441402/BR/2016
KY441403/BR/2016
KU365778/BR/2015
KU365779/BR/2015
KU365780/BR/2015
KU365777/BR/2015
KY014297/BR/2016
KY785450/BR/2016
MH513600/BR/2015
KU729217/BR/2015
A
KY120352/KR/BR/2016
MH882544/BR/2016
MH882545/BR/2016
MH882543/BR/2016
MH882542/BR/2016
MH882527/BR/2016
MH882535/BR/2016
MH882534/BR/2016
MH882540/BR/2016
MH882533/BR/2016
MH882531/BR/2016
MH882538/BR/2016
KY631492/BR/2016
KU497555/BR/2015
T
KY785455/BR/2016
KU940228/BR/2015
KX520666/BR/2015
KY441401/BR/2016
KX197192/BR/2015
MF352141/BR/2015
KX421193/UG/1947
KX830960/UG/1947
KX377335/UG/1947
LC002520/UG/1947
KY989511/UG/1947
KU963573/UG/1947
KU955594/UG/1947
MK105975/UG/1947
KX601169/UG/1947
DQ859059/UG/1947

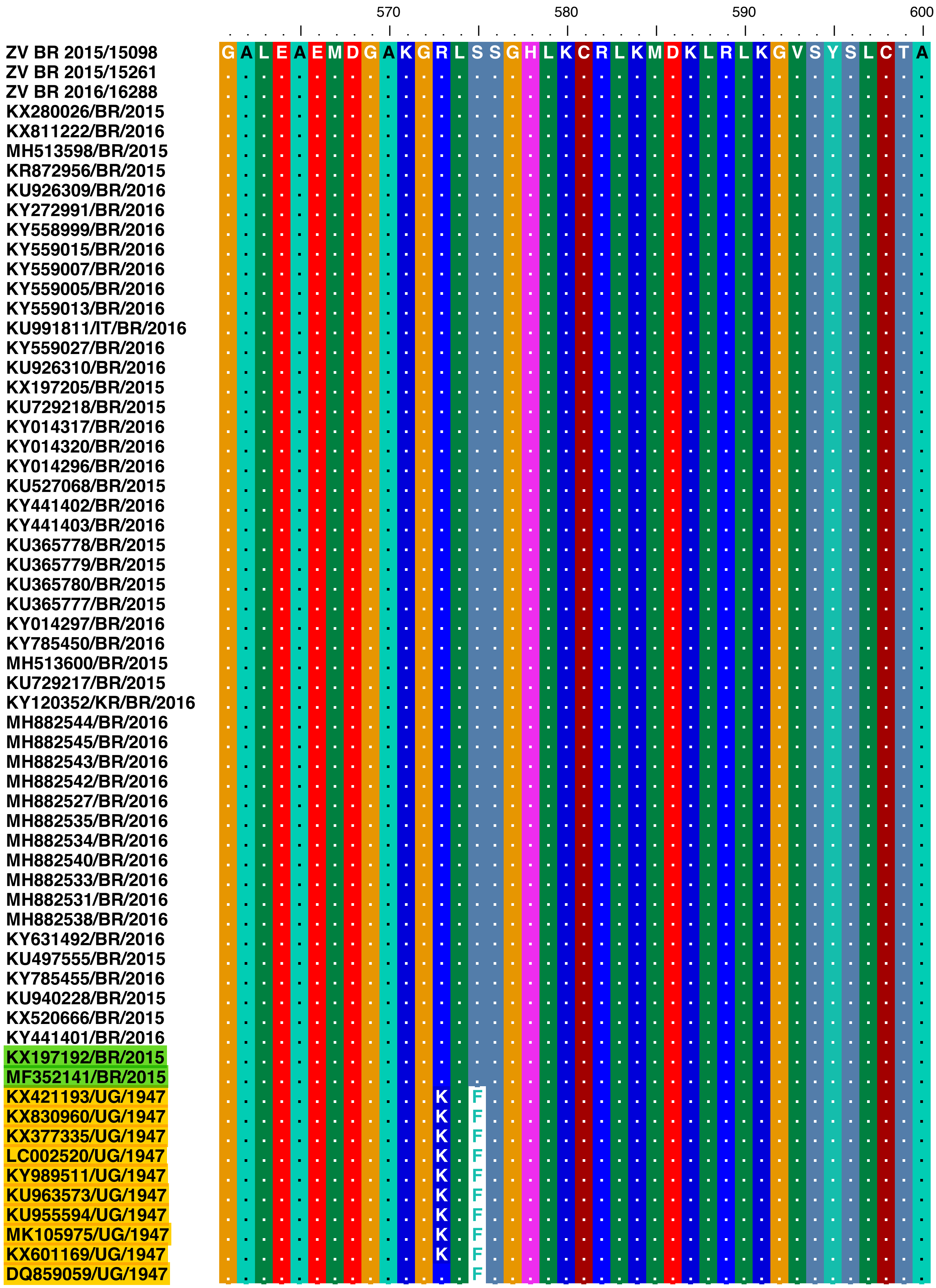
570
580
590
600
GALEAEMDGAKGRLSSGHLKCRLKMDKLRLKGVSYSLCTA
ZV BR 2015/15098
ZV BR 2015/15261
ZV BR 2016/16288
KX280026/BR/2015
KX811222/BR/2016
MH513598/BR/2015
KR872956/BR/2015
KU926309/BR/2016
KY272991/BR/2016
KY558999/BR/2016
KY559015/BR/2016
KY559007/BR/2016
KY559005/BR/2016
KY559013/BR/2016
KU991811/IT/BR/2016
KY559027/BR/2016
KU926310/BR/2016
KX197205/BR/2015
KU729218/BR/2015
KY014317/BR/2016
KY014320/BR/2016
KY014296/BR/2016
KU527068/BR/2015
KY441402/BR/2016
KY441403/BR/2016
KU365778/BR/2015
KU365779/BR/2015
KU365780/BR/2015
KU365777/BR/2015
KY014297/BR/2016
KY785450/BR/2016
MH513600/BR/2015
KU729217/BR/2015
KY120352/KR/BR/2016
MH882544/BR/2016
MH882545/BR/2016
MH882543/BR/2016
MH882542/BR/2016
MH882527/BR/2016
MH882535/BR/2016
MH882534/BR/2016
MH882540/BR/2016
MH882533/BR/2016
MH882531/BR/2016
MH882538/BR/2016
KY631492/BR/2016
KU497555/BR/2015
KY785455/BR/2016
KU940228/BR/2015
KX520666/BR/2015
KY441401/BR/2016
KX197192/BR/2015
MF352141/BR/2015
KX421193/UG/1947
KX830960/UG/1947
KX377335/UG/1947
LC002520/UG/1947
KY989511/UG/1947
KU963573/UG/1947
KU955594/UG/1947
MK105975/UG/1947
KX601169/UG/1947
DQ859059/UG/1947

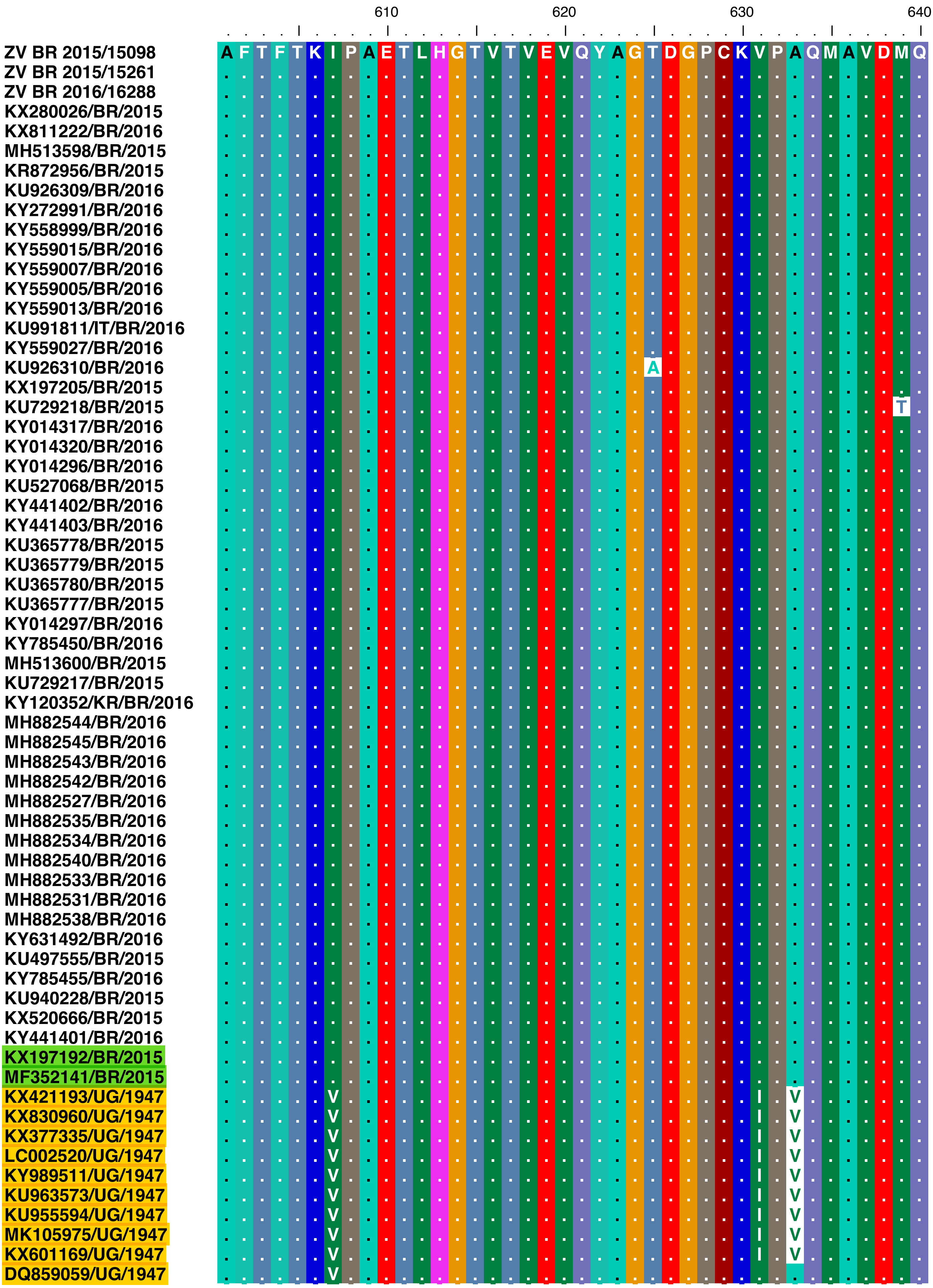
610
620
630
640
A F T F T K I P A E T L H G T V T V E V Q Y A G T D G P C K V P A Q M A V D M Q
ZV BR 2015/15098
ZV BR 2015/15261
ZV BR 2016/16288
KX280026/BR/2015
KX811222/BR/2016
MH513598/BR/2015
KR872956/BR/2015
KU926309/BR/2016
KY272991/BR/2016
KY558999/BR/2016
KY559015/BR/2016
KY559007/BR/2016
KY559005/BR/2016
KY559013/BR/2016
KU991811/IT/BR/2016
KY559027/BR/2016
KU926310/BR/2016
KX197205/BR/2015
KU729218/BR/2015
KY014317/BR/2016
KY014320/BR/2016
KY014296/BR/2016
KU527068/BR/2015
KY441402/BR/2016
KY441403/BR/2016
KU365778/BR/2015
KU365779/BR/2015
KU365780/BR/2015
KU365777/BR/2015
KY014297/BR/2016
KY785450/BR/2016
MH513600/BR/2015
KU729217/BR/2015
KY120352/KR/BR/2016
MH882544/BR/2016
MH882545/BR/2016
MH882543/BR/2016
MH882542/BR/2016
MH882527/BR/2016
MH882535/BR/2016
MH882534/BR/2016
MH882540/BR/2016
MH882533/BR/2016
MH882531/BR/2016
MH882538/BR/2016
KY631492/BR/2016
KU497555/BR/2015
KY785455/BR/2016
KU940228/BR/2015
KX520666/BR/2015
KY441401/BR/2016
KX197192/BR/2015
MF352141/BR/2015
KX421193/UG/1947
KX830960/UG/1947
KX377335/UG/1947
LC002520/UG/1947
KY989511/UG/1947
KU963573/UG/1947
KU955594/UG/1947
MK105975/UG/1947
KX601169/UG/1947
DQ859059/UG/1947

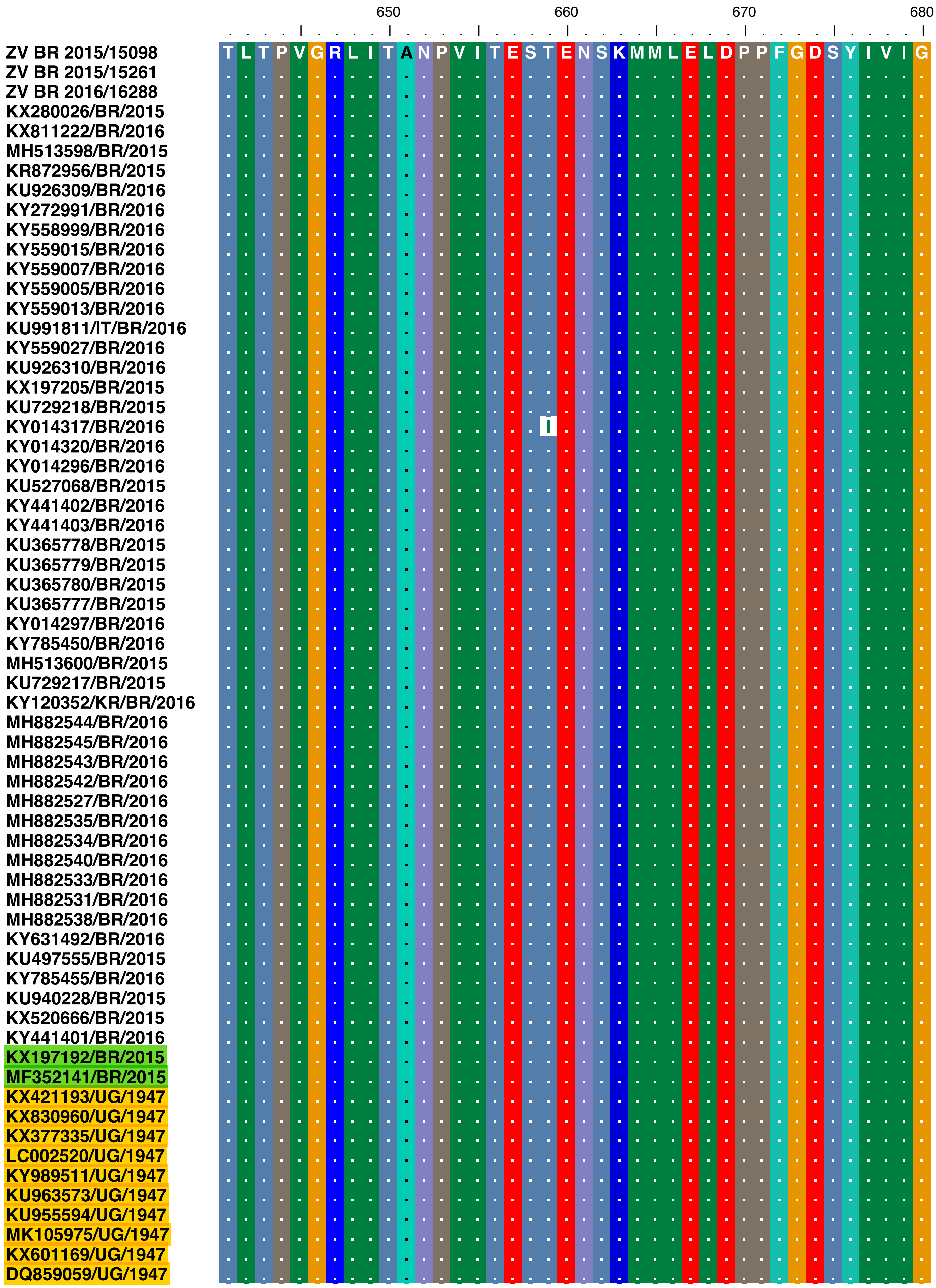

650
660
670
680
T L T P V G R L I T A N P V I T E S T E N S K M M L E L D P P F G D S Y I V I G
I
ZV BR 2015/15098
ZV BR 2015/15261
ZV BR 2016/16288
KX280026/BR/2015
KX811222/BR/2016
MH513598/BR/2015
KR872956/BR/2015
KU926309/BR/2016
KY272991/BR/2016
KY558999/BR/2016
KY559015/BR/2016
KY559007/BR/2016
KY559005/BR/2016
KY559013/BR/2016
KU991811/IT/BR/2016
KY559027/BR/2016
KU926310/BR/2016
KX197205/BR/2015
KU729218/BR/2015
KY014317/BR/2016
KY014320/BR/2016
KY014296/BR/2016
KU527068/BR/2015
KY441402/BR/2016
KY441403/BR/2016
KU365778/BR/2015
KU365779/BR/2015
KU365780/BR/2015
KU365777/BR/2015
KY014297/BR/2016
KY785450/BR/2016
MH513600/BR/2015
KU729217/BR/2015
KY120352/KR/BR/2016
MH882544/BR/2016
MH882545/BR/2016
MH882543/BR/2016
MH882542/BR/2016
MH882527/BR/2016
MH882535/BR/2016
MH882534/BR/2016
MH882540/BR/2016
MH882533/BR/2016
MH882531/BR/2016
MH882538/BR/2016
KY631492/BR/2016
KU497555/BR/2015
KY785455/BR/2016
KU940228/BR/2015
KX520666/BR/2015
KY441401/BR/2016
KX197192/BR/2015
MF352141/BR/2015
KX421193/UG/1947
KX830960/UG/1947
KX377335/UG/1947
LC002520/UG/1947
KY989511/UG/1947
KU963573/UG/1947
KU955594/UG/1947
MK105975/UG/1947
KX601169/UG/1947
DQ859059/UG/1947

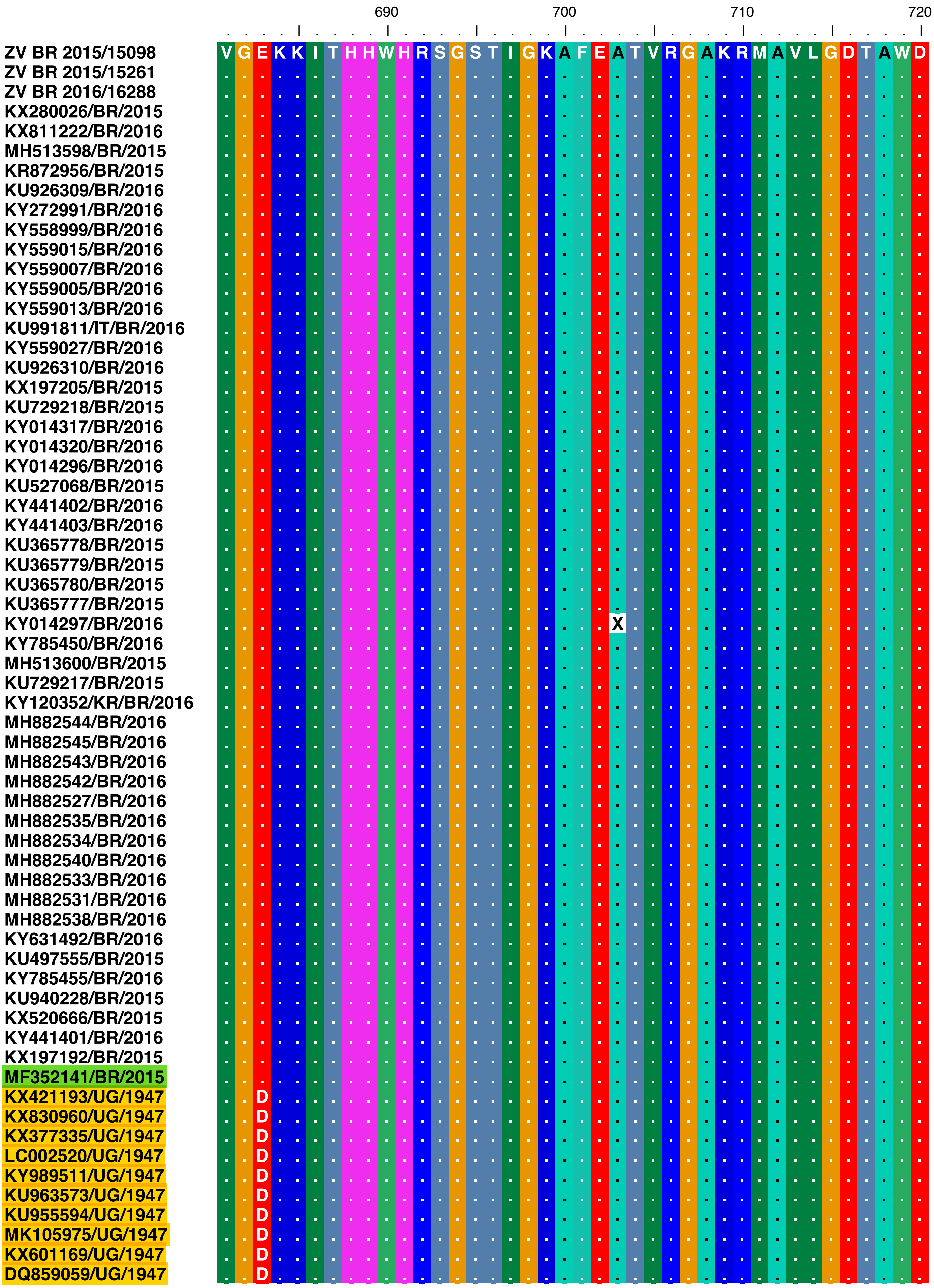

690
700
710
720
V G E K K I T H H W H R S G S T I G K A F E A T V R G A K R M A V L G D T A W D
ZV BR 2015/15098
ZV BR 2015/15261
ZV BR 2016/16288
KX280026/BR/2015
KX811222/BR/2016
MH513598/BR/2015
KR872956/BR/2015
KU926309/BR/2016
KY272991/BR/2016
KY558999/BR/2016
KY559015/BR/2016
KY559007/BR/2016
KY559005/BR/2016
KY559013/BR/2016
KU991811/IT/BR/2016
KY559027/BR/2016
KU926310/BR/2016
KX197205/BR/2015
KU729218/BR/2015
KY014317/BR/2016
KY014320/BR/2016
KY014296/BR/2016
KU527068/BR/2015
KY441402/BR/2016
KY441403/BR/2016
KU365778/BR/2015
KU365779/BR/2015
KU365780/BR/2015
KU365777/BR/2015
KY014297/BR/2016
X
KY785450/BR/2016
MH513600/BR/2015
KU729217/BR/2015
KY120352/KR/BR/2016
MH882544/BR/2016
MH882545/BR/2016
MH882543/BR/2016
MH882542/BR/2016
MH882527/BR/2016
MH882535/BR/2016
MH882534/BR/2016
MH882540/BR/2016
MH882533/BR/2016
MH882531/BR/2016
MH882538/BR/2016
KY631492/BR/2016
KU497555/BR/2015
KY785455/BR/2016
KU940228/BR/2015
KX520666/BR/2015
KY441401/BR/2016
KX197192/BR/2015
MF352141/BR/2015
KX421193/UG/1947
D
KX830960/UG/1947
D
KX377335/UG/1947
D
LC002520/UG/1947
D
KY989511/UG/1947
D
KU963573/UG/1947
D
KU955594/UG/1947
D
MK105975/UG/1947
D
KX601169/UG/1947
D
DQ859059/UG/1947
D

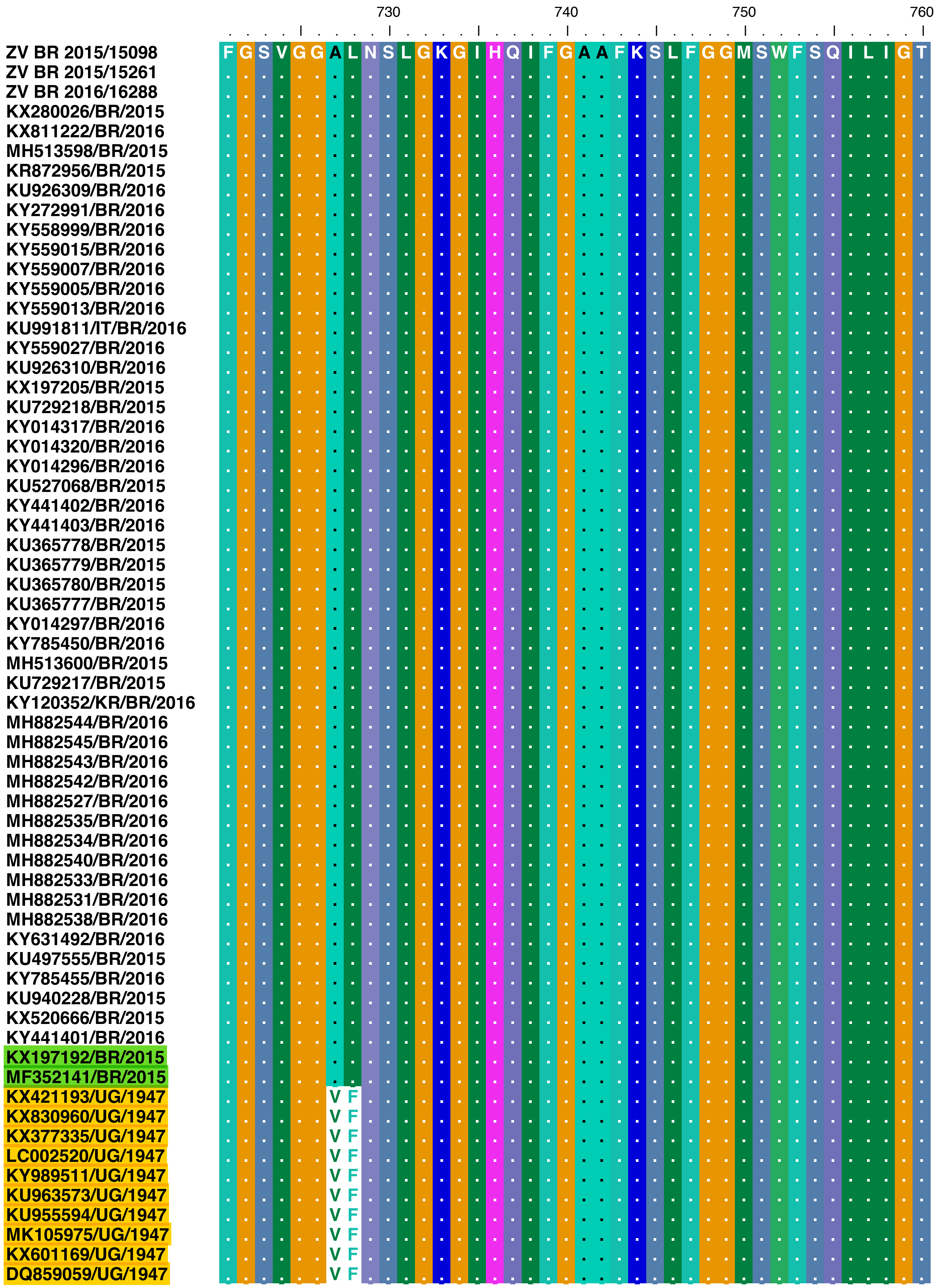

730
740
750
760
FGSVGGALNSLGKGIHQIFGAAFKSLFGGMSWFSQILIGT
ZV BR 2015/15098
ZV BR 2015/15261
ZV BR 2016/16288
KX280026/BR/2015
KX811222/BR/2016
MH513598/BR/2015
KR872956/BR/2015
KU926309/BR/2016
KY272991/BR/2016
KY558999/BR/2016
KY559015/BR/2016
KY559007/BR/2016
KY559005/BR/2016
KY559013/BR/2016
KU991811/IT/BR/2016
KY559027/BR/2016
KU926310/BR/2016
KX197205/BR/2015
KU729218/BR/2015
KY014317/BR/2016
KY014320/BR/2016
KY014296/BR/2016
KU527068/BR/2015
KY441402/BR/2016
KY441403/BR/2016
KU365778/BR/2015
KU365779/BR/2015
KU365780/BR/2015
KU365777/BR/2015
KY014297/BR/2016
KY785450/BR/2016
MH513600/BR/2015
KU729217/BR/2015
KY120352/KR/BR/2016
MH882544/BR/2016
MH882545/BR/2016
MH882543/BR/2016
MH882542/BR/2016
MH882527/BR/2016
MH882535/BR/2016
MH882534/BR/2016
MH882540/BR/2016
MH882533/BR/2016
MH882531/BR/2016
MH882538/BR/2016
KY631492/BR/2016
KU497555/BR/2015
KY785455/BR/2016
KU940228/BR/2015
KX520666/BR/2015
KY441401/BR/2016
KX197192/BR/2015
MF352141/BR/2015
KX421193/UG/1947 V F
KX830960/UG/1947 V F
KX377335/UG/1947 V F
LC002520/UG/1947 V F
KY989511/UG/1947 V F
KU963573/UG/1947 V F
KU955594/UG/1947 V F
MK105975/UG/1947 V F
KX601169/UG/1947 V F
DQ859059/UG/1947 V F

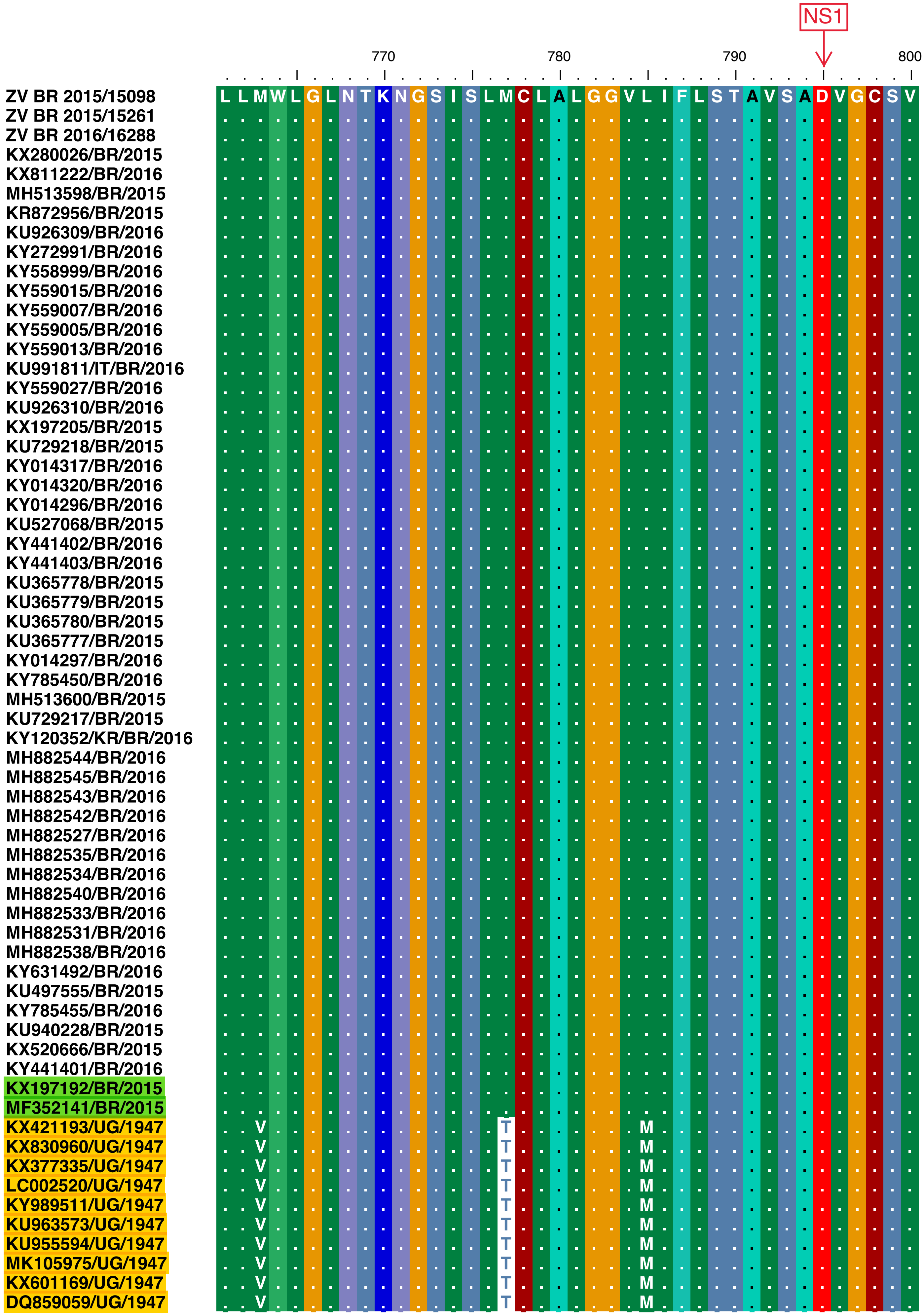
NS1
770
780
790
800
L L M W L G L N T K N G S I S L M C L A L G G V L I F L S T A V S A D V G C S V
ZV BR 2015/15098
ZV BR 2015/15261
ZV BR 2016/16288
KX280026/BR/2015
KX811222/BR/2016
MH513598/BR/2015
KR872956/BR/2015
KU926309/BR/2016
KY272991/BR/2016
KY558999/BR/2016
KY559015/BR/2016
KY559007/BR/2016
KY559005/BR/2016
KY559013/BR/2016
KU991811/IT/BR/2016
KY559027/BR/2016
KU926310/BR/2016
KX197205/BR/2015
KU729218/BR/2015
KY014317/BR/2016
KY014320/BR/2016
KY014296/BR/2016
KU527068/BR/2015
KY441402/BR/2016
KY441403/BR/2016
KU365778/BR/2015
KU365779/BR/2015
KU365780/BR/2015
KU365777/BR/2015
KY014297/BR/2016
KY785450/BR/2016
MH513600/BR/2015
KU729217/BR/2015
KY120352/KR/BR/2016
MH882544/BR/2016
MH882545/BR/2016
MH882543/BR/2016
MH882542/BR/2016
MH882527/BR/2016
MH882535/BR/2016
MH882534/BR/2016
MH882540/BR/2016
MH882533/BR/2016
MH882531/BR/2016
MH882538/BR/2016
KY631492/BR/2016
KU497555/BR/2015
KY785455/BR/2016
KU940228/BR/2015
KX520666/BR/2015
KY441401/BR/2016
KX197192/BR/2015
MF352141/BR/2015
KX421193/UG/1947
KX830960/UG/1947
KX377335/UG/1947
LC002520/UG/1947
KY989511/UG/1947
KU963573/UG/1947
KU955594/UG/1947
MK105975/UG/1947
KX601169/UG/1947
DQ859059/UG/1947
V
T
M

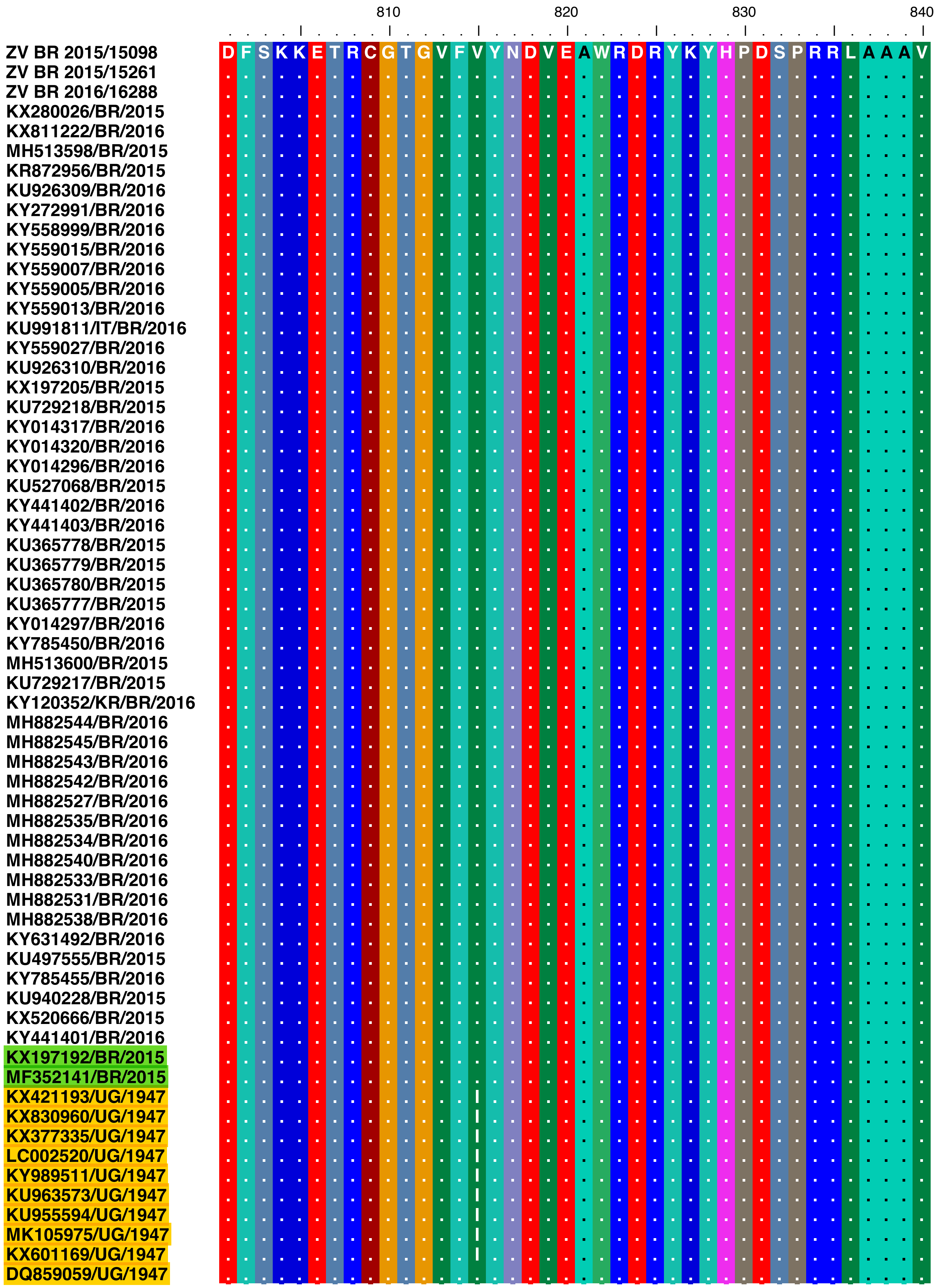
810
820
830
840
D F S K K E T R C G T G V F V Y N D V E A W R D R Y K Y H P D S P R R L A A A V
ZV BR 2015/15098
ZV BR 2015/15261
ZV BR 2016/16288
KX280026/BR/2015
KX811222/BR/2016
MH513598/BR/2015
KR872956/BR/2015
KU926309/BR/2016
KY272991/BR/2016
KY558999/BR/2016
KY559015/BR/2016
KY559007/BR/2016
KY559005/BR/2016
KY559013/BR/2016
KU991811/IT/BR/2016
KY559027/BR/2016
KU926310/BR/2016
KX197205/BR/2015
KU729218/BR/2015
KY014317/BR/2016
KY014320/BR/2016
KY014296/BR/2016
KU527068/BR/2015
KY441402/BR/2016
KY441403/BR/2016
KU365778/BR/2015
KU365779/BR/2015
KU365780/BR/2015
KU365777/BR/2015
KY014297/BR/2016
KY785450/BR/2016
MH513600/BR/2015
KU729217/BR/2015
KY120352/KR/BR/2016
MH882544/BR/2016
MH882545/BR/2016
MH882543/BR/2016
MH882542/BR/2016
MH882527/BR/2016
MH882535/BR/2016
MH882534/BR/2016
MH882540/BR/2016
MH882533/BR/2016
MH882531/BR/2016
MH882538/BR/2016
KY631492/BR/2016
KU497555/BR/2015
KY785455/BR/2016
KU940228/BR/2015
KX520666/BR/2015
KY441401/BR/2016
KX197192/BR/2015
MF352141/BR/2015
KX421193/UG/1947
KX830960/UG/1947
KX377335/UG/1947
LC002520/UG/1947
KY989511/UG/1947
KU963573/UG/1947
KU955594/UG/1947
MK105975/UG/1947
KX601169/UG/1947
DQ859059/UG/1947

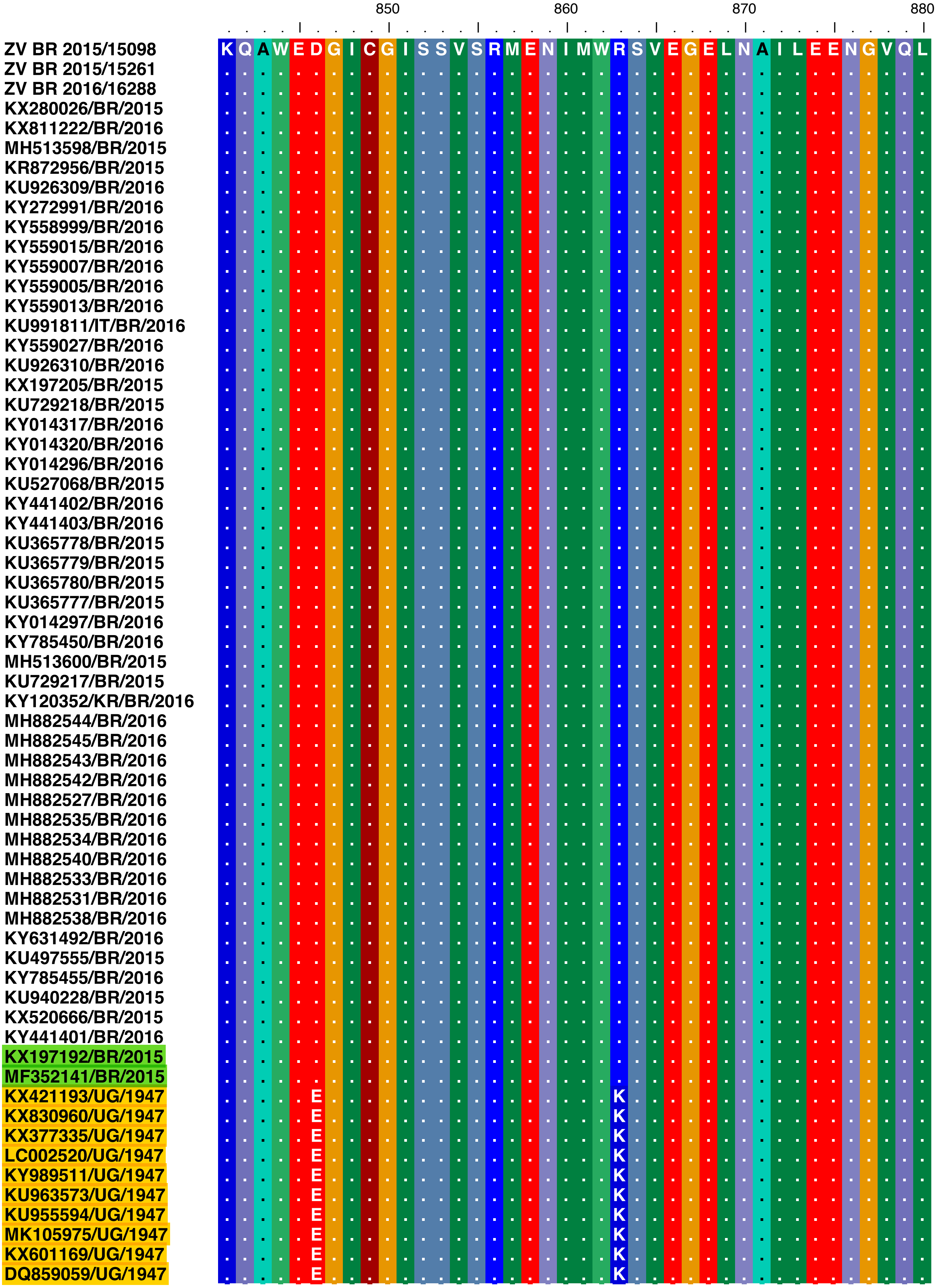

850
860
870
880
KQAWEDGICGISSVSRMENIMWRSVEGELNAILEENGVQL
ZV BR 2015/15098
ZV BR 2015/15261
ZV BR 2016/16288
KX280026/BR/2015
KX811222/BR/2016
MH513598/BR/2015
KR872956/BR/2015
KU926309/BR/2016
KY272991/BR/2016
KY558999/BR/2016
KY559015/BR/2016
KY559007/BR/2016
KY559005/BR/2016
KY559013/BR/2016
KU991811/IT/BR/2016
KY559027/BR/2016
KU926310/BR/2016
KX197205/BR/2015
KU729218/BR/2015
KY014317/BR/2016
KY014320/BR/2016
KY014296/BR/2016
KU527068/BR/2015
KY441402/BR/2016
KY441403/BR/2016
KU365778/BR/2015
KU365779/BR/2015
KU365780/BR/2015
KU365777/BR/2015
KY014297/BR/2016
KY785450/BR/2016
MH513600/BR/2015
KU729217/BR/2015
KY120352/KR/BR/2016
MH882544/BR/2016
MH882545/BR/2016
MH882543/BR/2016
MH882542/BR/2016
MH882527/BR/2016
MH882535/BR/2016
MH882534/BR/2016
MH882540/BR/2016
MH882533/BR/2016
MH882531/BR/2016
MH882538/BR/2016
KY631492/BR/2016
KU497555/BR/2015
KY785455/BR/2016
KU940228/BR/2015
KX520666/BR/2015
KY441401/BR/2016
KX197192/BR/2015
MF352141/BR/2015
KX421193/UG/1947
KX830960/UG/1947
KX377335/UG/1947
LC002520/UG/1947
KY989511/UG/1947
KU963573/UG/1947
KU955594/UG/1947
MK105975/UG/1947
KX601169/UG/1947
DQ859059/UG/1947
E
K

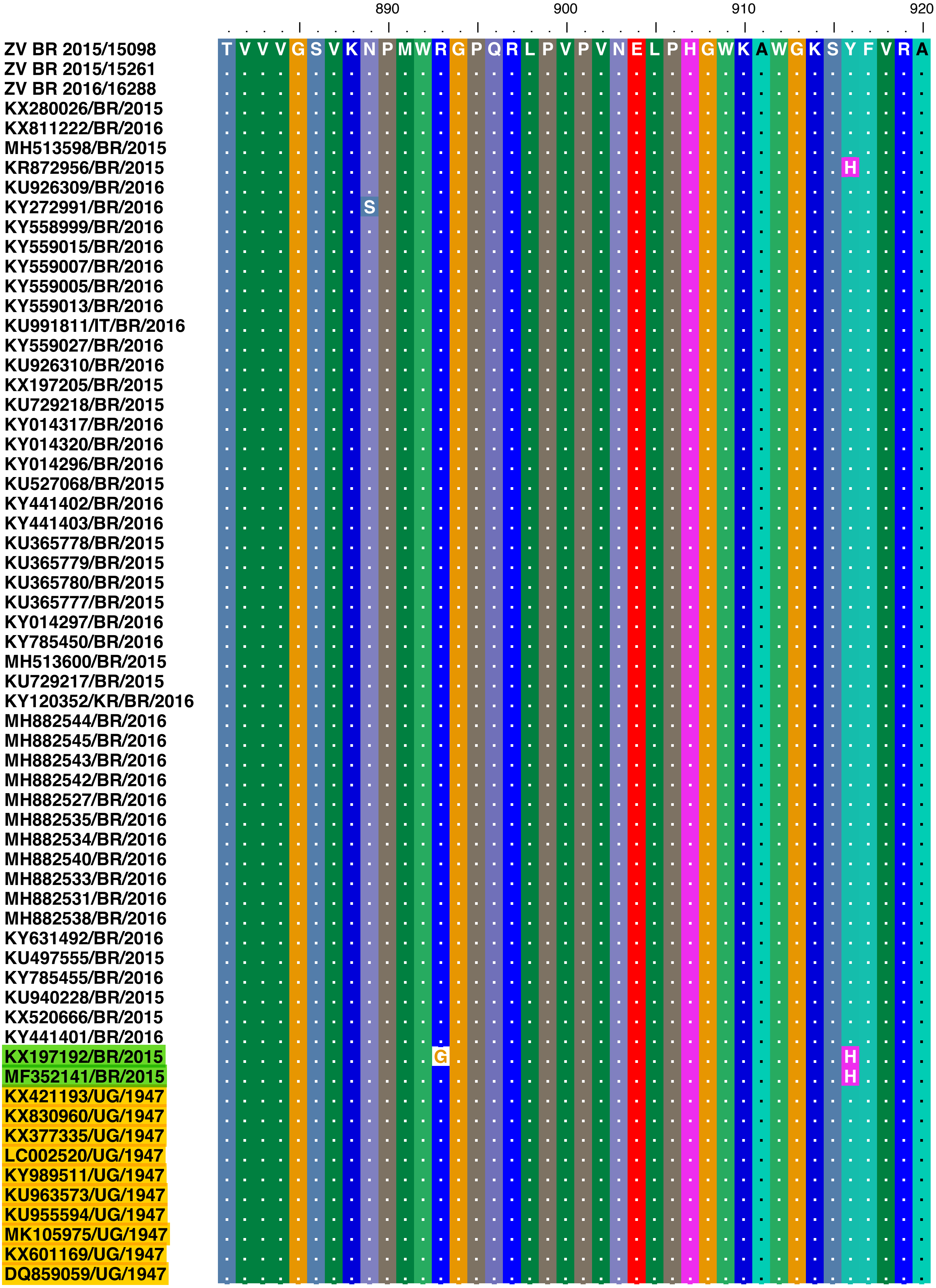
890
900
910
920
T V V V G S V K N P M W R G P Q R L P V P V N E L P H G W K A W G K S Y F V R A
ZV BR 2015/15098
ZV BR 2015/15261
ZV BR 2016/16288
KX280026/BR/2015
KX811222/BR/2016
MH513598/BR/2015
KR872956/BR/2015
H
KU926309/BR/2016
KY272991/BR/2016
S
KY558999/BR/2016
KY559015/BR/2016
KY559007/BR/2016
KY559005/BR/2016
KY559013/BR/2016
KU991811/IT/BR/2016
KY559027/BR/2016
KU926310/BR/2016
KX197205/BR/2015
KU729218/BR/2015
KY014317/BR/2016
KY014320/BR/2016
KY014296/BR/2016
KU527068/BR/2015
KY441402/BR/2016
KY441403/BR/2016
KU365778/BR/2015
KU365779/BR/2015
KU365780/BR/2015
KU365777/BR/2015
KY014297/BR/2016
KY785450/BR/2016
MH513600/BR/2015
KU729217/BR/2015
KY120352/KR/BR/2016
MH882544/BR/2016
MH882545/BR/2016
MH882543/BR/2016
MH882542/BR/2016
MH882527/BR/2016
MH882535/BR/2016
MH882534/BR/2016
MH882540/BR/2016
MH882533/BR/2016
MH882531/BR/2016
MH882538/BR/2016
KY631492/BR/2016
KU497555/BR/2015
KY785455/BR/2016
KU940228/BR/2015
KX520666/BR/2015
KY441401/BR/2016
KX197192/BR/2015
G
H
MF352141/BR/2015
H
KX421193/UG/1947
KX830960/UG/1947
KX377335/UG/1947
LC002520/UG/1947
KY989511/UG/1947
KU963573/UG/1947
KU955594/UG/1947
MK105975/UG/1947
KX601169/UG/1947
DQ859059/UG/1947

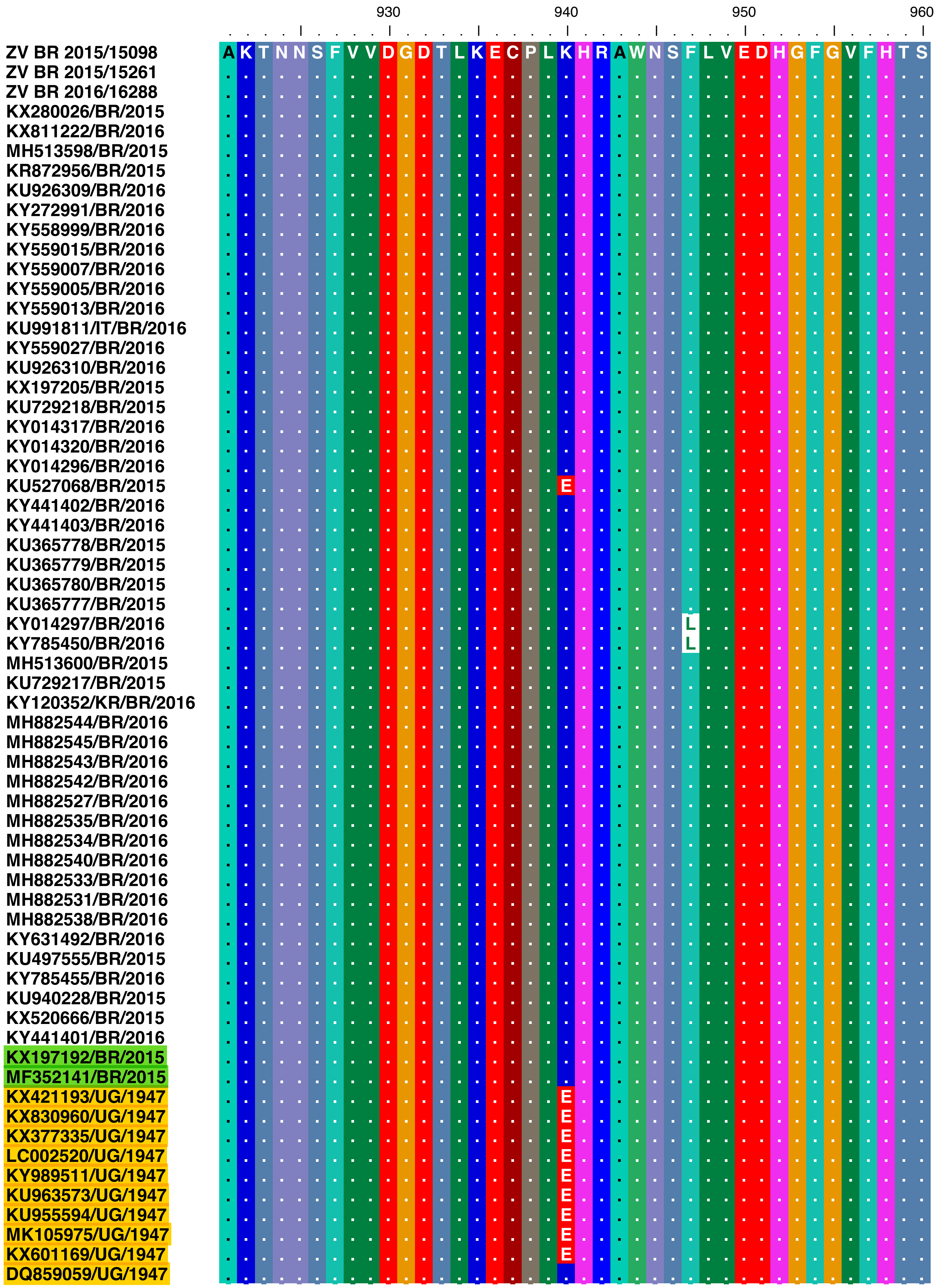
930
940
950
960
A K T N N S F V V D G D T L K E C P L K H R A W N S F L V E D H G F G V F H T S
ZV BR 2015/15098
ZV BR 2015/15261
ZV BR 2016/16288
KX280026/BR/2015
KX811222/BR/2016
MH513598/BR/2015
KR872956/BR/2015
KU926309/BR/2016
KY272991/BR/2016
KY558999/BR/2016
KY559015/BR/2016
KY559007/BR/2016
KY559005/BR/2016
KY559013/BR/2016
KU991811/IT/BR/2016
KY559027/BR/2016
KU926310/BR/2016
KX197205/BR/2015
KU729218/BR/2015
KY014317/BR/2016
KY014320/BR/2016
KY014296/BR/2016
KU527068/BR/2015
KY441402/BR/2016
KY441403/BR/2016
KU365778/BR/2015
KU365779/BR/2015
KU365780/BR/2015
KU365777/BR/2015
KY014297/BR/2016
KY785450/BR/2016
MH513600/BR/2015
KU729217/BR/2015
KY120352/KR/BR/2016
MH882544/BR/2016
MH882545/BR/2016
MH882543/BR/2016
MH882542/BR/2016
MH882527/BR/2016
MH882535/BR/2016
MH882534/BR/2016
MH882540/BR/2016
MH882533/BR/2016
MH882531/BR/2016
MH882538/BR/2016
KY631492/BR/2016
KU497555/BR/2015
KY785455/BR/2016
KU940228/BR/2015
KX520666/BR/2015
KY441401/BR/2016
KX197192/BR/2015
MF352141/BR/2015
KX421193/UG/1947
KX830960/UG/1947
KX377335/UG/1947
LC002520/UG/1947
KY989511/UG/1947
KU963573/UG/1947
KU955594/UG/1947
MK105975/UG/1947
KX601169/UG/1947
DQ859059/UG/1947

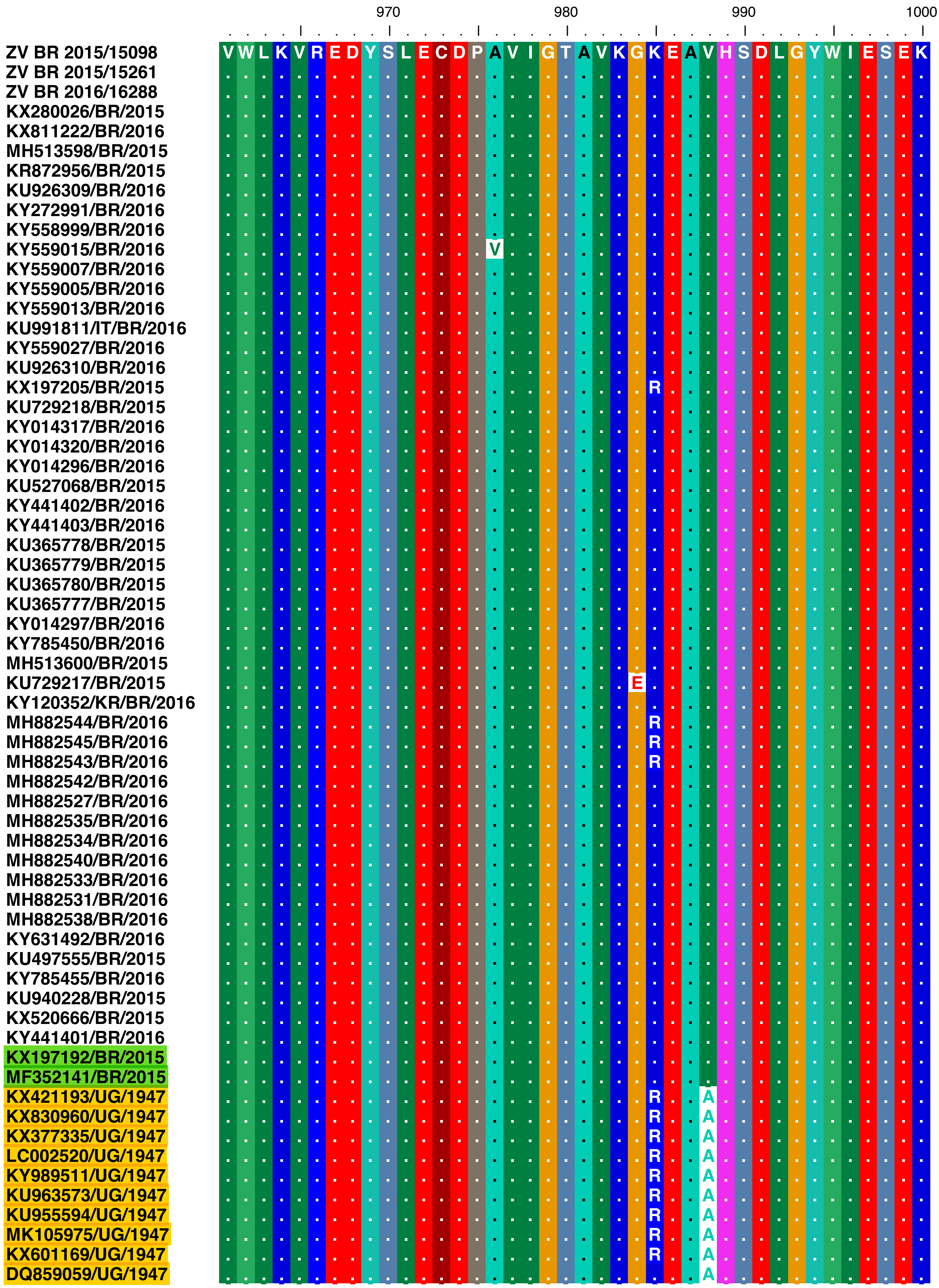
970
980
990
1000
VWLKVREDYSLECDPAVIGTAVKGKEAVHSDLGYWIESEK
ZV BR 2015/15098
ZV BR 2015/15261
ZV BR 2016/16288
KX280026/BR/2015
KX811222/BR/2016
MH513598/BR/2015
KR872956/BR/2015
KU926309/BR/2016
KY272991/BR/2016
KY558999/BR/2016
KY559015/BR/2016
KY559007/BR/2016
KY559005/BR/2016
KY559013/BR/2016
KU991811/IT/BR/2016
KY559027/BR/2016
KU926310/BR/2016
KX197205/BR/2015
KU729218/BR/2015
KY014317/BR/2016
KY014320/BR/2016
KY014296/BR/2016
KU527068/BR/2015
KY441402/BR/2016
KY441403/BR/2016
KU365778/BR/2015
KU365779/BR/2015
KU365780/BR/2015
KU365777/BR/2015
KY014297/BR/2016
KY785450/BR/2016
MH513600/BR/2015
KU729217/BR/2015
KY120352/KR/BR/2016
MH882544/BR/2016
MH882545/BR/2016
MH882543/BR/2016
MH882542/BR/2016
MH882527/BR/2016
MH882535/BR/2016
MH882534/BR/2016
MH882540/BR/2016
MH882533/BR/2016
MH882531/BR/2016
MH882538/BR/2016
KY631492/BR/2016
KU497555/BR/2015
KY785455/BR/2016
KU940228/BR/2015
KX520666/BR/2015
KY441401/BR/2016
KX197192/BR/2015
MF352141/BR/2015
KX421193/UG/1947
KX830960/UG/1947
KX377335/UG/1947
LC002520/UG/1947
KY989511/UG/1947
KU963573/UG/1947
KU955594/UG/1947
MK105975/UG/1947
KX601169/UG/1947
DQ859059/UG/1947

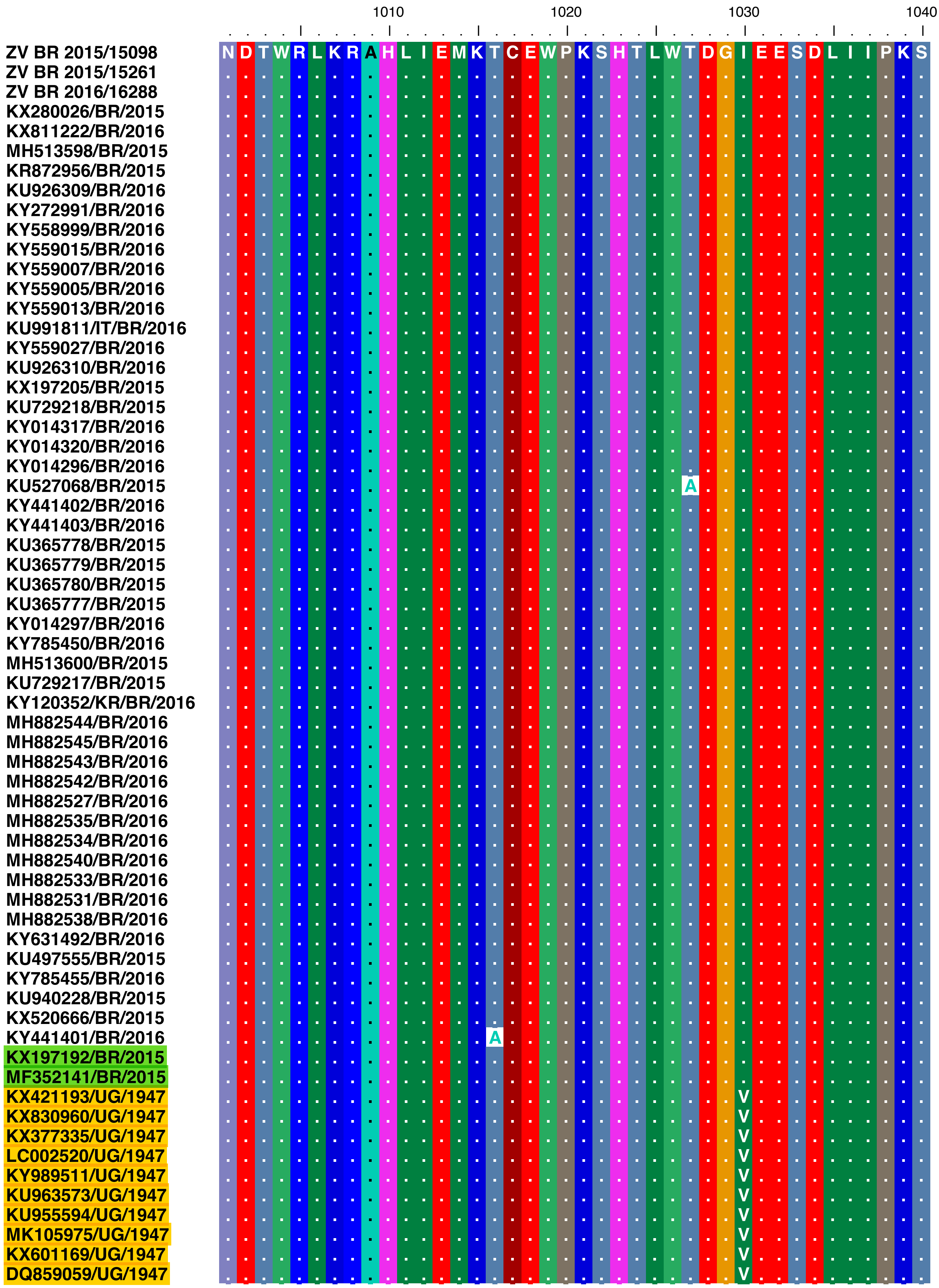

1010
1020
1030
1040
N D T W R L K R A H L I E M K T C E W P K S H T L W T D G I E E S D L I I P K S
ZV BR 2015/15098
ZV BR 2015/15261
ZV BR 2016/16288
KX280026/BR/2015
KX811222/BR/2016
MH513598/BR/2015
KR872956/BR/2015
KU926309/BR/2016
KY272991/BR/2016
KY558999/BR/2016
KY559015/BR/2016
KY559007/BR/2016
KY559005/BR/2016
KY559013/BR/2016
KU991811/IT/BR/2016
KY559027/BR/2016
KU926310/BR/2016
KX197205/BR/2015
KU729218/BR/2015
KY014317/BR/2016
KY014320/BR/2016
KY014296/BR/2016
KU527068/BR/2015
A
KY441402/BR/2016
KY441403/BR/2016
KU365778/BR/2015
KU365779/BR/2015
KU365780/BR/2015
KU365777/BR/2015
KY014297/BR/2016
KY785450/BR/2016
MH513600/BR/2015
KU729217/BR/2015
KY120352/KR/BR/2016
MH882544/BR/2016
MH882545/BR/2016
MH882543/BR/2016
MH882542/BR/2016
MH882527/BR/2016
MH882535/BR/2016
MH882534/BR/2016
MH882540/BR/2016
MH882533/BR/2016
MH882531/BR/2016
MH882538/BR/2016
KY631492/BR/2016
KU497555/BR/2015
KY785455/BR/2016
KU940228/BR/2015
KX520666/BR/2015
KY441401/BR/2016
A
KX197192/BR/2015
MF352141/BR/2015
KX421193/UG/1947
V
KX830960/UG/1947
V
KX377335/UG/1947
V
LC002520/UG/1947
V
KY989511/UG/1947
V
KU963573/UG/1947
V
KU955594/UG/1947
V
MK105975/UG/1947
V
KX601169/UG/1947
V
DQ859059/UG/1947
V

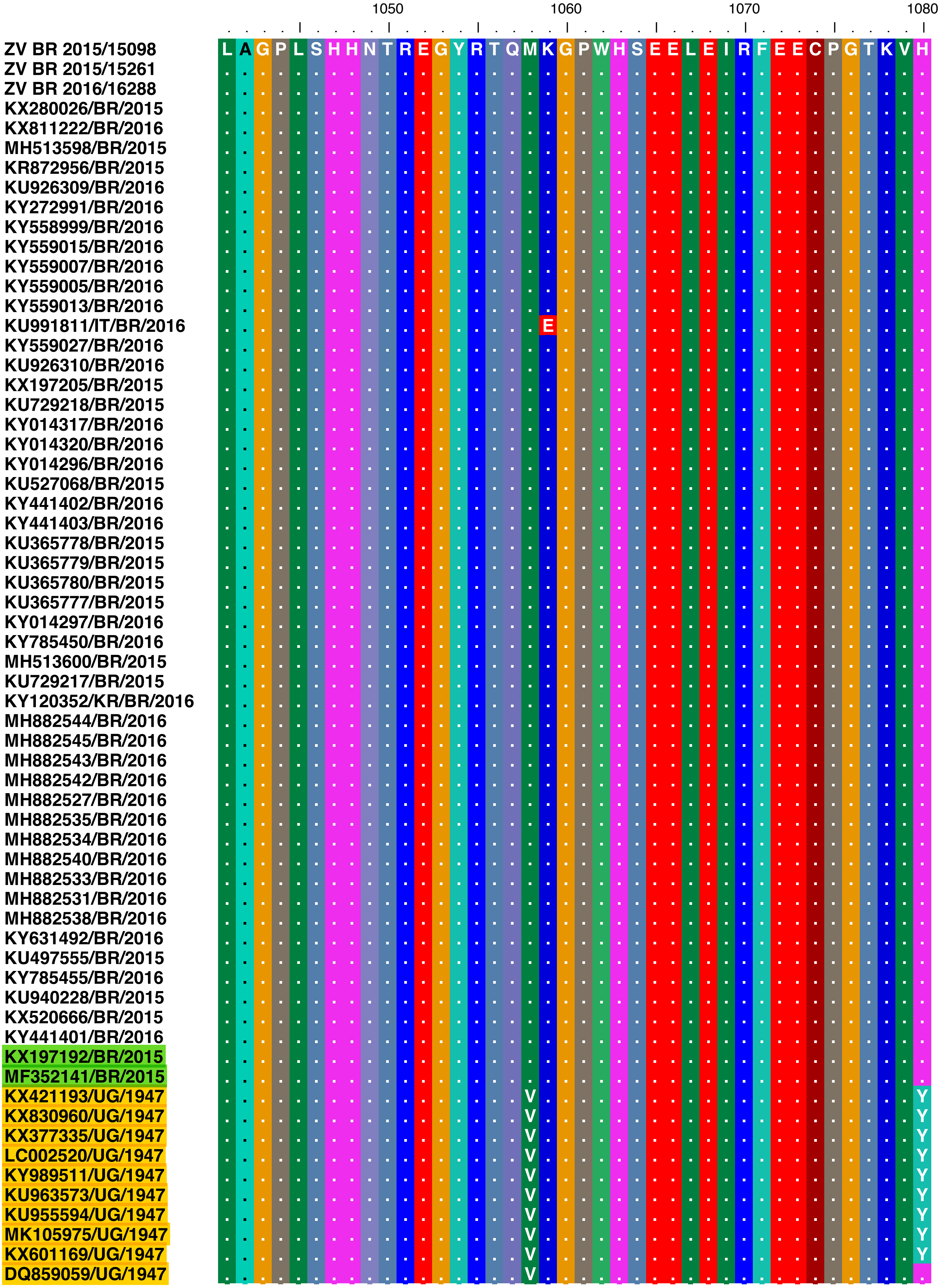

1050
1060
1070
1080
LAGPLSHHNTREGYRTQMKGPWHSEELEIRFEECPGTKVH
ZV BR 2015/15098
ZV BR 2015/15261
ZV BR 2016/16288
KX280026/BR/2015
KX811222/BR/2016
MH513598/BR/2015
KR872956/BR/2015
KU926309/BR/2016
KY272991/BR/2016
KY558999/BR/2016
KY559015/BR/2016
KY559007/BR/2016
KY559005/BR/2016
KY559013/BR/2016
KU991811/IT/BR/2016
E
KY559027/BR/2016
KU926310/BR/2016
KX197205/BR/2015
KU729218/BR/2015
KY014317/BR/2016
KY014320/BR/2016
KY014296/BR/2016
KU527068/BR/2015
KY441402/BR/2016
KY441403/BR/2016
KU365778/BR/2015
KU365779/BR/2015
KU365780/BR/2015
KU365777/BR/2015
KY014297/BR/2016
KY785450/BR/2016
MH513600/BR/2015
KU729217/BR/2015
KY120352/KR/BR/2016
MH882544/BR/2016
MH882545/BR/2016
MH882543/BR/2016
MH882542/BR/2016
MH882527/BR/2016
MH882535/BR/2016
MH882534/BR/2016
MH882540/BR/2016
MH882533/BR/2016
MH882531/BR/2016
MH882538/BR/2016
KY631492/BR/2016
KU497555/BR/2015
KY785455/BR/2016
KU940228/BR/2015
KX520666/BR/2015
KY441401/BR/2016
KX197192/BR/2015
MF352141/BR/2015
KX421193/UG/1947
KX830960/UG/1947
KX377335/UG/1947
LC002520/UG/1947
KY989511/UG/1947
KU963573/UG/1947
KU955594/UG/1947
MK105975/UG/1947
KX601169/UG/1947
DQ859059/UG/1947
V
Y

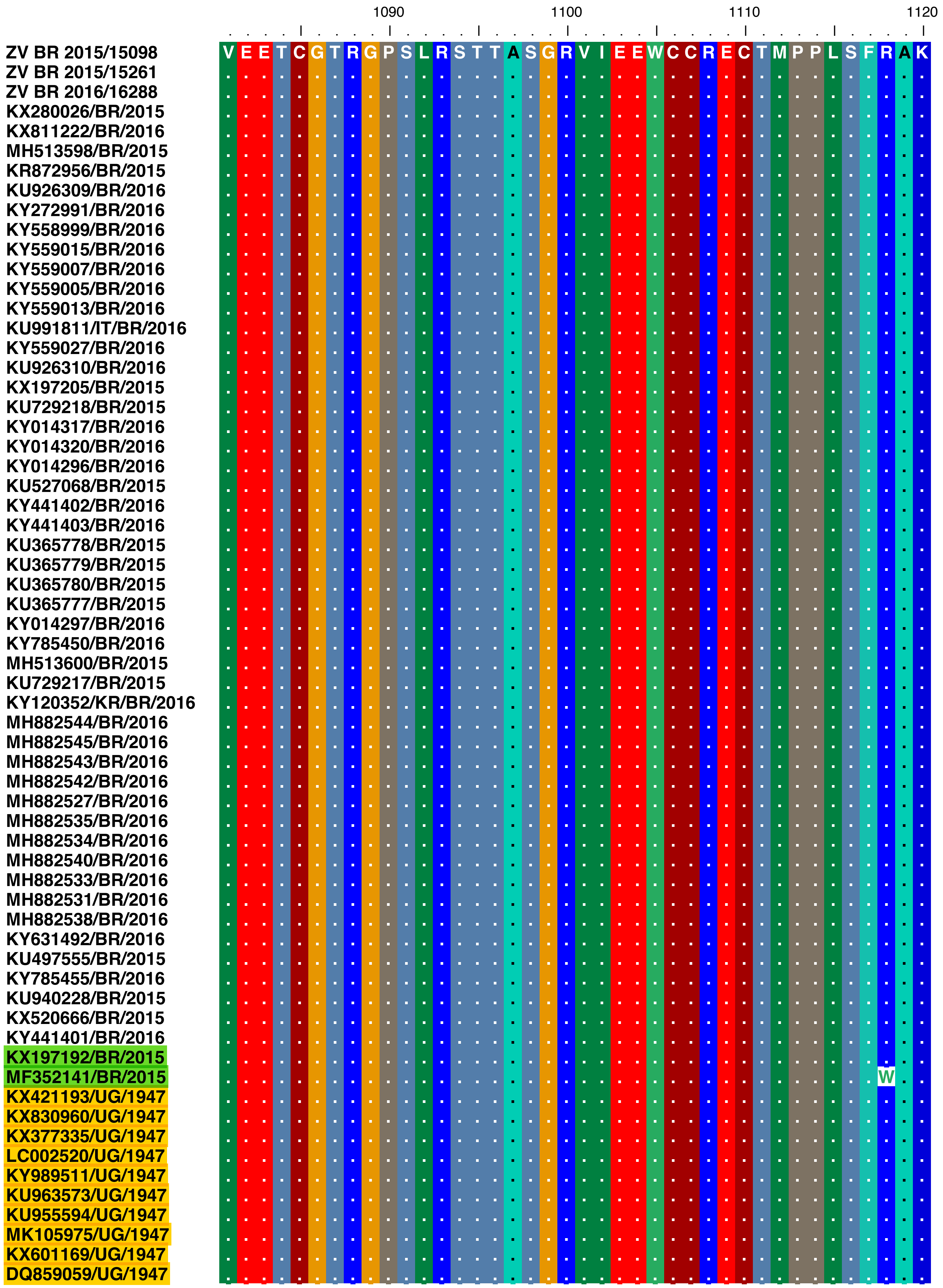
1090
1100
1110
1120
V E E T C G T R G P S L R S T T A S G R V I E E W C C R E C T M P P L S F R A K
ZV BR 2015/15098
ZV BR 2015/15261
ZV BR 2016/16288
KX280026/BR/2015
KX811222/BR/2016
MH513598/BR/2015
KR872956/BR/2015
KU926309/BR/2016
KY272991/BR/2016
KY558999/BR/2016
KY559015/BR/2016
KY559007/BR/2016
KY559005/BR/2016
KY559013/BR/2016
KU991811/IT/BR/2016
KY559027/BR/2016
KU926310/BR/2016
KX197205/BR/2015
KU729218/BR/2015
KY014317/BR/2016
KY014320/BR/2016
KY014296/BR/2016
KU527068/BR/2015
KY441402/BR/2016
KY441403/BR/2016
KU365778/BR/2015
KU365779/BR/2015
KU365780/BR/2015
KU365777/BR/2015
KY014297/BR/2016
KY785450/BR/2016
MH513600/BR/2015
KU729217/BR/2015
KY120352/KR/BR/2016
MH882544/BR/2016
MH882545/BR/2016
MH882543/BR/2016
MH882542/BR/2016
MH882527/BR/2016
MH882535/BR/2016
MH882534/BR/2016
MH882540/BR/2016
MH882533/BR/2016
MH882531/BR/2016
MH882538/BR/2016
KY631492/BR/2016
KU497555/BR/2015
KY785455/BR/2016
KU940228/BR/2015
KX520666/BR/2015
KY441401/BR/2016
KX197192/BR/2015
MF352141/BR/2015
W
KX421193/UG/1947
KX830960/UG/1947
KX377335/UG/1947
LC002520/UG/1947
KY989511/UG/1947
KU963573/UG/1947
KU955594/UG/1947
MK105975/UG/1947
KX601169/UG/1947
DQ859059/UG/1947

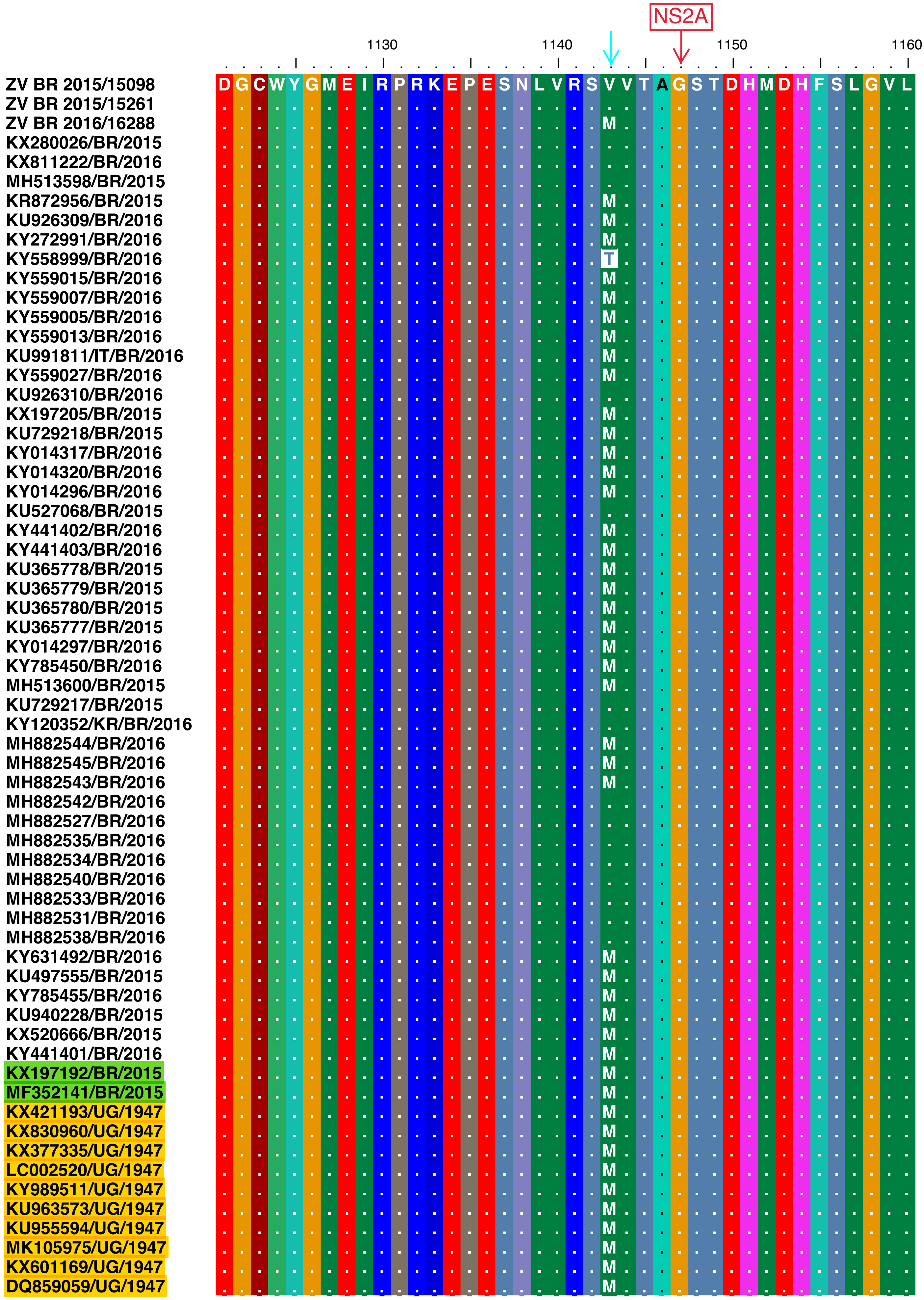
NS2A
1130
1140
1150
1160
D G C W Y G M E I R P R K E P E S N L V R S V V T A G S T D H M D H F S L G V L
ZV BR 2015/15098
ZV BR 2015/15261
ZV BR 2016/16288
KX280026/BR/2015
KX811222/BR/2016
MH513598/BR/2015
KR872956/BR/2015
KU926309/BR/2016
KY272991/BR/2016
KY558999/BR/2016
KY559015/BR/2016
KY559007/BR/2016
KY559005/BR/2016
KY559013/BR/2016
KU991811/IT/BR/2016
KY559027/BR/2016
KU926310/BR/2016
KX197205/BR/2015
KU729218/BR/2015
KY014317/BR/2016
KY014320/BR/2016
KY014296/BR/2016
KU527068/BR/2015
KY441402/BR/2016
KY441403/BR/2016
KU365778/BR/2015
KU365779/BR/2015
KU365780/BR/2015
KU365777/BR/2015
KY014297/BR/2016
KY785450/BR/2016
MH513600/BR/2015
KU729217/BR/2015
KY120352/KR/BR/2016
MH882544/BR/2016
MH882545/BR/2016
MH882543/BR/2016
MH882542/BR/2016
MH882527/BR/2016
MH882535/BR/2016
MH882534/BR/2016
MH882540/BR/2016
MH882533/BR/2016
MH882531/BR/2016
MH882538/BR/2016
KY631492/BR/2016
KU497555/BR/2015
KY785455/BR/2016
KU940228/BR/2015
KX520666/BR/2015
KY441401/BR/2016
KX197192/BR/2015
MF352141/BR/2015
KX421193/UG/1947
KX830960/UG/1947
KX377335/UG/1947
LC002520/UG/1947
KY989511/UG/1947
KU963573/UG/1947
KU955594/UG/1947
MK105975/UG/1947
KX601169/UG/1947
DQ859059/UG/1947

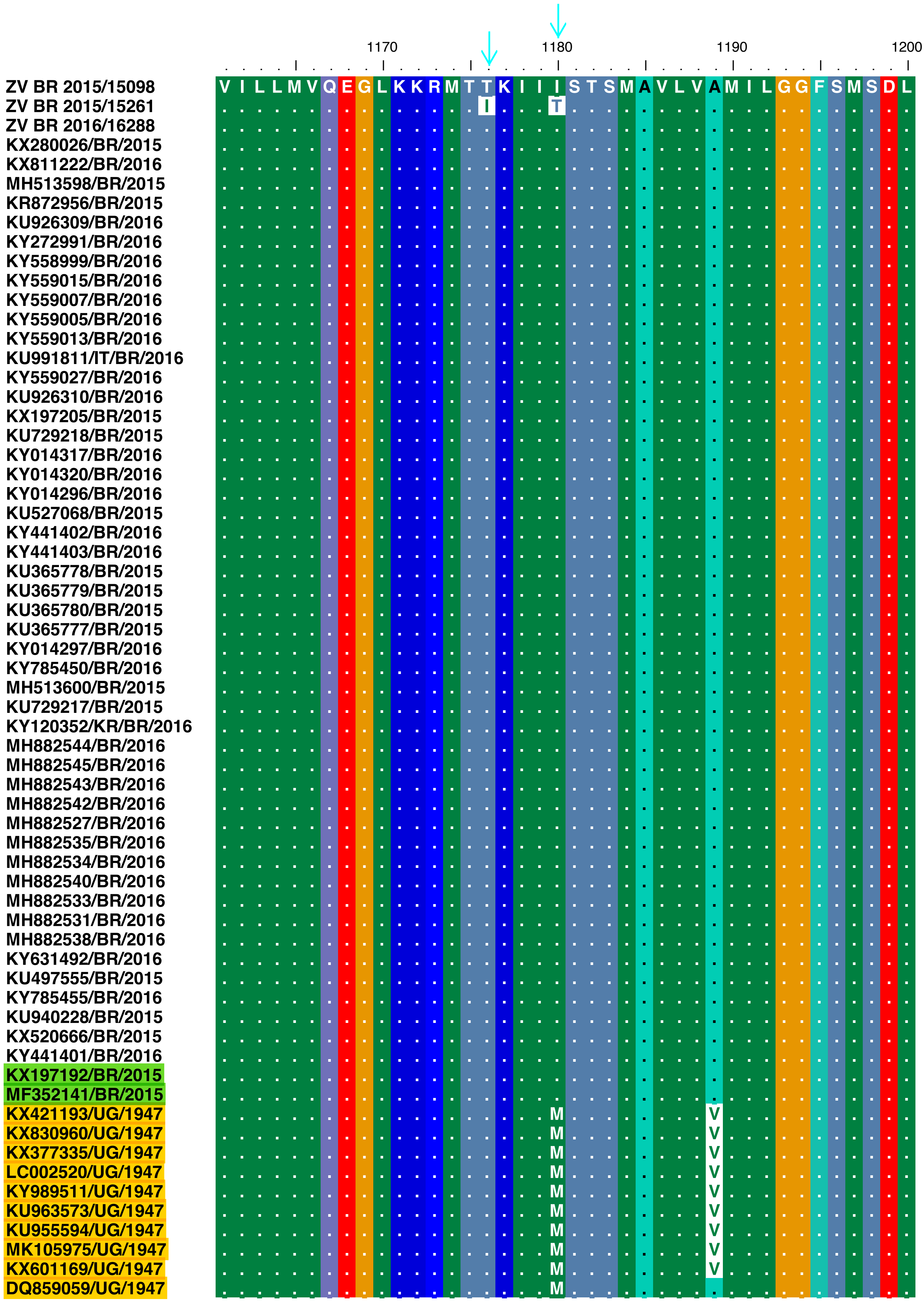

1170
1180
1190
1200
V I L L M V Q E G L K K R M T T K I I I S T S M A V L V A M I L G G F S M S D L
I
T
M
V
ZV BR 2015/15098
ZV BR 2015/15261
ZV BR 2016/16288
KX280026/BR/2015
KX811222/BR/2016
MH513598/BR/2015
KR872956/BR/2015
KU926309/BR/2016
KY272991/BR/2016
KY558999/BR/2016
KY559015/BR/2016
KY559007/BR/2016
KY559005/BR/2016
KY559013/BR/2016
KU991811/IT/BR/2016
KY559027/BR/2016
KU926310/BR/2016
KX197205/BR/2015
KU729218/BR/2015
KY014317/BR/2016
KY014320/BR/2016
KY014296/BR/2016
KU527068/BR/2015
KY441402/BR/2016
KY441403/BR/2016
KU365778/BR/2015
KU365779/BR/2015
KU365780/BR/2015
KU365777/BR/2015
KY014297/BR/2016
KY785450/BR/2016
MH513600/BR/2015
KU729217/BR/2015
KY120352/KR/BR/2016
MH882544/BR/2016
MH882545/BR/2016
MH882543/BR/2016
MH882542/BR/2016
MH882527/BR/2016
MH882535/BR/2016
MH882534/BR/2016
MH882540/BR/2016
MH882533/BR/2016
MH882531/BR/2016
MH882538/BR/2016
KY631492/BR/2016
KU497555/BR/2015
KY785455/BR/2016
KU940228/BR/2015
KX520666/BR/2015
KY441401/BR/2016
KX197192/BR/2015
MF352141/BR/2015
KX421193/UG/1947
KX830960/UG/1947
KX377335/UG/1947
LC002520/UG/1947
KY989511/UG/1947
KU963573/UG/1947
KU955594/UG/1947
MK105975/UG/1947
KX601169/UG/1947
DQ859059/UG/1947

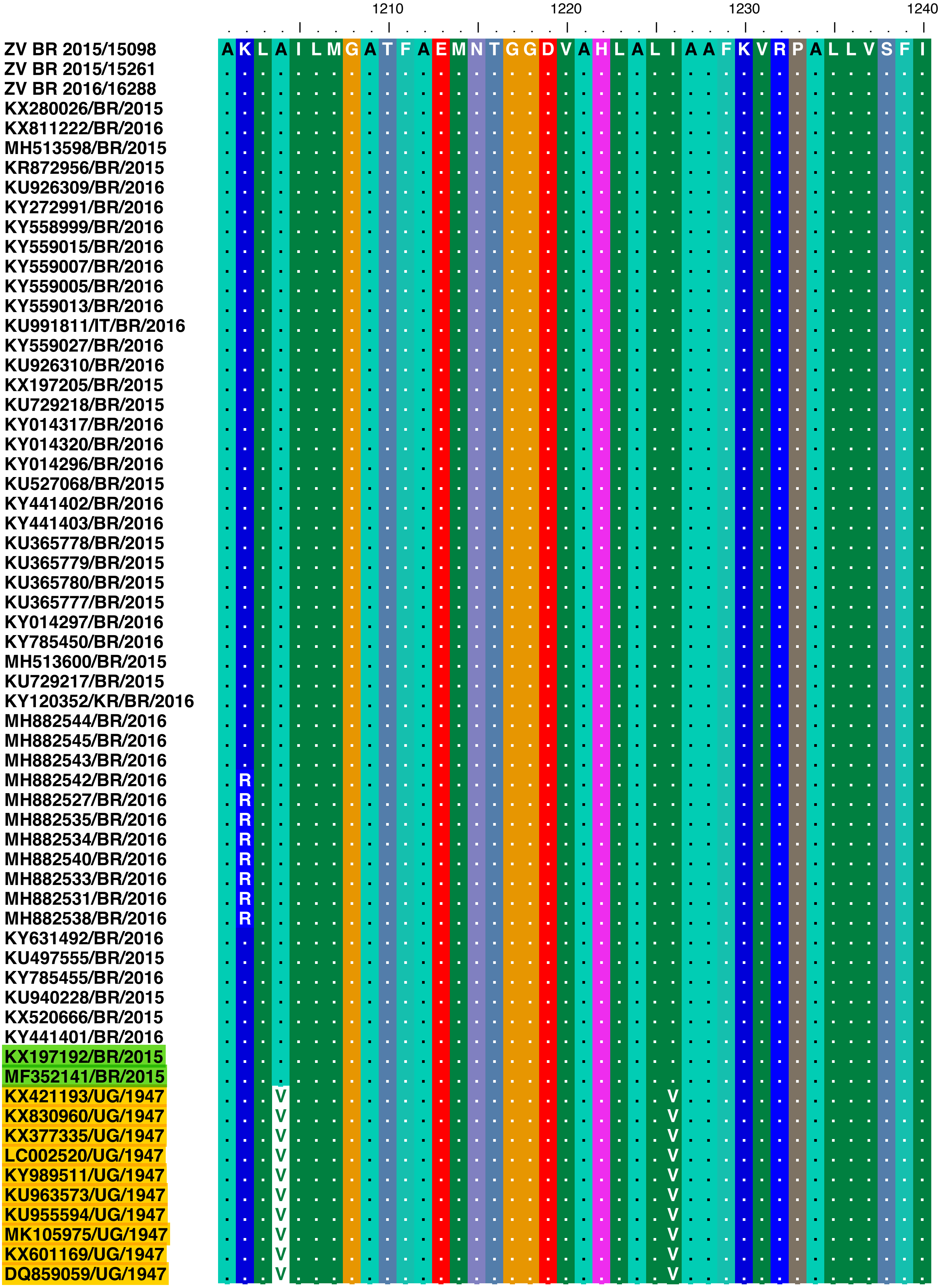
1210
1220
1230
1240
A K L A I L M G A T F A E M N T G G D V A H L A L I A A F K V R P A L L V S F I
ZV BR 2015/15098
ZV BR 2015/15261
ZV BR 2016/16288
KX280026/BR/2015
KX811222/BR/2016
MH513598/BR/2015
KR872956/BR/2015
KU926309/BR/2016
KY272991/BR/2016
KY558999/BR/2016
KY559015/BR/2016
KY559007/BR/2016
KY559005/BR/2016
KY559013/BR/2016
KU991811/IT/BR/2016
KY559027/BR/2016
KU926310/BR/2016
KX197205/BR/2015
KU729218/BR/2015
KY014317/BR/2016
KY014320/BR/2016
KY014296/BR/2016
KU527068/BR/2015
KY441402/BR/2016
KY441403/BR/2016
KU365778/BR/2015
KU365779/BR/2015
KU365780/BR/2015
KU365777/BR/2015
KY014297/BR/2016
KY785450/BR/2016
MH513600/BR/2015
KU729217/BR/2015
KY120352/KR/BR/2016
MH882544/BR/2016
MH882545/BR/2016
MH882543/BR/2016
MH882542/BR/2016
MH882527/BR/2016
MH882535/BR/2016
MH882534/BR/2016
MH882540/BR/2016
MH882533/BR/2016
MH882531/BR/2016
MH882538/BR/2016
KY631492/BR/2016
KU497555/BR/2015
KY785455/BR/2016
KU940228/BR/2015
KX520666/BR/2015
KY441401/BR/2016
KX197192/BR/2015
MF352141/BR/2015
KX421193/UG/1947
KX830960/UG/1947
KX377335/UG/1947
LC002520/UG/1947
KY989511/UG/1947
KU963573/UG/1947
KU955594/UG/1947
MK105975/UG/1947
KX601169/UG/1947
DQ859059/UG/1947

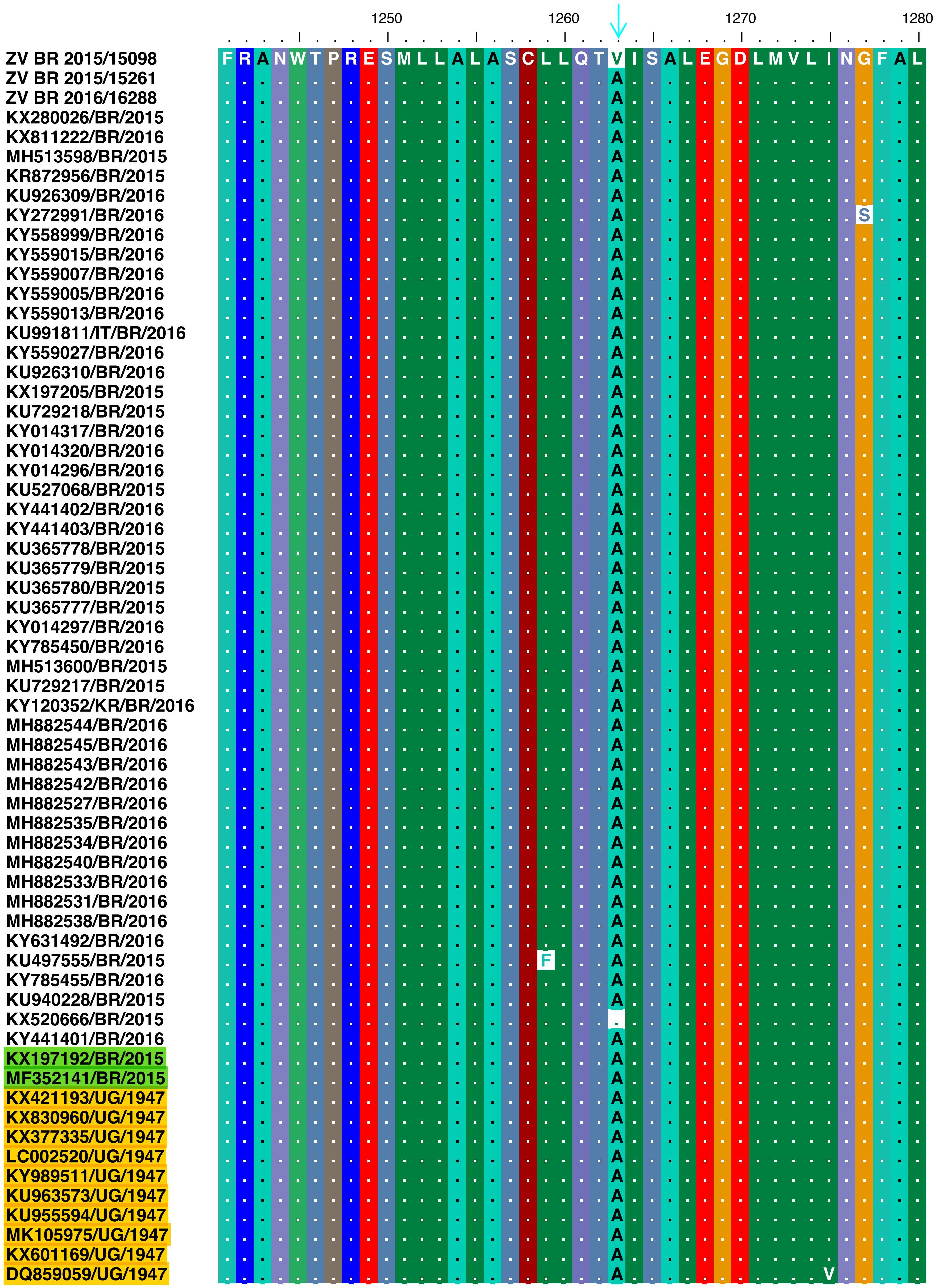

1250
1260
1270
1280
FRANWTPRESMLLALASCLLQTVISALEGDLMVLINGFAL
ZV BR 2015/15098
ZV BR 2015/15261
ZV BR 2016/16288
KX280026/BR/2015
KX811222/BR/2016
MH513598/BR/2015
KR872956/BR/2015
KU926309/BR/2016
KY272991/BR/2016
KY558999/BR/2016
KY559015/BR/2016
KY559007/BR/2016
KY559005/BR/2016
KY559013/BR/2016
KU991811/IT/BR/2016
KY559027/BR/2016
KU926310/BR/2016
KX197205/BR/2015
KU729218/BR/2015
KY014317/BR/2016
KY014320/BR/2016
KY014296/BR/2016
KU527068/BR/2015
KY441402/BR/2016
KY441403/BR/2016
KU365778/BR/2015
KU365779/BR/2015
KU365780/BR/2015
KU365777/BR/2015
KY014297/BR/2016
KY785450/BR/2016
MH513600/BR/2015
KU729217/BR/2015
KY120352/KR/BR/2016
MH882544/BR/2016
MH882545/BR/2016
MH882543/BR/2016
MH882542/BR/2016
MH882527/BR/2016
MH882535/BR/2016
MH882534/BR/2016
MH882540/BR/2016
MH882533/BR/2016
MH882531/BR/2016
MH882538/BR/2016
KY631492/BR/2016
KU497555/BR/2015
KY785455/BR/2016
KU940228/BR/2015
KX520666/BR/2015
KY441401/BR/2016
KX197192/BR/2015
MF352141/BR/2015
KX421193/UG/1947
KX830960/UG/1947
KX377335/UG/1947
LC002520/UG/1947
KY989511/UG/1947
KU963573/UG/1947
KU955594/UG/1947
MK105975/UG/1947
KX601169/UG/1947
DQ859059/UG/1947

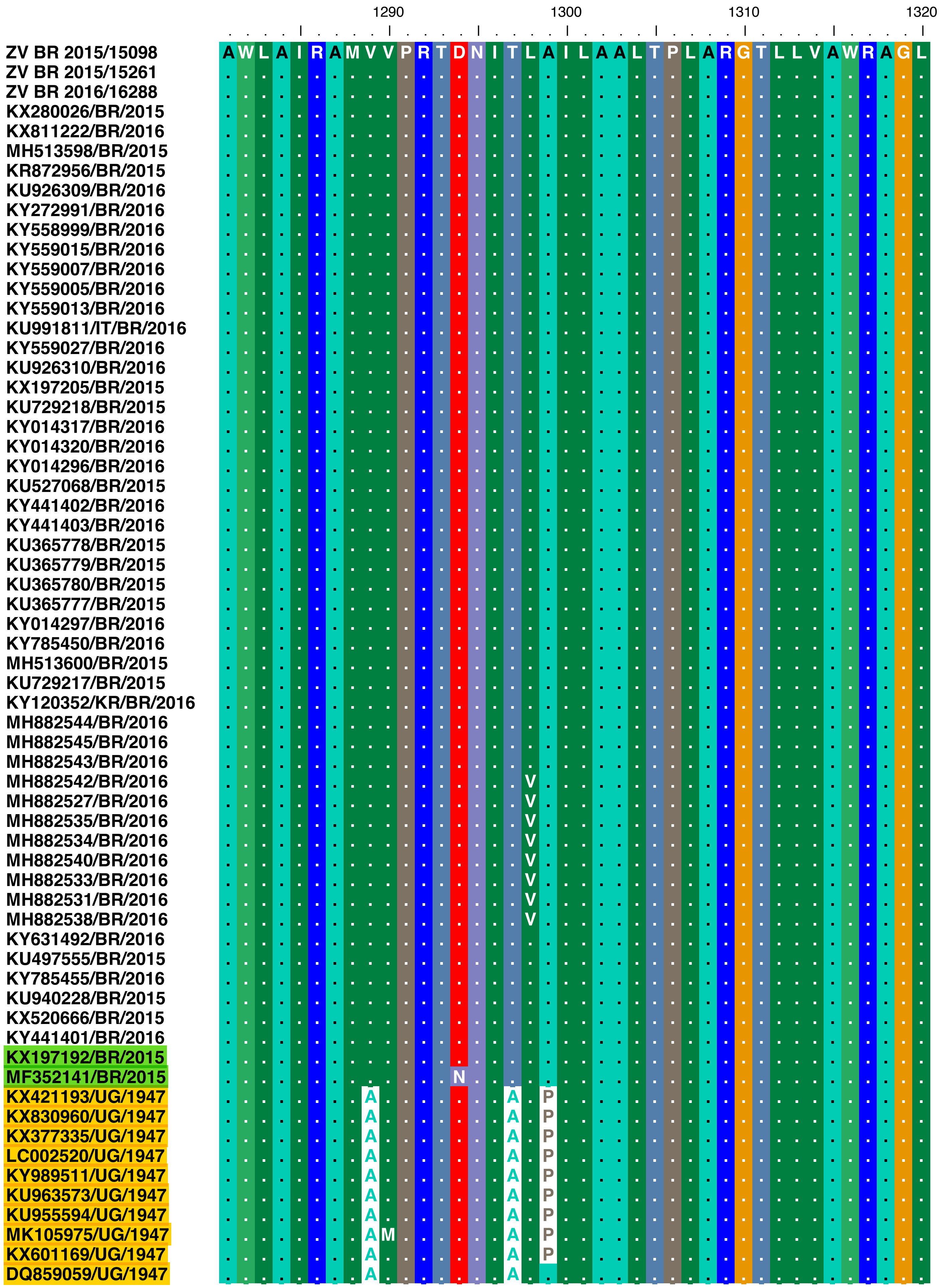
1290
1300
1310
1320
A W L A I R A M V V P R T D N I T L A I L A A L T P L A R G T L L V A W R A G L
ZV BR 2015/15098
ZV BR 2015/15261
ZV BR 2016/16288
KX280026/BR/2015
KX811222/BR/2016
MH513598/BR/2015
KR872956/BR/2015
KU926309/BR/2016
KY272991/BR/2016
KY558999/BR/2016
KY559015/BR/2016
KY559007/BR/2016
KY559005/BR/2016
KY559013/BR/2016
KU991811/IT/BR/2016
KY559027/BR/2016
KU926310/BR/2016
KX197205/BR/2015
KU729218/BR/2015
KY014317/BR/2016
KY014320/BR/2016
KY014296/BR/2016
KU527068/BR/2015
KY441402/BR/2016
KY441403/BR/2016
KU365778/BR/2015
KU365779/BR/2015
KU365780/BR/2015
KU365777/BR/2015
KY014297/BR/2016
KY785450/BR/2016
MH513600/BR/2015
KU729217/BR/2015
KY120352/KR/BR/2016
MH882544/BR/2016
MH882545/BR/2016
MH882543/BR/2016
MH882542/BR/2016
MH882527/BR/2016
MH882535/BR/2016
MH882534/BR/2016
MH882540/BR/2016
MH882533/BR/2016
MH882531/BR/2016
MH882538/BR/2016
KY631492/BR/2016
KU497555/BR/2015
KY785455/BR/2016
KU940228/BR/2015
KX520666/BR/2015
KY441401/BR/2016
KX197192/BR/2015
MF352141/BR/2015
KX421193/UG/1947
KX830960/UG/1947
KX377335/UG/1947
LC002520/UG/1947
KY989511/UG/1947
KU963573/UG/1947
KU955594/UG/1947
MK105975/UG/1947
KX601169/UG/1947
DQ859059/UG/1947

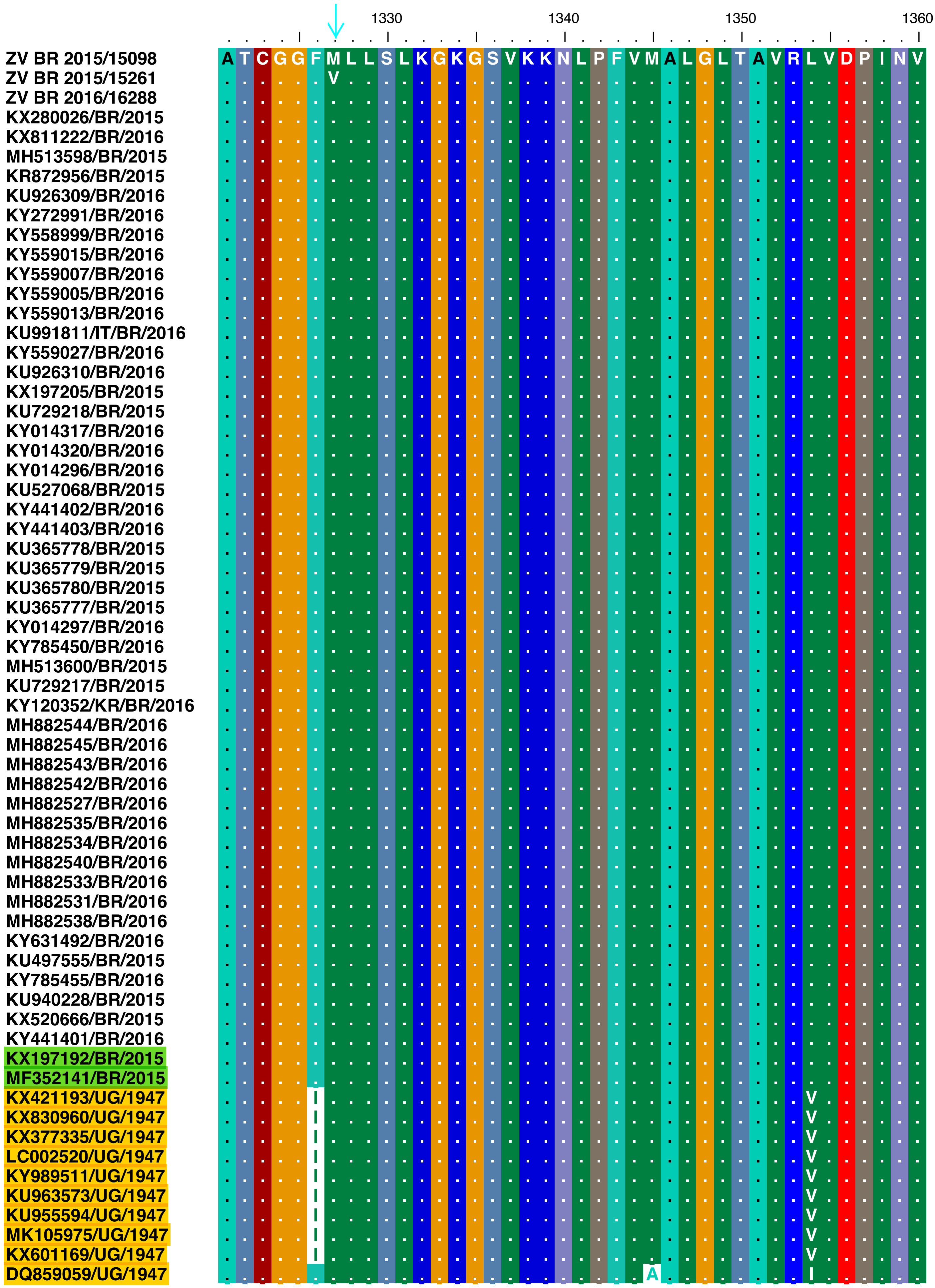
1330
1340
1350
1360
ATCGGFMLLSLKGKGSVKKNLPFVMALGLTAVRLVDPINV
ZV BR 2015/15098
ZV BR 2015/15261
ZV BR 2016/16288
KX280026/BR/2015
KX811222/BR/2016
MH513598/BR/2015
KR872956/BR/2015
KU926309/BR/2016
KY272991/BR/2016
KY558999/BR/2016
KY559015/BR/2016
KY559007/BR/2016
KY559005/BR/2016
KY559013/BR/2016
KU991811/IT/BR/2016
KY559027/BR/2016
KU926310/BR/2016
KX197205/BR/2015
KU729218/BR/2015
KY014317/BR/2016
KY014320/BR/2016
KY014296/BR/2016
KU527068/BR/2015
KY441402/BR/2016
KY441403/BR/2016
KU365778/BR/2015
KU365779/BR/2015
KU365780/BR/2015
KU365777/BR/2015
KY014297/BR/2016
KY785450/BR/2016
MH513600/BR/2015
KU729217/BR/2015
KY120352/KR/BR/2016
MH882544/BR/2016
MH882545/BR/2016
MH882543/BR/2016
MH882542/BR/2016
MH882527/BR/2016
MH882535/BR/2016
MH882534/BR/2016
MH882540/BR/2016
MH882533/BR/2016
MH882531/BR/2016
MH882538/BR/2016
KY631492/BR/2016
KU497555/BR/2015
KY785455/BR/2016
KU940228/BR/2015
KX520666/BR/2015
KY441401/BR/2016
KX197192/BR/2015
MF352141/BR/2015
KX421193/UG/1947
KX830960/UG/1947
KX377335/UG/1947
LC002520/UG/1947
KY989511/UG/1947
KU963573/UG/1947
KU955594/UG/1947
MK105975/UG/1947
KX601169/UG/1947
DQ859059/UG/1947

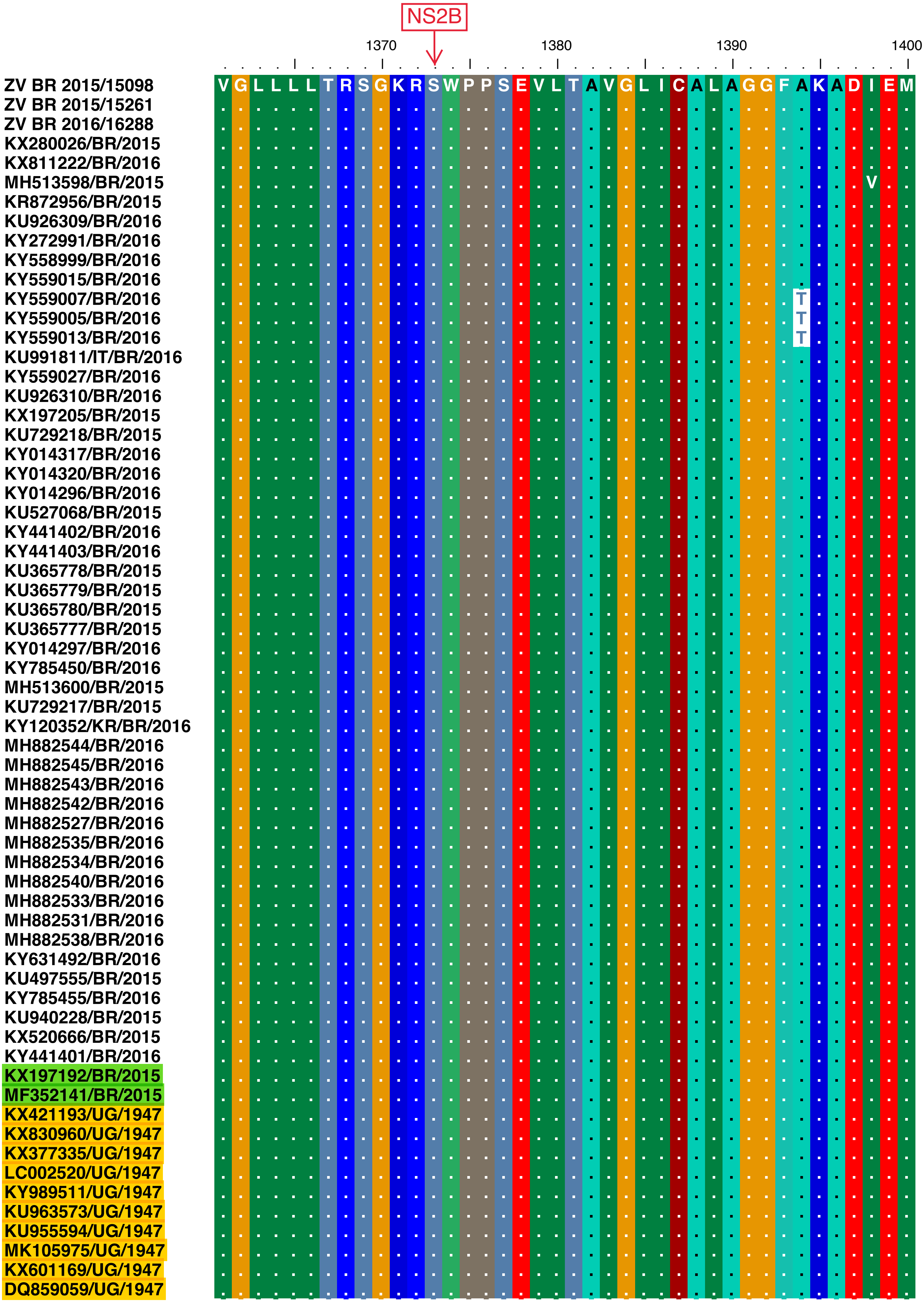
NS2B
1370
1380
1390
1400
V G L L L L T R S G K R S W P P S E V L T A V G L I C A L A G G F A K A D I E M
ZV BR 2015/15098
ZV BR 2015/15261
ZV BR 2016/16288
KX280026/BR/2015
KX811222/BR/2016
MH513598/BR/2015
V
KR872956/BR/2015
KU926309/BR/2016
KY272991/BR/2016
KY558999/BR/2016
KY559015/BR/2016
KY559007/BR/2016
T
KY559005/BR/2016
T
KY559013/BR/2016
T
KU991811/IT/BR/2016
KY559027/BR/2016
KU926310/BR/2016
KX197205/BR/2015
KU729218/BR/2015
KY014317/BR/2016
KY014320/BR/2016
KY014296/BR/2016
KU527068/BR/2015
KY441402/BR/2016
KY441403/BR/2016
KU365778/BR/2015
KU365779/BR/2015
KU365780/BR/2015
KU365777/BR/2015
KY014297/BR/2016
KY785450/BR/2016
MH513600/BR/2015
KU729217/BR/2015
KY120352/KR/BR/2016
MH882544/BR/2016
MH882545/BR/2016
MH882543/BR/2016
MH882542/BR/2016
MH882527/BR/2016
MH882535/BR/2016
MH882534/BR/2016
MH882540/BR/2016
MH882533/BR/2016
MH882531/BR/2016
MH882538/BR/2016
KY631492/BR/2016
KU497555/BR/2015
KY785455/BR/2016
KU940228/BR/2015
KX520666/BR/2015
KY441401/BR/2016
KX197192/BR/2015
MF352141/BR/2015
KX421193/UG/1947
KX830960/UG/1947
KX377335/UG/1947
LC002520/UG/1947
KY989511/UG/1947
KU963573/UG/1947
KU955594/UG/1947
MK105975/UG/1947
KX601169/UG/1947
DQ859059/UG/1947

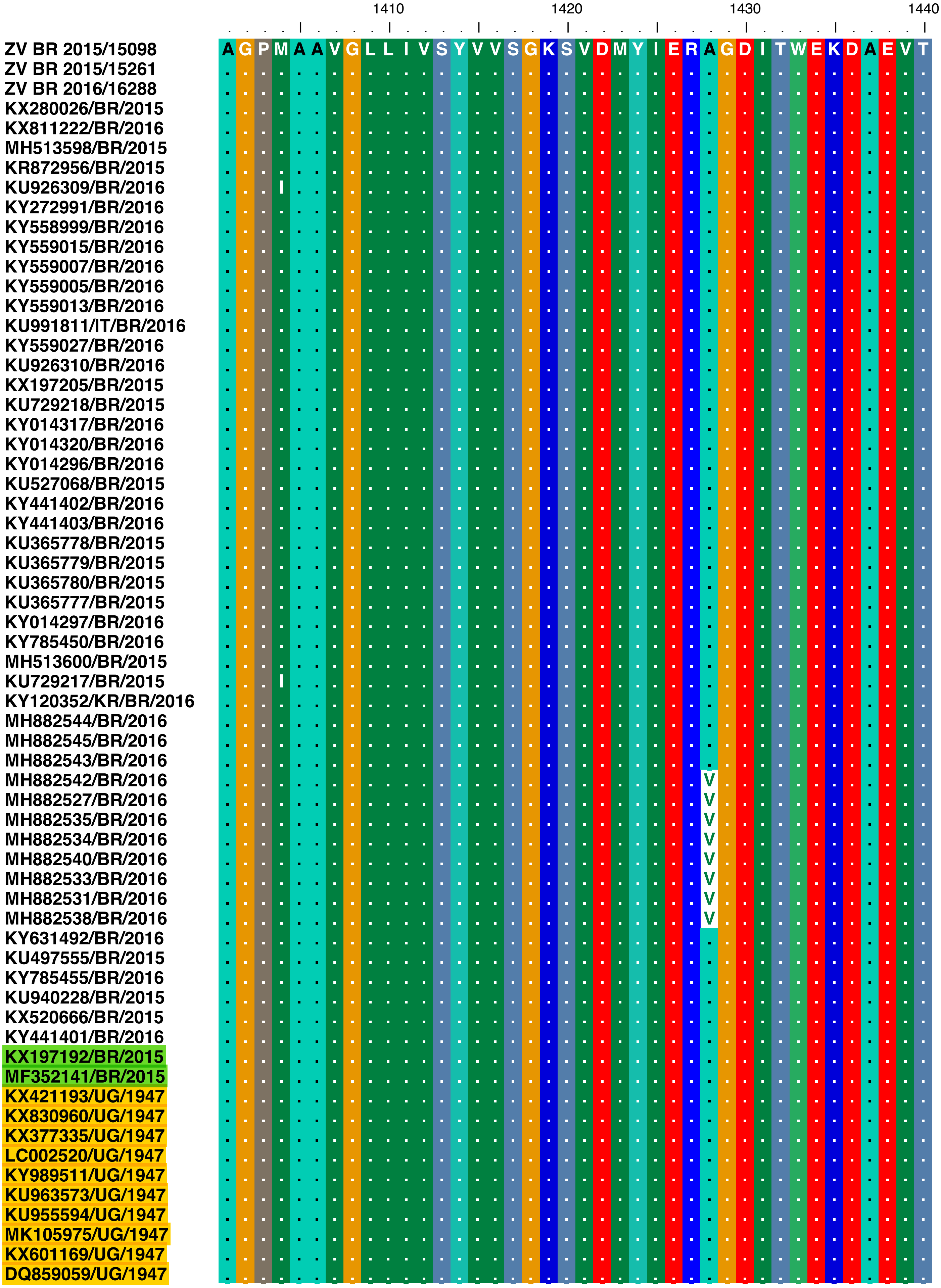

1410
1420
1430
1440
ZV BR 2015/15098 A G P M A A V G L L I V S Y V V S G K S V D M Y I E R A G D I T W E K D A E V T
ZV BR 2015/15261
ZV BR 2016/16288
KX280026/BR/2015
KX811222/BR/2016
MH513598/BR/2015
KR872956/BR/2015
KU926309/BR/2016 I
KY272991/BR/2016
KY558999/BR/2016
KY559015/BR/2016
KY559007/BR/2016
KY559005/BR/2016
KY559013/BR/2016
KU991811/IT/BR/2016
KY559027/BR/2016
KU926310/BR/2016
KX197205/BR/2015
KU729218/BR/2015
KY014317/BR/2016
KY014320/BR/2016
KY014296/BR/2016
KU527068/BR/2015
KY441402/BR/2016
KY441403/BR/2016
KU365778/BR/2015
KU365779/BR/2015
KU365780/BR/2015
KU365777/BR/2015
KY014297/BR/2016
KY785450/BR/2016
MH513600/BR/2015
KU729217/BR/2015 I
KY120352/KR/BR/2016
MH882544/BR/2016
MH882545/BR/2016
MH882543/BR/2016
MH882542/BR/2016 V
MH882527/BR/2016 V
MH882535/BR/2016 V
MH882534/BR/2016 V
MH882540/BR/2016 V
MH882533/BR/2016 V
MH882531/BR/2016 V
MH882538/BR/2016 V
KY631492/BR/2016
KU497555/BR/2015
KY785455/BR/2016
KU940228/BR/2015
KX520666/BR/2015
KY441401/BR/2016
KX197192/BR/2015
MF352141/BR/2015
KX421193/UG/1947
KX830960/UG/1947
KX377335/UG/1947
LC002520/UG/1947
KY989511/UG/1947
KU963573/UG/1947
KU955594/UG/1947
MK105975/UG/1947
KX601169/UG/1947
DQ859059/UG/1947

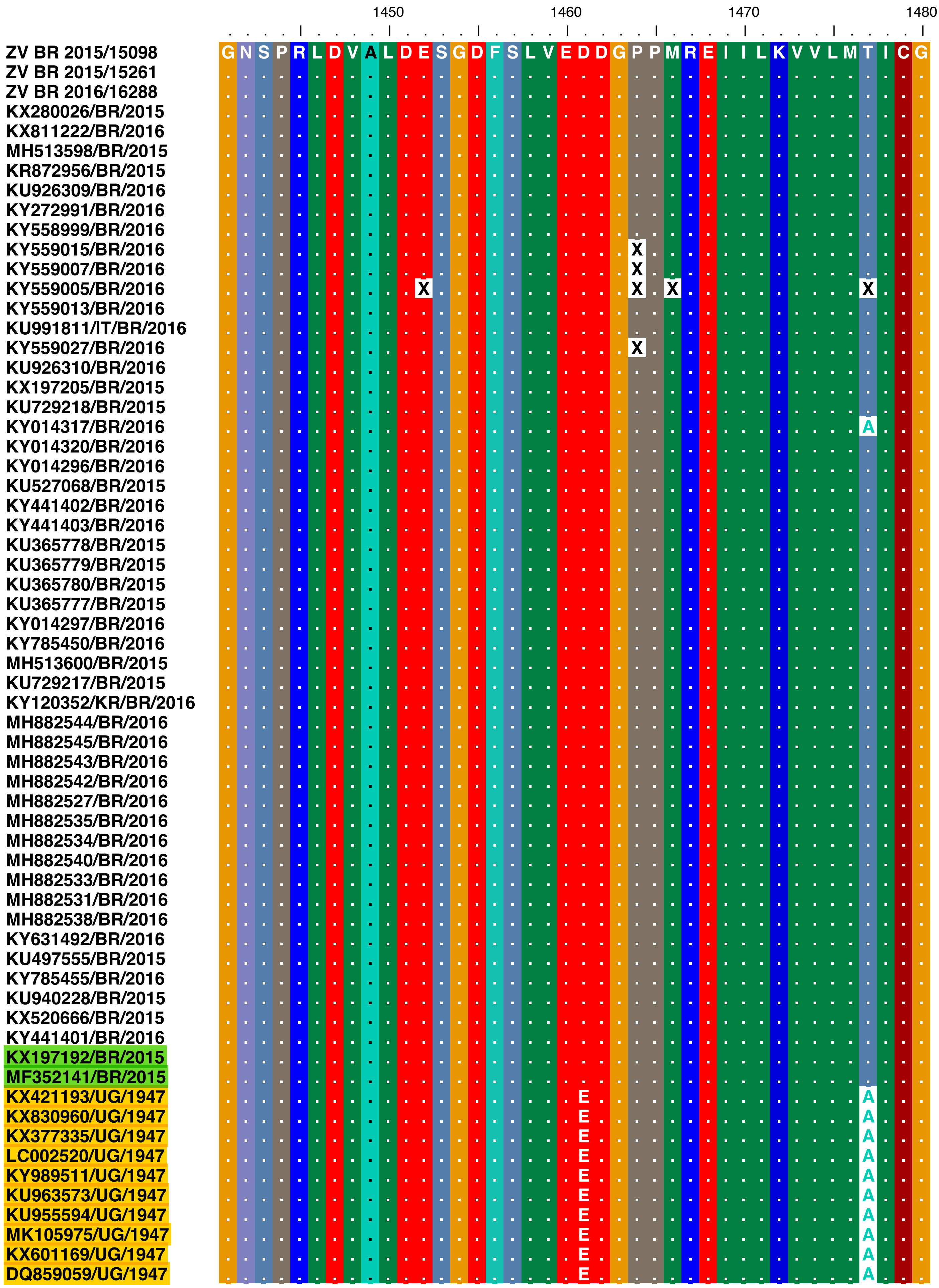

1450
1460
1470
1480
G N S P R L D V A L D E S G D F S L V E D D G P P M R E I I L K V V L M T I C G
ZV BR 2015/15098
ZV BR 2015/15261
ZV BR 2016/16288
KX280026/BR/2015
KX811222/BR/2016
MH513598/BR/2015
KR872956/BR/2015
KU926309/BR/2016
KY272991/BR/2016
KY558999/BR/2016
KY559015/BR/2016
KY559007/BR/2016
KY559005/BR/2016
KY559013/BR/2016
KU991811/IT/BR/2016
KY559027/BR/2016
KU926310/BR/2016
KX197205/BR/2015
KU729218/BR/2015
KY014317/BR/2016
KY014320/BR/2016
KY014296/BR/2016
KU527068/BR/2015
KY441402/BR/2016
KY441403/BR/2016
KU365778/BR/2015
KU365779/BR/2015
KU365780/BR/2015
KU365777/BR/2015
KY014297/BR/2016
KY785450/BR/2016
MH513600/BR/2015
KU729217/BR/2015
KY120352/KR/BR/2016
MH882544/BR/2016
MH882545/BR/2016
MH882543/BR/2016
MH882542/BR/2016
MH882527/BR/2016
MH882535/BR/2016
MH882534/BR/2016
MH882540/BR/2016
MH882533/BR/2016
MH882531/BR/2016
MH882538/BR/2016
KY631492/BR/2016
KU497555/BR/2015
KY785455/BR/2016
KU940228/BR/2015
KX520666/BR/2015
KY441401/BR/2016
KX197192/BR/2015
MF352141/BR/2015
KX421193/UG/1947
KX830960/UG/1947
KX377335/UG/1947
LC002520/UG/1947
KY989511/UG/1947
KU963573/UG/1947
KU955594/UG/1947
MK105975/UG/1947
KX601169/UG/1947
DQ859059/UG/1947

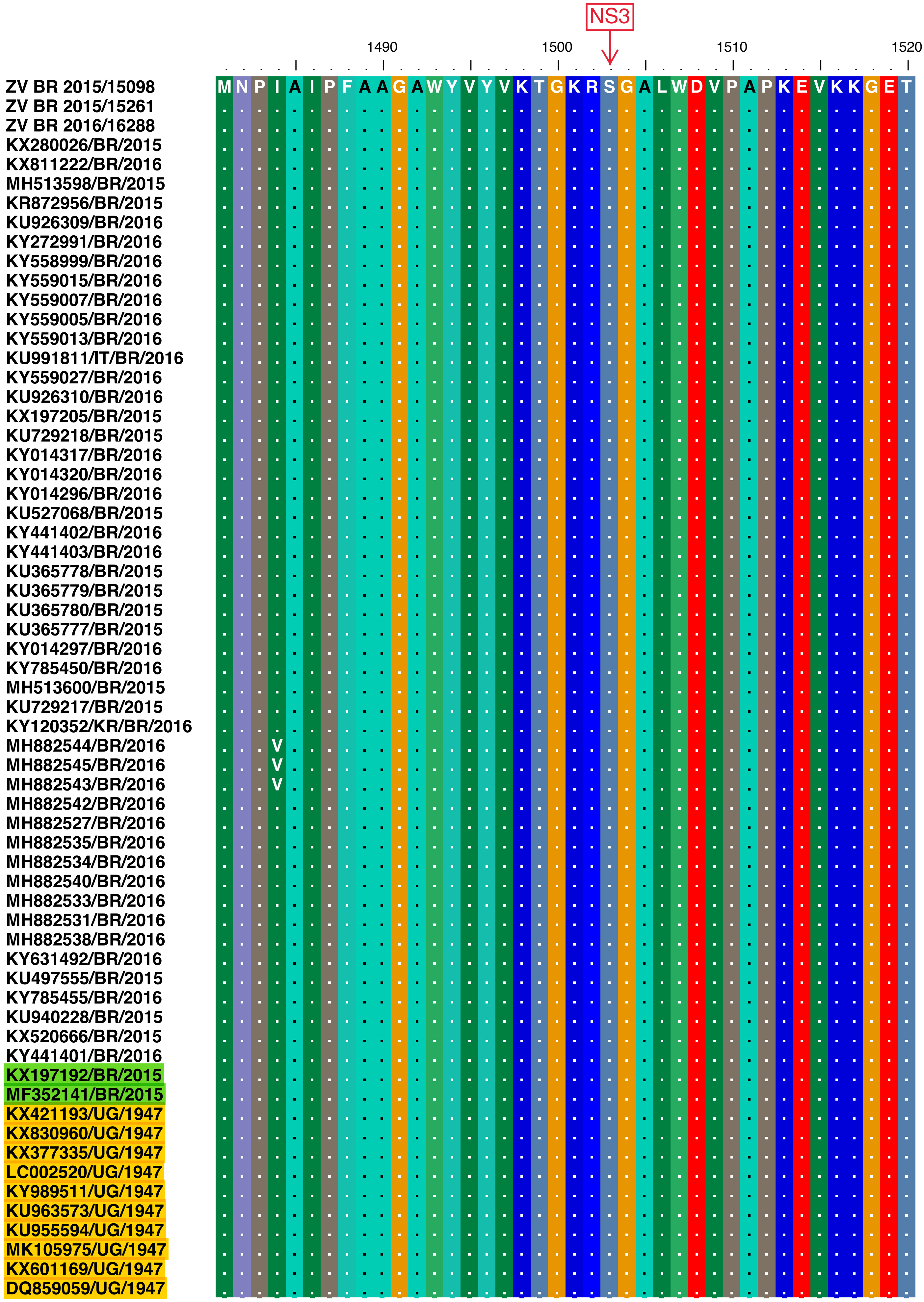
NS3
1490
1500
1510
1520
MNPIAIPFAAGAWYVYVKTGKRSGALWDVPAPKEVKKGET
ZV BR 2015/15098
ZV BR 2015/15261
ZV BR 2016/16288
KX280026/BR/2015
KX811222/BR/2016
MH513598/BR/2015
KR872956/BR/2015
KU926309/BR/2016
KY272991/BR/2016
KY558999/BR/2016
KY559015/BR/2016
KY559007/BR/2016
KY559005/BR/2016
KY559013/BR/2016
KU991811/IT/BR/2016
KY559027/BR/2016
KU926310/BR/2016
KX197205/BR/2015
KU729218/BR/2015
KY014317/BR/2016
KY014320/BR/2016
KY014296/BR/2016
KU527068/BR/2015
KY441402/BR/2016
KY441403/BR/2016
KU365778/BR/2015
KU365779/BR/2015
KU365780/BR/2015
KU365777/BR/2015
KY014297/BR/2016
KY785450/BR/2016
MH513600/BR/2015
KU729217/BR/2015
KY120352/KR/BR/2016
MH882544/BR/2016 V
MH882545/BR/2016 V
MH882543/BR/2016 V
MH882542/BR/2016
MH882527/BR/2016
MH882535/BR/2016
MH882534/BR/2016
MH882540/BR/2016
MH882533/BR/2016
MH882531/BR/2016
MH882538/BR/2016
KY631492/BR/2016
KU497555/BR/2015
KY785455/BR/2016
KU940228/BR/2015
KX520666/BR/2015
KY441401/BR/2016
KX197192/BR/2015
MF352141/BR/2015
KX421193/UG/1947
KX830960/UG/1947
KX377335/UG/1947
LC002520/UG/1947
KY989511/UG/1947
KU963573/UG/1947
KU955594/UG/1947
MK105975/UG/1947
KX601169/UG/1947
DQ859059/UG/1947

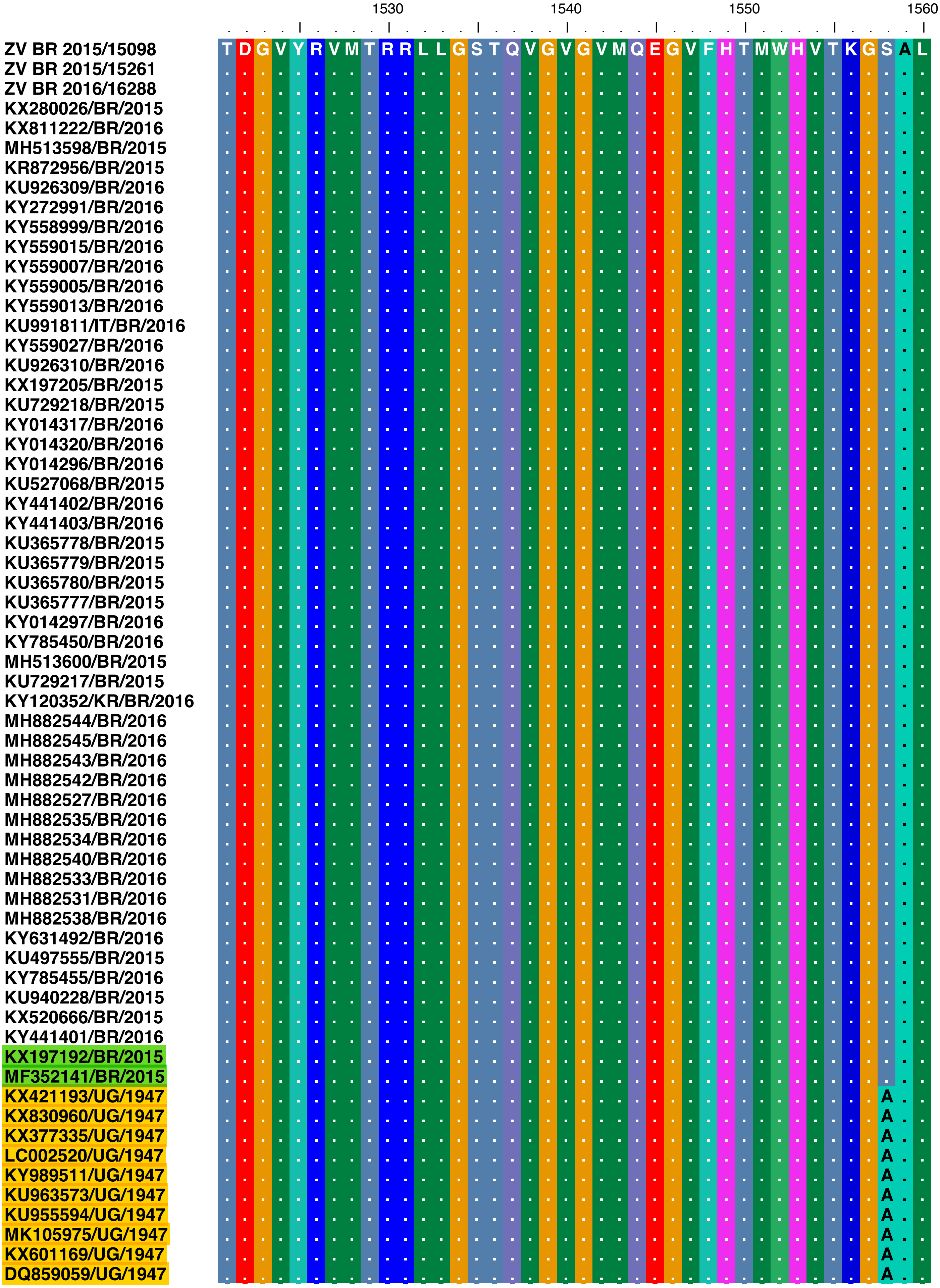
1530
1540
1550
1560
T D G V Y R V M T R R L L G S T Q V G V G V M Q E G V F H T M W H V T K G S A L
ZV BR 2015/15098
ZV BR 2015/15261
ZV BR 2016/16288
KX280026/BR/2015
KX811222/BR/2016
MH513598/BR/2015
KR872956/BR/2015
KU926309/BR/2016
KY272991/BR/2016
KY558999/BR/2016
KY559015/BR/2016
KY559007/BR/2016
KY559005/BR/2016
KY559013/BR/2016
KU991811/IT/BR/2016
KY559027/BR/2016
KU926310/BR/2016
KX197205/BR/2015
KU729218/BR/2015
KY014317/BR/2016
KY014320/BR/2016
KY014296/BR/2016
KU527068/BR/2015
KY441402/BR/2016
KY441403/BR/2016
KU365778/BR/2015
KU365779/BR/2015
KU365780/BR/2015
KU365777/BR/2015
KY014297/BR/2016
KY785450/BR/2016
MH513600/BR/2015
KU729217/BR/2015
KY120352/KR/BR/2016
MH882544/BR/2016
MH882545/BR/2016
MH882543/BR/2016
MH882542/BR/2016
MH882527/BR/2016
MH882535/BR/2016
MH882534/BR/2016
MH882540/BR/2016
MH882533/BR/2016
MH882531/BR/2016
MH882538/BR/2016
KY631492/BR/2016
KU497555/BR/2015
KY785455/BR/2016
KU940228/BR/2015
KX520666/BR/2015
KY441401/BR/2016
KX197192/BR/2015
MF352141/BR/2015
KX421193/UG/1947
KX830960/UG/1947
KX377335/UG/1947
LC002520/UG/1947
KY989511/UG/1947
KU963573/UG/1947
KU955594/UG/1947
MK105975/UG/1947
KX601169/UG/1947
DQ859059/UG/1947
A

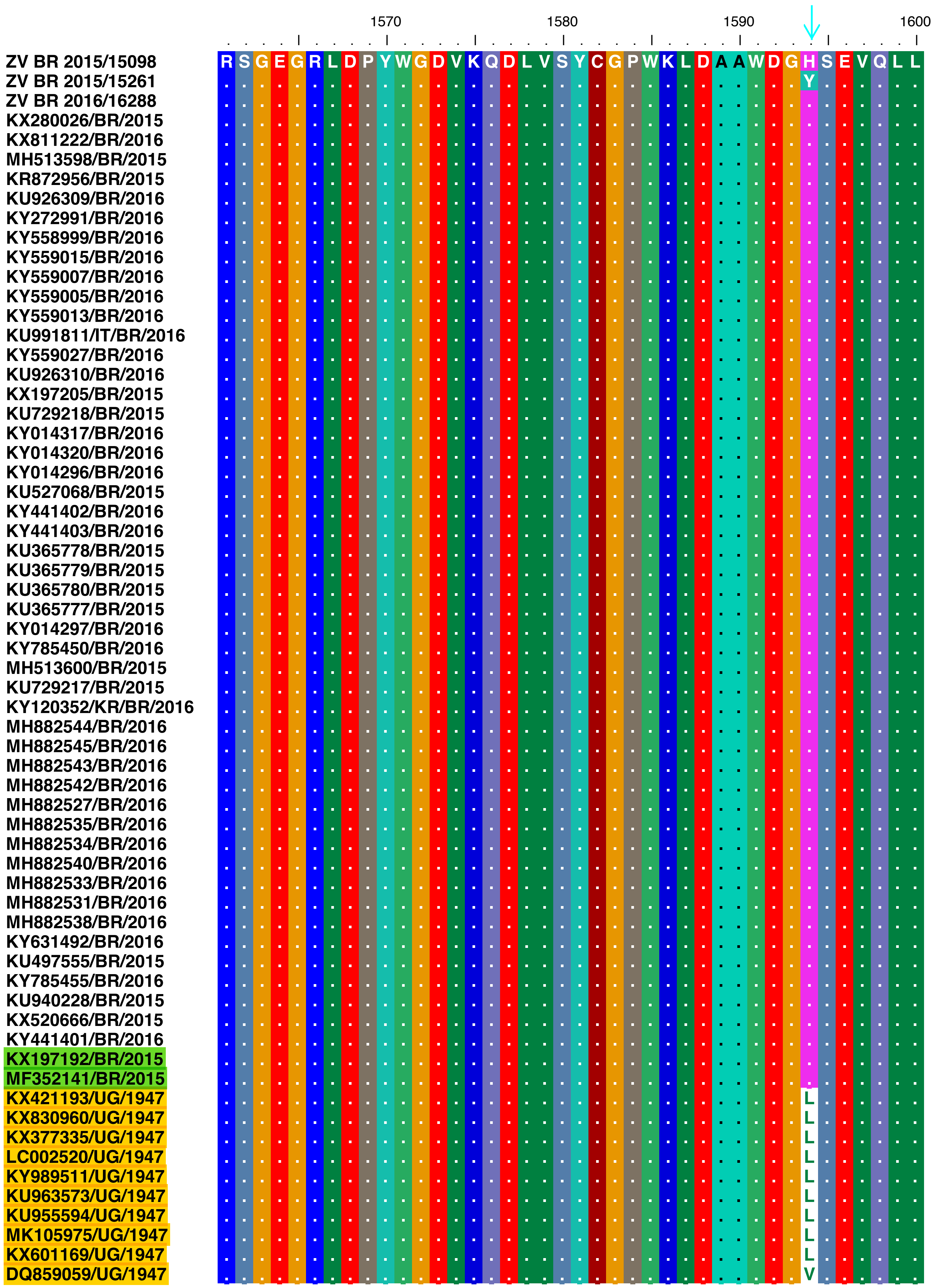

1570
1580
1590
1600
ZV BR 2015/15098
R S G E G R L D P Y W G D V K Q D L V S Y C G P W K L D A A W D G H S E V Q L L
ZV BR 2015/15261
Y
ZV BR 2016/16288
KX280026/BR/2015
KX811222/BR/2016
MH513598/BR/2015
KR872956/BR/2015
KU926309/BR/2016
KY272991/BR/2016
KY558999/BR/2016
KY559015/BR/2016
KY559007/BR/2016
KY559005/BR/2016
KY559013/BR/2016
KU991811/IT/BR/2016
KY559027/BR/2016
KU926310/BR/2016
KX197205/BR/2015
KU729218/BR/2015
KY014317/BR/2016
KY014320/BR/2016
KY014296/BR/2016
KU527068/BR/2015
KY441402/BR/2016
KY441403/BR/2016
KU365778/BR/2015
KU365779/BR/2015
KU365780/BR/2015
KU365777/BR/2015
KY014297/BR/2016
KY785450/BR/2016
MH513600/BR/2015
KU729217/BR/2015
KY120352/KR/BR/2016
MH882544/BR/2016
MH882545/BR/2016
MH882543/BR/2016
MH882542/BR/2016
MH882527/BR/2016
MH882535/BR/2016
MH882534/BR/2016
MH882540/BR/2016
MH882533/BR/2016
MH882531/BR/2016
MH882538/BR/2016
KY631492/BR/2016
KU497555/BR/2015
KY785455/BR/2016
KU940228/BR/2015
KX520666/BR/2015
KY441401/BR/2016
KX197192/BR/2015
MF352141/BR/2015
KX421193/UG/1947 L
KX830960/UG/1947 L
KX377335/UG/1947 L
LC002520/UG/1947 L
KY989511/UG/1947 L
KU963573/UG/1947 L
KU955594/UG/1947 L
MK105975/UG/1947 L
KX601169/UG/1947 L
DQ859059/UG/1947 V

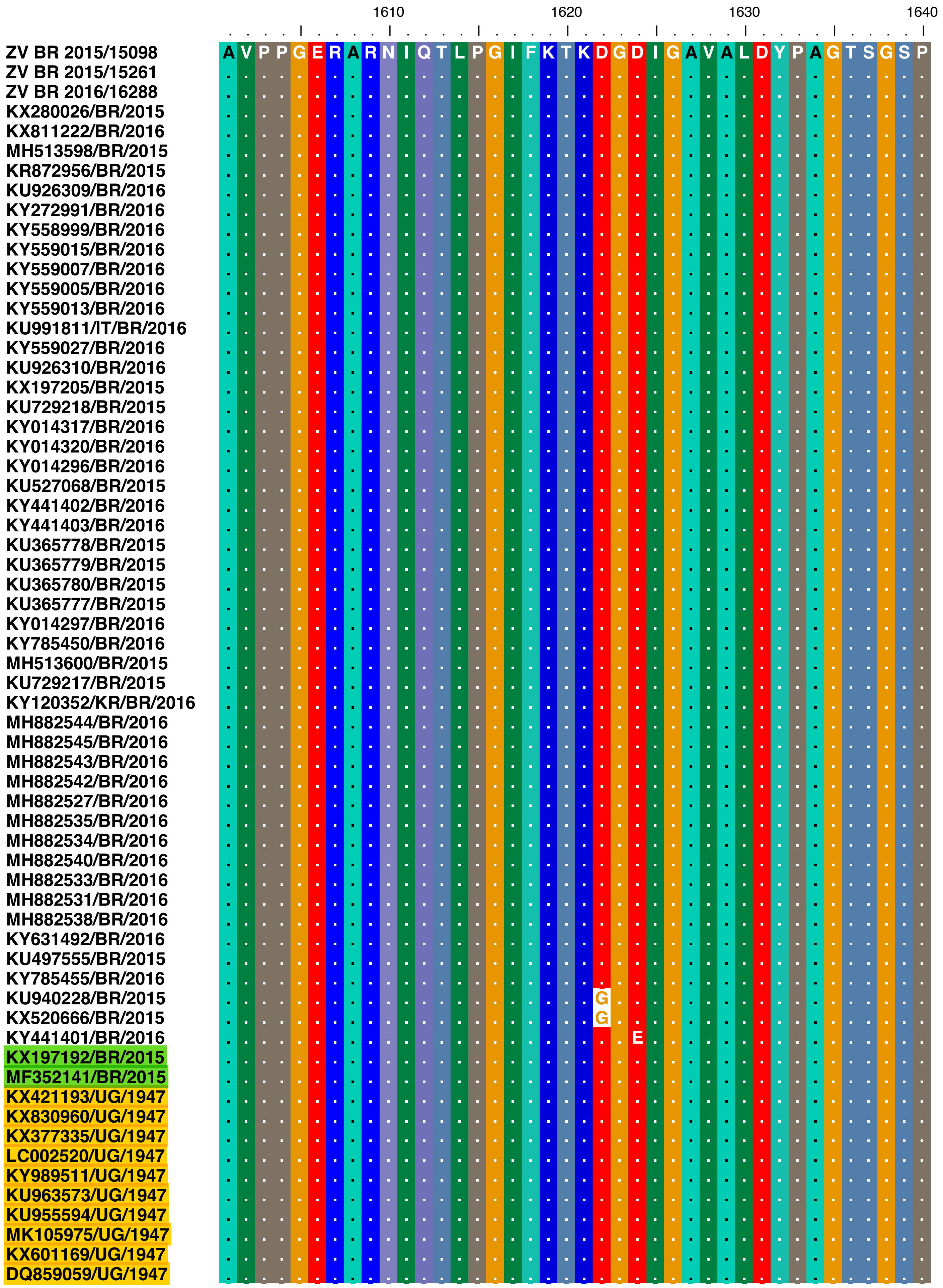
1610
1620
1630
1640
AVPPGERARNIQTLPGIFKTKDGDIGAVALDYPAGTSGSP
ZV BR 2015/15098
ZV BR 2015/15261
ZV BR 2016/16288
KX280026/BR/2015
KX811222/BR/2016
MH513598/BR/2015
KR872956/BR/2015
KU926309/BR/2016
KY272991/BR/2016
KY558999/BR/2016
KY559015/BR/2016
KY559007/BR/2016
KY559005/BR/2016
KY559013/BR/2016
KU991811/IT/BR/2016
KY559027/BR/2016
KU926310/BR/2016
KX197205/BR/2015
KU729218/BR/2015
KY014317/BR/2016
KY014320/BR/2016
KY014296/BR/2016
KU527068/BR/2015
KY441402/BR/2016
KY441403/BR/2016
KU365778/BR/2015
KU365779/BR/2015
KU365780/BR/2015
KU365777/BR/2015
KY014297/BR/2016
KY785450/BR/2016
MH513600/BR/2015
KU729217/BR/2015
KY120352/KR/BR/2016
MH882544/BR/2016
MH882545/BR/2016
MH882543/BR/2016
MH882542/BR/2016
MH882527/BR/2016
MH882535/BR/2016
MH882534/BR/2016
MH882540/BR/2016
MH882533/BR/2016
MH882531/BR/2016
MH882538/BR/2016
KY631492/BR/2016
KU497555/BR/2015
KY785455/BR/2016
KU940228/BR/2015 G
KX520666/BR/2015 G
KY441401/BR/2016 E
KX197192/BR/2015
MF352141/BR/2015
KX421193/UG/1947
KX830960/UG/1947
KX377335/UG/1947
LC002520/UG/1947
KY989511/UG/1947
KU963573/UG/1947
KU955594/UG/1947
MK105975/UG/1947
KX601169/UG/1947
DQ859059/UG/1947

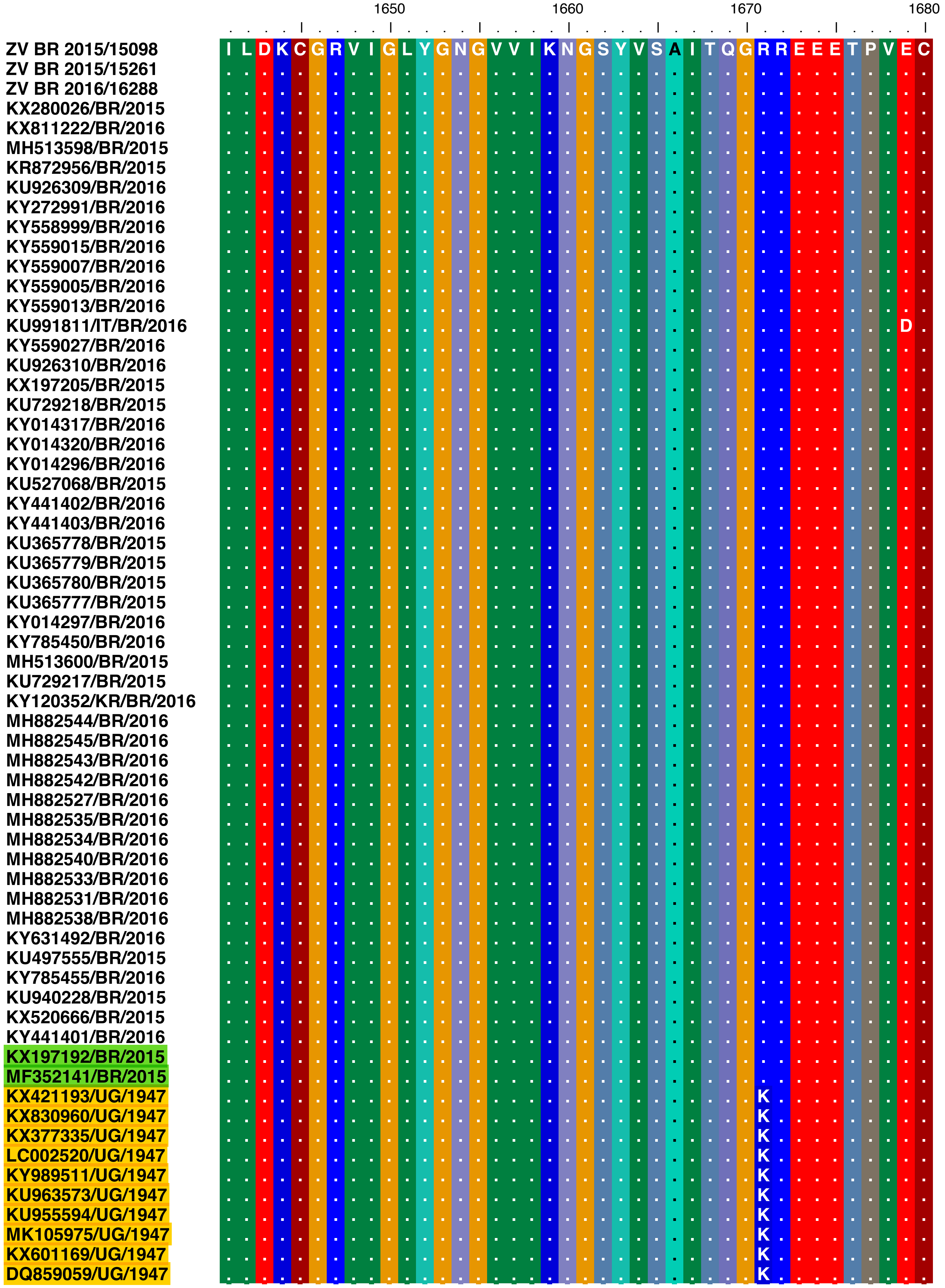
1650
1660
1670
1680
I L D K C G R V I G L Y G N G V V I K N G S Y V S A I T Q G R R E E E T P V E C
ZV BR 2015/15098
ZV BR 2015/15261
ZV BR 2016/16288
KX280026/BR/2015
KX811222/BR/2016
MH513598/BR/2015
KR872956/BR/2015
KU926309/BR/2016
KY272991/BR/2016
KY558999/BR/2016
KY559015/BR/2016
KY559007/BR/2016
KY559005/BR/2016
KY559013/BR/2016
KU991811/IT/BR/2016
D
KY559027/BR/2016
KU926310/BR/2016
KX197205/BR/2015
KU729218/BR/2015
KY014317/BR/2016
KY014320/BR/2016
KY014296/BR/2016
KU527068/BR/2015
KY441402/BR/2016
KY441403/BR/2016
KU365778/BR/2015
KU365779/BR/2015
KU365780/BR/2015
KU365777/BR/2015
KY014297/BR/2016
KY785450/BR/2016
MH513600/BR/2015
KU729217/BR/2015
KY120352/KR/BR/2016
MH882544/BR/2016
MH882545/BR/2016
MH882543/BR/2016
MH882542/BR/2016
MH882527/BR/2016
MH882535/BR/2016
MH882534/BR/2016
MH882540/BR/2016
MH882533/BR/2016
MH882531/BR/2016
MH882538/BR/2016
KY631492/BR/2016
KU497555/BR/2015
KY785455/BR/2016
KU940228/BR/2015
KX520666/BR/2015
KY441401/BR/2016
KX197192/BR/2015
MF352141/BR/2015
KX421193/UG/1947
K
KX830960/UG/1947
K
KX377335/UG/1947
K
LC002520/UG/1947
K
KY989511/UG/1947
K
KU963573/UG/1947
K
KU955594/UG/1947
K
MK105975/UG/1947
K
KX601169/UG/1947
K
DQ859059/UG/1947
K

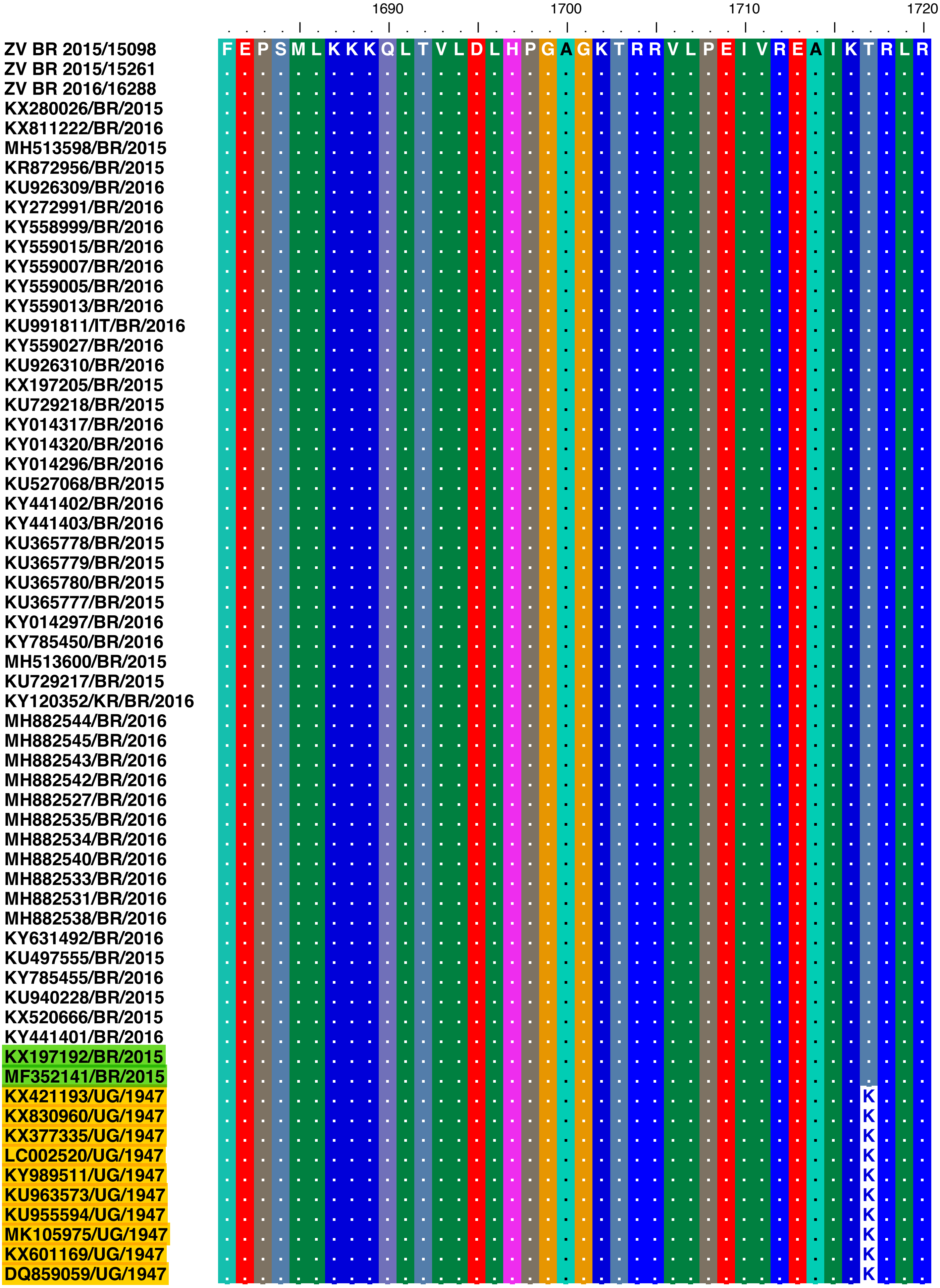
1690
1700
1710
1720
F E P S M L K K K Q L T V L D L H P G A G K T R R V L P E I V R E A I K T R L R
ZV BR 2015/15098
ZV BR 2015/15261
ZV BR 2016/16288
KX280026/BR/2015
KX811222/BR/2016
MH513598/BR/2015
KR872956/BR/2015
KU926309/BR/2016
KY272991/BR/2016
KY558999/BR/2016
KY559015/BR/2016
KY559007/BR/2016
KY559005/BR/2016
KY559013/BR/2016
KU991811/IT/BR/2016
KY559027/BR/2016
KU926310/BR/2016
KX197205/BR/2015
KU729218/BR/2015
KY014317/BR/2016
KY014320/BR/2016
KY014296/BR/2016
KU527068/BR/2015
KY441402/BR/2016
KY441403/BR/2016
KU365778/BR/2015
KU365779/BR/2015
KU365780/BR/2015
KU365777/BR/2015
KY014297/BR/2016
KY785450/BR/2016
MH513600/BR/2015
KU729217/BR/2015
KY120352/KR/BR/2016
MH882544/BR/2016
MH882545/BR/2016
MH882543/BR/2016
MH882542/BR/2016
MH882527/BR/2016
MH882535/BR/2016
MH882534/BR/2016
MH882540/BR/2016
MH882533/BR/2016
MH882531/BR/2016
MH882538/BR/2016
KY631492/BR/2016
KU497555/BR/2015
KY785455/BR/2016
KU940228/BR/2015
KX520666/BR/2015
KY441401/BR/2016
KX197192/BR/2015
MF352141/BR/2015
KX421193/UG/1947 K
KX830960/UG/1947 K
KX377335/UG/1947 K
LC002520/UG/1947 K
KY989511/UG/1947 K
KU963573/UG/1947 K
KU955594/UG/1947 K
MK105975/UG/1947 K
KX601169/UG/1947 K
DQ859059/UG/1947 K

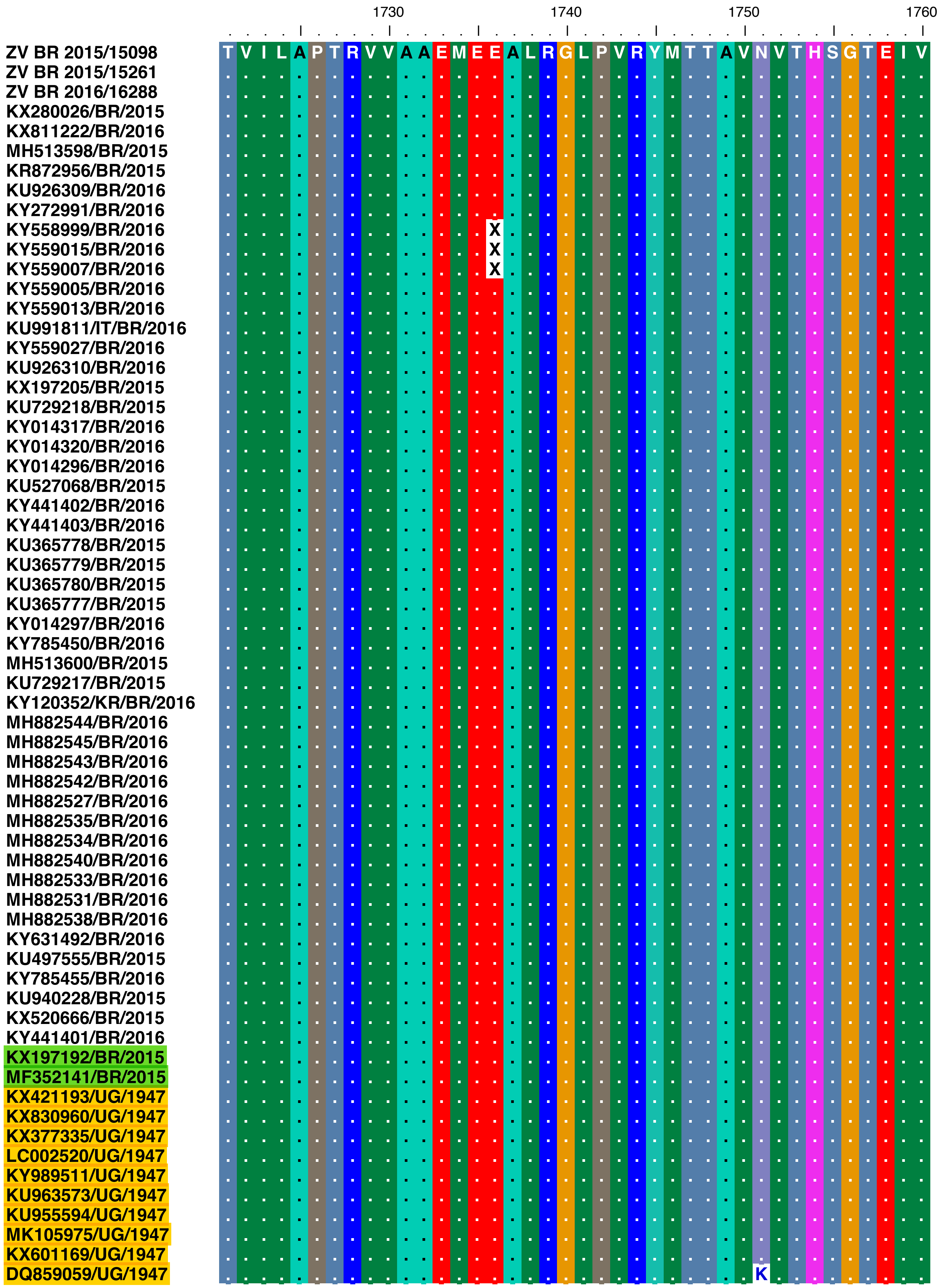
1730
1740
1750
1760
T V I L A P T R V V A A E M E E A L R G L P V R Y M T T A V N V T H S G T E I V
ZV BR 2015/15098
ZV BR 2015/15261
ZV BR 2016/16288
KX280026/BR/2015
KX811222/BR/2016
MH513598/BR/2015
KR872956/BR/2015
KU926309/BR/2016
KY272991/BR/2016
KY558999/BR/2016
KY559015/BR/2016
KY559007/BR/2016
KY559005/BR/2016
KY559013/BR/2016
KU991811/IT/BR/2016
KY559027/BR/2016
KU926310/BR/2016
KX197205/BR/2015
KU729218/BR/2015
KY014317/BR/2016
KY014320/BR/2016
KY014296/BR/2016
KU527068/BR/2015
KY441402/BR/2016
KY441403/BR/2016
KU365778/BR/2015
KU365779/BR/2015
KU365780/BR/2015
KU365777/BR/2015
KY014297/BR/2016
KY785450/BR/2016
MH513600/BR/2015
KU729217/BR/2015
KY120352/KR/BR/2016
MH882544/BR/2016
MH882545/BR/2016
MH882543/BR/2016
MH882542/BR/2016
MH882527/BR/2016
MH882535/BR/2016
MH882534/BR/2016
MH882540/BR/2016
MH882533/BR/2016
MH882531/BR/2016
MH882538/BR/2016
KY631492/BR/2016
KU497555/BR/2015
KY785455/BR/2016
KU940228/BR/2015
KX520666/BR/2015
KY441401/BR/2016
KX197192/BR/2015
MF352141/BR/2015
KX421193/UG/1947
KX830960/UG/1947
KX377335/UG/1947
LC002520/UG/1947
KY989511/UG/1947
KU963573/UG/1947
KU955594/UG/1947
MK105975/UG/1947
KX601169/UG/1947
DQ859059/UG/1947
X
X
X
K

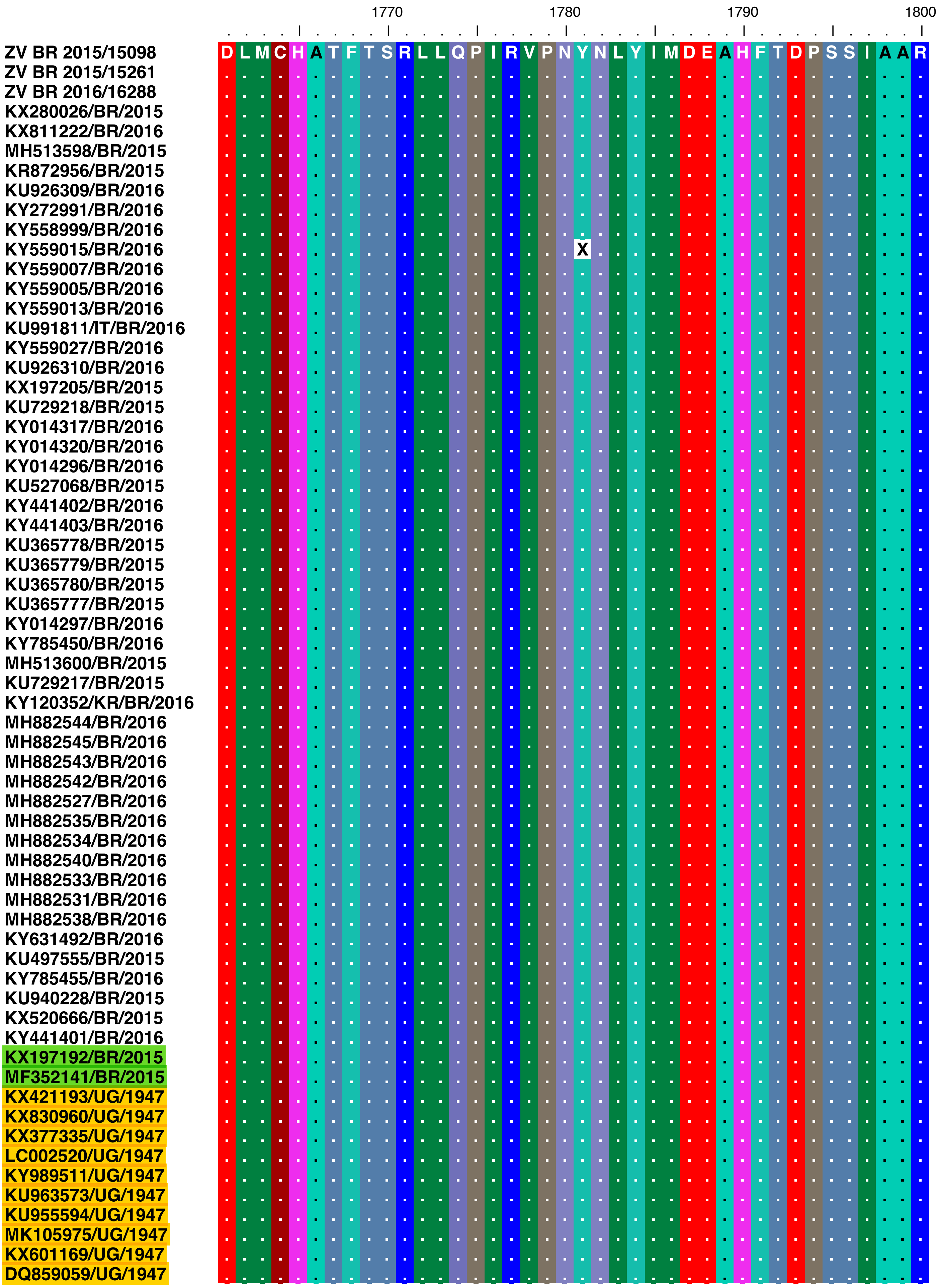

1770
1780
1790
1800
ZV BR 2015/15098 DLMCHATFTSRLLQPIRVPNYNLYIMDEAHFTDPSSIAAR
ZV BR 2015/15261
ZV BR 2016/16288
KX280026/BR/2015
KX811222/BR/2016
MH513598/BR/2015
KR872956/BR/2015
KU926309/BR/2016
KY272991/BR/2016
KY558999/BR/2016
KY559015/BR/2016 X
KY559007/BR/2016
KY559005/BR/2016
KY559013/BR/2016
KU991811/IT/BR/2016
KY559027/BR/2016
KU926310/BR/2016
KX197205/BR/2015
KU729218/BR/2015
KY014317/BR/2016
KY014320/BR/2016
KY014296/BR/2016
KU527068/BR/2015
KY441402/BR/2016
KY441403/BR/2016
KU365778/BR/2015
KU365779/BR/2015
KU365780/BR/2015
KU365777/BR/2015
KY014297/BR/2016
KY785450/BR/2016
MH513600/BR/2015
KU729217/BR/2015
KY120352/KR/BR/2016
MH882544/BR/2016
MH882545/BR/2016
MH882543/BR/2016
MH882542/BR/2016
MH882527/BR/2016
MH882535/BR/2016
MH882534/BR/2016
MH882540/BR/2016
MH882533/BR/2016
MH882531/BR/2016
MH882538/BR/2016
KY631492/BR/2016
KU497555/BR/2015
KY785455/BR/2016
KU940228/BR/2015
KX520666/BR/2015
KY441401/BR/2016
KX197192/BR/2015
MF352141/BR/2015
KX421193/UG/1947
KX830960/UG/1947
KX377335/UG/1947
LC002520/UG/1947
KY989511/UG/1947
KU963573/UG/1947
KU955594/UG/1947
MK105975/UG/1947
KX601169/UG/1947
DQ859059/UG/1947

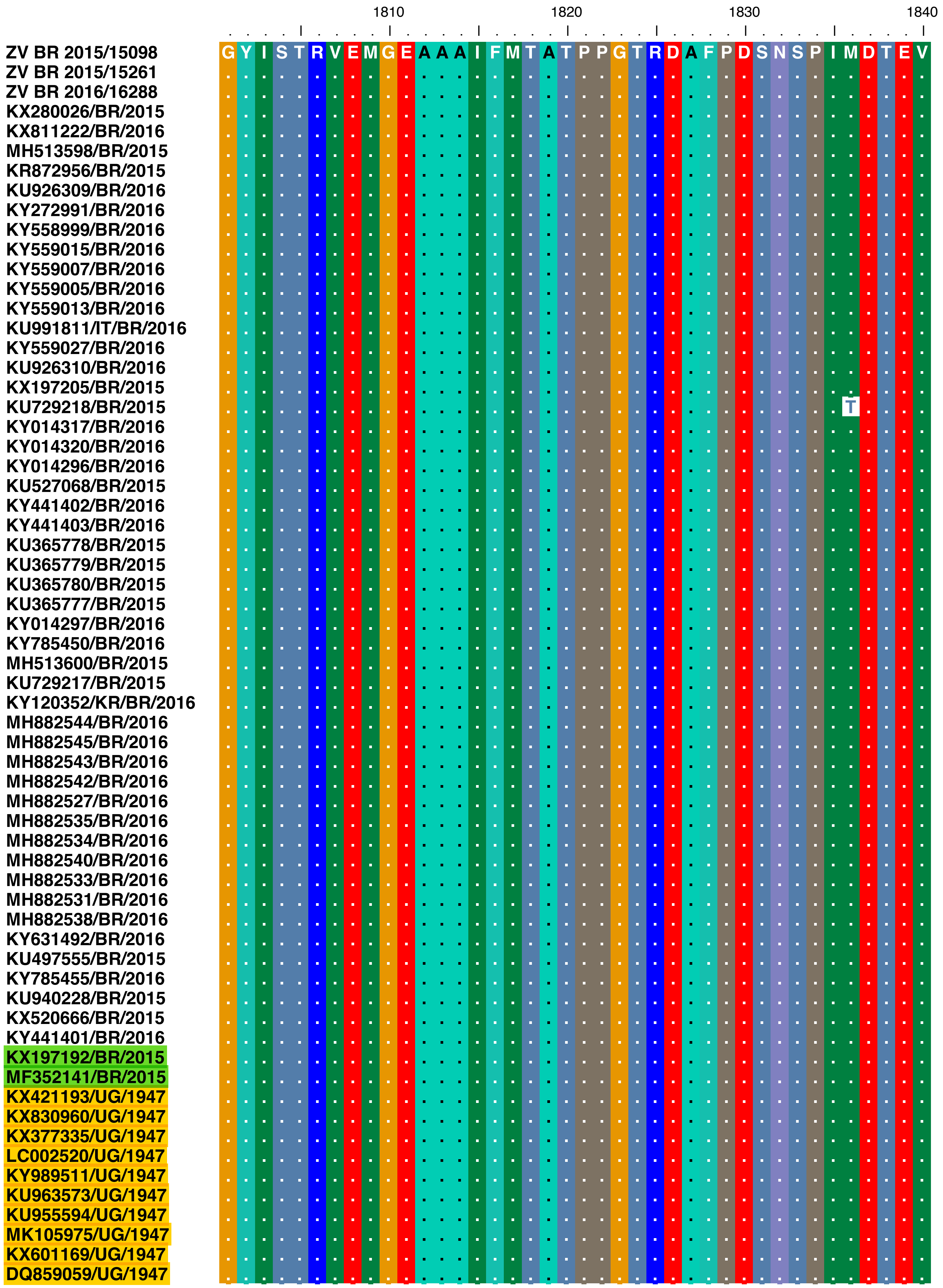
1810
1820
1830
1840
G Y I S T R V E M G E A A A I F M T A T P P G T R D A F P D S N S P I M D T E V
ZV BR 2015/15098
ZV BR 2015/15261
ZV BR 2016/16288
KX280026/BR/2015
KX811222/BR/2016
MH513598/BR/2015
KR872956/BR/2015
KU926309/BR/2016
KY272991/BR/2016
KY558999/BR/2016
KY559015/BR/2016
KY559007/BR/2016
KY559005/BR/2016
KY559013/BR/2016
KU991811/IT/BR/2016
KY559027/BR/2016
KU926310/BR/2016
KX197205/BR/2015
KU729218/BR/2015
T
KY014317/BR/2016
KY014320/BR/2016
KY014296/BR/2016
KU527068/BR/2015
KY441402/BR/2016
KY441403/BR/2016
KU365778/BR/2015
KU365779/BR/2015
KU365780/BR/2015
KU365777/BR/2015
KY014297/BR/2016
KY785450/BR/2016
MH513600/BR/2015
KU729217/BR/2015
KY120352/KR/BR/2016
MH882544/BR/2016
MH882545/BR/2016
MH882543/BR/2016
MH882542/BR/2016
MH882527/BR/2016
MH882535/BR/2016
MH882534/BR/2016
MH882540/BR/2016
MH882533/BR/2016
MH882531/BR/2016
MH882538/BR/2016
KY631492/BR/2016
KU497555/BR/2015
KY785455/BR/2016
KU940228/BR/2015
KX520666/BR/2015
KY441401/BR/2016
KX197192/BR/2015
MF352141/BR/2015
KX421193/UG/1947
KX830960/UG/1947
KX377335/UG/1947
LC002520/UG/1947
KY989511/UG/1947
KU963573/UG/1947
KU955594/UG/1947
MK105975/UG/1947
KX601169/UG/1947
DQ859059/UG/1947

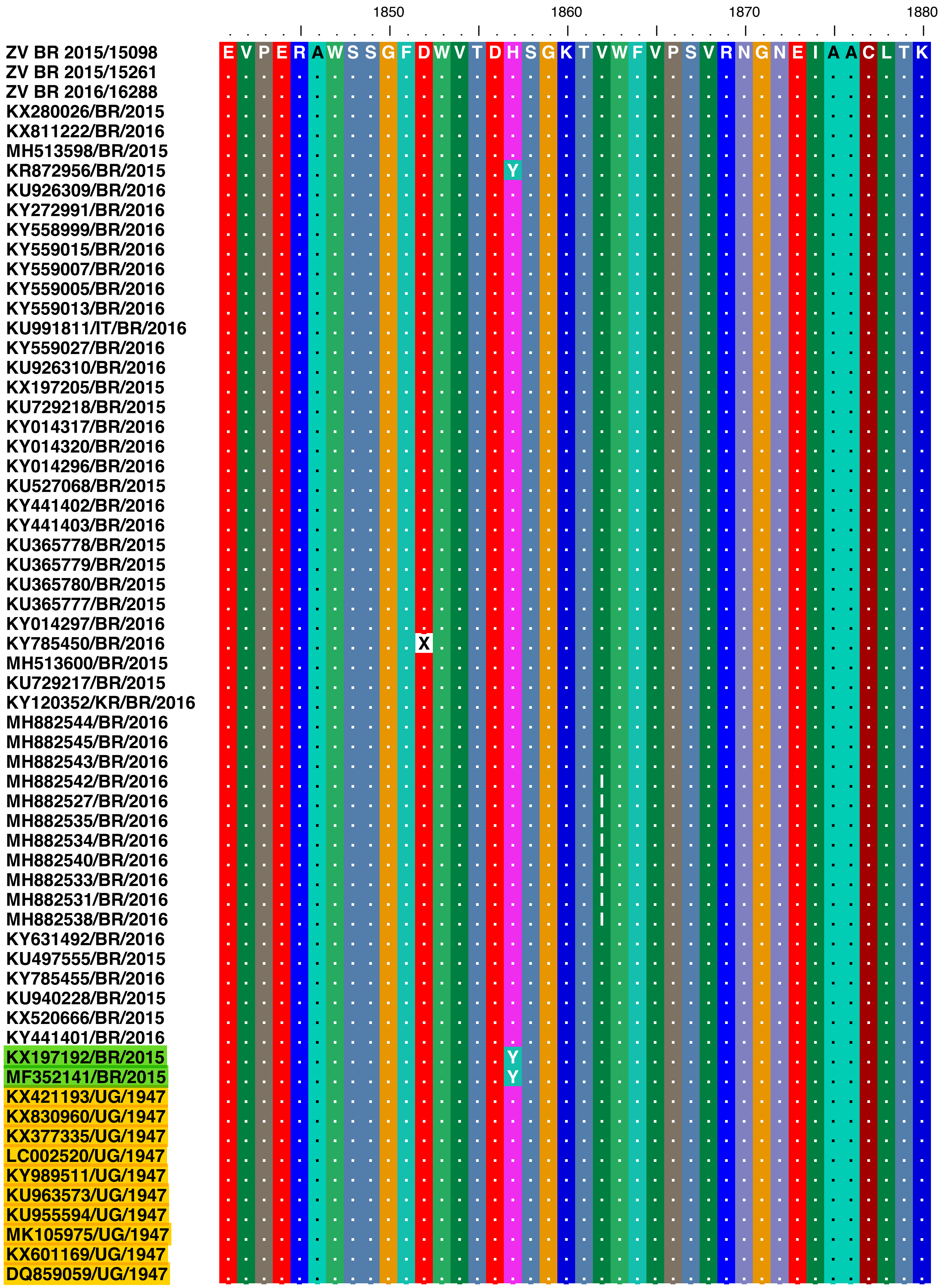

1850
1860
1870
1880
E V P E R A W S S G F D W V T D H S G K T V W F V P S V R N G N E I A A C L T K
ZV BR 2015/15098
ZV BR 2015/15261
ZV BR 2016/16288
KX280026/BR/2015
KX811222/BR/2016
MH513598/BR/2015
KR872956/BR/2015
Y
KU926309/BR/2016
KY272991/BR/2016
KY558999/BR/2016
KY559015/BR/2016
KY559007/BR/2016
KY559005/BR/2016
KY559013/BR/2016
KU991811/IT/BR/2016
KY559027/BR/2016
KU926310/BR/2016
KX197205/BR/2015
KU729218/BR/2015
KY014317/BR/2016
KY014320/BR/2016
KY014296/BR/2016
KU527068/BR/2015
KY441402/BR/2016
KY441403/BR/2016
KU365778/BR/2015
KU365779/BR/2015
KU365780/BR/2015
KU365777/BR/2015
KY014297/BR/2016
KY785450/BR/2016
X
MH513600/BR/2015
KU729217/BR/2015
KY120352/KR/BR/2016
MH882544/BR/2016
MH882545/BR/2016
MH882543/BR/2016
MH882542/BR/2016
I
MH882527/BR/2016
I
MH882535/BR/2016
I
MH882534/BR/2016
I
MH882540/BR/2016
I
MH882533/BR/2016
I
MH882531/BR/2016
I
MH882538/BR/2016
I
KY631492/BR/2016
KU497555/BR/2015
KY785455/BR/2016
KU940228/BR/2015
KX520666/BR/2015
KY441401/BR/2016
KX197192/BR/2015
Y
MF352141/BR/2015
Y
KX421193/UG/1947
KX830960/UG/1947
KX377335/UG/1947
LC002520/UG/1947
KY989511/UG/1947
KU963573/UG/1947
KU955594/UG/1947
MK105975/UG/1947
KX601169/UG/1947
DQ859059/UG/1947

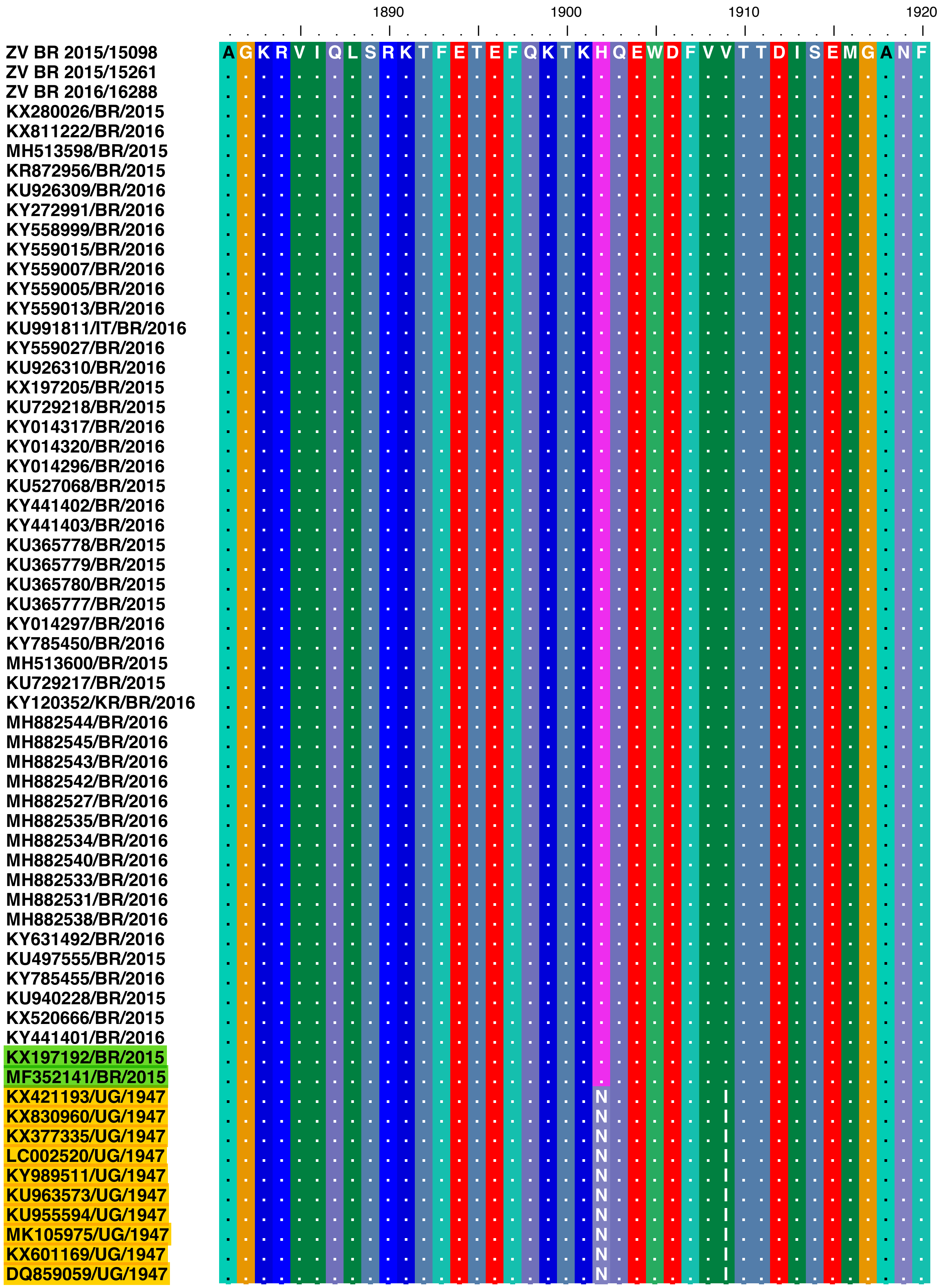
1890
1900
1910
1920
A G K R V I Q L S R K T F E T E F Q K T K H Q E W D F V V T T D I S E M G A N F
ZV BR 2015/15098
ZV BR 2015/15261
ZV BR 2016/16288
KX280026/BR/2015
KX811222/BR/2016
MH513598/BR/2015
KR872956/BR/2015
KU926309/BR/2016
KY272991/BR/2016
KY558999/BR/2016
KY559015/BR/2016
KY559007/BR/2016
KY559005/BR/2016
KY559013/BR/2016
KU991811/IT/BR/2016
KY559027/BR/2016
KU926310/BR/2016
KX197205/BR/2015
KU729218/BR/2015
KY014317/BR/2016
KY014320/BR/2016
KY014296/BR/2016
KU527068/BR/2015
KY441402/BR/2016
KY441403/BR/2016
KU365778/BR/2015
KU365779/BR/2015
KU365780/BR/2015
KU365777/BR/2015
KY014297/BR/2016
KY785450/BR/2016
MH513600/BR/2015
KU729217/BR/2015
KY120352/KR/BR/2016
MH882544/BR/2016
MH882545/BR/2016
MH882543/BR/2016
MH882542/BR/2016
MH882527/BR/2016
MH882535/BR/2016
MH882534/BR/2016
MH882540/BR/2016
MH882533/BR/2016
MH882531/BR/2016
MH882538/BR/2016
KY631492/BR/2016
KU497555/BR/2015
KY785455/BR/2016
KU940228/BR/2015
KX520666/BR/2015
KY441401/BR/2016
KX197192/BR/2015
MF352141/BR/2015
KX421193/UG/1947
KX830960/UG/1947
KX377335/UG/1947
LC002520/UG/1947
KY989511/UG/1947
KU963573/UG/1947
KU955594/UG/1947
MK105975/UG/1947
KX601169/UG/1947
DQ859059/UG/1947
N
I

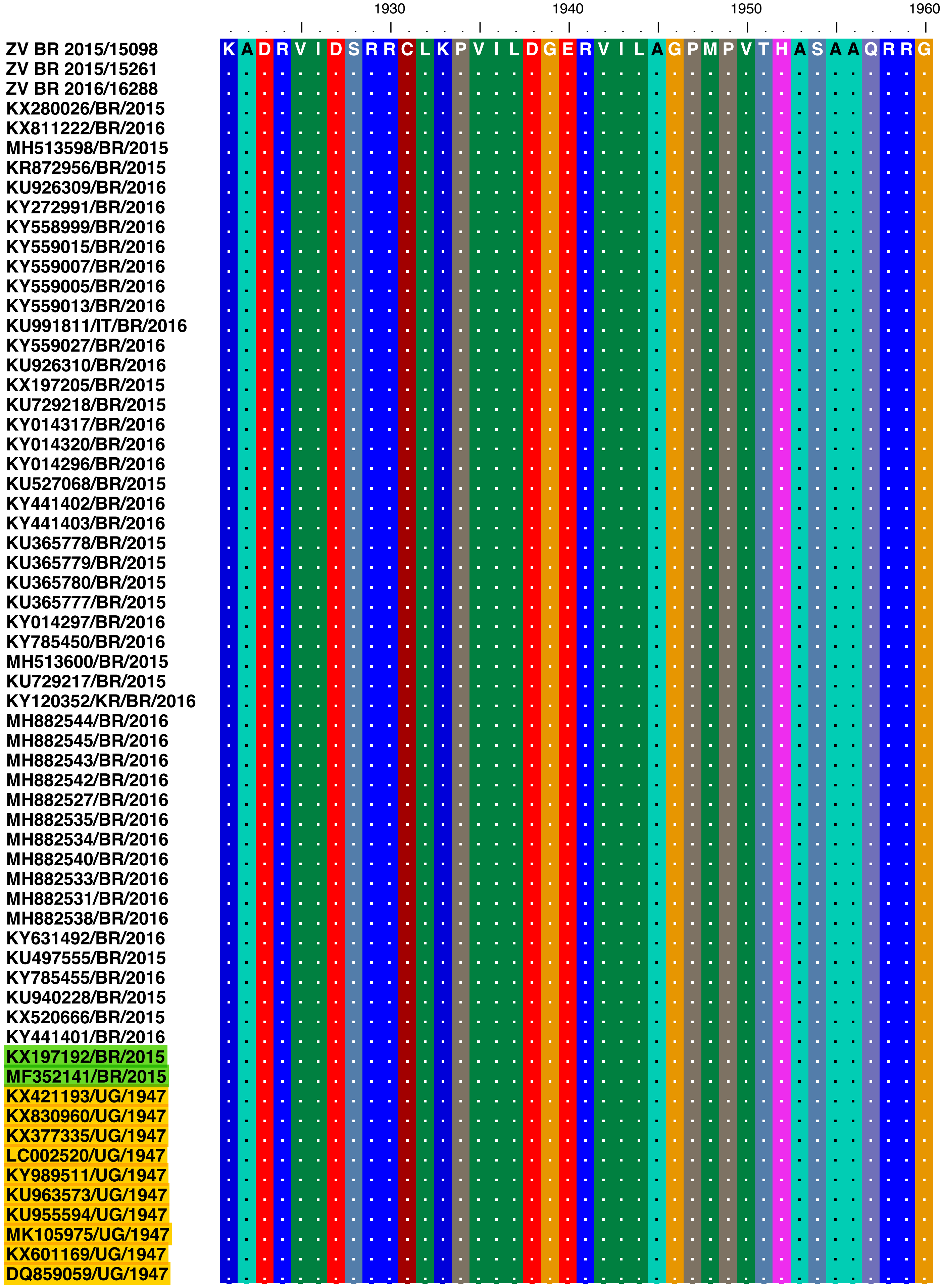

1930
1940
1950
1960
ZV BR 2015/15098
ZV BR 2015/15261
ZV BR 2016/16288
KX280026/BR/2015
KX811222/BR/2016
MH513598/BR/2015
KR872956/BR/2015
KU926309/BR/2016
KY272991/BR/2016
KY558999/BR/2016
KY559015/BR/2016
KY559007/BR/2016
KY559005/BR/2016
KY559013/BR/2016
KU991811/IT/BR/2016
KY559027/BR/2016
KU926310/BR/2016
KX197205/BR/2015
KU729218/BR/2015
KY014317/BR/2016
KY014320/BR/2016
KY014296/BR/2016
KU527068/BR/2015
KY441402/BR/2016
KY441403/BR/2016
KU365778/BR/2015
KU365779/BR/2015
KU365780/BR/2015
KU365777/BR/2015
KY014297/BR/2016
KY785450/BR/2016
MH513600/BR/2015
KU729217/BR/2015
KY120352/KR/BR/2016
MH882544/BR/2016
MH882545/BR/2016
MH882543/BR/2016
MH882542/BR/2016
MH882527/BR/2016
MH882535/BR/2016
MH882534/BR/2016
MH882540/BR/2016
MH882533/BR/2016
MH882531/BR/2016
MH882538/BR/2016
KY631492/BR/2016
KU497555/BR/2015
KY785455/BR/2016
KU940228/BR/2015
KX520666/BR/2015
KY441401/BR/2016
KX197192/BR/2015
MF352141/BR/2015
KX421193/UG/1947
KX830960/UG/1947
KX377335/UG/1947
LC002520/UG/1947
KY989511/UG/1947
KU963573/UG/1947
KU955594/UG/1947
MK105975/UG/1947
KX601169/UG/1947
DQ859059/UG/1947
K A D R V I D S R R C L K P V I L D G E R V I L A G P M P V T H A S A A Q R R G

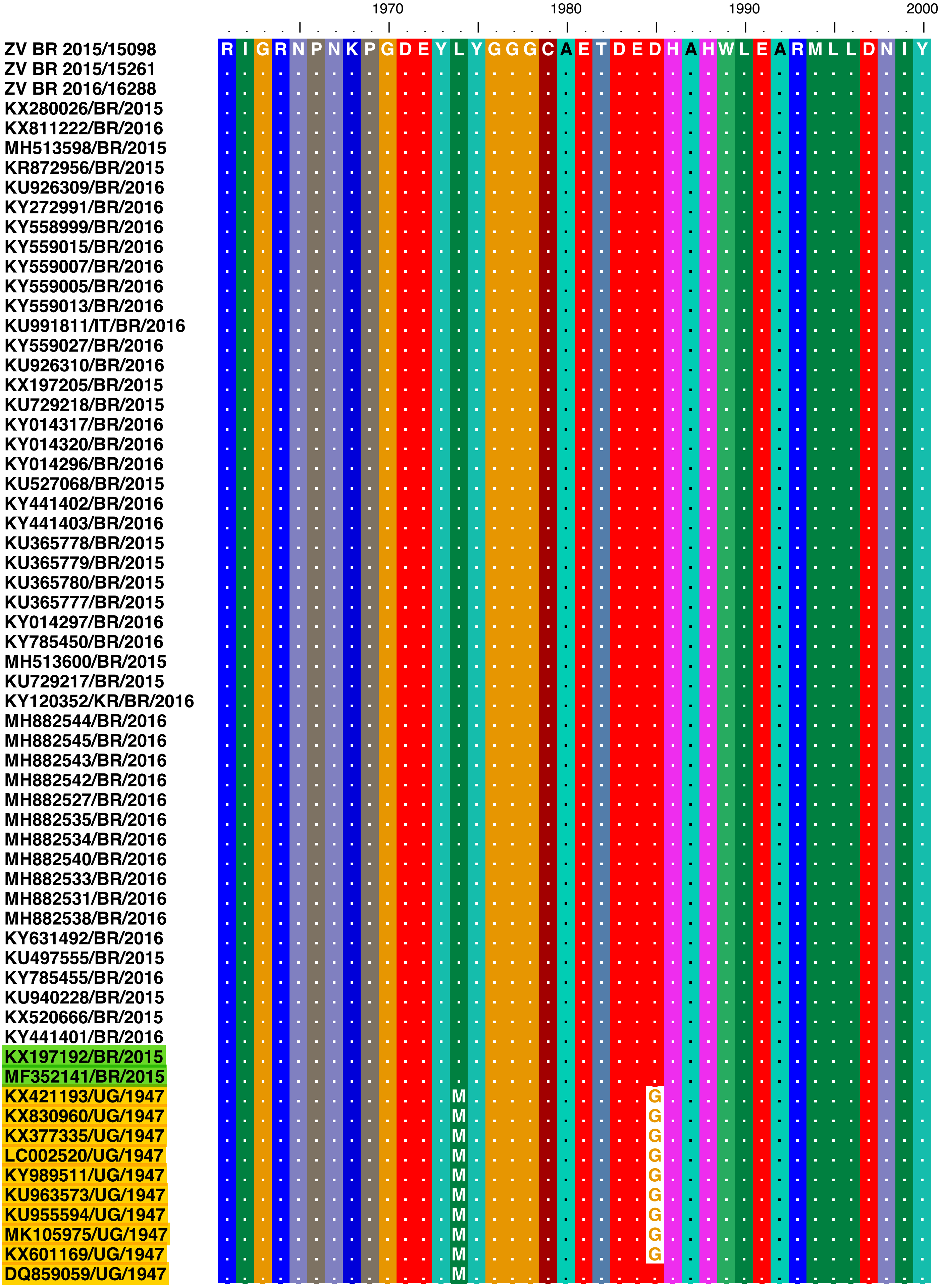
1970
1980
1990
2000
R I G R N P N K P G D E Y L Y G G G C A E T D E D H A H W L E A R M L L D N I Y
ZV BR 2015/15098
ZV BR 2015/15261
ZV BR 2016/16288
KX280026/BR/2015
KX811222/BR/2016
MH513598/BR/2015
KR872956/BR/2015
KU926309/BR/2016
KY272991/BR/2016
KY558999/BR/2016
KY559015/BR/2016
KY559007/BR/2016
KY559005/BR/2016
KY559013/BR/2016
KU991811/IT/BR/2016
KY559027/BR/2016
KU926310/BR/2016
KX197205/BR/2015
KU729218/BR/2015
KY014317/BR/2016
KY014320/BR/2016
KY014296/BR/2016
KU527068/BR/2015
KY441402/BR/2016
KY441403/BR/2016
KU365778/BR/2015
KU365779/BR/2015
KU365780/BR/2015
KU365777/BR/2015
KY014297/BR/2016
KY785450/BR/2016
MH513600/BR/2015
KU729217/BR/2015
KY120352/KR/BR/2016
MH882544/BR/2016
MH882545/BR/2016
MH882543/BR/2016
MH882542/BR/2016
MH882527/BR/2016
MH882535/BR/2016
MH882534/BR/2016
MH882540/BR/2016
MH882533/BR/2016
MH882531/BR/2016
MH882538/BR/2016
KY631492/BR/2016
KU497555/BR/2015
KY785455/BR/2016
KU940228/BR/2015
KX520666/BR/2015
KY441401/BR/2016
KX197192/BR/2015
MF352141/BR/2015
KX421193/UG/1947 M G
KX830960/UG/1947 M G
KX377335/UG/1947 M G
LC002520/UG/1947 M G
KY989511/UG/1947 M G
KU963573/UG/1947 M G
KU955594/UG/1947 M G
MK105975/UG/1947 M G
KX601169/UG/1947 M G
DQ859059/UG/1947 M

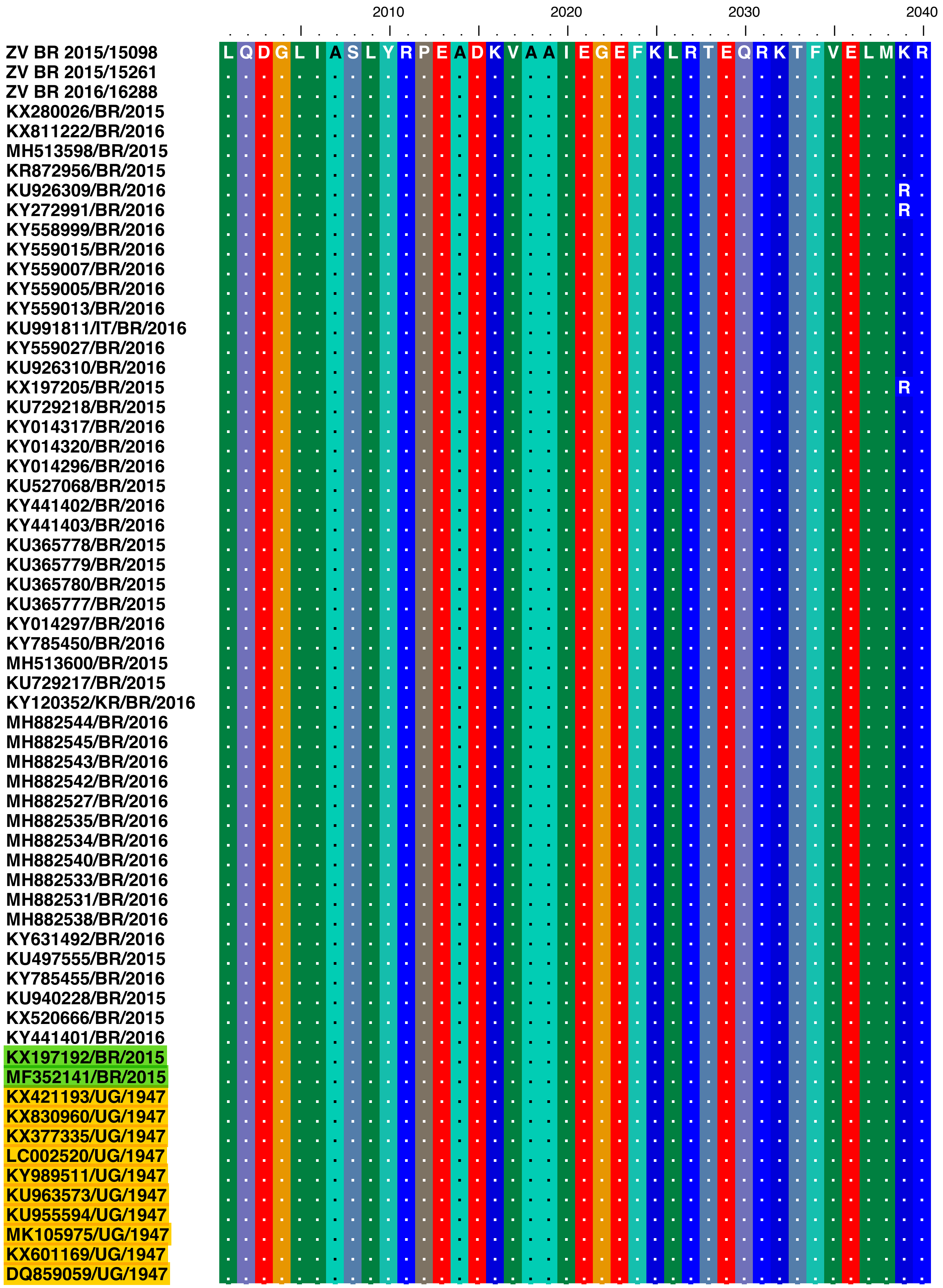
2010
2020
2030
2040
L Q D G L I A S L Y R P E A D K V A A I E G E F K L R T E Q R K T F V E L M K R
ZV BR 2015/15098
ZV BR 2015/15261
ZV BR 2016/16288
KX280026/BR/2015
KX811222/BR/2016
MH513598/BR/2015
KR872956/BR/2015
KU926309/BR/2016
R
KY272991/BR/2016
R
KY558999/BR/2016
KY559015/BR/2016
KY559007/BR/2016
KY559005/BR/2016
KY559013/BR/2016
KU991811/IT/BR/2016
KY559027/BR/2016
KU926310/BR/2016
KX197205/BR/2015
R
KU729218/BR/2015
KY014317/BR/2016
KY014320/BR/2016
KY014296/BR/2016
KU527068/BR/2015
KY441402/BR/2016
KY441403/BR/2016
KU365778/BR/2015
KU365779/BR/2015
KU365780/BR/2015
KU365777/BR/2015
KY014297/BR/2016
KY785450/BR/2016
MH513600/BR/2015
KU729217/BR/2015
KY120352/KR/BR/2016
MH882544/BR/2016
MH882545/BR/2016
MH882543/BR/2016
MH882542/BR/2016
MH882527/BR/2016
MH882535/BR/2016
MH882534/BR/2016
MH882540/BR/2016
MH882533/BR/2016
MH882531/BR/2016
MH882538/BR/2016
KY631492/BR/2016
KU497555/BR/2015
KY785455/BR/2016
KU940228/BR/2015
KX520666/BR/2015
KY441401/BR/2016
KX197192/BR/2015
MF352141/BR/2015
KX421193/UG/1947
KX830960/UG/1947
KX377335/UG/1947
LC002520/UG/1947
KY989511/UG/1947
KU963573/UG/1947
KU955594/UG/1947
MK105975/UG/1947
KX601169/UG/1947
DQ859059/UG/1947

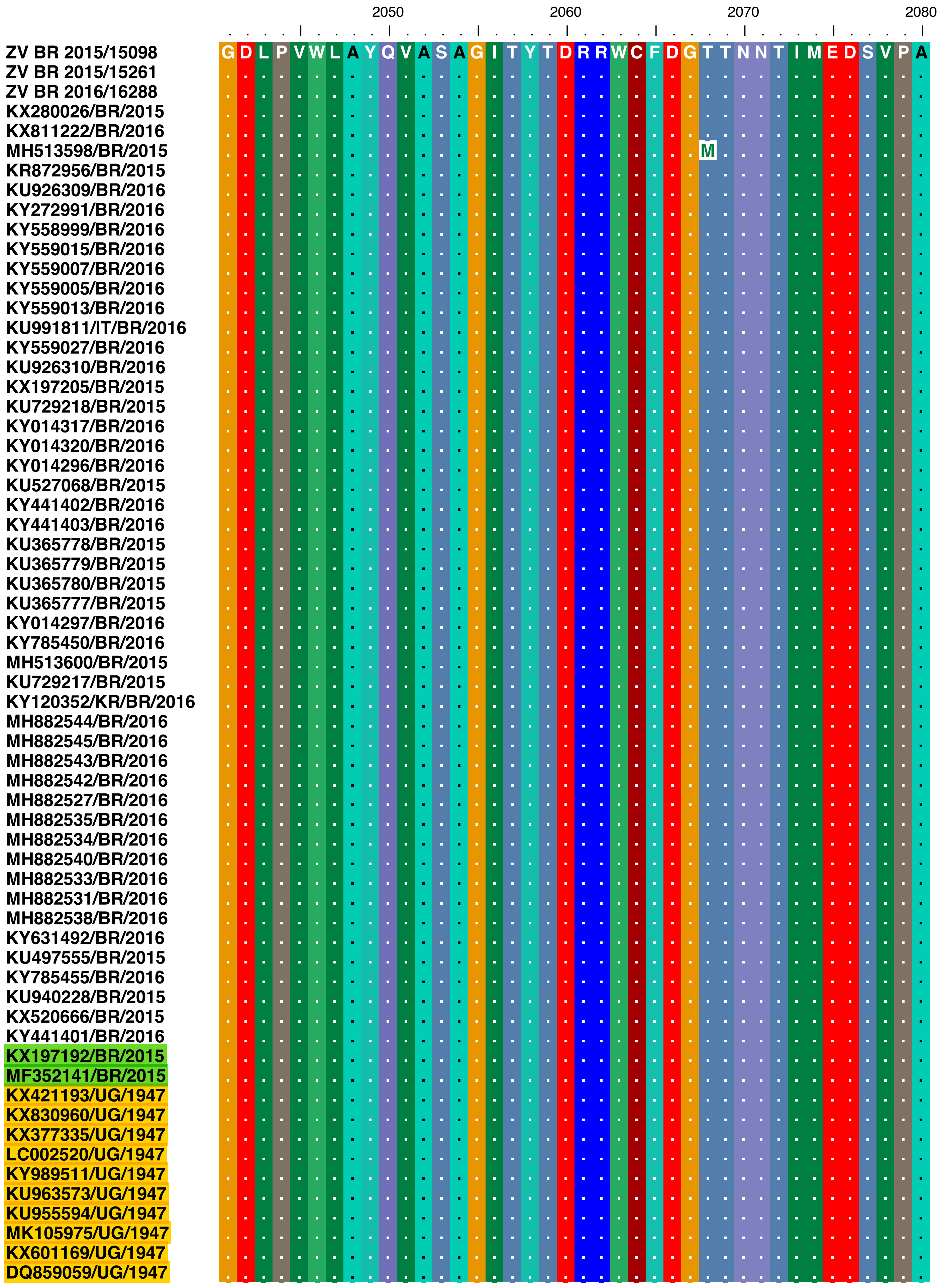

2050
2060
2070
2080
GDLPVWLAYQVASAGITYTDRRWCFDGTTNNTIMEDSVPA
M
ZV BR 2015/15098
ZV BR 2015/15261
ZV BR 2016/16288
KX280026/BR/2015
KX811222/BR/2016
MH513598/BR/2015
KR872956/BR/2015
KU926309/BR/2016
KY272991/BR/2016
KY558999/BR/2016
KY559015/BR/2016
KY559007/BR/2016
KY559005/BR/2016
KY559013/BR/2016
KU991811/IT/BR/2016
KY559027/BR/2016
KU926310/BR/2016
KX197205/BR/2015
KU729218/BR/2015
KY014317/BR/2016
KY014320/BR/2016
KY014296/BR/2016
KU527068/BR/2015
KY441402/BR/2016
KY441403/BR/2016
KU365778/BR/2015
KU365779/BR/2015
KU365780/BR/2015
KU365777/BR/2015
KY014297/BR/2016
KY785450/BR/2016
MH513600/BR/2015
KU729217/BR/2015
KY120352/KR/BR/2016
MH882544/BR/2016
MH882545/BR/2016
MH882543/BR/2016
MH882542/BR/2016
MH882527/BR/2016
MH882535/BR/2016
MH882534/BR/2016
MH882540/BR/2016
MH882533/BR/2016
MH882531/BR/2016
MH882538/BR/2016
KY631492/BR/2016
KU497555/BR/2015
KY785455/BR/2016
KU940228/BR/2015
KX520666/BR/2015
KY441401/BR/2016
KX197192/BR/2015
MF352141/BR/2015
KX421193/UG/1947
KX830960/UG/1947
KX377335/UG/1947
LC002520/UG/1947
KY989511/UG/1947
KU963573/UG/1947
KU955594/UG/1947
MK105975/UG/1947
KX601169/UG/1947
DQ859059/UG/1947

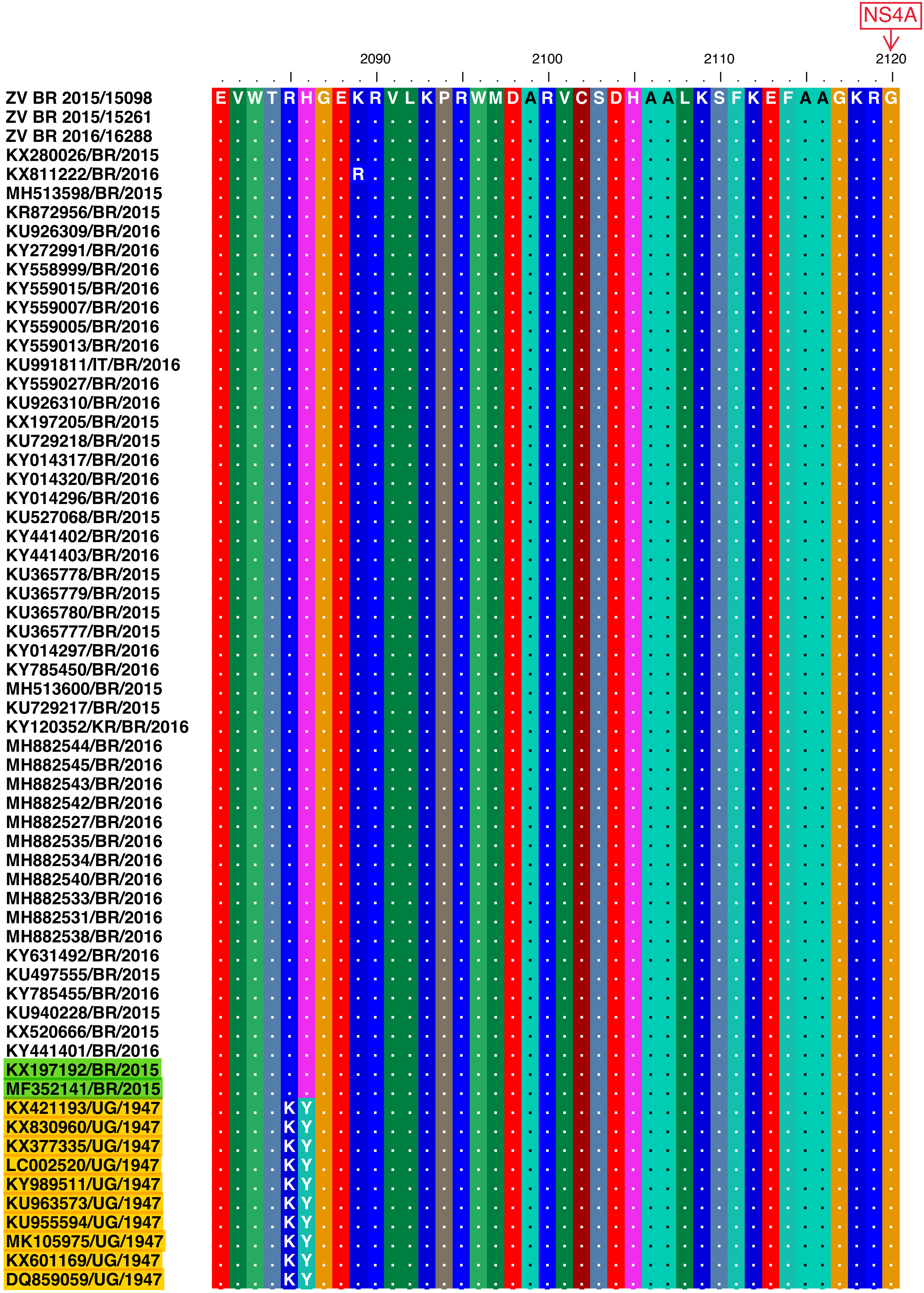
NS4A
2090
2100
2110
2120
EVWTRHGEKRVLKPRWMDARVCSDHAALKSFKEFAAGKRG
ZV BR 2015/15098
ZV BR 2015/15261
ZV BR 2016/16288
KX280026/BR/2015
KX811222/BR/2016
MH513598/BR/2015
KR872956/BR/2015
KU926309/BR/2016
KY272991/BR/2016
KY558999/BR/2016
KY559015/BR/2016
KY559007/BR/2016
KY559005/BR/2016
KY559013/BR/2016
KU991811/IT/BR/2016
KY559027/BR/2016
KU926310/BR/2016
KX197205/BR/2015
KU729218/BR/2015
KY014317/BR/2016
KY014320/BR/2016
KY014296/BR/2016
KU527068/BR/2015
KY441402/BR/2016
KY441403/BR/2016
KU365778/BR/2015
KU365779/BR/2015
KU365780/BR/2015
KU365777/BR/2015
KY014297/BR/2016
KY785450/BR/2016
MH513600/BR/2015
KU729217/BR/2015
KY120352/KR/BR/2016
MH882544/BR/2016
MH882545/BR/2016
MH882543/BR/2016
MH882542/BR/2016
MH882527/BR/2016
MH882535/BR/2016
MH882534/BR/2016
MH882540/BR/2016
MH882533/BR/2016
MH882531/BR/2016
MH882538/BR/2016
KY631492/BR/2016
KU497555/BR/2015
KY785455/BR/2016
KU940228/BR/2015
KX520666/BR/2015
KY441401/BR/2016
KX197192/BR/2015
MF352141/BR/2015
KX421193/UG/1947
KX830960/UG/1947
KX377335/UG/1947
LC002520/UG/1947
KY989511/UG/1947
KU963573/UG/1947
KU955594/UG/1947
MK105975/UG/1947
KX601169/UG/1947
DQ859059/UG/1947

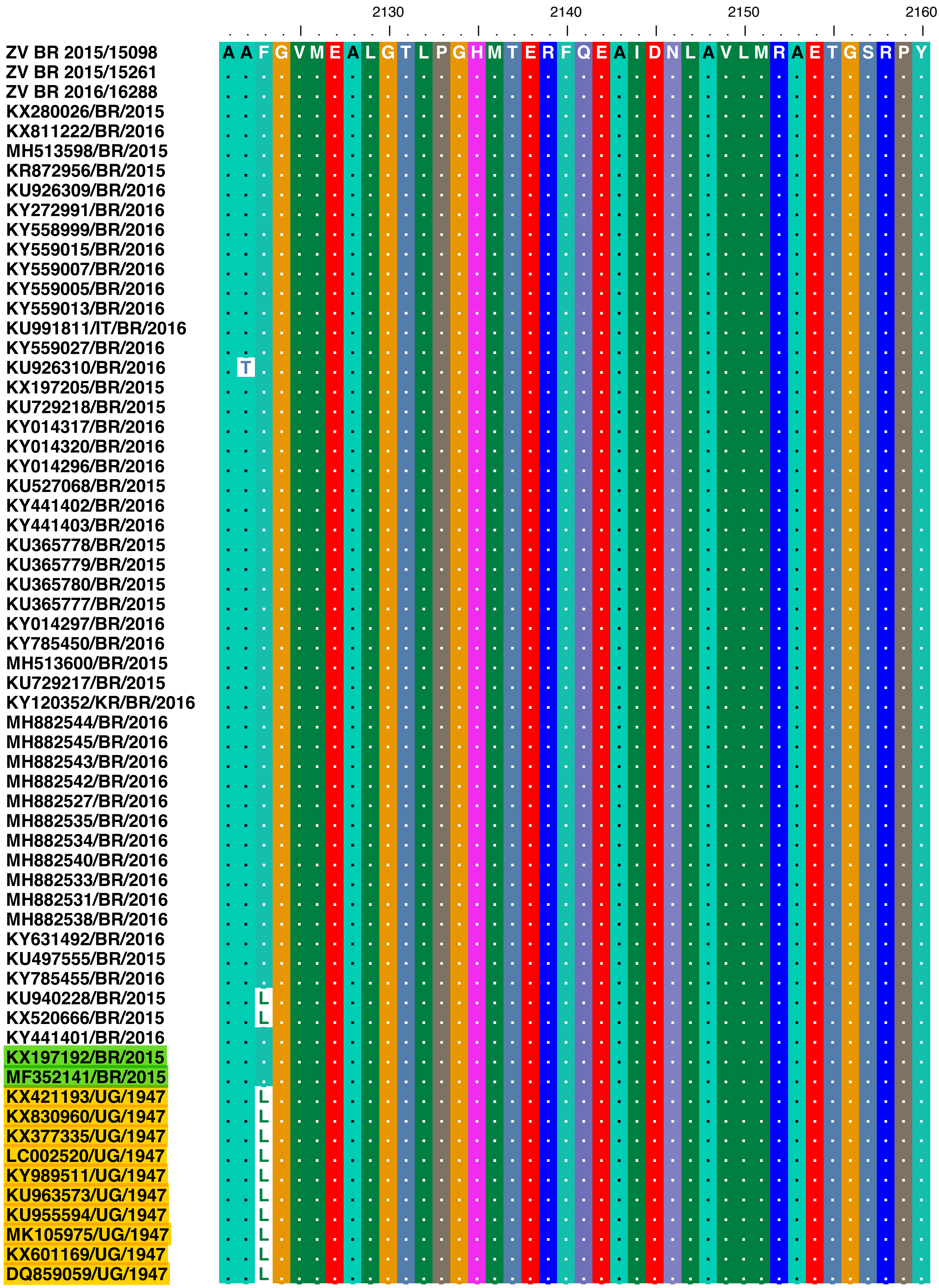
2130
2140
2150
2160
A A F G V M E A L G T L P G H M T E R F Q E A I D N L A V L M R A E T G S R P Y
ZV BR 2015/15098
ZV BR 2015/15261
ZV BR 2016/16288
KX280026/BR/2015
KX811222/BR/2016
MH513598/BR/2015
KR872956/BR/2015
KU926309/BR/2016
KY272991/BR/2016
KY558999/BR/2016
KY559015/BR/2016
KY559007/BR/2016
KY559005/BR/2016
KY559013/BR/2016
KU991811/IT/BR/2016
KY559027/BR/2016
KU926310/BR/2016
T
KX197205/BR/2015
KU729218/BR/2015
KY014317/BR/2016
KY014320/BR/2016
KY014296/BR/2016
KU527068/BR/2015
KY441402/BR/2016
KY441403/BR/2016
KU365778/BR/2015
KU365779/BR/2015
KU365780/BR/2015
KU365777/BR/2015
KY014297/BR/2016
KY785450/BR/2016
MH513600/BR/2015
KU729217/BR/2015
KY120352/KR/BR/2016
MH882544/BR/2016
MH882545/BR/2016
MH882543/BR/2016
MH882542/BR/2016
MH882527/BR/2016
MH882535/BR/2016
MH882534/BR/2016
MH882540/BR/2016
MH882533/BR/2016
MH882531/BR/2016
MH882538/BR/2016
KY631492/BR/2016
KU497555/BR/2015
KY785455/BR/2016
KU940228/BR/2015
L
KX520666/BR/2015
L
KY441401/BR/2016
KX197192/BR/2015
MF352141/BR/2015
KX421193/UG/1947
L
KX830960/UG/1947
L
KX377335/UG/1947
L
LC002520/UG/1947
L
KY989511/UG/1947
L
KU963573/UG/1947
L
KU955594/UG/1947
L
MK105975/UG/1947
L
KX601169/UG/1947
L
DQ859059/UG/1947
L

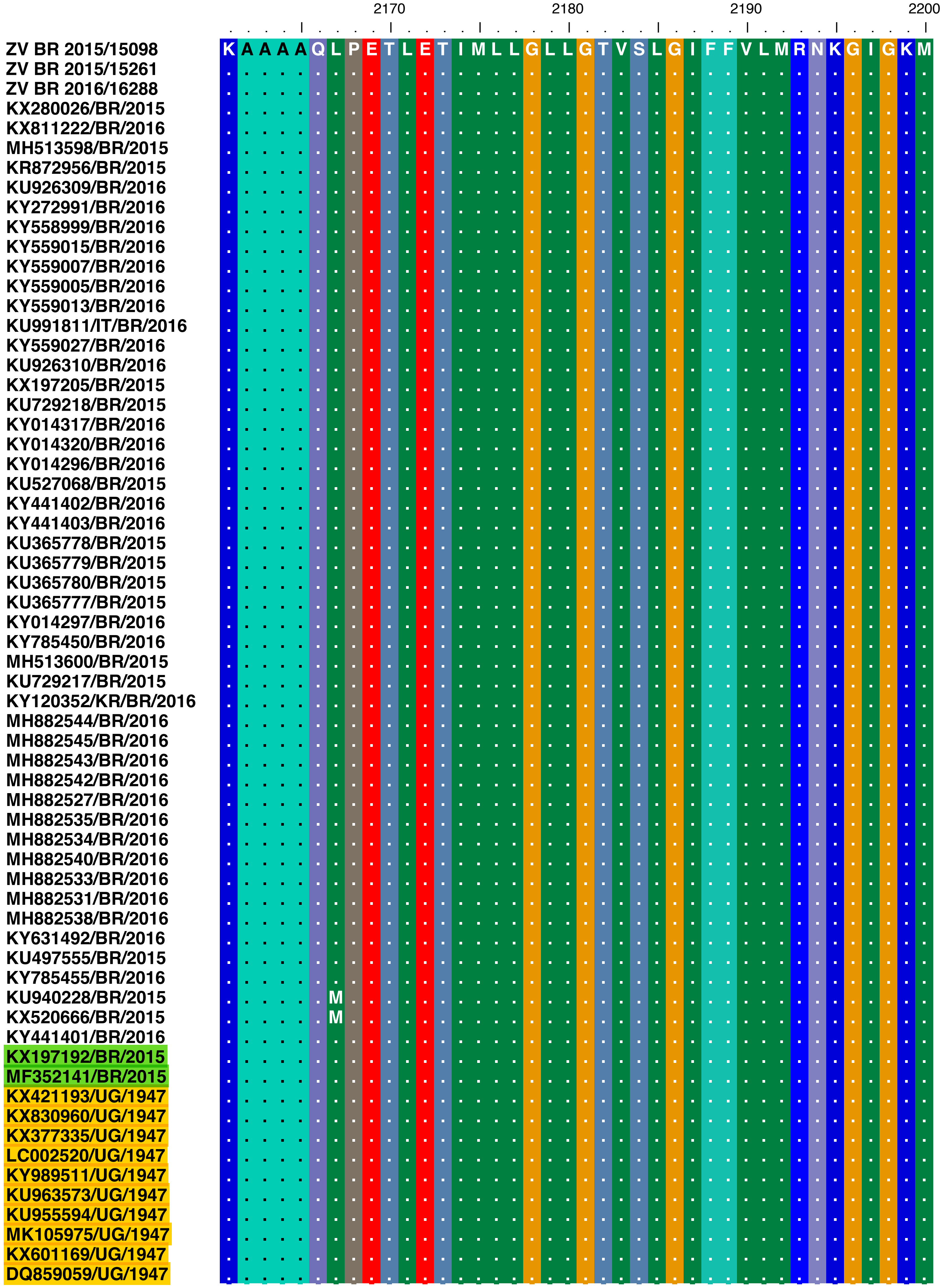
2170
2180
2190
2200
K A A A A Q L P E T L E T I M L L G L L G T V S L G I F F V L M R N K G I G K M
ZV BR 2015/15098
ZV BR 2015/15261
ZV BR 2016/16288
KX280026/BR/2015
KX811222/BR/2016
MH513598/BR/2015
KR872956/BR/2015
KU926309/BR/2016
KY272991/BR/2016
KY558999/BR/2016
KY559015/BR/2016
KY559007/BR/2016
KY559005/BR/2016
KY559013/BR/2016
KU991811/IT/BR/2016
KY559027/BR/2016
KU926310/BR/2016
KX197205/BR/2015
KU729218/BR/2015
KY014317/BR/2016
KY014320/BR/2016
KY014296/BR/2016
KU527068/BR/2015
KY441402/BR/2016
KY441403/BR/2016
KU365778/BR/2015
KU365779/BR/2015
KU365780/BR/2015
KU365777/BR/2015
KY014297/BR/2016
KY785450/BR/2016
MH513600/BR/2015
KU729217/BR/2015
KY120352/KR/BR/2016
MH882544/BR/2016
MH882545/BR/2016
MH882543/BR/2016
MH882542/BR/2016
MH882527/BR/2016
MH882535/BR/2016
MH882534/BR/2016
MH882540/BR/2016
MH882533/BR/2016
MH882531/BR/2016
MH882538/BR/2016
KY631492/BR/2016
KU497555/BR/2015
KY785455/BR/2016
KU940228/BR/2015 M
KX520666/BR/2015 M
KY441401/BR/2016
KX197192/BR/2015
MF352141/BR/2015
KX421193/UG/1947
KX830960/UG/1947
KX377335/UG/1947
LC002520/UG/1947
KY989511/UG/1947
KU963573/UG/1947
KU955594/UG/1947
MK105975/UG/1947
KX601169/UG/1947
DQ859059/UG/1947

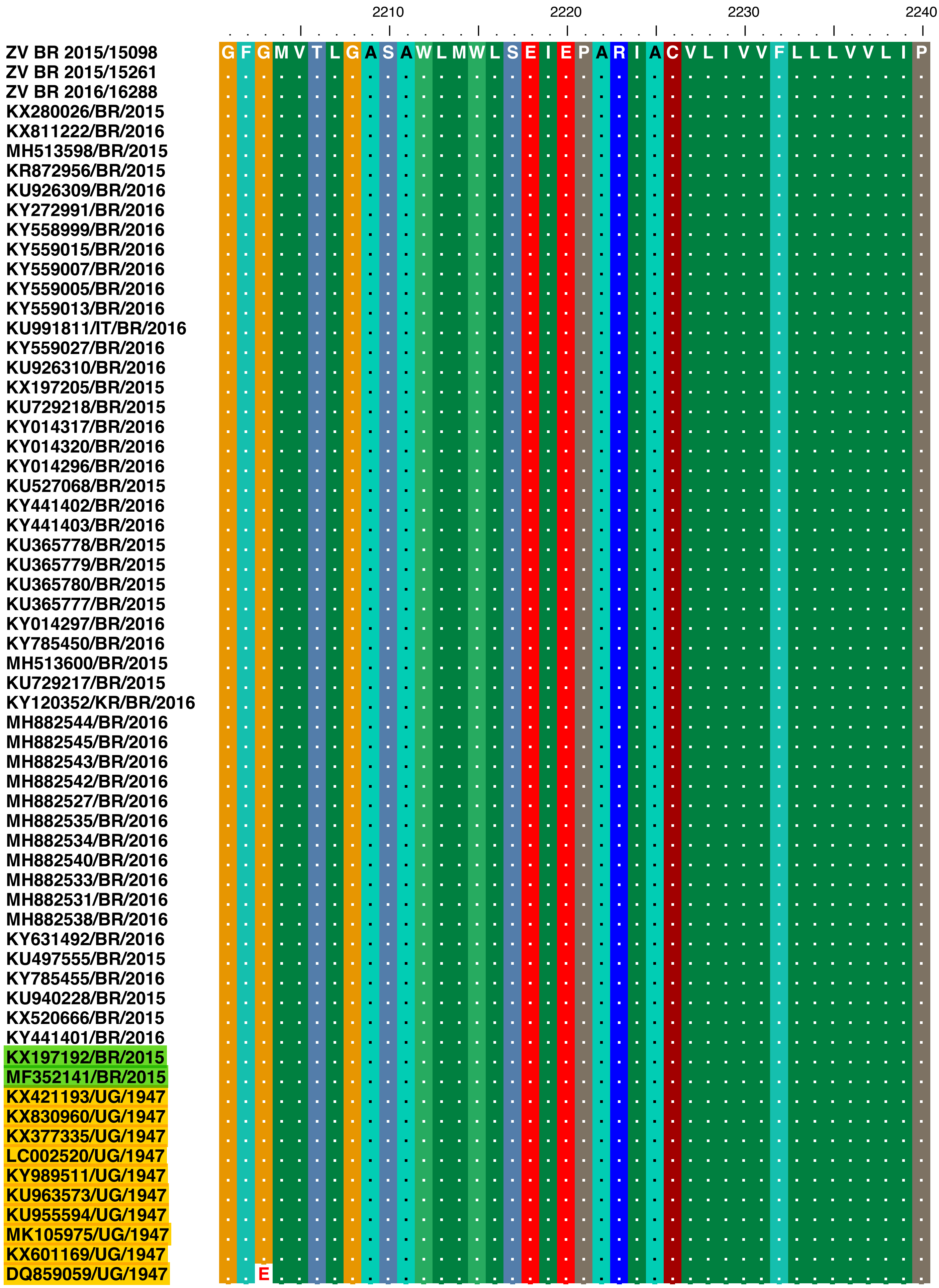

2210
2220
2230
2240
ZV BR 2015/15098
ZV BR 2015/15261
ZV BR 2016/16288
KX280026/BR/2015
KX811222/BR/2016
MH513598/BR/2015
KR872956/BR/2015
KU926309/BR/2016
KY272991/BR/2016
KY558999/BR/2016
KY559015/BR/2016
KY559007/BR/2016
KY559005/BR/2016
KY559013/BR/2016
KU991811/IT/BR/2016
KY559027/BR/2016
KU926310/BR/2016
KX197205/BR/2015
KU729218/BR/2015
KY014317/BR/2016
KY014320/BR/2016
KY014296/BR/2016
KU527068/BR/2015
KY441402/BR/2016
KY441403/BR/2016
KU365778/BR/2015
KU365779/BR/2015
KU365780/BR/2015
KU365777/BR/2015
KY014297/BR/2016
KY785450/BR/2016
MH513600/BR/2015
KU729217/BR/2015
KY120352/KR/BR/2016
MH882544/BR/2016
MH882545/BR/2016
MH882543/BR/2016
MH882542/BR/2016
MH882527/BR/2016
MH882535/BR/2016
MH882534/BR/2016
MH882540/BR/2016
MH882533/BR/2016
MH882531/BR/2016
MH882538/BR/2016
KY631492/BR/2016
KU497555/BR/2015
KY785455/BR/2016
KU940228/BR/2015
KX520666/BR/2015
KY441401/BR/2016
KX197192/BR/2015
MF352141/BR/2015
KX421193/UG/1947
KX830960/UG/1947
KX377335/UG/1947
LC002520/UG/1947
KY989511/UG/1947
KU963573/UG/1947
KU955594/UG/1947
MK105975/UG/1947
KX601169/UG/1947
DQ859059/UG/1947
G F G M V T L G A S A W L M W L S E I E P A R I A C V L I V V F L L L V V L I P
E

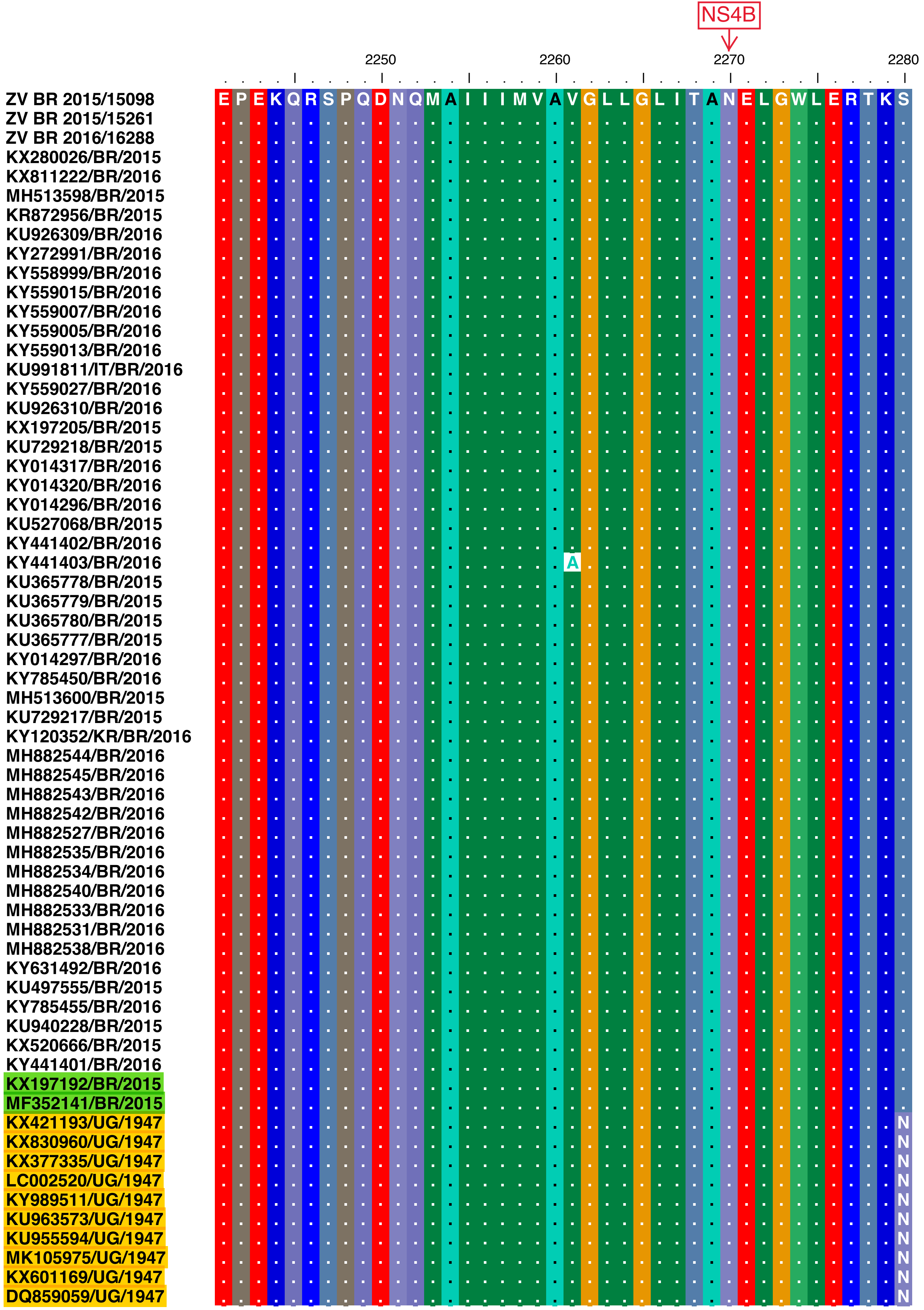
NS4B
2250
2260
2270
2280
EPEKQRSPQDNQMAIIIMVAVGLLGLITANELGWLERTKS
ZV BR 2015/15098
ZV BR 2015/15261
ZV BR 2016/16288
KX280026/BR/2015
KX811222/BR/2016
MH513598/BR/2015
KR872956/BR/2015
KU926309/BR/2016
KY272991/BR/2016
KY558999/BR/2016
KY559015/BR/2016
KY559007/BR/2016
KY559005/BR/2016
KY559013/BR/2016
KU991811/IT/BR/2016
KY559027/BR/2016
KU926310/BR/2016
KX197205/BR/2015
KU729218/BR/2015
KY014317/BR/2016
KY014320/BR/2016
KY014296/BR/2016
KU527068/BR/2015
KY441402/BR/2016
KY441403/BR/2016
A
KU365778/BR/2015
KU365779/BR/2015
KU365780/BR/2015
KU365777/BR/2015
KY014297/BR/2016
KY785450/BR/2016
MH513600/BR/2015
KU729217/BR/2015
KY120352/KR/BR/2016
MH882544/BR/2016
MH882545/BR/2016
MH882543/BR/2016
MH882542/BR/2016
MH882527/BR/2016
MH882535/BR/2016
MH882534/BR/2016
MH882540/BR/2016
MH882533/BR/2016
MH882531/BR/2016
MH882538/BR/2016
KY631492/BR/2016
KU497555/BR/2015
KY785455/BR/2016
KU940228/BR/2015
KX520666/BR/2015
KY441401/BR/2016
KX197192/BR/2015
MF352141/BR/2015
KX421193/UG/1947
N
KX830960/UG/1947
N
KX377335/UG/1947
N
LC002520/UG/1947
N
KY989511/UG/1947
N
KU963573/UG/1947
N
KU955594/UG/1947
N
MK105975/UG/1947
N
KX601169/UG/1947
N
DQ859059/UG/1947
N

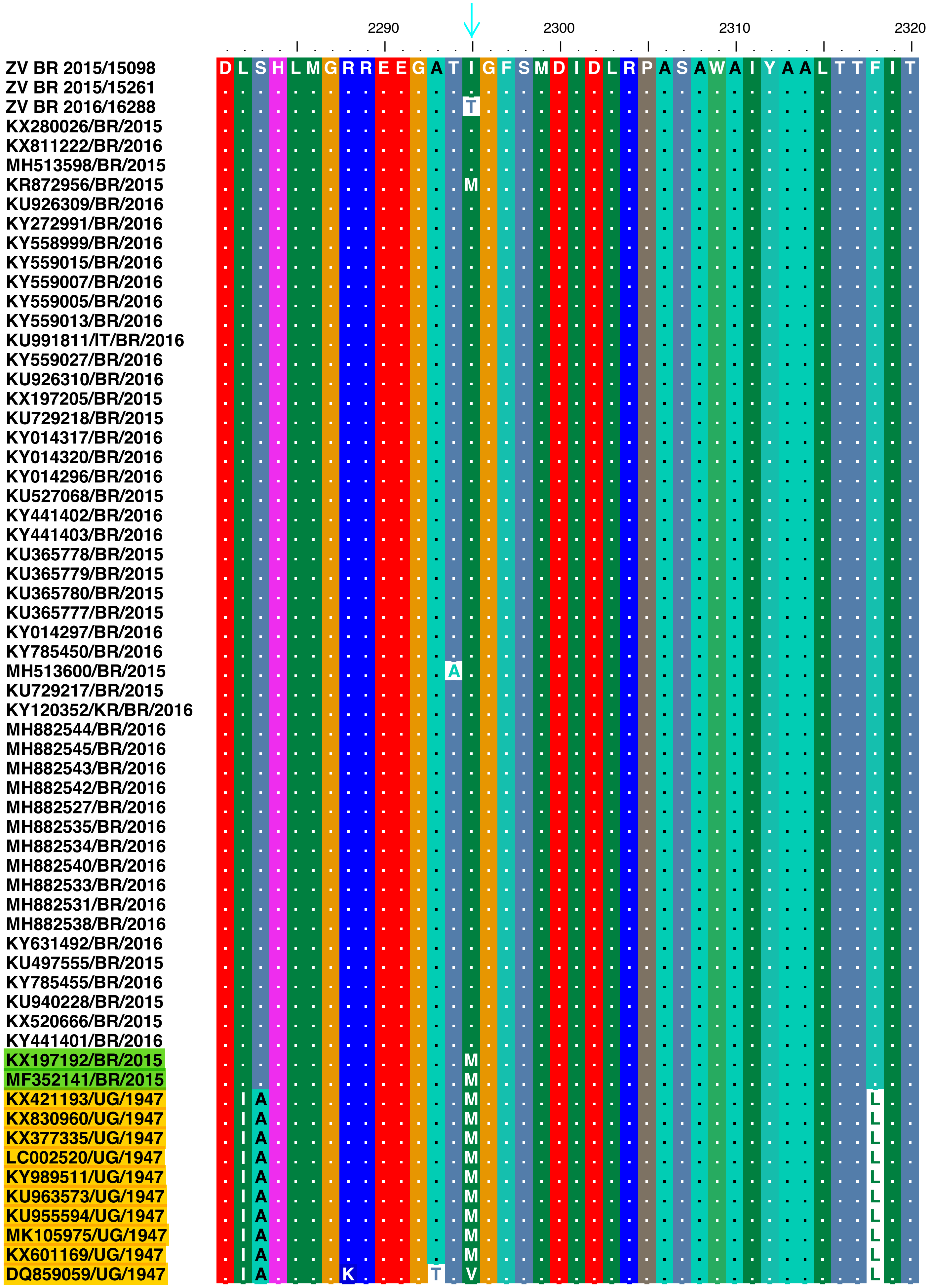
2290
2300
2310
2320
D L S H L M G R R E E G A T I G F S M D I D L R P A S A W A I Y A A L T T F I T
ZV BR 2015/15098
ZV BR 2015/15261
ZV BR 2016/16288
KX280026/BR/2015
KX811222/BR/2016
MH513598/BR/2015
KR872956/BR/2015
KU926309/BR/2016
KY272991/BR/2016
KY558999/BR/2016
KY559015/BR/2016
KY559007/BR/2016
KY559005/BR/2016
KY559013/BR/2016
KU991811/IT/BR/2016
KY559027/BR/2016
KU926310/BR/2016
KX197205/BR/2015
KU729218/BR/2015
KY014317/BR/2016
KY014320/BR/2016
KY014296/BR/2016
KU527068/BR/2015
KY441402/BR/2016
KY441403/BR/2016
KU365778/BR/2015
KU365779/BR/2015
KU365780/BR/2015
KU365777/BR/2015
KY014297/BR/2016
KY785450/BR/2016
MH513600/BR/2015
KU729217/BR/2015
KY120352/KR/BR/2016
MH882544/BR/2016
MH882545/BR/2016
MH882543/BR/2016
MH882542/BR/2016
MH882527/BR/2016
MH882535/BR/2016
MH882534/BR/2016
MH882540/BR/2016
MH882533/BR/2016
MH882531/BR/2016
MH882538/BR/2016
KY631492/BR/2016
KU497555/BR/2015
KY785455/BR/2016
KU940228/BR/2015
KX520666/BR/2015
KY441401/BR/2016
KX197192/BR/2015
MF352141/BR/2015
KX421193/UG/1947
KX830960/UG/1947
KX377335/UG/1947
LC002520/UG/1947
KY989511/UG/1947
KU963573/UG/1947
KU955594/UG/1947
MK105975/UG/1947
KX601169/UG/1947
DQ859059/UG/1947

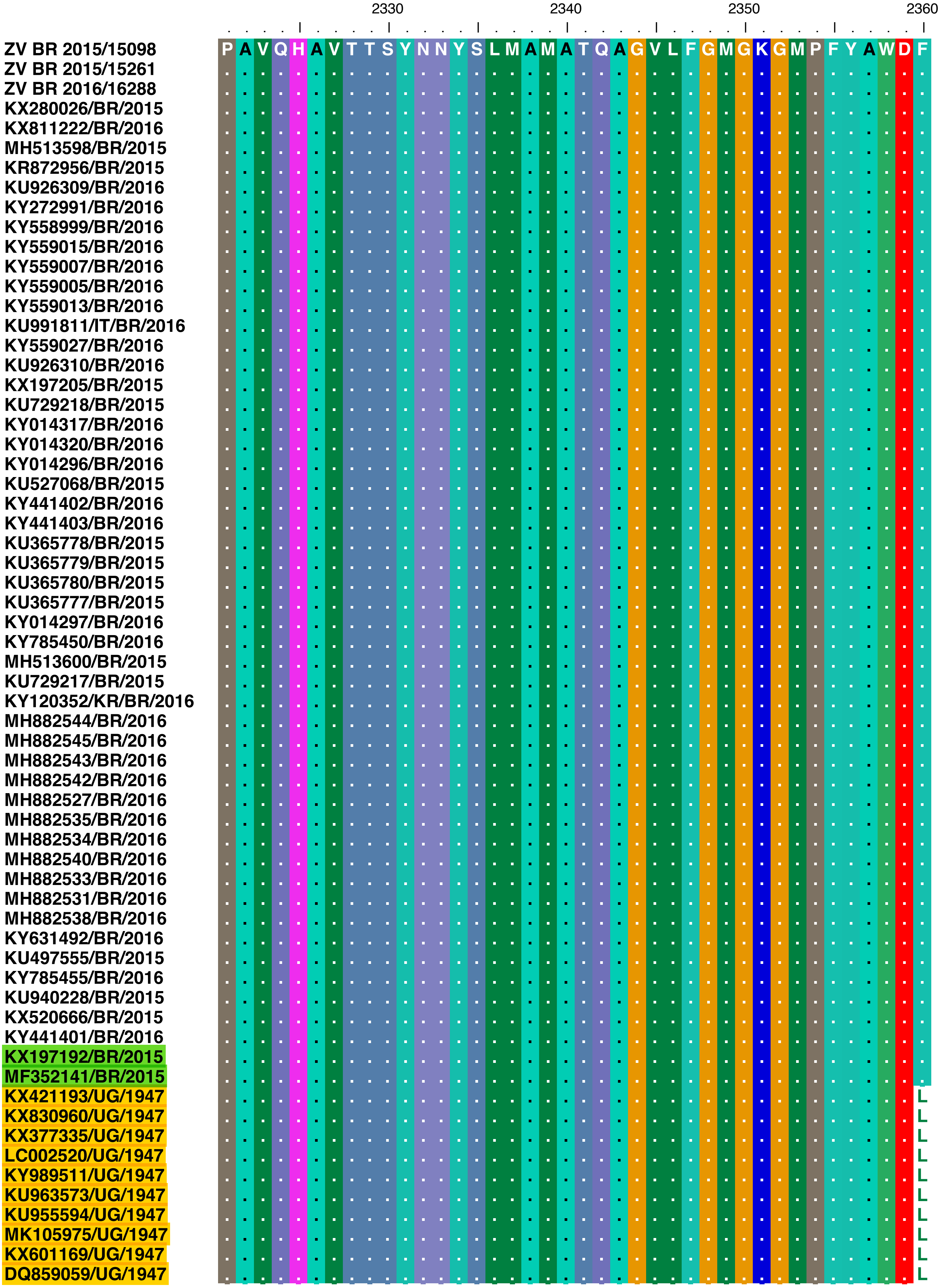
2330
2340
2350
2360
PAVQHAVTTSYNNYSLMAMATQAGVLFGMGKGMPFYAWDF
ZV BR 2015/15098
ZV BR 2015/15261
ZV BR 2016/16288
KX280026/BR/2015
KX811222/BR/2016
MH513598/BR/2015
KR872956/BR/2015
KU926309/BR/2016
KY272991/BR/2016
KY558999/BR/2016
KY559015/BR/2016
KY559007/BR/2016
KY559005/BR/2016
KY559013/BR/2016
KU991811/IT/BR/2016
KY559027/BR/2016
KU926310/BR/2016
KX197205/BR/2015
KU729218/BR/2015
KY014317/BR/2016
KY014320/BR/2016
KY014296/BR/2016
KU527068/BR/2015
KY441402/BR/2016
KY441403/BR/2016
KU365778/BR/2015
KU365779/BR/2015
KU365780/BR/2015
KU365777/BR/2015
KY014297/BR/2016
KY785450/BR/2016
MH513600/BR/2015
KU729217/BR/2015
KY120352/KR/BR/2016
MH882544/BR/2016
MH882545/BR/2016
MH882543/BR/2016
MH882542/BR/2016
MH882527/BR/2016
MH882535/BR/2016
MH882534/BR/2016
MH882540/BR/2016
MH882533/BR/2016
MH882531/BR/2016
MH882538/BR/2016
KY631492/BR/2016
KU497555/BR/2015
KY785455/BR/2016
KU940228/BR/2015
KX520666/BR/2015
KY441401/BR/2016
KX197192/BR/2015
MF352141/BR/2015
KX421193/UG/1947
KX830960/UG/1947
KX377335/UG/1947
LC002520/UG/1947
KY989511/UG/1947
KU963573/UG/1947
KU955594/UG/1947
MK105975/UG/1947
KX601169/UG/1947
DQ859059/UG/1947
L

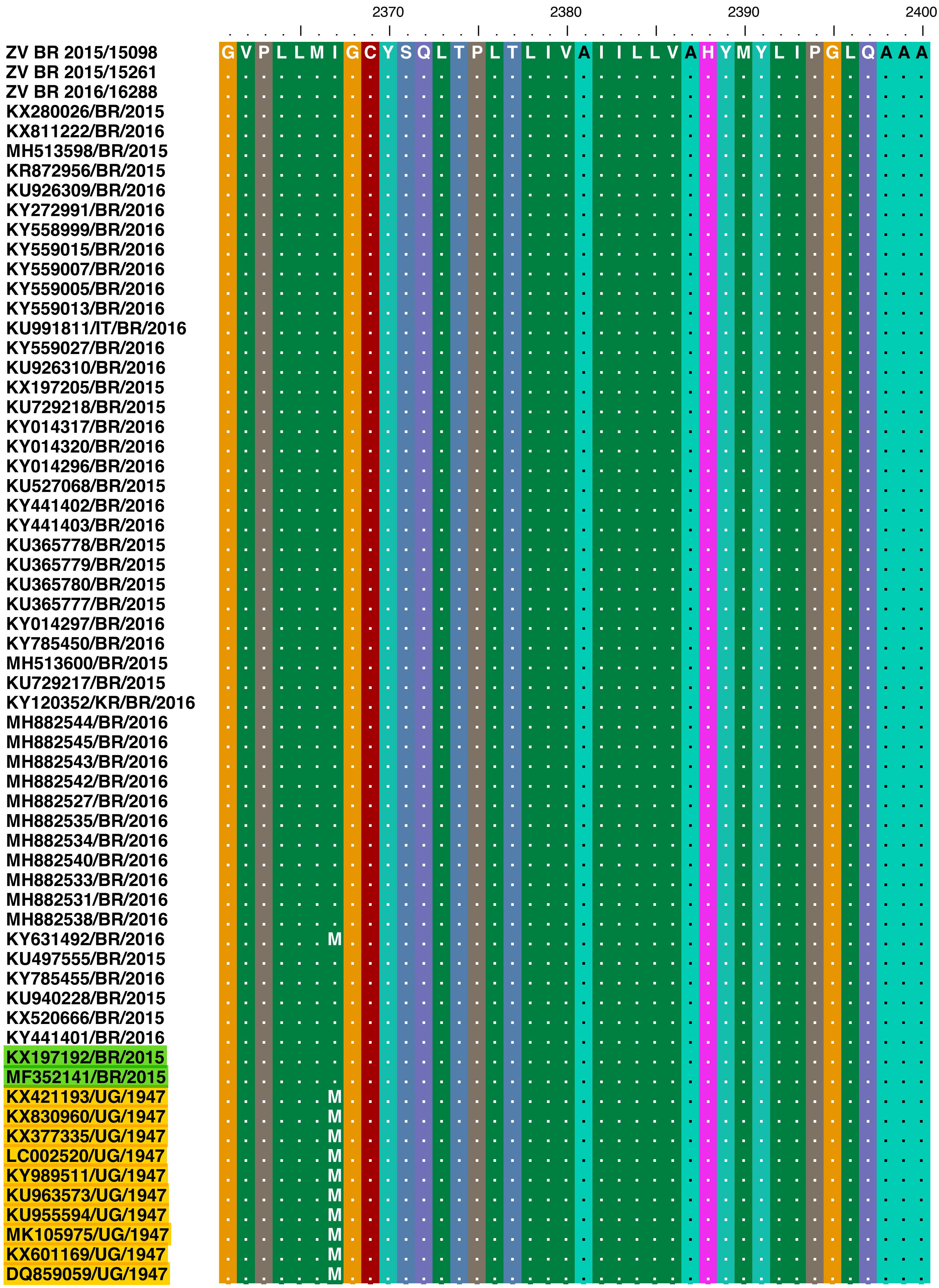
2370 2380 2390 2400
ZV BR 2015/15098 G V P L L M I G C Y S Q L T P L T L I V A I I L L V A H Y M Y L I P G L Q A A A
ZV BR 2015/15261
ZV BR 2016/16288
KX280026/BR/2015
KX811222/BR/2016
MH513598/BR/2015
KR872956/BR/2015
KU926309/BR/2016
KY272991/BR/2016
KY558999/BR/2016
KY559015/BR/2016
KY559007/BR/2016
KY559005/BR/2016
KY559013/BR/2016
KU991811/IT/BR/2016
KY559027/BR/2016
KU926310/BR/2016
KX197205/BR/2015
KU729218/BR/2015
KY014317/BR/2016
KY014320/BR/2016
KY014296/BR/2016
KU527068/BR/2015
KY441402/BR/2016
KY441403/BR/2016
KU365778/BR/2015
KU365779/BR/2015
KU365780/BR/2015
KU365777/BR/2015
KY014297/BR/2016
KY785450/BR/2016
MH513600/BR/2015
KU729217/BR/2015
KY120352/KR/BR/2016
MH882544/BR/2016
MH882545/BR/2016
MH882543/BR/2016
MH882542/BR/2016
MH882527/BR/2016
MH882535/BR/2016
MH882534/BR/2016
MH882540/BR/2016
MH882533/BR/2016
MH882531/BR/2016
MH882538/BR/2016
KY631492/BR/2016 M
KU497555/BR/2015
KY785455/BR/2016
KU940228/BR/2015
KX520666/BR/2015
KY441401/BR/2016
KX197192/BR/2015
MF352141/BR/2015
KX421193/UG/1947 M
KX830960/UG/1947 M
KX377335/UG/1947 M
LC002520/UG/1947 M
KY989511/UG/1947 M
KU963573/UG/1947 M
KU955594/UG/1947 M
MK105975/UG/1947 M
KX601169/UG/1947 M
DQ859059/UG/1947 M

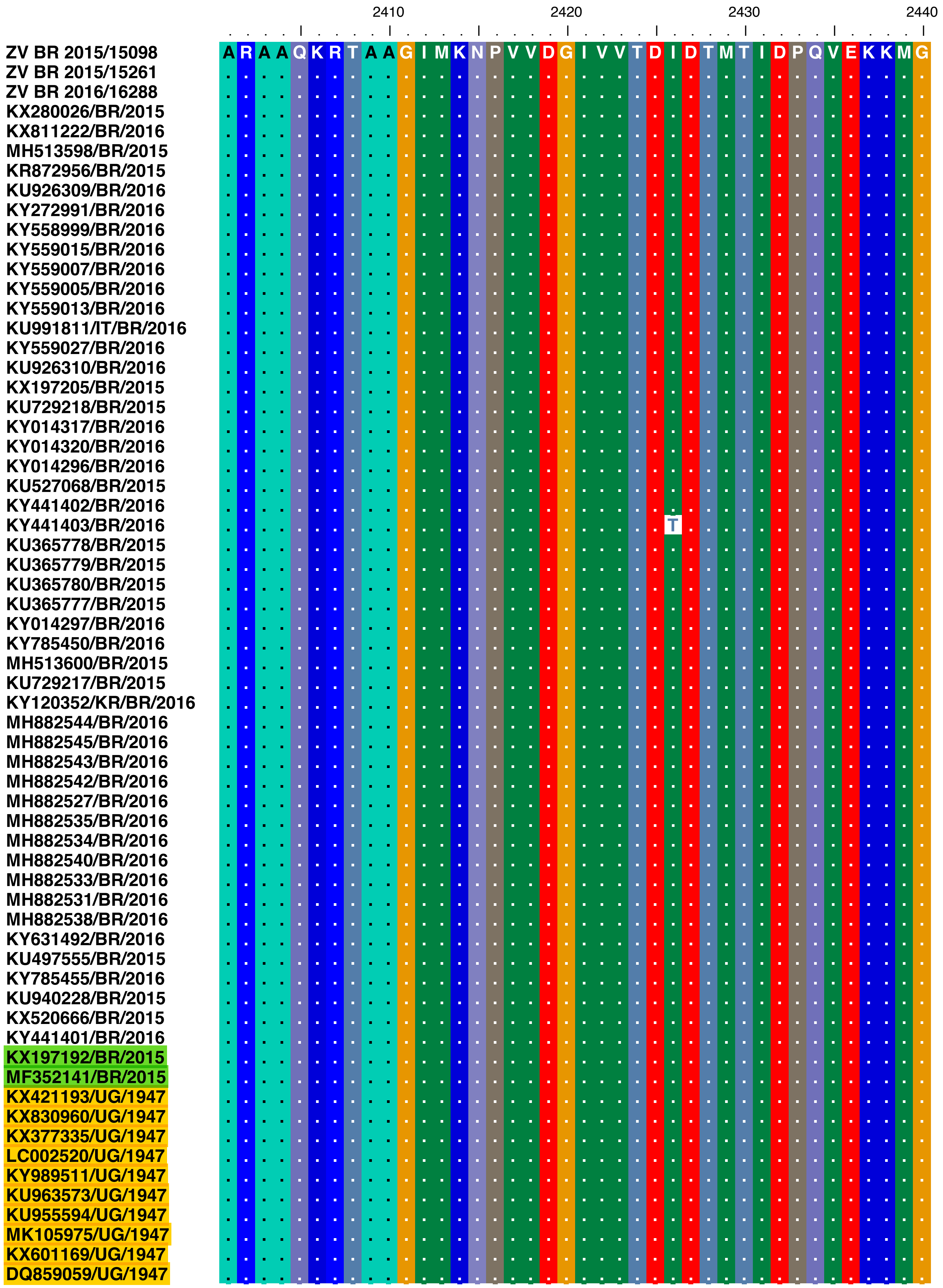
2410
2420
2430
2440
ZV BR 2015/15098 ARAAQKRTAAGIMKNPVVDGIVVTDIDTMTIDPQVEKKMG
ZV BR 2015/15261
ZV BR 2016/16288
KX280026/BR/2015
KX811222/BR/2016
MH513598/BR/2015
KR872956/BR/2015
KU926309/BR/2016
KY272991/BR/2016
KY558999/BR/2016
KY559015/BR/2016
KY559007/BR/2016
KY559005/BR/2016
KY559013/BR/2016
KU991811/IT/BR/2016
KY559027/BR/2016
KU926310/BR/2016
KX197205/BR/2015
KU729218/BR/2015
KY014317/BR/2016
KY014320/BR/2016
KY014296/BR/2016
KU527068/BR/2015
KY441402/BR/2016
KY441403/BR/2016 T
KU365778/BR/2015
KU365779/BR/2015
KU365780/BR/2015
KU365777/BR/2015
KY014297/BR/2016
KY785450/BR/2016
MH513600/BR/2015
KU729217/BR/2015
KY120352/KR/BR/2016
MH882544/BR/2016
MH882545/BR/2016
MH882543/BR/2016
MH882542/BR/2016
MH882527/BR/2016
MH882535/BR/2016
MH882534/BR/2016
MH882540/BR/2016
MH882533/BR/2016
MH882531/BR/2016
MH882538/BR/2016
KY631492/BR/2016
KU497555/BR/2015
KY785455/BR/2016
KU940228/BR/2015
KX520666/BR/2015
KY441401/BR/2016
KX197192/BR/2015
MF352141/BR/2015
KX421193/UG/1947
KX830960/UG/1947
KX377335/UG/1947
LC002520/UG/1947
KY989511/UG/1947
KU963573/UG/1947
KU955594/UG/1947
MK105975/UG/1947
KX601169/UG/1947
DQ859059/UG/1947

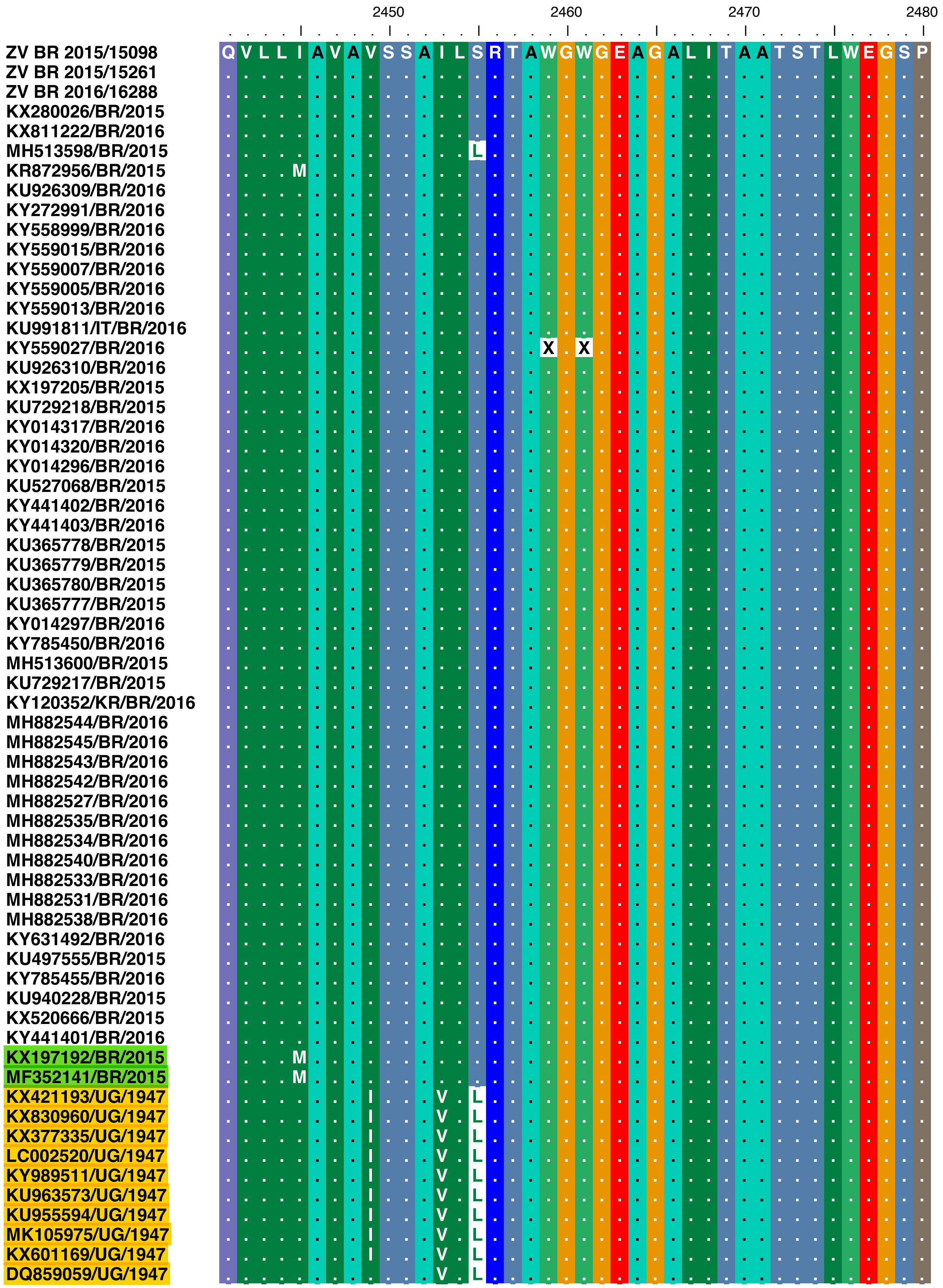

2450
2460
2470
2480
Q V L L I A V A V S S A I L S R T A W G W G E A G A L I T A A T S T L W E G S P
ZV BR 2015/15098
ZV BR 2015/15261
ZV BR 2016/16288
KX280026/BR/2015
KX811222/BR/2016
MH513598/BR/2015
KR872956/BR/2015
KU926309/BR/2016
KY272991/BR/2016
KY558999/BR/2016
KY559015/BR/2016
KY559007/BR/2016
KY559005/BR/2016
KY559013/BR/2016
KU991811/IT/BR/2016
KY559027/BR/2016
KU926310/BR/2016
KX197205/BR/2015
KU729218/BR/2015
KY014317/BR/2016
KY014320/BR/2016
KY014296/BR/2016
KU527068/BR/2015
KY441402/BR/2016
KY441403/BR/2016
KU365778/BR/2015
KU365779/BR/2015
KU365780/BR/2015
KU365777/BR/2015
KY014297/BR/2016
KY785450/BR/2016
MH513600/BR/2015
KU729217/BR/2015
KY120352/KR/BR/2016
MH882544/BR/2016
MH882545/BR/2016
MH882543/BR/2016
MH882542/BR/2016
MH882527/BR/2016
MH882535/BR/2016
MH882534/BR/2016
MH882540/BR/2016
MH882533/BR/2016
MH882531/BR/2016
MH882538/BR/2016
KY631492/BR/2016
KU497555/BR/2015
KY785455/BR/2016
KU940228/BR/2015
KX520666/BR/2015
KY441401/BR/2016
KX197192/BR/2015
MF352141/BR/2015
KX421193/UG/1947
KX830960/UG/1947
KX377335/UG/1947
LC002520/UG/1947
KY989511/UG/1947
KU963573/UG/1947
KU955594/UG/1947
MK105975/UG/1947
KX601169/UG/1947
DQ859059/UG/1947

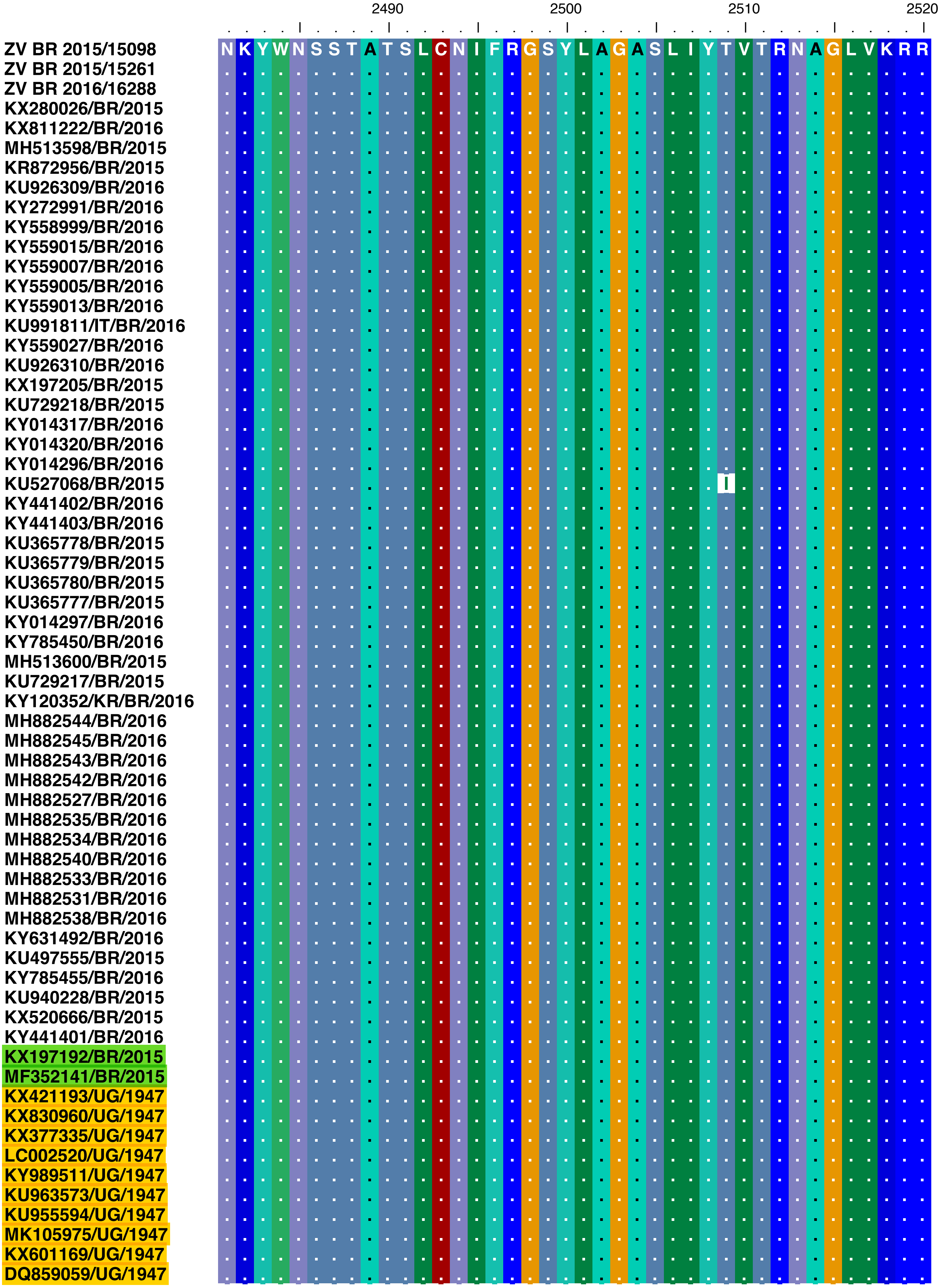
2490
2500
2510
2520
NKYWNSSTATSLCNIFRGSYLAGASLIYTVTRNAGLVKRR
ZV BR 2015/15098
ZV BR 2015/15261
ZV BR 2016/16288
KX280026/BR/2015
KX811222/BR/2016
MH513598/BR/2015
KR872956/BR/2015
KU926309/BR/2016
KY272991/BR/2016
KY558999/BR/2016
KY559015/BR/2016
KY559007/BR/2016
KY559005/BR/2016
KY559013/BR/2016
KU991811/IT/BR/2016
KY559027/BR/2016
KU926310/BR/2016
KX197205/BR/2015
KU729218/BR/2015
KY014317/BR/2016
KY014320/BR/2016
KY014296/BR/2016
KU527068/BR/2015
I
KY441402/BR/2016
KY441403/BR/2016
KU365778/BR/2015
KU365779/BR/2015
KU365780/BR/2015
KU365777/BR/2015
KY014297/BR/2016
KY785450/BR/2016
MH513600/BR/2015
KU729217/BR/2015
KY120352/KR/BR/2016
MH882544/BR/2016
MH882545/BR/2016
MH882543/BR/2016
MH882542/BR/2016
MH882527/BR/2016
MH882535/BR/2016
MH882534/BR/2016
MH882540/BR/2016
MH882533/BR/2016
MH882531/BR/2016
MH882538/BR/2016
KY631492/BR/2016
KU497555/BR/2015
KY785455/BR/2016
KU940228/BR/2015
KX520666/BR/2015
KY441401/BR/2016
KX197192/BR/2015
MF352141/BR/2015
KX421193/UG/1947
KX830960/UG/1947
KX377335/UG/1947
LC002520/UG/1947
KY989511/UG/1947
KU963573/UG/1947
KU955594/UG/1947
MK105975/UG/1947
KX601169/UG/1947
DQ859059/UG/1947

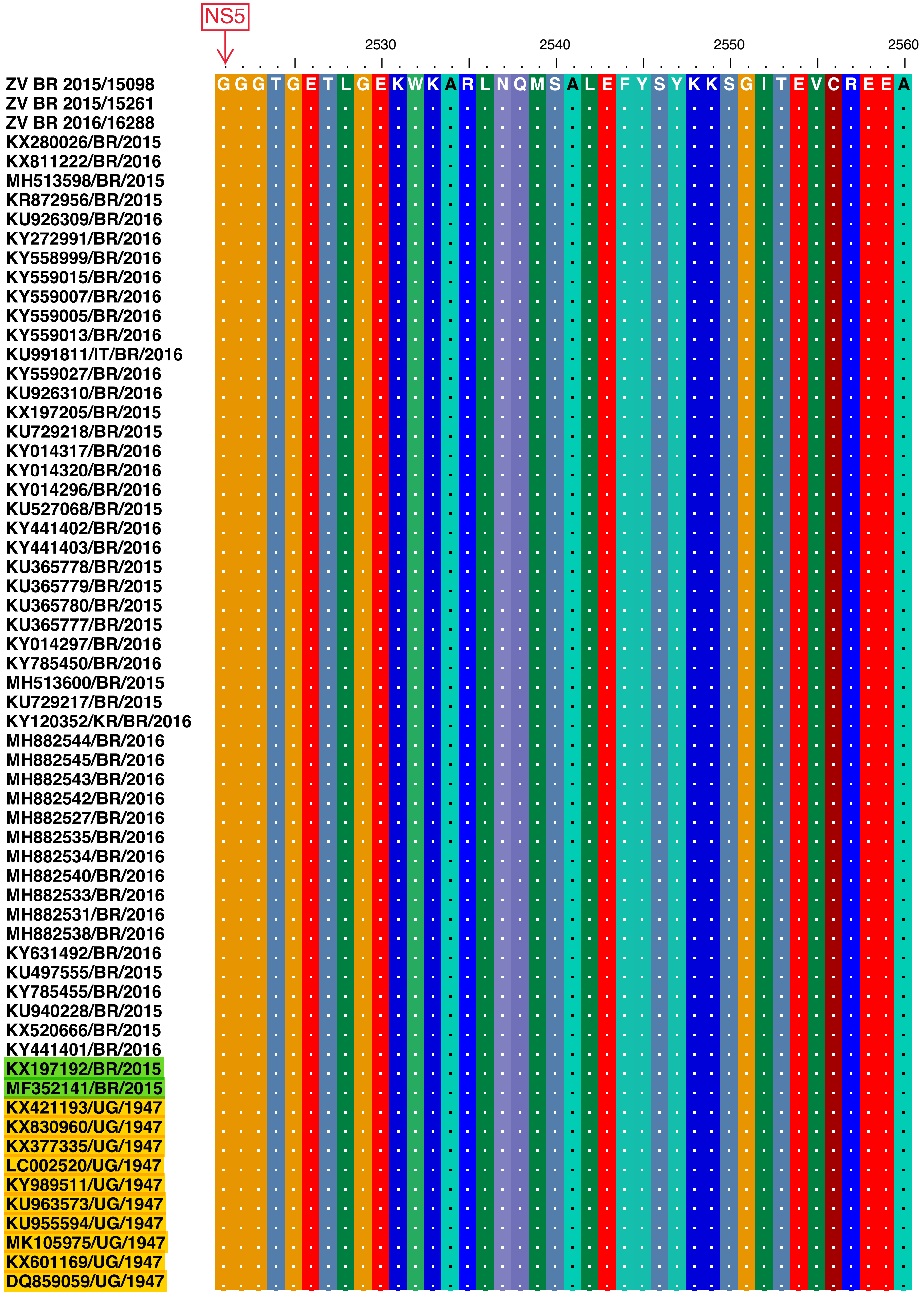
NS5
2530
2540
2550
2560
GGGTGETLGEKWKARLNQMSALEFYSYKKSGITEVCREEA
ZV BR 2015/15098
ZV BR 2015/15261
ZV BR 2016/16288
KX280026/BR/2015
KX811222/BR/2016
MH513598/BR/2015
KR872956/BR/2015
KU926309/BR/2016
KY272991/BR/2016
KY558999/BR/2016
KY559015/BR/2016
KY559007/BR/2016
KY559005/BR/2016
KY559013/BR/2016
KU991811/IT/BR/2016
KY559027/BR/2016
KU926310/BR/2016
KX197205/BR/2015
KU729218/BR/2015
KY014317/BR/2016
KY014320/BR/2016
KY014296/BR/2016
KU527068/BR/2015
KY441402/BR/2016
KY441403/BR/2016
KU365778/BR/2015
KU365779/BR/2015
KU365780/BR/2015
KU365777/BR/2015
KY014297/BR/2016
KY785450/BR/2016
MH513600/BR/2015
KU729217/BR/2015
KY120352/KR/BR/2016
MH882544/BR/2016
MH882545/BR/2016
MH882543/BR/2016
MH882542/BR/2016
MH882527/BR/2016
MH882535/BR/2016
MH882534/BR/2016
MH882540/BR/2016
MH882533/BR/2016
MH882531/BR/2016
MH882538/BR/2016
KY631492/BR/2016
KU497555/BR/2015
KY785455/BR/2016
KU940228/BR/2015
KX520666/BR/2015
KY441401/BR/2016
KX197192/BR/2015
MF352141/BR/2015
KX421193/UG/1947
KX830960/UG/1947
KX377335/UG/1947
LC002520/UG/1947
KY989511/UG/1947
KU963573/UG/1947
KU955594/UG/1947
MK105975/UG/1947
KX601169/UG/1947
DQ859059/UG/1947

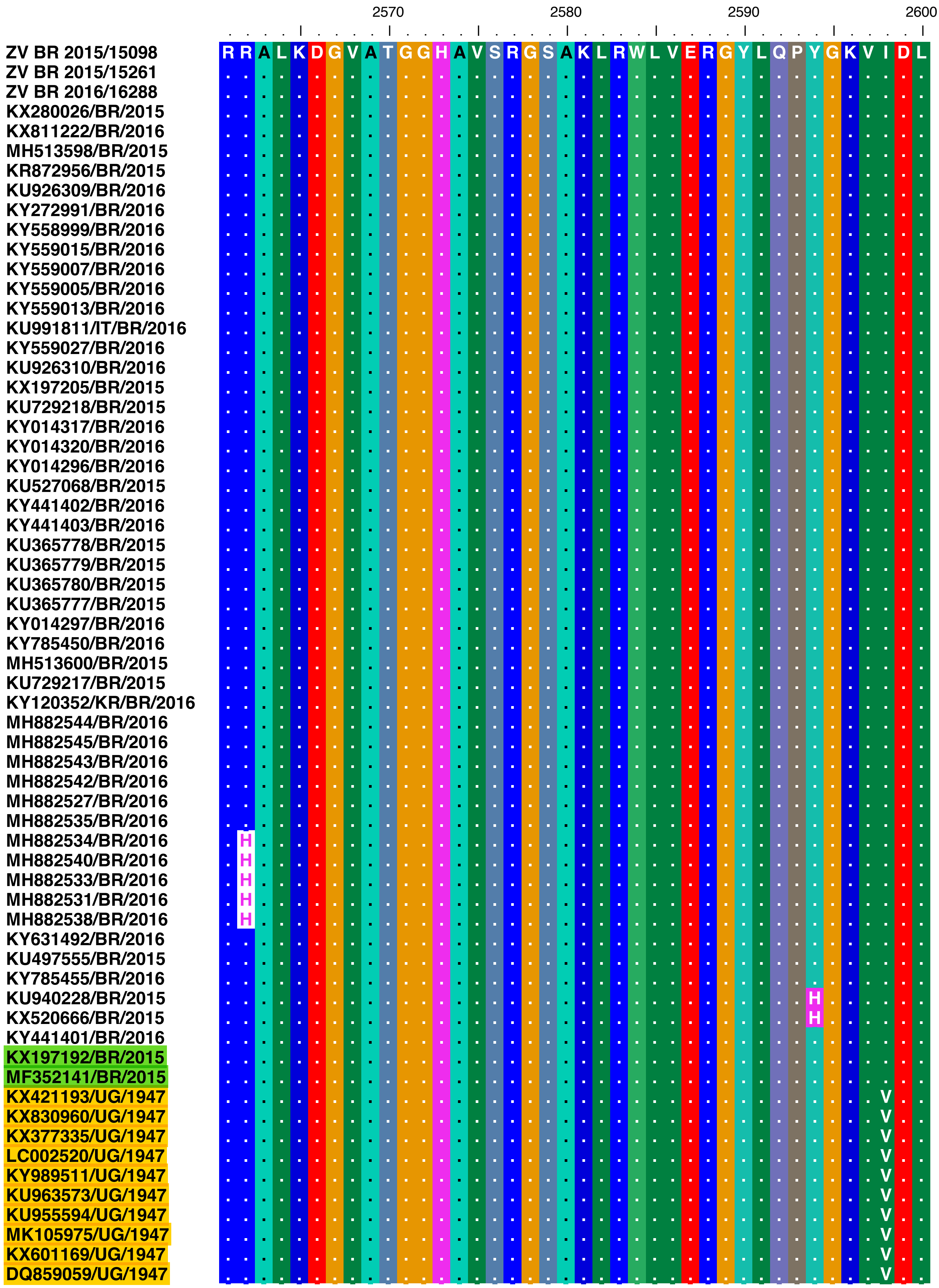
2570
2580
2590
2600
ZV BR 2015/15098 R R A L K D G V A T G G H A V S R G S A K L R W L V E R G Y L Q P Y G K V I D L
ZV BR 2015/15261
ZV BR 2016/16288
KX280026/BR/2015
KX811222/BR/2016
MH513598/BR/2015
KR872956/BR/2015
KU926309/BR/2016
KY272991/BR/2016
KY558999/BR/2016
KY559015/BR/2016
KY559007/BR/2016
KY559005/BR/2016
KY559013/BR/2016
KU991811/IT/BR/2016
KY559027/BR/2016
KU926310/BR/2016
KX197205/BR/2015
KU729218/BR/2015
KY014317/BR/2016
KY014320/BR/2016
KY014296/BR/2016
KU527068/BR/2015
KY441402/BR/2016
KY441403/BR/2016
KU365778/BR/2015
KU365779/BR/2015
KU365780/BR/2015
KU365777/BR/2015
KY014297/BR/2016
KY785450/BR/2016
MH513600/BR/2015
KU729217/BR/2015
KY120352/KR/BR/2016
MH882544/BR/2016
MH882545/BR/2016
MH882543/BR/2016
MH882542/BR/2016
MH882527/BR/2016
MH882535/BR/2016
MH882534/BR/2016 H
MH882540/BR/2016 H
MH882533/BR/2016 H
MH882531/BR/2016 H
MH882538/BR/2016 H
KY631492/BR/2016
KU497555/BR/2015
KY785455/BR/2016
KU940228/BR/2015 H
KX520666/BR/2015 H
KY441401/BR/2016
KX197192/BR/2015
MF352141/BR/2015
KX421193/UG/1947 V
KX830960/UG/1947 V
KX377335/UG/1947 V
LC002520/UG/1947 V
KY989511/UG/1947 V
KU963573/UG/1947 V
KU955594/UG/1947 V
MK105975/UG/1947 V
KX601169/UG/1947 V
DQ859059/UG/1947 V

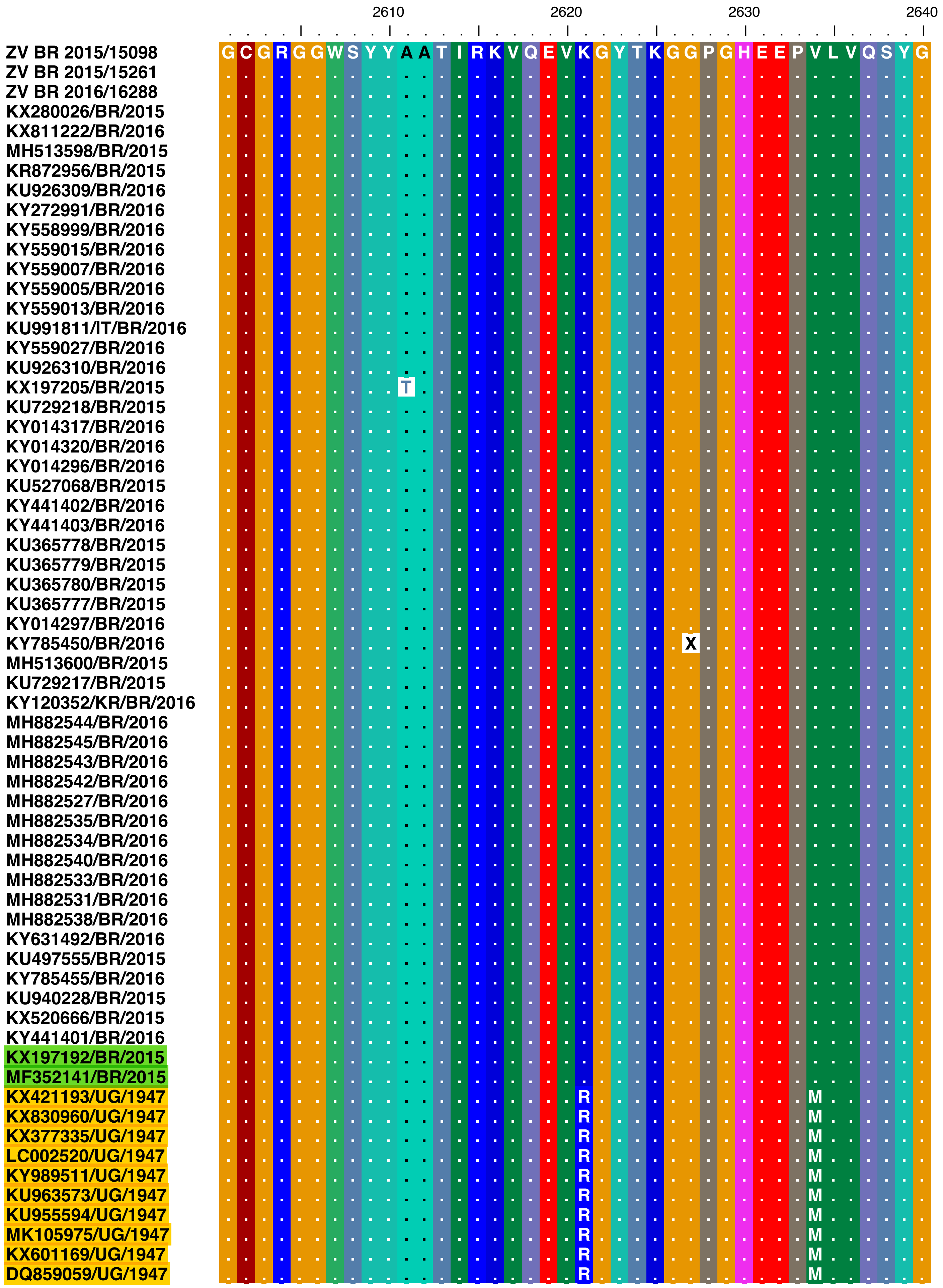

2610
2620
2630
2640
ZV BR 2015/15098
G C G R G G W S Y Y A A T I R K V Q E V K G Y T K G G P G H E E P V L V Q S Y G
ZV BR 2015/15261
ZV BR 2016/16288
KX280026/BR/2015
KX811222/BR/2016
MH513598/BR/2015
KR872956/BR/2015
KU926309/BR/2016
KY272991/BR/2016
KY558999/BR/2016
KY559015/BR/2016
KY559007/BR/2016
KY559005/BR/2016
KY559013/BR/2016
KU991811/IT/BR/2016
KY559027/BR/2016
KU926310/BR/2016
KX197205/BR/2015
KU729218/BR/2015
KY014317/BR/2016
KY014320/BR/2016
KY014296/BR/2016
KU527068/BR/2015
KY441402/BR/2016
KY441403/BR/2016
KU365778/BR/2015
KU365779/BR/2015
KU365780/BR/2015
KU365777/BR/2015
KY014297/BR/2016
KY785450/BR/2016
MH513600/BR/2015
KU729217/BR/2015
KY120352/KR/BR/2016
MH882544/BR/2016
MH882545/BR/2016
MH882543/BR/2016
MH882542/BR/2016
MH882527/BR/2016
MH882535/BR/2016
MH882534/BR/2016
MH882540/BR/2016
MH882533/BR/2016
MH882531/BR/2016
MH882538/BR/2016
KY631492/BR/2016
KU497555/BR/2015
KY785455/BR/2016
KU940228/BR/2015
KX520666/BR/2015
KY441401/BR/2016
KX197192/BR/2015
MF352141/BR/2015
KX421193/UG/1947
KX830960/UG/1947
KX377335/UG/1947
LC002520/UG/1947
KY989511/UG/1947
KU963573/UG/1947
KU955594/UG/1947
MK105975/UG/1947
KX601169/UG/1947
DQ859059/UG/1947

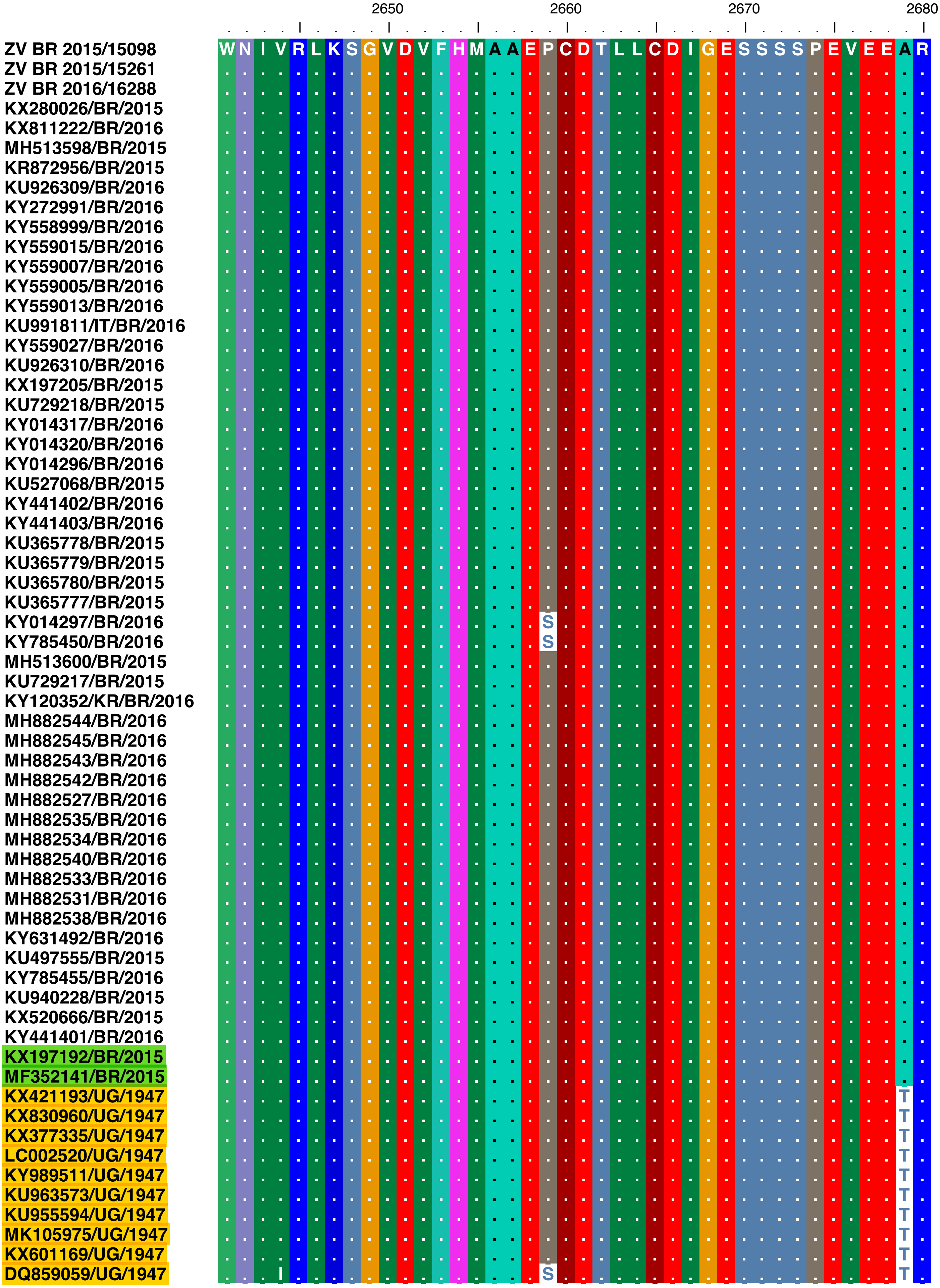
2650
2660
2670
2680
W N I V R L K S G V D V F H M A A E P C D T L L C D I G E S S S S P E V E E A R
ZV BR 2015/15098
ZV BR 2015/15261
ZV BR 2016/16288
KX280026/BR/2015
KX811222/BR/2016
MH513598/BR/2015
KR872956/BR/2015
KU926309/BR/2016
KY272991/BR/2016
KY558999/BR/2016
KY559015/BR/2016
KY559007/BR/2016
KY559005/BR/2016
KY559013/BR/2016
KU991811/IT/BR/2016
KY559027/BR/2016
KU926310/BR/2016
KX197205/BR/2015
KU729218/BR/2015
KY014317/BR/2016
KY014320/BR/2016
KY014296/BR/2016
KU527068/BR/2015
KY441402/BR/2016
KY441403/BR/2016
KU365778/BR/2015
KU365779/BR/2015
KU365780/BR/2015
KU365777/BR/2015
KY014297/BR/2016
KY785450/BR/2016
MH513600/BR/2015
KU729217/BR/2015
KY120352/KR/BR/2016
MH882544/BR/2016
MH882545/BR/2016
MH882543/BR/2016
MH882542/BR/2016
MH882527/BR/2016
MH882535/BR/2016
MH882534/BR/2016
MH882540/BR/2016
MH882533/BR/2016
MH882531/BR/2016
MH882538/BR/2016
KY631492/BR/2016
KU497555/BR/2015
KY785455/BR/2016
KU940228/BR/2015
KX520666/BR/2015
KY441401/BR/2016
KX197192/BR/2015
MF352141/BR/2015
KX421193/UG/1947
KX830960/UG/1947
KX377335/UG/1947
LC002520/UG/1947
KY989511/UG/1947
KU963573/UG/1947
KU955594/UG/1947
MK105975/UG/1947
KX601169/UG/1947
DQ859059/UG/1947
S
S
T
T
T
T
T
T
T
T
T
T
I
S

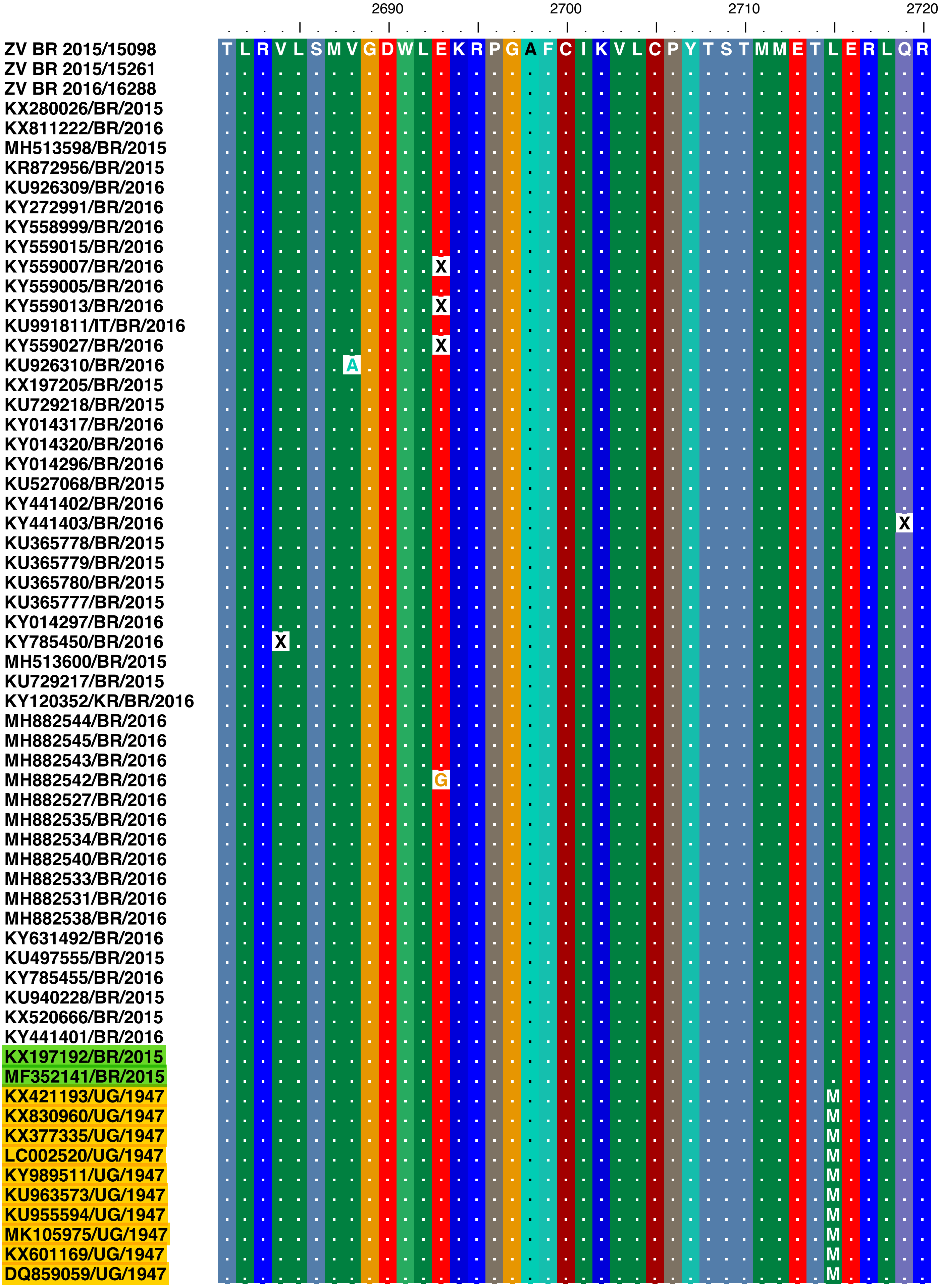

2690
2700
2710
2720
T L R V L S M V G D W L E K R P G A F C I K V L C P Y T S T M M E T L E R L Q R
ZV BR 2015/15098
ZV BR 2015/15261
ZV BR 2016/16288
KX280026/BR/2015
KX811222/BR/2016
MH513598/BR/2015
KR872956/BR/2015
KU926309/BR/2016
KY272991/BR/2016
KY558999/BR/2016
KY559015/BR/2016
KY559007/BR/2016
KY559005/BR/2016
KY559013/BR/2016
KU991811/IT/BR/2016
KY559027/BR/2016
KU926310/BR/2016
KX197205/BR/2015
KU729218/BR/2015
KY014317/BR/2016
KY014320/BR/2016
KY014296/BR/2016
KU527068/BR/2015
KY441402/BR/2016
KY441403/BR/2016
KU365778/BR/2015
KU365779/BR/2015
KU365780/BR/2015
KU365777/BR/2015
KY014297/BR/2016
KY785450/BR/2016
MH513600/BR/2015
KU729217/BR/2015
KY120352/KR/BR/2016
MH882544/BR/2016
MH882545/BR/2016
MH882543/BR/2016
MH882542/BR/2016
MH882527/BR/2016
MH882535/BR/2016
MH882534/BR/2016
MH882540/BR/2016
MH882533/BR/2016
MH882531/BR/2016
MH882538/BR/2016
KY631492/BR/2016
KU497555/BR/2015
KY785455/BR/2016
KU940228/BR/2015
KX520666/BR/2015
KY441401/BR/2016
KX197192/BR/2015
MF352141/BR/2015
KX421193/UG/1947
KX830960/UG/1947
KX377335/UG/1947
LC002520/UG/1947
KY989511/UG/1947
KU963573/UG/1947
KU955594/UG/1947
MK105975/UG/1947
KX601169/UG/1947
DQ859059/UG/1947
X
X
X
A
X
X
G
M

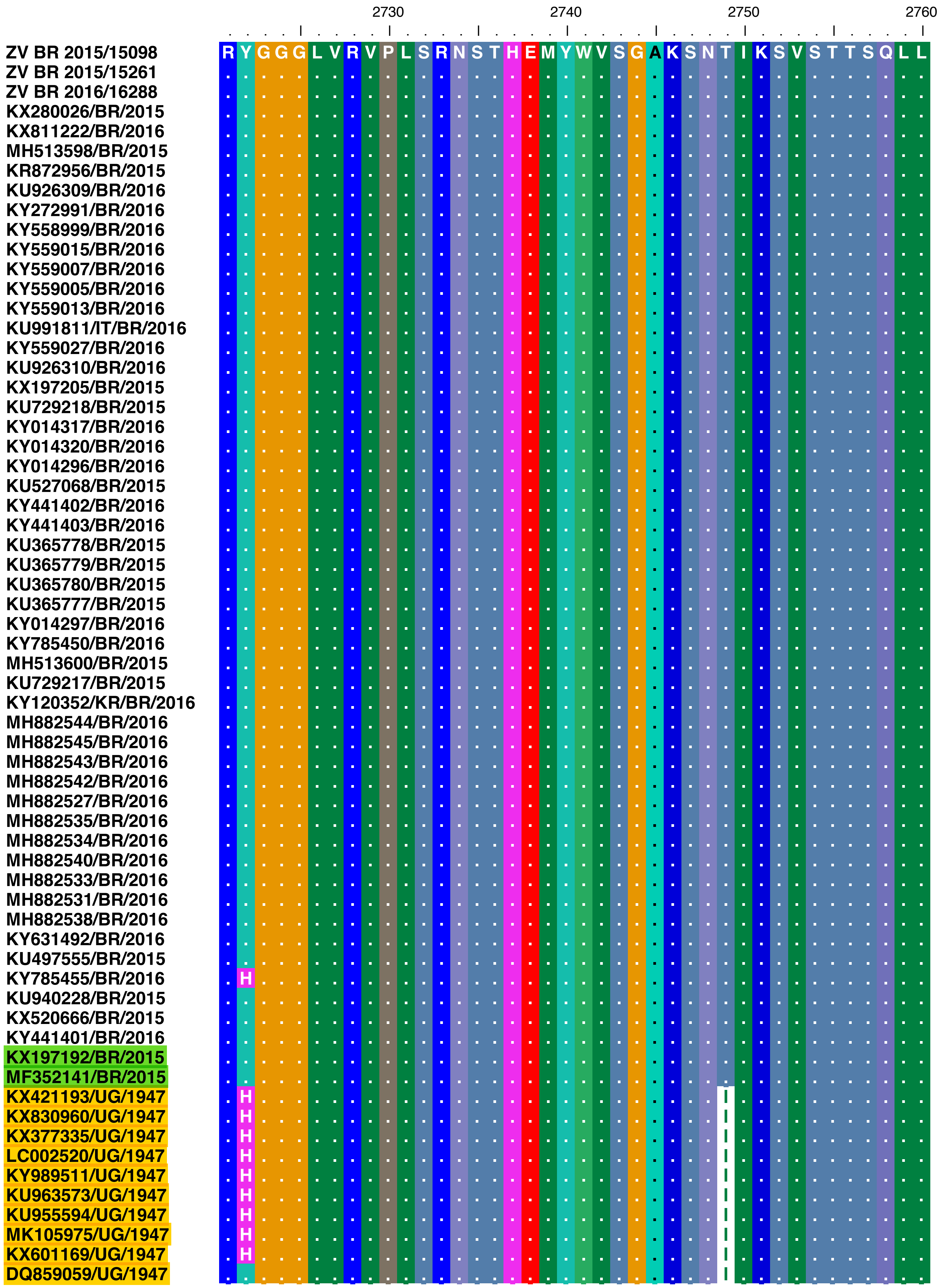
2730
2740
2750
2760
R Y G G G L V R V P L S R N S T H E M Y W V S G A K S N T I K S V S T T S Q L L
ZV BR 2015/15098
ZV BR 2015/15261
ZV BR 2016/16288
KX280026/BR/2015
KX811222/BR/2016
MH513598/BR/2015
KR872956/BR/2015
KU926309/BR/2016
KY272991/BR/2016
KY558999/BR/2016
KY559015/BR/2016
KY559007/BR/2016
KY559005/BR/2016
KY559013/BR/2016
KU991811/IT/BR/2016
KY559027/BR/2016
KU926310/BR/2016
KX197205/BR/2015
KU729218/BR/2015
KY014317/BR/2016
KY014320/BR/2016
KY014296/BR/2016
KU527068/BR/2015
KY441402/BR/2016
KY441403/BR/2016
KU365778/BR/2015
KU365779/BR/2015
KU365780/BR/2015
KU365777/BR/2015
KY014297/BR/2016
KY785450/BR/2016
MH513600/BR/2015
KU729217/BR/2015
KY120352/KR/BR/2016
MH882544/BR/2016
MH882545/BR/2016
MH882543/BR/2016
MH882542/BR/2016
MH882527/BR/2016
MH882535/BR/2016
MH882534/BR/2016
MH882540/BR/2016
MH882533/BR/2016
MH882531/BR/2016
MH882538/BR/2016
KY631492/BR/2016
KU497555/BR/2015
KY785455/BR/2016
KU940228/BR/2015
KX520666/BR/2015
KY441401/BR/2016
KX197192/BR/2015
MF352141/BR/2015
KX421193/UG/1947
KX830960/UG/1947
KX377335/UG/1947
LC002520/UG/1947
KY989511/UG/1947
KU963573/UG/1947
KU955594/UG/1947
MK105975/UG/1947
KX601169/UG/1947
DQ859059/UG/1947
H

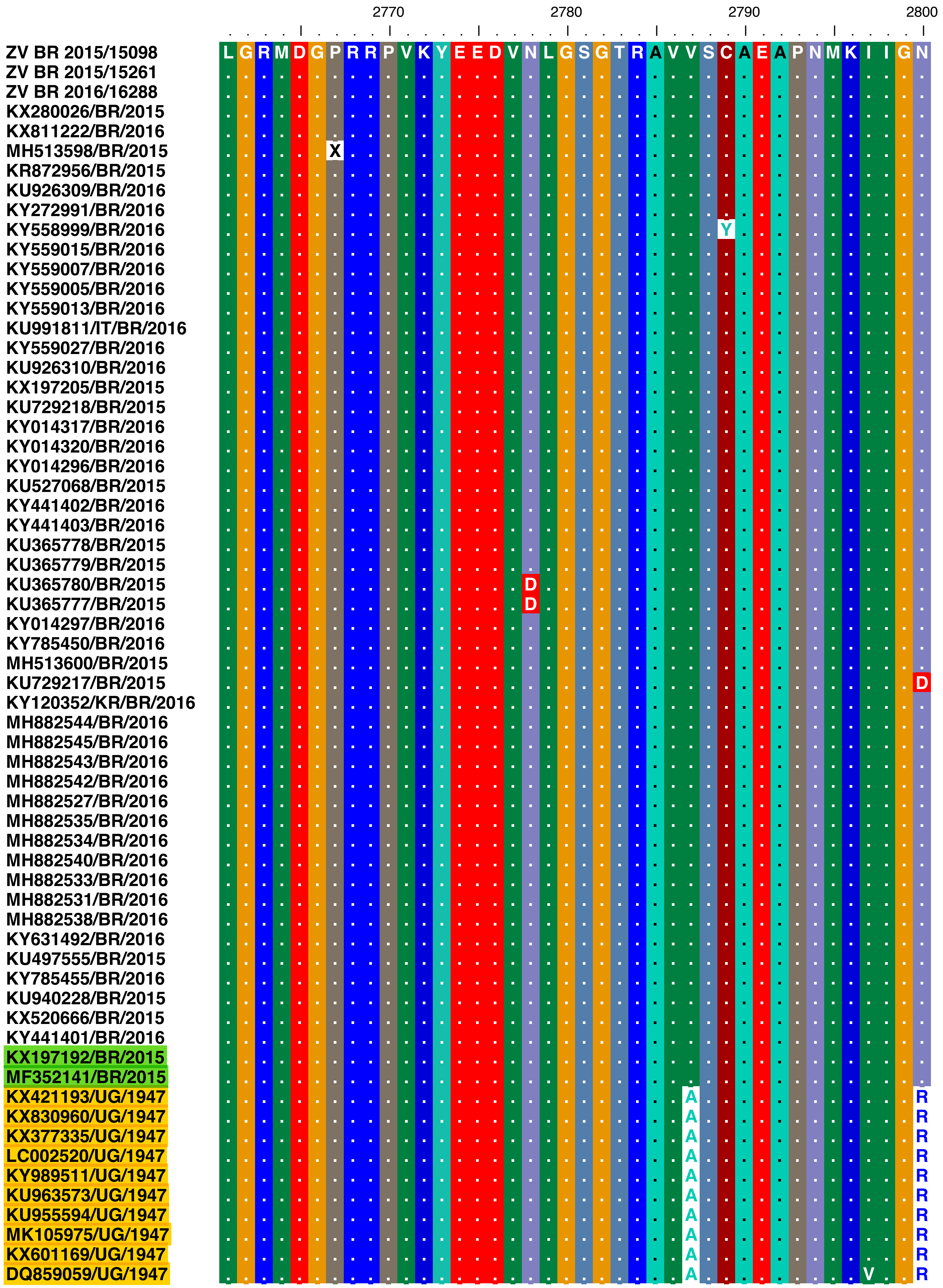

2770
2780
2790
2800
LGRMDGPRRPVKYEEDVNLGSGTRAVVSCAEAPNMKIIGN
ZV BR 2015/15098
ZV BR 2015/15261
ZV BR 2016/16288
KX280026/BR/2015
KX811222/BR/2016
MH513598/BR/2015
KR872956/BR/2015
KU926309/BR/2016
KY272991/BR/2016
KY558999/BR/2016
KY559015/BR/2016
KY559007/BR/2016
KY559005/BR/2016
KY559013/BR/2016
KU991811/IT/BR/2016
KY559027/BR/2016
KU926310/BR/2016
KX197205/BR/2015
KU729218/BR/2015
KY014317/BR/2016
KY014320/BR/2016
KY014296/BR/2016
KU527068/BR/2015
KY441402/BR/2016
KY441403/BR/2016
KU365778/BR/2015
KU365779/BR/2015
KU365780/BR/2015
KU365777/BR/2015
KY014297/BR/2016
KY785450/BR/2016
MH513600/BR/2015
KU729217/BR/2015
KY120352/KR/BR/2016
MH882544/BR/2016
MH882545/BR/2016
MH882543/BR/2016
MH882542/BR/2016
MH882527/BR/2016
MH882535/BR/2016
MH882534/BR/2016
MH882540/BR/2016
MH882533/BR/2016
MH882531/BR/2016
MH882538/BR/2016
KY631492/BR/2016
KU497555/BR/2015
KY785455/BR/2016
KU940228/BR/2015
KX520666/BR/2015
KY441401/BR/2016
KX197192/BR/2015
MF352141/BR/2015
KX421193/UG/1947
KX830960/UG/1947
KX377335/UG/1947
LC002520/UG/1947
KY989511/UG/1947
KU963573/UG/1947
KU955594/UG/1947
MK105975/UG/1947
KX601169/UG/1947
DQ859059/UG/1947
X
Y
D
D
D
A
R
V

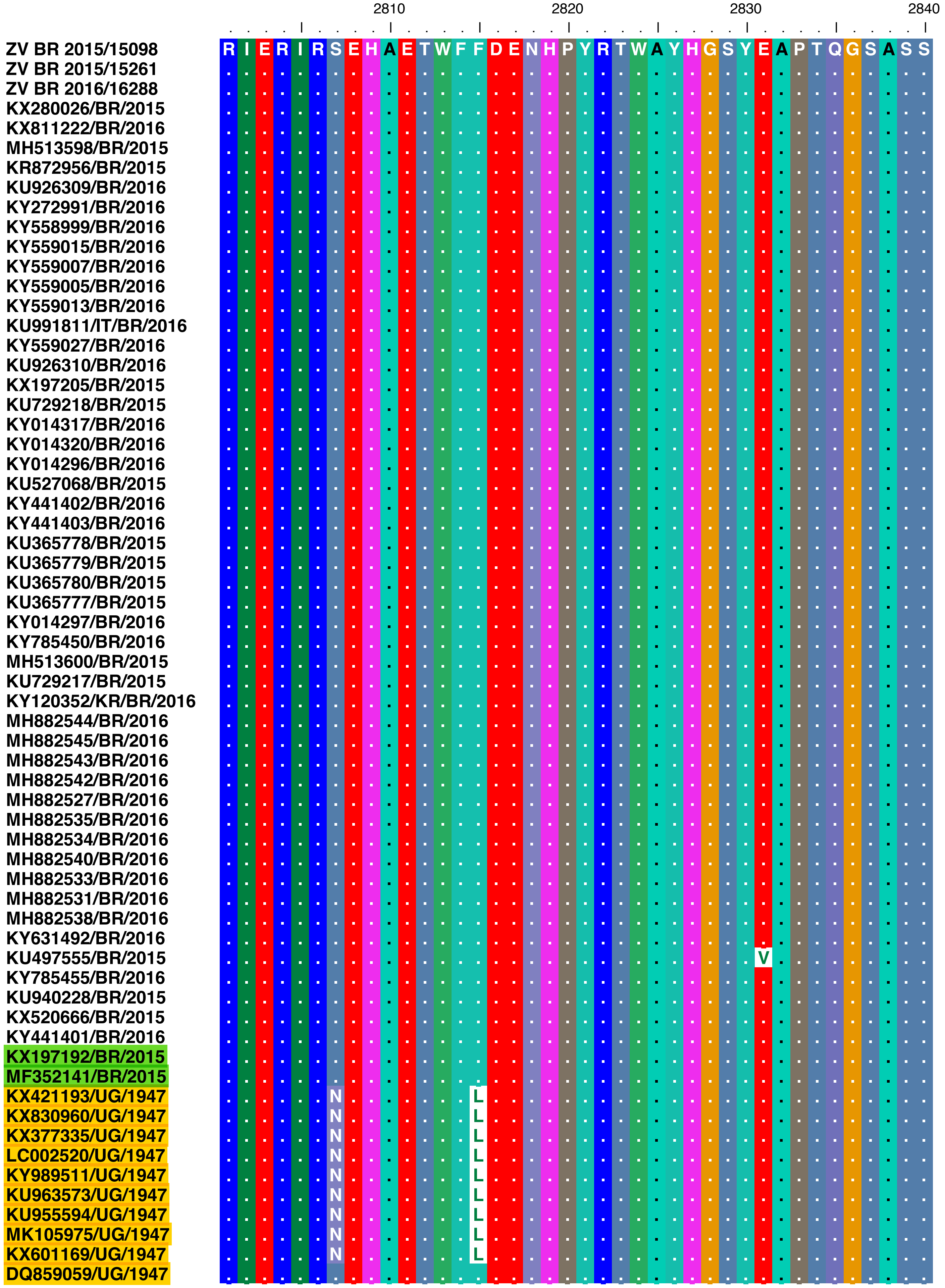

2810
2820
2830
2840
R I E R I R S E H A E T W F F D E N H P Y R T W A Y H G S Y E A P T Q G S A S S
ZV BR 2015/15098
ZV BR 2015/15261
ZV BR 2016/16288
KX280026/BR/2015
KX811222/BR/2016
MH513598/BR/2015
KR872956/BR/2015
KU926309/BR/2016
KY272991/BR/2016
KY558999/BR/2016
KY559015/BR/2016
KY559007/BR/2016
KY559005/BR/2016
KY559013/BR/2016
KU991811/IT/BR/2016
KY559027/BR/2016
KU926310/BR/2016
KX197205/BR/2015
KU729218/BR/2015
KY014317/BR/2016
KY014320/BR/2016
KY014296/BR/2016
KU527068/BR/2015
KY441402/BR/2016
KY441403/BR/2016
KU365778/BR/2015
KU365779/BR/2015
KU365780/BR/2015
KU365777/BR/2015
KY014297/BR/2016
KY785450/BR/2016
MH513600/BR/2015
KU729217/BR/2015
KY120352/KR/BR/2016
MH882544/BR/2016
MH882545/BR/2016
MH882543/BR/2016
MH882542/BR/2016
MH882527/BR/2016
MH882535/BR/2016
MH882534/BR/2016
MH882540/BR/2016
MH882533/BR/2016
MH882531/BR/2016
MH882538/BR/2016
KY631492/BR/2016
KU497555/BR/2015
KY785455/BR/2016
KU940228/BR/2015
KX520666/BR/2015
KY441401/BR/2016
KX197192/BR/2015
MF352141/BR/2015
KX421193/UG/1947
KX830960/UG/1947
KX377335/UG/1947
LC002520/UG/1947
KY989511/UG/1947
KU963573/UG/1947
KU955594/UG/1947
MK105975/UG/1947
KX601169/UG/1947
DQ859059/UG/1947
V
N
L

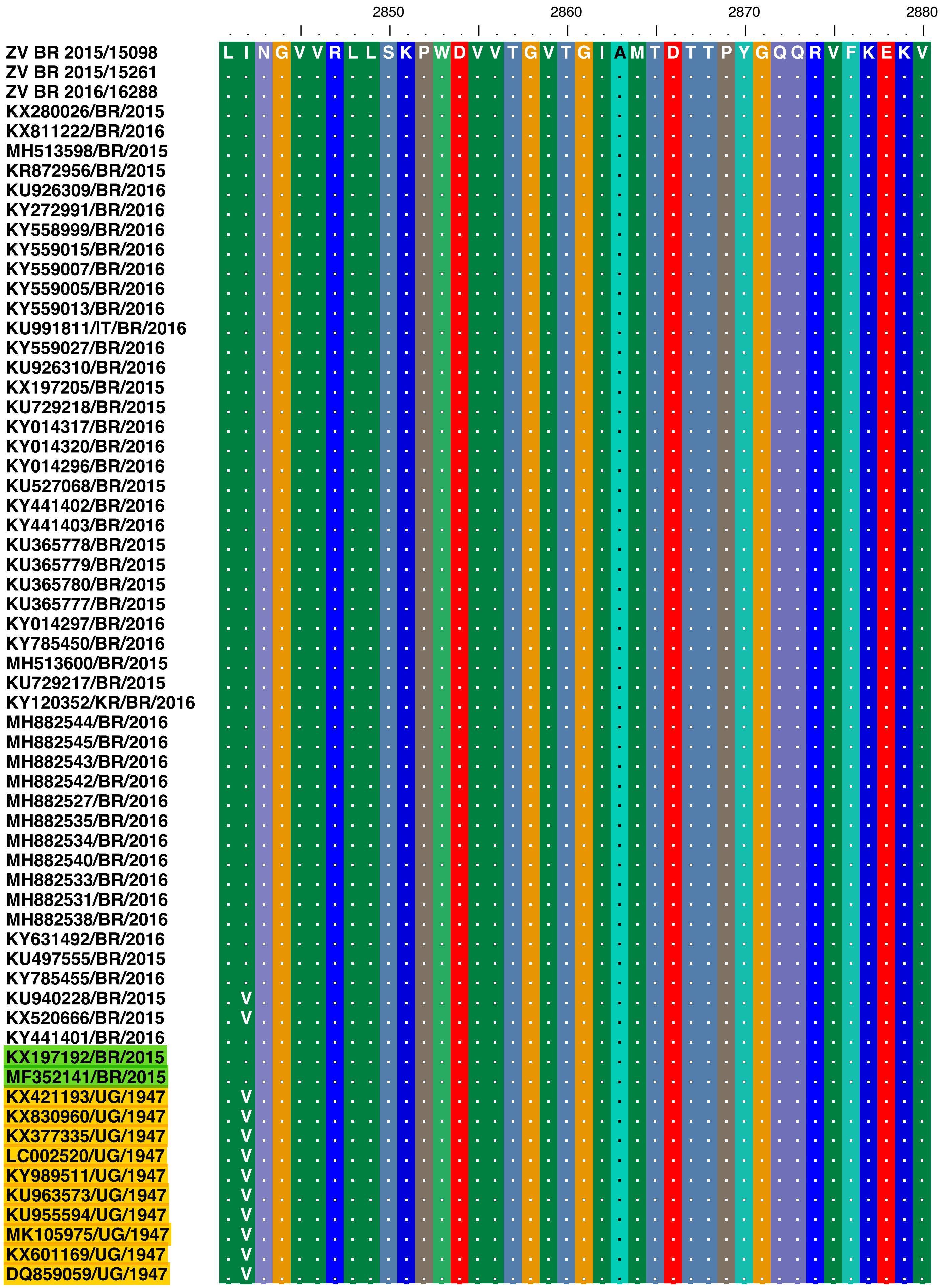
2850
2860
2870
2880
L I N G V V R L L S K P W D V V T G V T G I A M T D T T P Y G Q Q R V F K E K V
ZV BR 2015/15098
ZV BR 2015/15261
ZV BR 2016/16288
KX280026/BR/2015
KX811222/BR/2016
MH513598/BR/2015
KR872956/BR/2015
KU926309/BR/2016
KY272991/BR/2016
KY558999/BR/2016
KY559015/BR/2016
KY559007/BR/2016
KY559005/BR/2016
KY559013/BR/2016
KU991811/IT/BR/2016
KY559027/BR/2016
KU926310/BR/2016
KX197205/BR/2015
KU729218/BR/2015
KY014317/BR/2016
KY014320/BR/2016
KY014296/BR/2016
KU527068/BR/2015
KY441402/BR/2016
KY441403/BR/2016
KU365778/BR/2015
KU365779/BR/2015
KU365780/BR/2015
KU365777/BR/2015
KY014297/BR/2016
KY785450/BR/2016
MH513600/BR/2015
KU729217/BR/2015
KY120352/KR/BR/2016
MH882544/BR/2016
MH882545/BR/2016
MH882543/BR/2016
MH882542/BR/2016
MH882527/BR/2016
MH882535/BR/2016
MH882534/BR/2016
MH882540/BR/2016
MH882533/BR/2016
MH882531/BR/2016
MH882538/BR/2016
KY631492/BR/2016
KU497555/BR/2015
KY785455/BR/2016
KU940228/BR/2015
KX520666/BR/2015
KY441401/BR/2016
KX197192/BR/2015
MF352141/BR/2015
KX421193/UG/1947
KX830960/UG/1947
KX377335/UG/1947
LC002520/UG/1947
KY989511/UG/1947
KU963573/UG/1947
KU955594/UG/1947
MK105975/UG/1947
KX601169/UG/1947
DQ859059/UG/1947

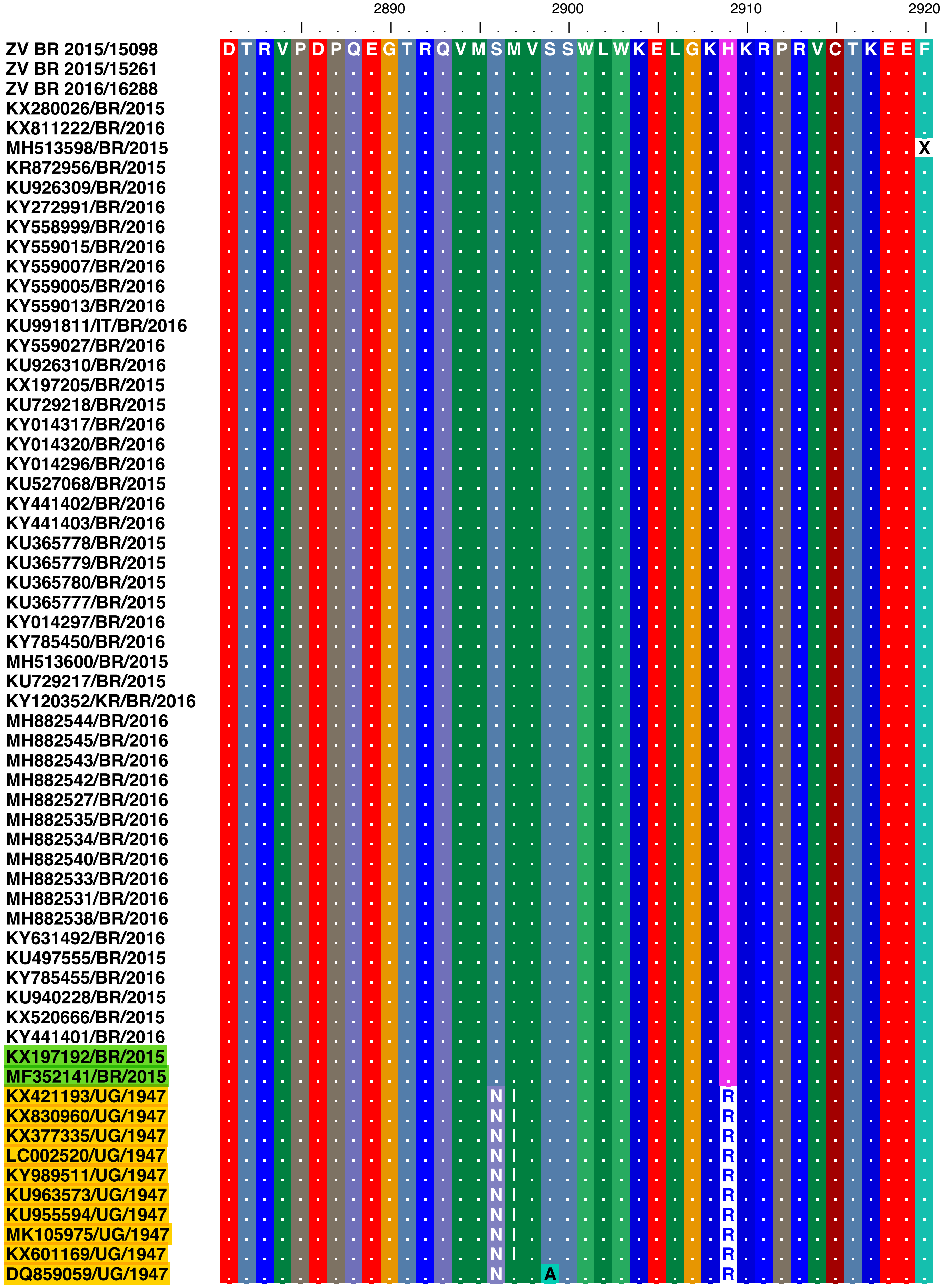

2890
2900
2910
2920
D T R V P D P Q E G T R Q V M S M V S S W L W K E L G K H K R P R V C T K E E F
ZV BR 2015/15098
ZV BR 2015/15261
ZV BR 2016/16288
KX280026/BR/2015
KX811222/BR/2016
MH513598/BR/2015
X
KR872956/BR/2015
KU926309/BR/2016
KY272991/BR/2016
KY558999/BR/2016
KY559015/BR/2016
KY559007/BR/2016
KY559005/BR/2016
KY559013/BR/2016
KU991811/IT/BR/2016
KY559027/BR/2016
KU926310/BR/2016
KX197205/BR/2015
KU729218/BR/2015
KY014317/BR/2016
KY014320/BR/2016
KY014296/BR/2016
KU527068/BR/2015
KY441402/BR/2016
KY441403/BR/2016
KU365778/BR/2015
KU365779/BR/2015
KU365780/BR/2015
KU365777/BR/2015
KY014297/BR/2016
KY785450/BR/2016
MH513600/BR/2015
KU729217/BR/2015
KY120352/KR/BR/2016
MH882544/BR/2016
MH882545/BR/2016
MH882543/BR/2016
MH882542/BR/2016
MH882527/BR/2016
MH882535/BR/2016
MH882534/BR/2016
MH882540/BR/2016
MH882533/BR/2016
MH882531/BR/2016
MH882538/BR/2016
KY631492/BR/2016
KU497555/BR/2015
KY785455/BR/2016
KU940228/BR/2015
KX520666/BR/2015
KY441401/BR/2016
KX197192/BR/2015
MF352141/BR/2015
KX421193/UG/1947 N I R
KX830960/UG/1947 N I R
KX377335/UG/1947 N I R
LC002520/UG/1947 N I R
KY989511/UG/1947 N I R
KU963573/UG/1947 N I R
KU955594/UG/1947 N I R
MK105975/UG/1947 N I R
KX601169/UG/1947 N I R
DQ859059/UG/1947 N A R

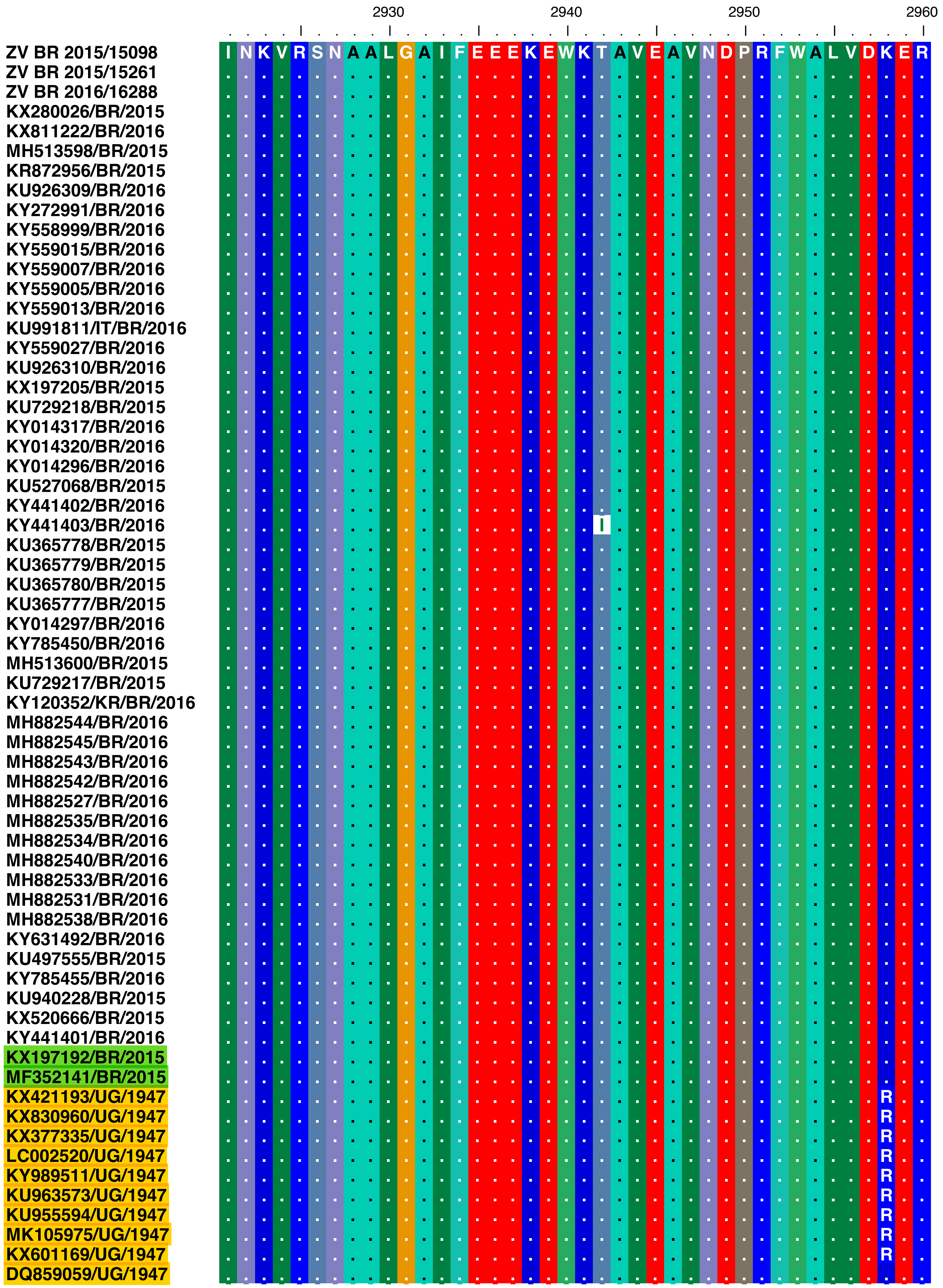
2930
2940
2950
2960
I N K V R S N A A L G A I F E E E K E W K T A V E A V N D P R F W A L V D K E R
ZV BR 2015/15098
ZV BR 2015/15261
ZV BR 2016/16288
KX280026/BR/2015
KX811222/BR/2016
MH513598/BR/2015
KR872956/BR/2015
KU926309/BR/2016
KY272991/BR/2016
KY558999/BR/2016
KY559015/BR/2016
KY559007/BR/2016
KY559005/BR/2016
KY559013/BR/2016
KU991811/IT/BR/2016
KY559027/BR/2016
KU926310/BR/2016
KX197205/BR/2015
KU729218/BR/2015
KY014317/BR/2016
KY014320/BR/2016
KY014296/BR/2016
KU527068/BR/2015
KY441402/BR/2016
KY441403/BR/2016
I
KU365778/BR/2015
KU365779/BR/2015
KU365780/BR/2015
KU365777/BR/2015
KY014297/BR/2016
KY785450/BR/2016
MH513600/BR/2015
KU729217/BR/2015
KY120352/KR/BR/2016
MH882544/BR/2016
MH882545/BR/2016
MH882543/BR/2016
MH882542/BR/2016
MH882527/BR/2016
MH882535/BR/2016
MH882534/BR/2016
MH882540/BR/2016
MH882533/BR/2016
MH882531/BR/2016
MH882538/BR/2016
KY631492/BR/2016
KU497555/BR/2015
KY785455/BR/2016
KU940228/BR/2015
KX520666/BR/2015
KY441401/BR/2016
KX197192/BR/2015
MF352141/BR/2015
KX421193/UG/1947
R
KX830960/UG/1947
R
KX377335/UG/1947
R
LC002520/UG/1947
R
KY989511/UG/1947
R
KU963573/UG/1947
R
KU955594/UG/1947
R
MK105975/UG/1947
R
KX601169/UG/1947
R
DQ859059/UG/1947

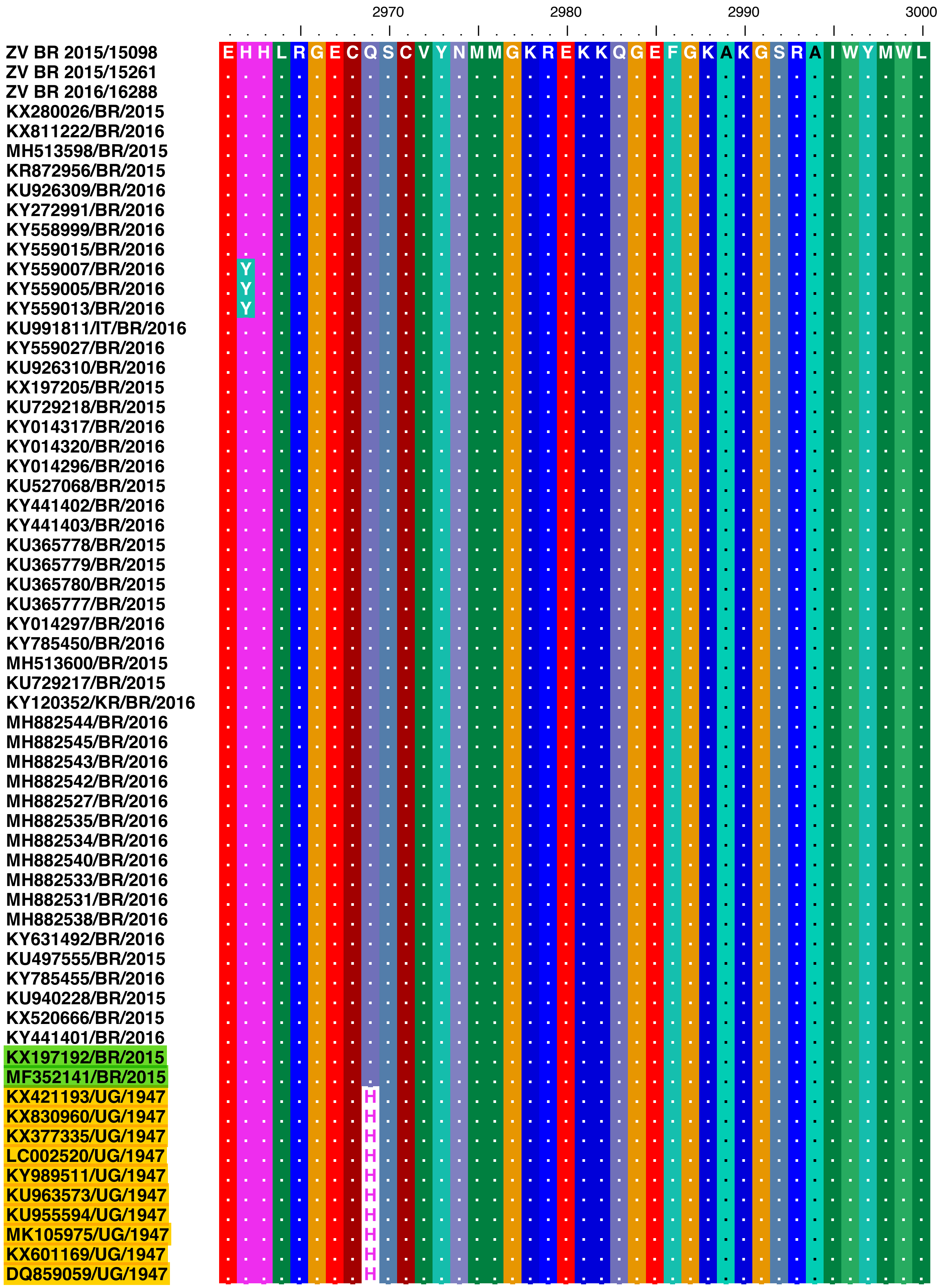
2970
2980
2990
3000
E H H L R G E C Q S C V Y N M M G K R E K K Q G E F G K A K G S R A I W Y M W L
ZV BR 2015/15098
ZV BR 2015/15261
ZV BR 2016/16288
KX280026/BR/2015
KX811222/BR/2016
MH513598/BR/2015
KR872956/BR/2015
KU926309/BR/2016
KY272991/BR/2016
KY558999/BR/2016
KY559015/BR/2016
KY559007/BR/2016
KY559005/BR/2016
KY559013/BR/2016
KU991811/IT/BR/2016
KY559027/BR/2016
KU926310/BR/2016
KX197205/BR/2015
KU729218/BR/2015
KY014317/BR/2016
KY014320/BR/2016
KY014296/BR/2016
KU527068/BR/2015
KY441402/BR/2016
KY441403/BR/2016
KU365778/BR/2015
KU365779/BR/2015
KU365780/BR/2015
KU365777/BR/2015
KY014297/BR/2016
KY785450/BR/2016
MH513600/BR/2015
KU729217/BR/2015
KY120352/KR/BR/2016
MH882544/BR/2016
MH882545/BR/2016
MH882543/BR/2016
MH882542/BR/2016
MH882527/BR/2016
MH882535/BR/2016
MH882534/BR/2016
MH882540/BR/2016
MH882533/BR/2016
MH882531/BR/2016
MH882538/BR/2016
KY631492/BR/2016
KU497555/BR/2015
KY785455/BR/2016
KU940228/BR/2015
KX520666/BR/2015
KY441401/BR/2016
KX197192/BR/2015
MF352141/BR/2015
KX421193/UG/1947
KX830960/UG/1947
KX377335/UG/1947
LC002520/UG/1947
KY989511/UG/1947
KU963573/UG/1947
KU955594/UG/1947
MK105975/UG/1947
KX601169/UG/1947
DQ859059/UG/1947
Y
Y
Y
H
H
H
H
H
H
H
H
H
H

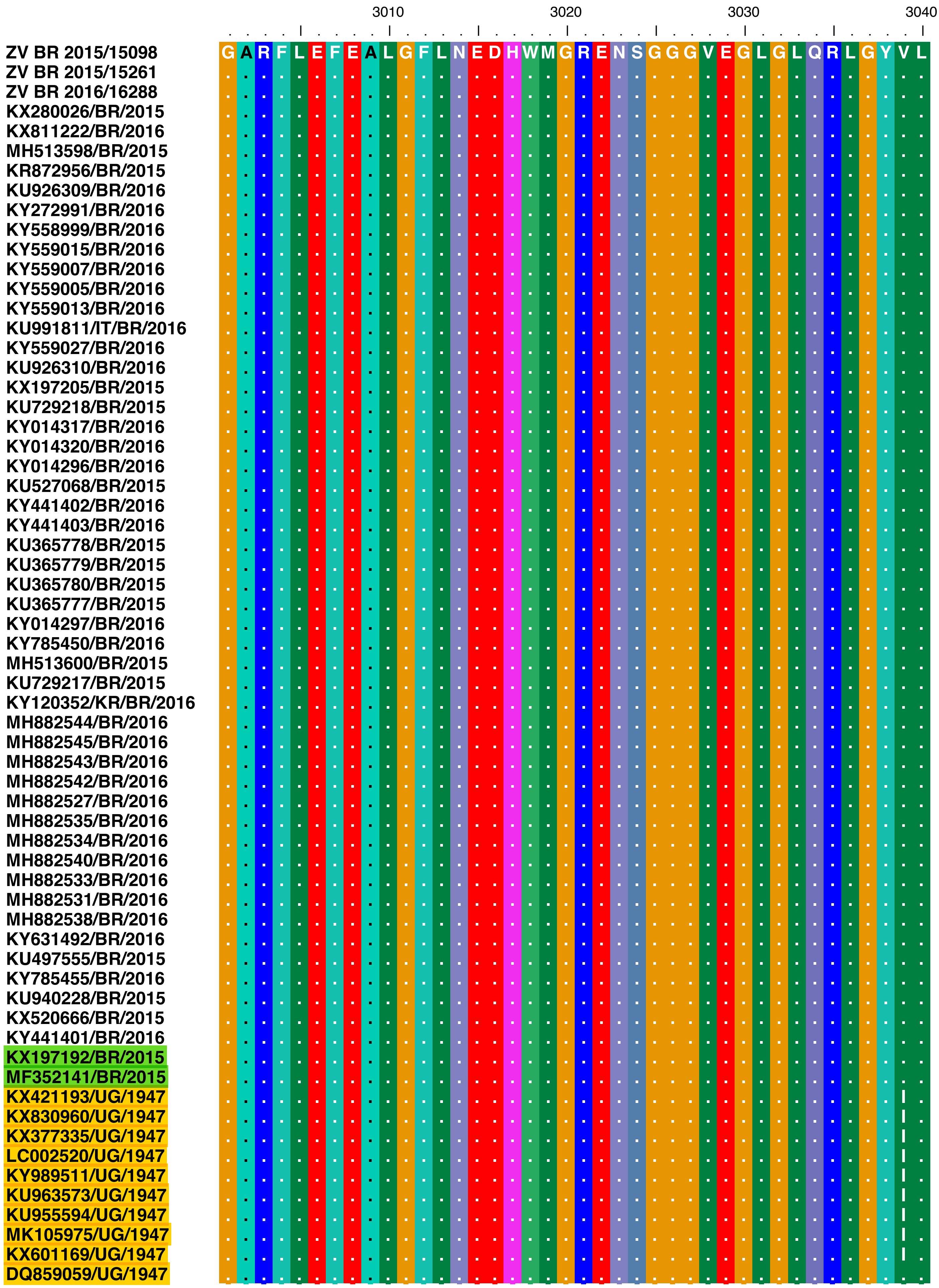
3010
3020
3030
3040
G A R F L E F E A L G F L N E D H W M G R E N S G G G V E G L G L Q R L G Y V L
ZV BR 2015/15098
ZV BR 2015/15261
ZV BR 2016/16288
KX280026/BR/2015
KX811222/BR/2016
MH513598/BR/2015
KR872956/BR/2015
KU926309/BR/2016
KY272991/BR/2016
KY558999/BR/2016
KY559015/BR/2016
KY559007/BR/2016
KY559005/BR/2016
KY559013/BR/2016
KU991811/IT/BR/2016
KY559027/BR/2016
KU926310/BR/2016
KX197205/BR/2015
KU729218/BR/2015
KY014317/BR/2016
KY014320/BR/2016
KY014296/BR/2016
KU527068/BR/2015
KY441402/BR/2016
KY441403/BR/2016
KU365778/BR/2015
KU365779/BR/2015
KU365780/BR/2015
KU365777/BR/2015
KY014297/BR/2016
KY785450/BR/2016
MH513600/BR/2015
KU729217/BR/2015
KY120352/KR/BR/2016
MH882544/BR/2016
MH882545/BR/2016
MH882543/BR/2016
MH882542/BR/2016
MH882527/BR/2016
MH882535/BR/2016
MH882534/BR/2016
MH882540/BR/2016
MH882533/BR/2016
MH882531/BR/2016
MH882538/BR/2016
KY631492/BR/2016
KU497555/BR/2015
KY785455/BR/2016
KU940228/BR/2015
KX520666/BR/2015
KY441401/BR/2016
KX197192/BR/2015
MF352141/BR/2015
KX421193/UG/1947
KX830960/UG/1947
KX377335/UG/1947
LC002520/UG/1947
KY989511/UG/1947
KU963573/UG/1947
KU955594/UG/1947
MK105975/UG/1947
KX601169/UG/1947
DQ859059/UG/1947

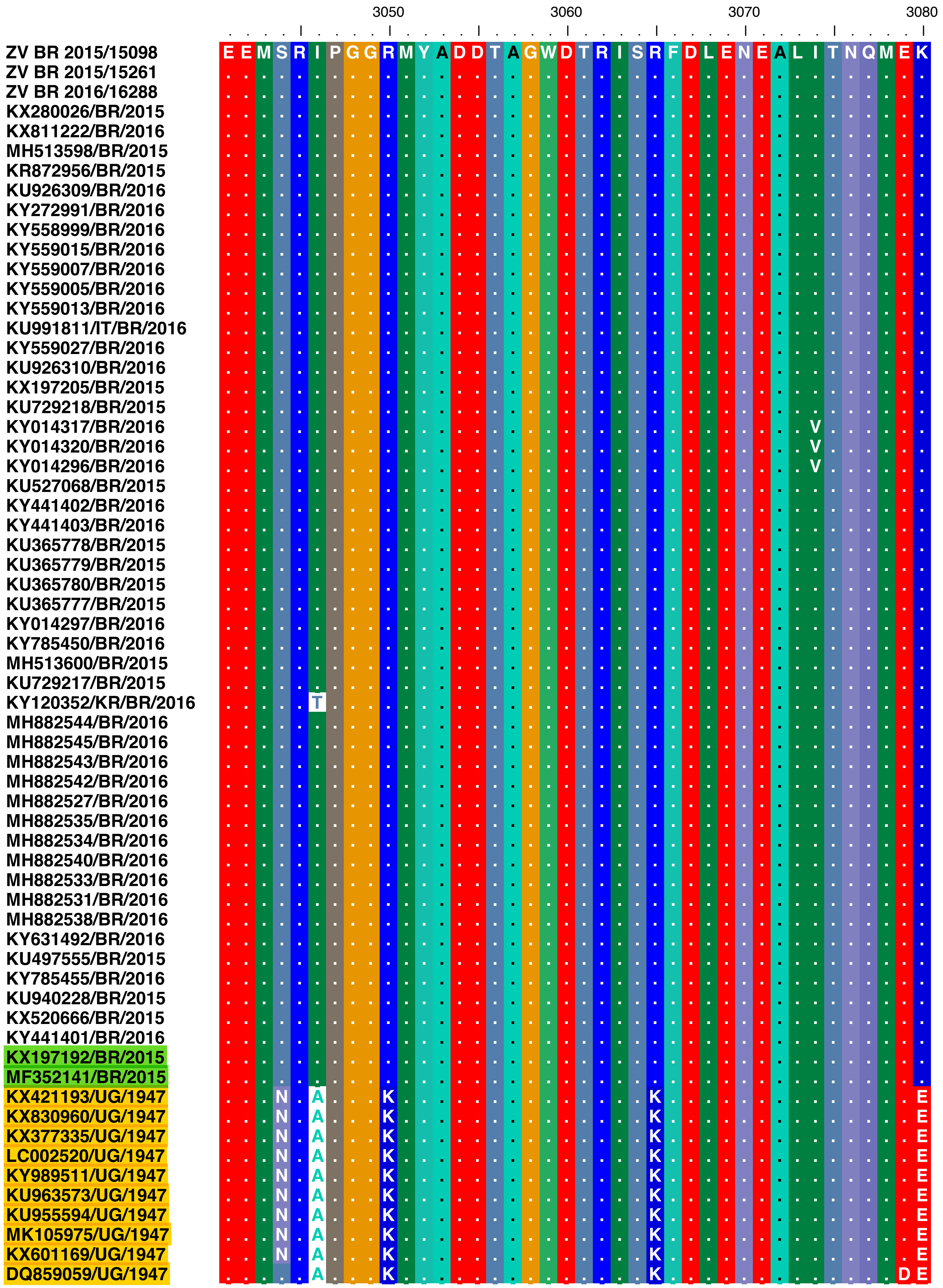
3050
3060
3070
3080
E E M S R I P G G R M Y A D D T A G W D T R I S R F D L E N E A L I T N Q M E K
ZV BR 2015/15098
ZV BR 2015/15261
ZV BR 2016/16288
KX280026/BR/2015
KX811222/BR/2016
MH513598/BR/2015
KR872956/BR/2015
KU926309/BR/2016
KY272991/BR/2016
KY558999/BR/2016
KY559015/BR/2016
KY559007/BR/2016
KY559005/BR/2016
KY559013/BR/2016
KU991811/IT/BR/2016
KY559027/BR/2016
KU926310/BR/2016
KX197205/BR/2015
KU729218/BR/2015
KY014317/BR/2016
KY014320/BR/2016
KY014296/BR/2016
KU527068/BR/2015
KY441402/BR/2016
KY441403/BR/2016
KU365778/BR/2015
KU365779/BR/2015
KU365780/BR/2015
KU365777/BR/2015
KY014297/BR/2016
KY785450/BR/2016
MH513600/BR/2015
KU729217/BR/2015
KY120352/KR/BR/2016
MH882544/BR/2016
MH882545/BR/2016
MH882543/BR/2016
MH882542/BR/2016
MH882527/BR/2016
MH882535/BR/2016
MH882534/BR/2016
MH882540/BR/2016
MH882533/BR/2016
MH882531/BR/2016
MH882538/BR/2016
KY631492/BR/2016
KU497555/BR/2015
KY785455/BR/2016
KU940228/BR/2015
KX520666/BR/2015
KY441401/BR/2016
KX197192/BR/2015
MF352141/BR/2015
KX421193/UG/1947
KX830960/UG/1947
KX377335/UG/1947
LC002520/UG/1947
KY989511/UG/1947
KU963573/UG/1947
KU955594/UG/1947
MK105975/UG/1947
KX601169/UG/1947
DQ859059/UG/1947

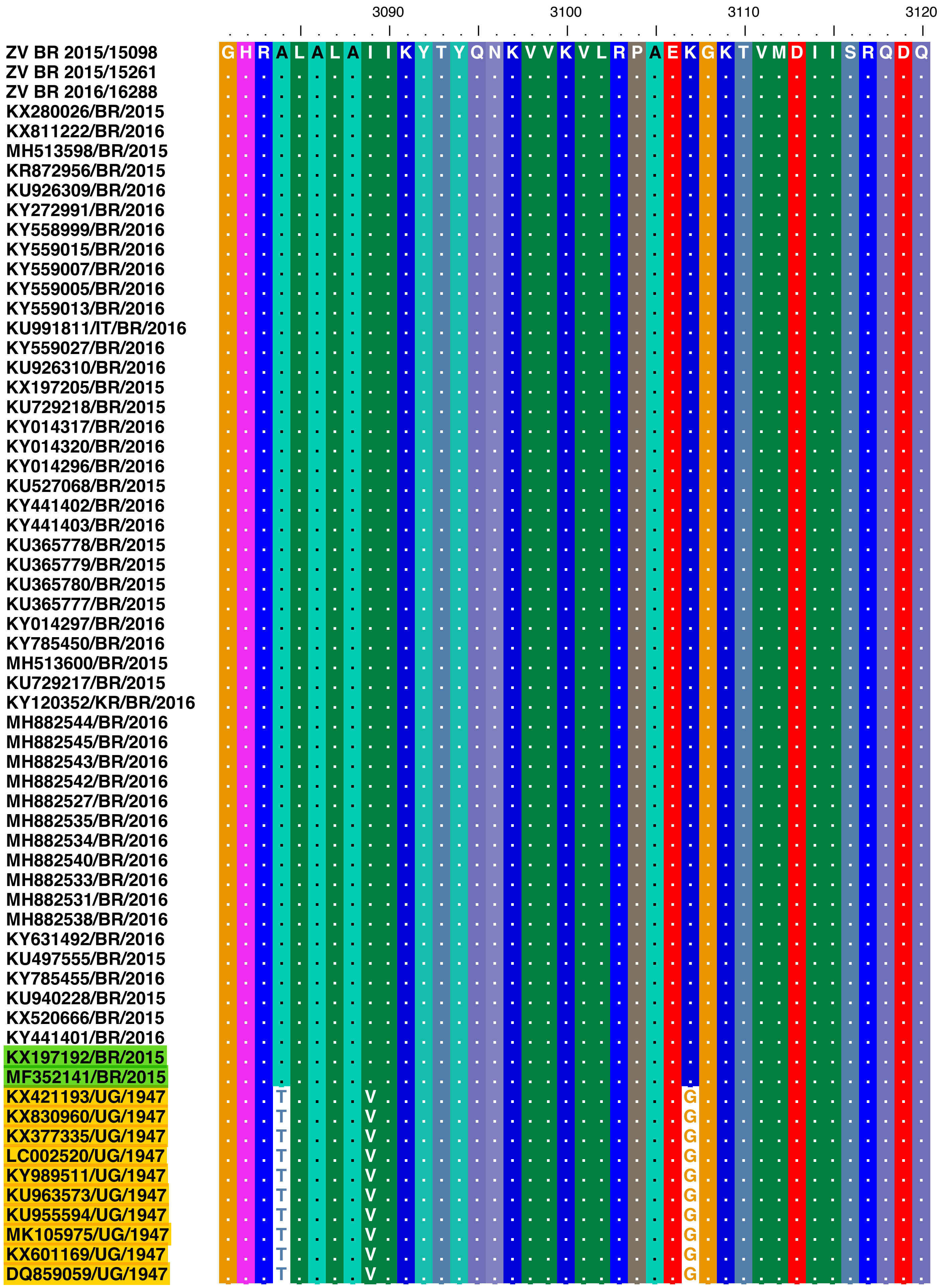

3090
3100
3110
3120
G H R A L A L A I I K Y T Y Q N K V V K V L R P A E K G K T V M D I I S R Q D Q
ZV BR 2015/15098
ZV BR 2015/15261
ZV BR 2016/16288
KX280026/BR/2015
KX811222/BR/2016
MH513598/BR/2015
KR872956/BR/2015
KU926309/BR/2016
KY272991/BR/2016
KY558999/BR/2016
KY559015/BR/2016
KY559007/BR/2016
KY559005/BR/2016
KY559013/BR/2016
KU991811/IT/BR/2016
KY559027/BR/2016
KU926310/BR/2016
KX197205/BR/2015
KU729218/BR/2015
KY014317/BR/2016
KY014320/BR/2016
KY014296/BR/2016
KU527068/BR/2015
KY441402/BR/2016
KY441403/BR/2016
KU365778/BR/2015
KU365779/BR/2015
KU365780/BR/2015
KU365777/BR/2015
KY014297/BR/2016
KY785450/BR/2016
MH513600/BR/2015
KU729217/BR/2015
KY120352/KR/BR/2016
MH882544/BR/2016
MH882545/BR/2016
MH882543/BR/2016
MH882542/BR/2016
MH882527/BR/2016
MH882535/BR/2016
MH882534/BR/2016
MH882540/BR/2016
MH882533/BR/2016
MH882531/BR/2016
MH882538/BR/2016
KY631492/BR/2016
KU497555/BR/2015
KY785455/BR/2016
KU940228/BR/2015
KX520666/BR/2015
KY441401/BR/2016
KX197192/BR/2015
MF352141/BR/2015
KX421193/UG/1947 T V G
KX830960/UG/1947 T V G
KX377335/UG/1947 T V G
LC002520/UG/1947 T V G
KY989511/UG/1947 T V G
KU963573/UG/1947 T V G
KU955594/UG/1947 T V G
MK105975/UG/1947 T V G
KX601169/UG/1947 T V G
DQ859059/UG/1947 T V G

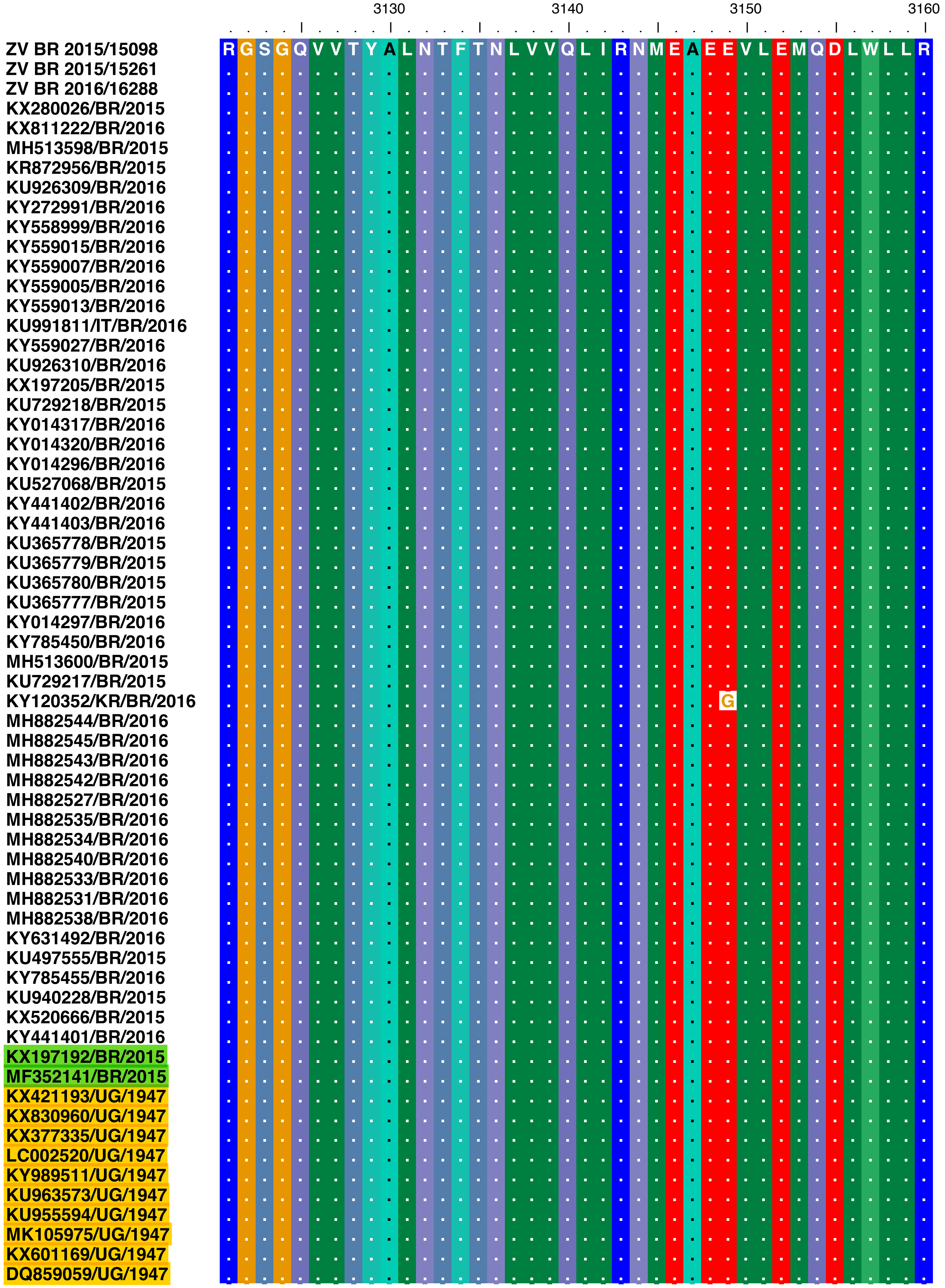

3130
3140
3150
3160
R G S G Q V V T Y A L N T F T N L V V Q L I R N M E A E E V L E M Q D L W L L R
ZV BR 2015/15098
ZV BR 2015/15261
ZV BR 2016/16288
KX280026/BR/2015
KX811222/BR/2016
MH513598/BR/2015
KR872956/BR/2015
KU926309/BR/2016
KY272991/BR/2016
KY558999/BR/2016
KY559015/BR/2016
KY559007/BR/2016
KY559005/BR/2016
KY559013/BR/2016
KU991811/IT/BR/2016
KY559027/BR/2016
KU926310/BR/2016
KX197205/BR/2015
KU729218/BR/2015
KY014317/BR/2016
KY014320/BR/2016
KY014296/BR/2016
KU527068/BR/2015
KY441402/BR/2016
KY441403/BR/2016
KU365778/BR/2015
KU365779/BR/2015
KU365780/BR/2015
KU365777/BR/2015
KY014297/BR/2016
KY785450/BR/2016
MH513600/BR/2015
KU729217/BR/2015
KY120352/KR/BR/2016
G
MH882544/BR/2016
MH882545/BR/2016
MH882543/BR/2016
MH882542/BR/2016
MH882527/BR/2016
MH882535/BR/2016
MH882534/BR/2016
MH882540/BR/2016
MH882533/BR/2016
MH882531/BR/2016
MH882538/BR/2016
KY631492/BR/2016
KU497555/BR/2015
KY785455/BR/2016
KU940228/BR/2015
KX520666/BR/2015
KY441401/BR/2016
KX197192/BR/2015
MF352141/BR/2015
KX421193/UG/1947
KX830960/UG/1947
KX377335/UG/1947
LC002520/UG/1947
KY989511/UG/1947
KU963573/UG/1947
KU955594/UG/1947
MK105975/UG/1947
KX601169/UG/1947
DQ859059/UG/1947

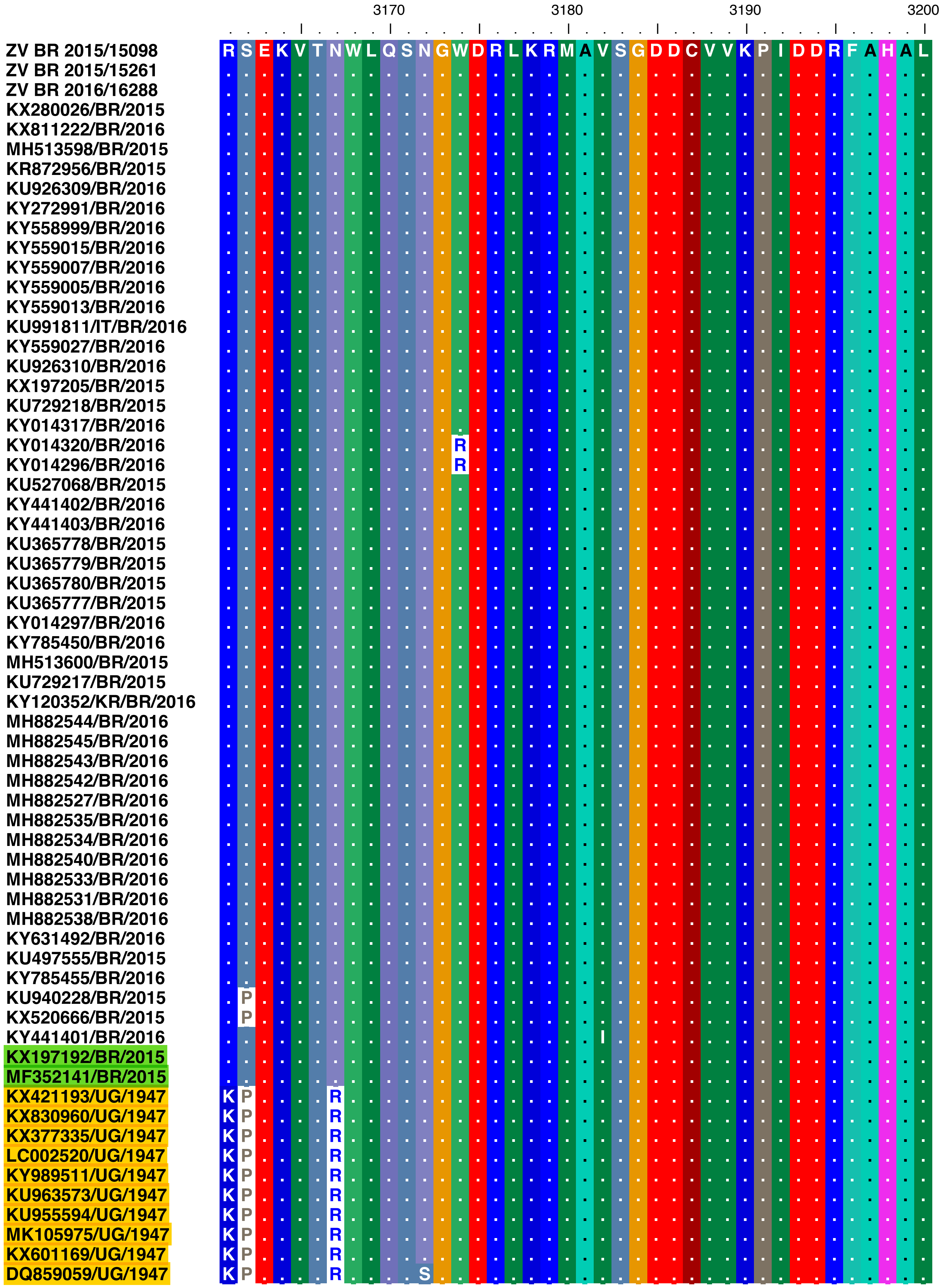
3170
3180
3190
3200
RSEKVTNWLQSNGWDRLKRMAVSGDDCVVKPIDDRFAHAL
ZV BR 2015/15098
ZV BR 2015/15261
ZV BR 2016/16288
KX280026/BR/2015
KX811222/BR/2016
MH513598/BR/2015
KR872956/BR/2015
KU926309/BR/2016
KY272991/BR/2016
KY558999/BR/2016
KY559015/BR/2016
KY559007/BR/2016
KY559005/BR/2016
KY559013/BR/2016
KU991811/IT/BR/2016
KY559027/BR/2016
KU926310/BR/2016
KX197205/BR/2015
KU729218/BR/2015
KY014317/BR/2016
KY014320/BR/2016
KY014296/BR/2016
KU527068/BR/2015
KY441402/BR/2016
KY441403/BR/2016
KU365778/BR/2015
KU365779/BR/2015
KU365780/BR/2015
KU365777/BR/2015
KY014297/BR/2016
KY785450/BR/2016
MH513600/BR/2015
KU729217/BR/2015
KY120352/KR/BR/2016
MH882544/BR/2016
MH882545/BR/2016
MH882543/BR/2016
MH882542/BR/2016
MH882527/BR/2016
MH882535/BR/2016
MH882534/BR/2016
MH882540/BR/2016
MH882533/BR/2016
MH882531/BR/2016
MH882538/BR/2016
KY631492/BR/2016
KU497555/BR/2015
KY785455/BR/2016
KU940228/BR/2015
KX520666/BR/2015
KY441401/BR/2016
KX197192/BR/2015
MF352141/BR/2015
KX421193/UG/1947
KX830960/UG/1947
KX377335/UG/1947
LC002520/UG/1947
KY989511/UG/1947
KU963573/UG/1947
KU955594/UG/1947
MK105975/UG/1947
KX601169/UG/1947
DQ859059/UG/1947

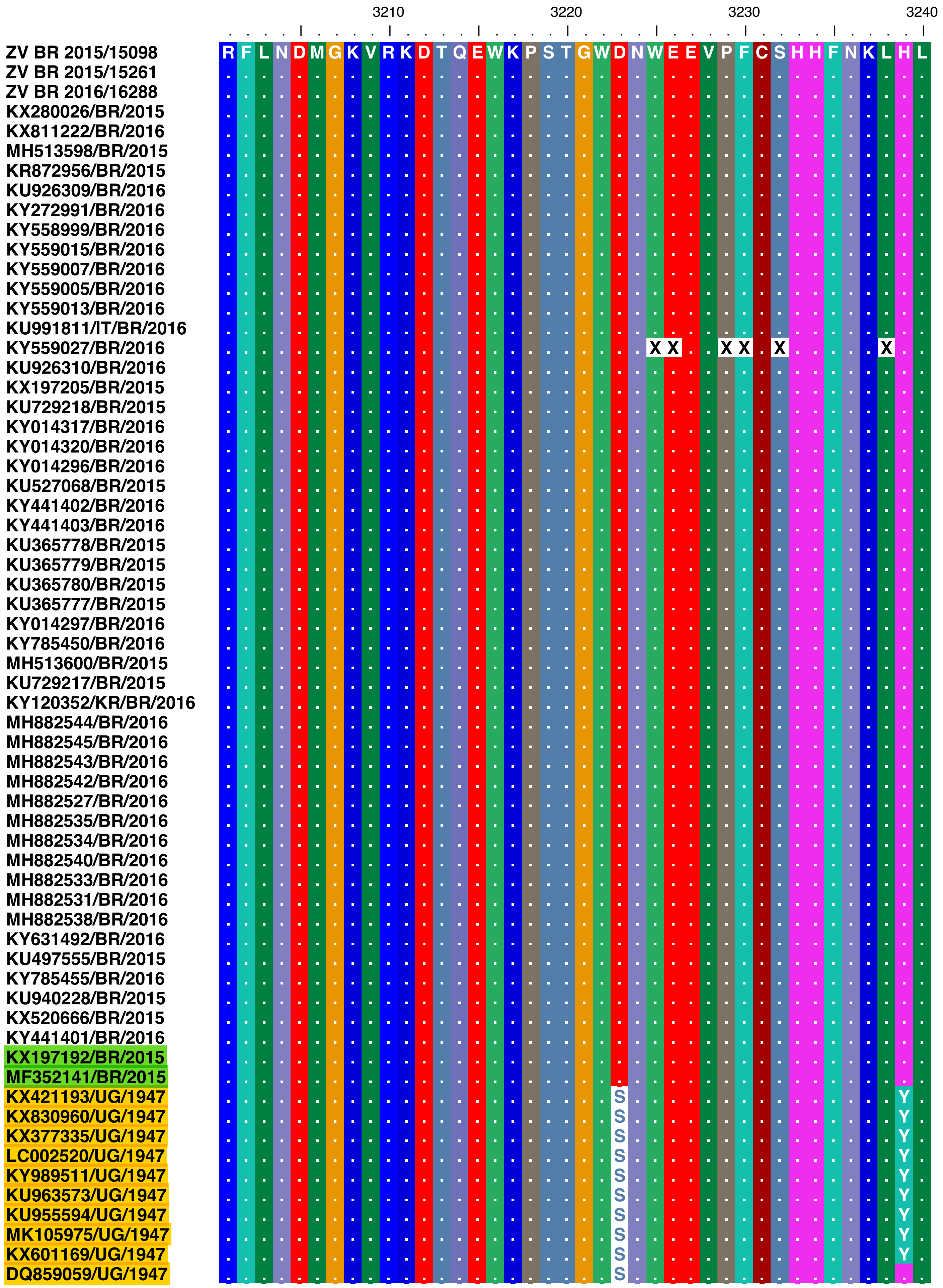
3210
3220
3230
3240
R F L N D M G K V R K D T Q E W K P S T G W D N W E E V P F C S H H F N K L H L
ZV BR 2015/15098
ZV BR 2015/15261
ZV BR 2016/16288
KX280026/BR/2015
KX811222/BR/2016
MH513598/BR/2015
KR872956/BR/2015
KU926309/BR/2016
KY272991/BR/2016
KY558999/BR/2016
KY559015/BR/2016
KY559007/BR/2016
KY559005/BR/2016
KY559013/BR/2016
KU991811/IT/BR/2016
KY559027/BR/2016
KU926310/BR/2016
KX197205/BR/2015
KU729218/BR/2015
KY014317/BR/2016
KY014320/BR/2016
KY014296/BR/2016
KU527068/BR/2015
KY441402/BR/2016
KY441403/BR/2016
KU365778/BR/2015
KU365779/BR/2015
KU365780/BR/2015
KU365777/BR/2015
KY014297/BR/2016
KY785450/BR/2016
MH513600/BR/2015
KU729217/BR/2015
KY120352/KR/BR/2016
MH882544/BR/2016
MH882545/BR/2016
MH882543/BR/2016
MH882542/BR/2016
MH882527/BR/2016
MH882535/BR/2016
MH882534/BR/2016
MH882540/BR/2016
MH882533/BR/2016
MH882531/BR/2016
MH882538/BR/2016
KY631492/BR/2016
KU497555/BR/2015
KY785455/BR/2016
KU940228/BR/2015
KX520666/BR/2015
KY441401/BR/2016
KX197192/BR/2015
MF352141/BR/2015
KX421193/UG/1947
KX830960/UG/1947
KX377335/UG/1947
LC002520/UG/1947
KY989511/UG/1947
KU963573/UG/1947
KU955594/UG/1947
MK105975/UG/1947
KX601169/UG/1947
DQ859059/UG/1947
X X X X X X
S
Y

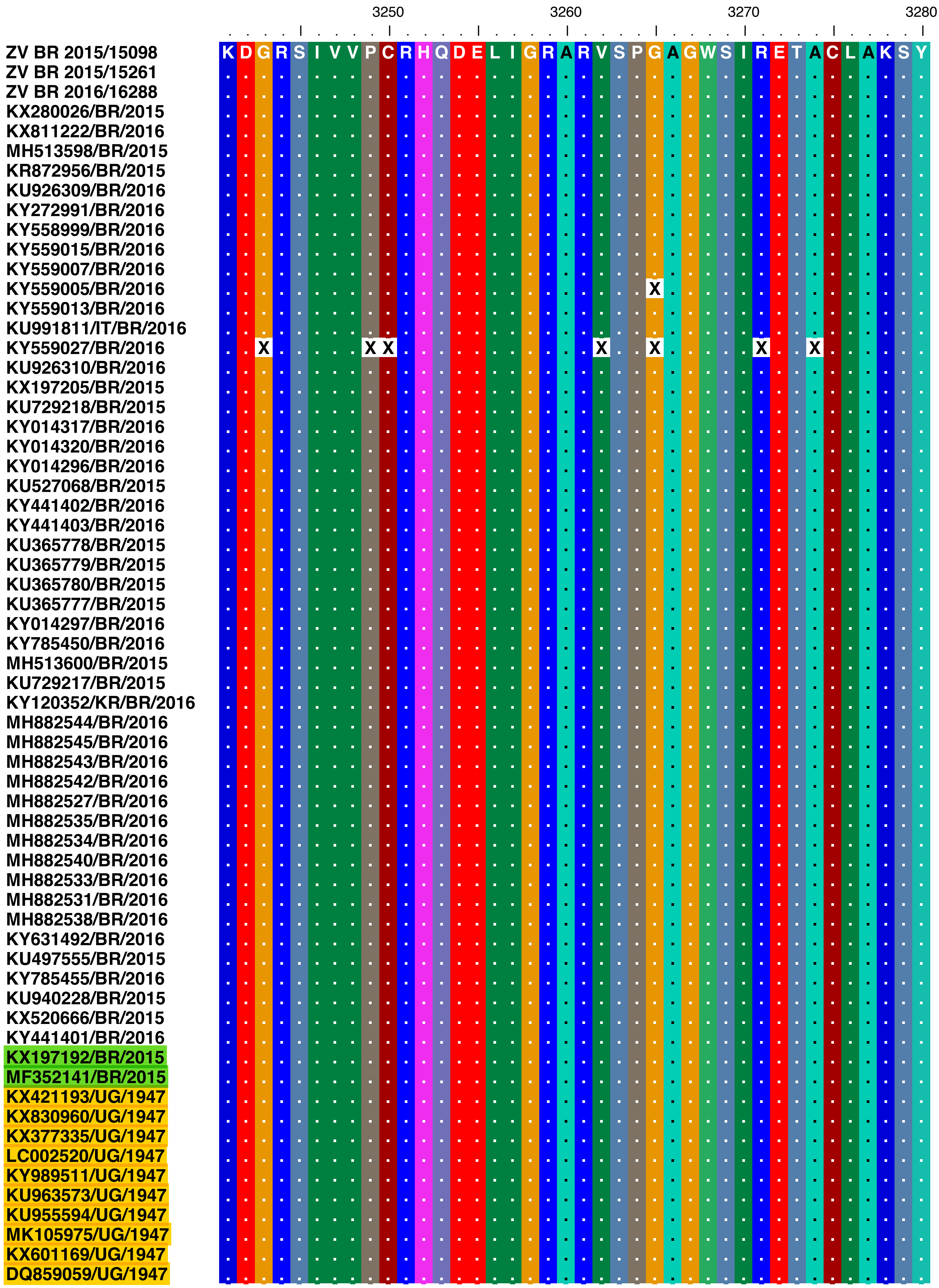
3250
3260
3270
3280
K D G R S I V V P C R H Q D E L I G R A R V S P G A G W S I R E T A C L A K S Y
ZV BR 2015/15098
ZV BR 2015/15261
ZV BR 2016/16288
KX280026/BR/2015
KX811222/BR/2016
MH513598/BR/2015
KR872956/BR/2015
KU926309/BR/2016
KY272991/BR/2016
KY558999/BR/2016
KY559015/BR/2016
KY559007/BR/2016
KY559005/BR/2016
KY559013/BR/2016
KU991811/IT/BR/2016
KY559027/BR/2016
KU926310/BR/2016
KX197205/BR/2015
KU729218/BR/2015
KY014317/BR/2016
KY014320/BR/2016
KY014296/BR/2016
KU527068/BR/2015
KY441402/BR/2016
KY441403/BR/2016
KU365778/BR/2015
KU365779/BR/2015
KU365780/BR/2015
KU365777/BR/2015
KY014297/BR/2016
KY785450/BR/2016
MH513600/BR/2015
KU729217/BR/2015
KY120352/KR/BR/2016
MH882544/BR/2016
MH882545/BR/2016
MH882543/BR/2016
MH882542/BR/2016
MH882527/BR/2016
MH882535/BR/2016
MH882534/BR/2016
MH882540/BR/2016
MH882533/BR/2016
MH882531/BR/2016
MH882538/BR/2016
KY631492/BR/2016
KU497555/BR/2015
KY785455/BR/2016
KU940228/BR/2015
KX520666/BR/2015
KY441401/BR/2016
KX197192/BR/2015
MF352141/BR/2015
KX421193/UG/1947
KX830960/UG/1947
KX377335/UG/1947
LC002520/UG/1947
KY989511/UG/1947
KU963573/UG/1947
KU955594/UG/1947
MK105975/UG/1947
KX601169/UG/1947
DQ859059/UG/1947
X

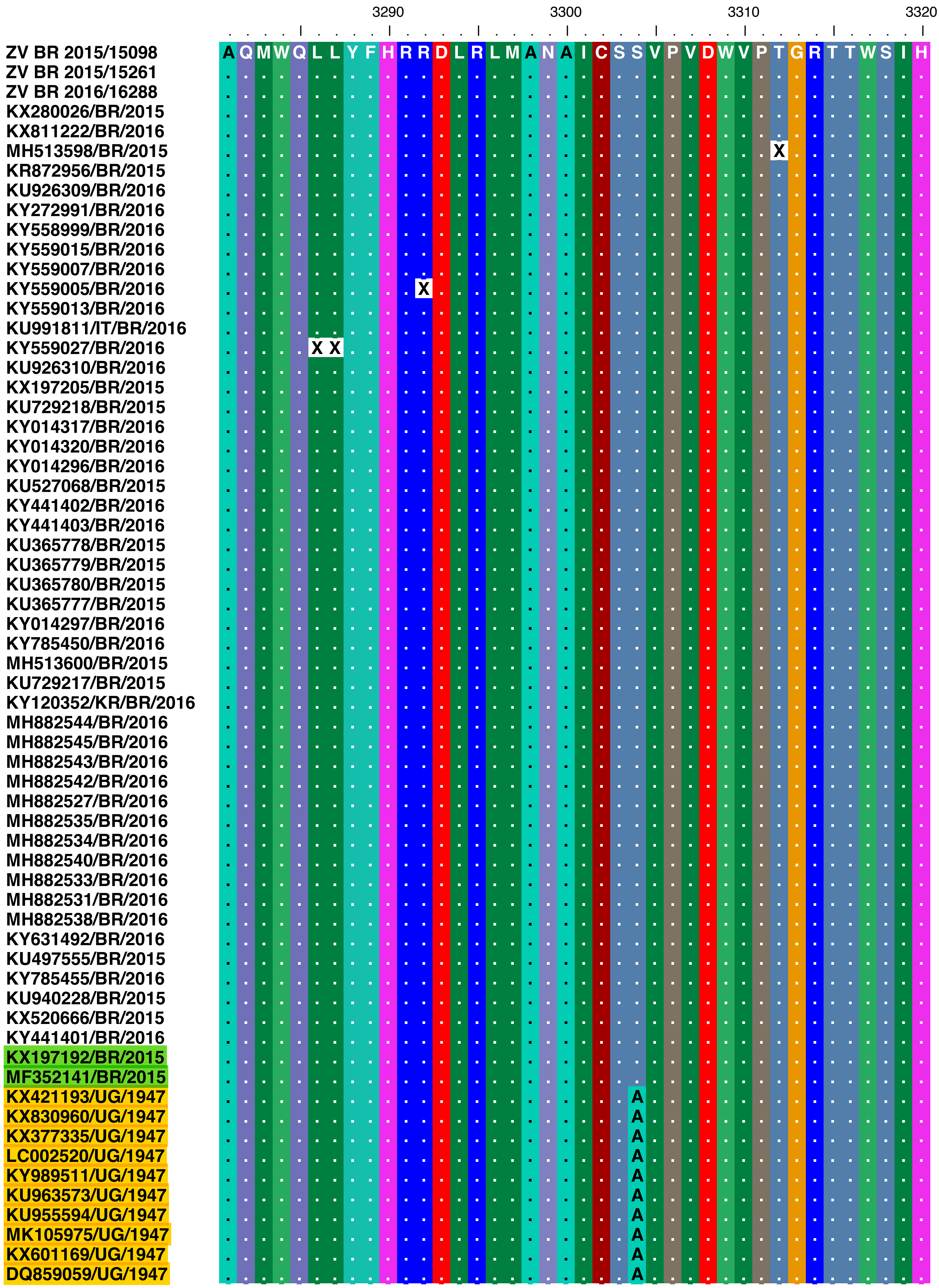
3290
3300
3310
3320
A Q M W Q L L Y F H R R D L R L M A N A I C S S V P V D W V P T G R T T W S I H
ZV BR 2015/15098
ZV BR 2015/15261
ZV BR 2016/16288
KX280026/BR/2015
KX811222/BR/2016
MH513598/BR/2015
KR872956/BR/2015
KU926309/BR/2016
KY272991/BR/2016
KY558999/BR/2016
KY559015/BR/2016
KY559007/BR/2016
KY559005/BR/2016
KY559013/BR/2016
KU991811/IT/BR/2016
KY559027/BR/2016
KU926310/BR/2016
KX197205/BR/2015
KU729218/BR/2015
KY014317/BR/2016
KY014320/BR/2016
KY014296/BR/2016
KU527068/BR/2015
KY441402/BR/2016
KY441403/BR/2016
KU365778/BR/2015
KU365779/BR/2015
KU365780/BR/2015
KU365777/BR/2015
KY014297/BR/2016
KY785450/BR/2016
MH513600/BR/2015
KU729217/BR/2015
KY120352/KR/BR/2016
MH882544/BR/2016
MH882545/BR/2016
MH882543/BR/2016
MH882542/BR/2016
MH882527/BR/2016
MH882535/BR/2016
MH882534/BR/2016
MH882540/BR/2016
MH882533/BR/2016
MH882531/BR/2016
MH882538/BR/2016
KY631492/BR/2016
KU497555/BR/2015
KY785455/BR/2016
KU940228/BR/2015
KX520666/BR/2015
KY441401/BR/2016
KX197192/BR/2015
MF352141/BR/2015
KX421193/UG/1947
KX830960/UG/1947
KX377335/UG/1947
LC002520/UG/1947
KY989511/UG/1947
KU963573/UG/1947
KU955594/UG/1947
MK105975/UG/1947
KX601169/UG/1947
DQ859059/UG/1947

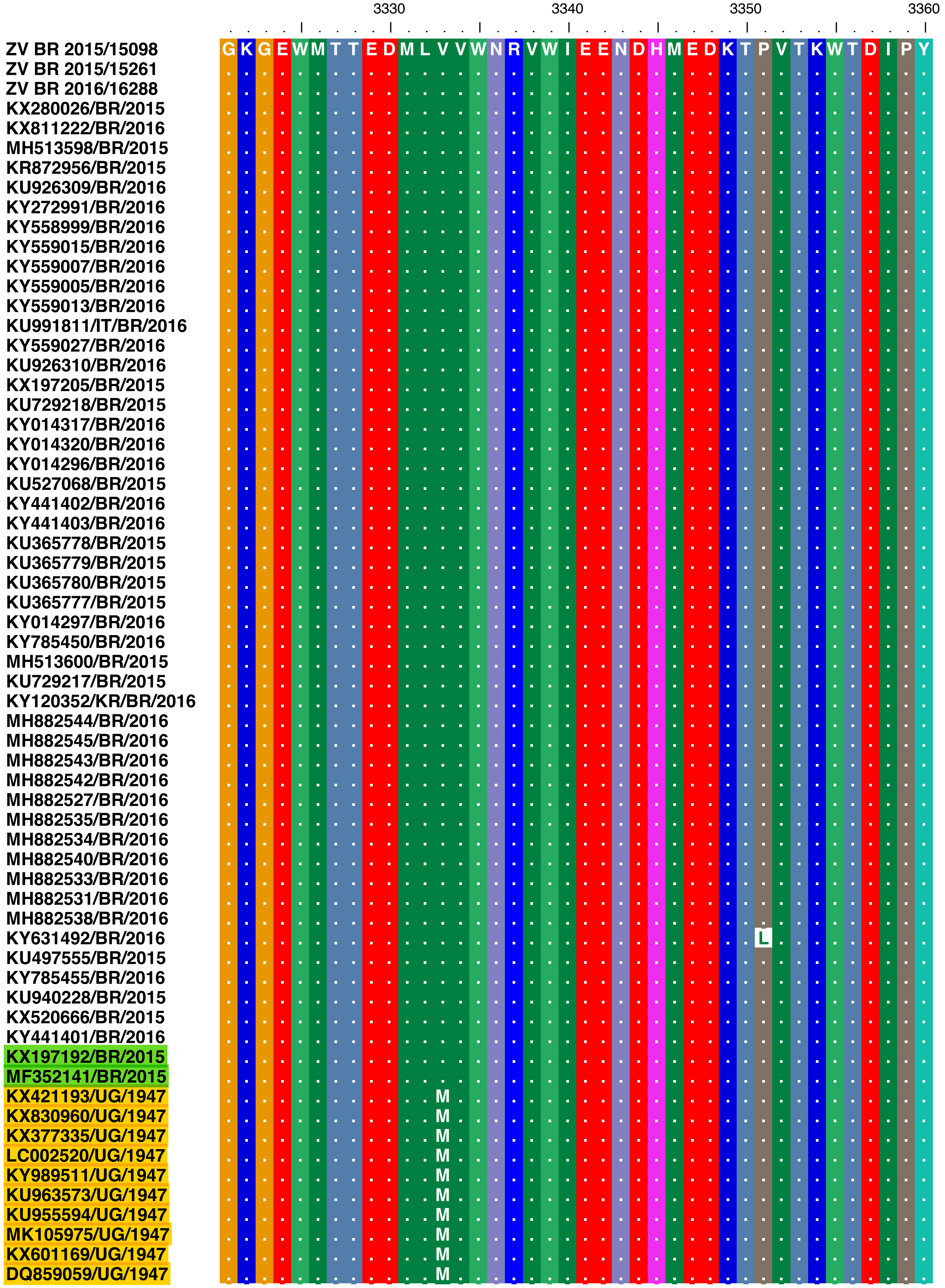
3330
3340
3350
3360
G K G E W M T T E D M L V V W N R V W I E E N D H M E D K T P V T K W T D I P Y
ZV BR 2015/15098
ZV BR 2015/15261
ZV BR 2016/16288
KX280026/BR/2015
KX811222/BR/2016
MH513598/BR/2015
KR872956/BR/2015
KU926309/BR/2016
KY272991/BR/2016
KY558999/BR/2016
KY559015/BR/2016
KY559007/BR/2016
KY559005/BR/2016
KY559013/BR/2016
KU991811/IT/BR/2016
KY559027/BR/2016
KU926310/BR/2016
KX197205/BR/2015
KU729218/BR/2015
KY014317/BR/2016
KY014320/BR/2016
KY014296/BR/2016
KU527068/BR/2015
KY441402/BR/2016
KY441403/BR/2016
KU365778/BR/2015
KU365779/BR/2015
KU365780/BR/2015
KU365777/BR/2015
KY014297/BR/2016
KY785450/BR/2016
MH513600/BR/2015
KU729217/BR/2015
KY120352/KR/BR/2016
MH882544/BR/2016
MH882545/BR/2016
MH882543/BR/2016
MH882542/BR/2016
MH882527/BR/2016
MH882535/BR/2016
MH882534/BR/2016
MH882540/BR/2016
MH882533/BR/2016
MH882531/BR/2016
MH882538/BR/2016
KY631492/BR/2016
L
KU497555/BR/2015
KY785455/BR/2016
KU940228/BR/2015
KX520666/BR/2015
KY441401/BR/2016
KX197192/BR/2015
MF352141/BR/2015
KX421193/UG/1947
M
KX830960/UG/1947
M
KX377335/UG/1947
M
LC002520/UG/1947
M
KY989511/UG/1947
M
KU963573/UG/1947
M
KU955594/UG/1947
M
MK105975/UG/1947
M
KX601169/UG/1947
M
DQ859059/UG/1947
M

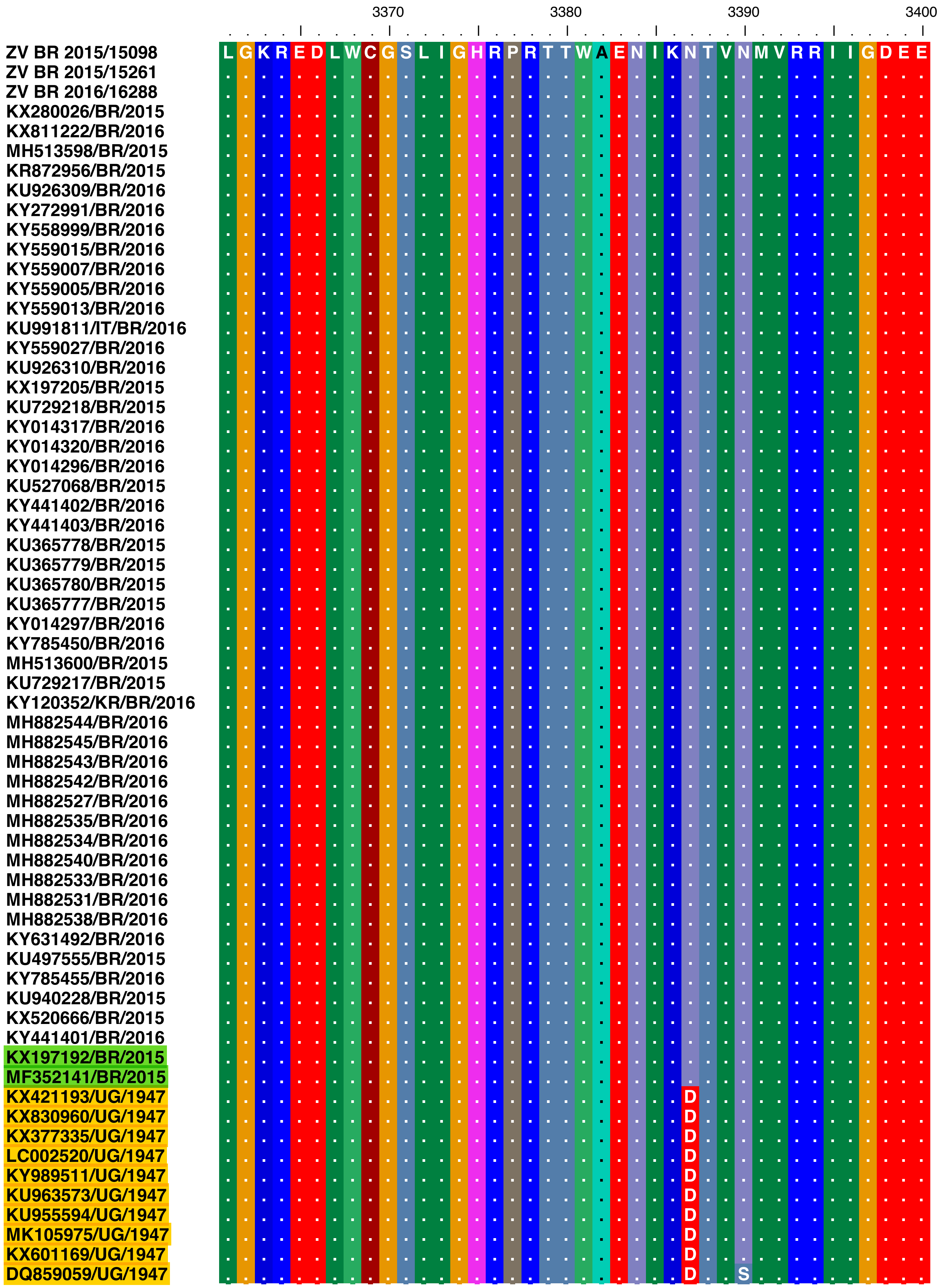
3370
3380
3390
3400
L G K R E D L W C G S L I G H R P R T T W A E N I K N T V N M V R R I I G D E E
ZV BR 2015/15098
ZV BR 2015/15261
ZV BR 2016/16288
KX280026/BR/2015
KX811222/BR/2016
MH513598/BR/2015
KR872956/BR/2015
KU926309/BR/2016
KY272991/BR/2016
KY558999/BR/2016
KY559015/BR/2016
KY559007/BR/2016
KY559005/BR/2016
KY559013/BR/2016
KU991811/IT/BR/2016
KY559027/BR/2016
KU926310/BR/2016
KX197205/BR/2015
KU729218/BR/2015
KY014317/BR/2016
KY014320/BR/2016
KY014296/BR/2016
KU527068/BR/2015
KY441402/BR/2016
KY441403/BR/2016
KU365778/BR/2015
KU365779/BR/2015
KU365780/BR/2015
KU365777/BR/2015
KY014297/BR/2016
KY785450/BR/2016
MH513600/BR/2015
KU729217/BR/2015
KY120352/KR/BR/2016
MH882544/BR/2016
MH882545/BR/2016
MH882543/BR/2016
MH882542/BR/2016
MH882527/BR/2016
MH882535/BR/2016
MH882534/BR/2016
MH882540/BR/2016
MH882533/BR/2016
MH882531/BR/2016
MH882538/BR/2016
KY631492/BR/2016
KU497555/BR/2015
KY785455/BR/2016
KU940228/BR/2015
KX520666/BR/2015
KY441401/BR/2016
KX197192/BR/2015
MF352141/BR/2015
KX421193/UG/1947
KX830960/UG/1947
KX377335/UG/1947
LC002520/UG/1947
KY989511/UG/1947
KU963573/UG/1947
KU955594/UG/1947
MK105975/UG/1947
KX601169/UG/1947
DQ859059/UG/1947
D
S

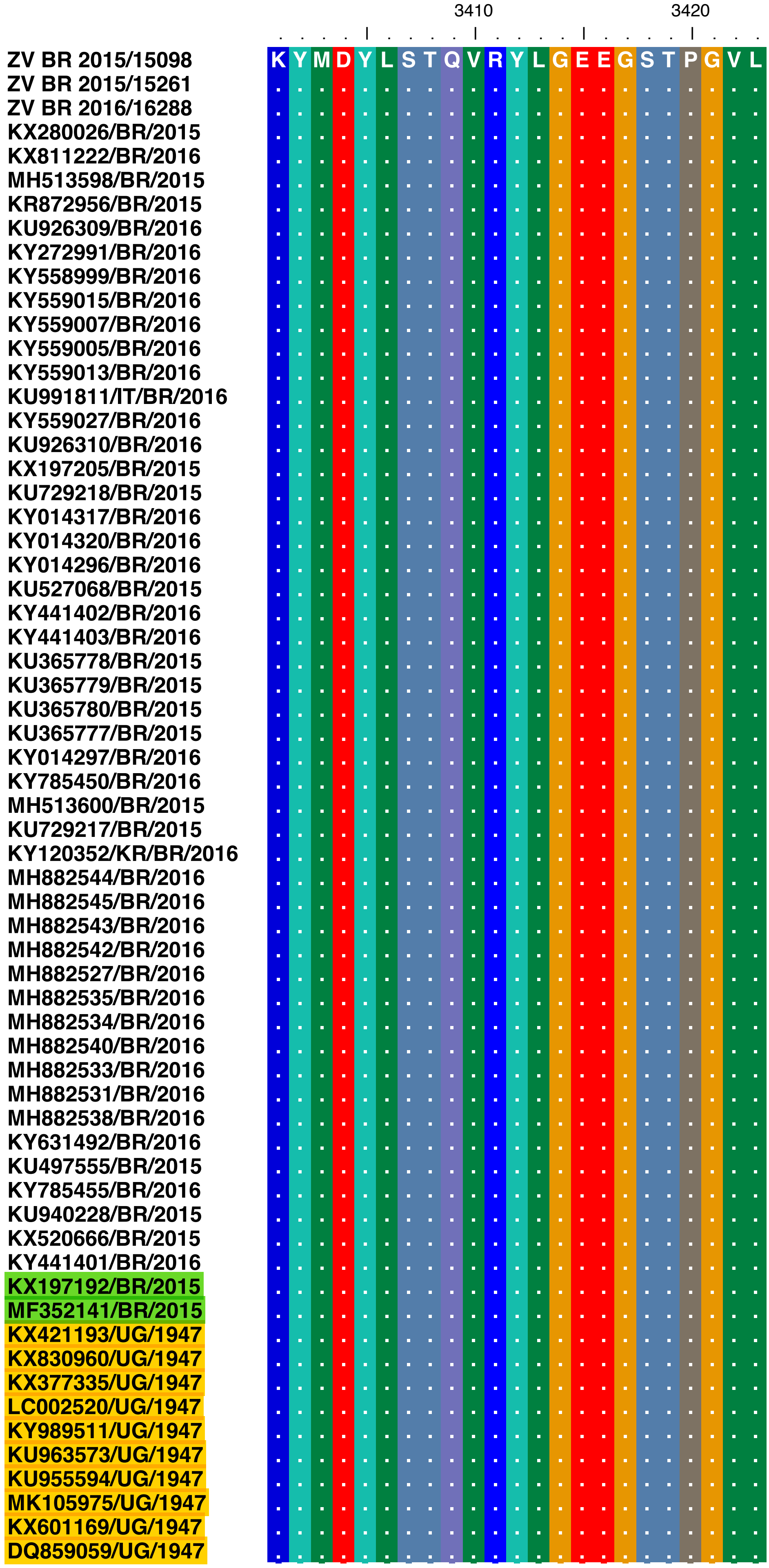

↓: non-synonymous substitutions shown in Table 3

Green highlight: sequences of PE243 strain

Yellow highlight: sequences of MR766 strain

X: codons presenting ambiguous nucleotide notation
